# Synthesis and Evaluation of the Antiproliferative Activity of the Derivatives of 3,5-Diaryl-3,4-dihydro-2*H*-pyrrole-2-carboxylic Acids

**DOI:** 10.3390/molecules30071602

**Published:** 2025-04-03

**Authors:** Vesela Mihaylova, Ivan Iliev, Anelia Vasileva, Elizabeth Mazzio, Bereket Mochona, Nelly Mateeva, Donka Tasheva

**Affiliations:** 1 Department of Organic Chemistry and Pharmacognosy, Faculty of Chemistry and Pharmacy, Sofia University “St. Kliment Ohridski”, 1 J. Bourchier Blvd., 1164 Sofia, Bulgaria; ohvmm@chem.uni-sofia.bg; 2 Institute of Experimental Morphology, Pathology and Anthropology with Museum, Bulgarian Academy of Sciences, 1113 Sofia, Bulgaria; taparsky@abv.bg; 3 Department of Medical Chemistry and Biochemistry, Medical University of Sofia, 2, Zdrave Str., 1431 Sofia, Bulgaria; avasileva@medfac.mu-sofia.bg; 4 College of Pharmacy and Pharmaceutical Science, Florida A&M University, 1415 South M.L. King, Jr. Blvd., Tallahassee, FL 32307, USA; elizabeth.mazzio@famu.edu; 5 Department of Chemistry, Florida A&M University, 1530 South M.L. King, Jr. Blvd., Tallahassee, FL 32307, USA; bereket.mochona@famu.edu (B.M.); nelly.mateeva@famu.edu (N.M.)

**Keywords:** Michael addition, hydrolytic cyclization, 3,4-dihydro-2*H*-pyrrole-2-carbonitriles, in vitro screening, antiproliferative activity, selectivity index

## Abstract

The metabolic cycle of L-proline plays a crucial role in cancer cell survival, proliferation, and metastasis. A key intermediate in the biosynthesis and degradation of proline is 3,4-dihydro-2*H*-pyrrole-2-carboxylic acid. A direct route for synthesizing substituted derivatives of this acid involves the cyclization of 2-amino-5-oxonitriles. Michael additions of [(diphenylmethylene)amino]acetonitrile to enones in a basic medium—either with aqueous sodium hydroxide or under solid–liquid phase-transfer catalysis conditions using CaO as a base—enable the synthesis of substituted 2-amino-5-oxonitriles as single diastereoisomers or as diastereoisomeric mixtures. Selective removal of the diphenylmethylene-protecting group, followed by in situ cyclization in acidic conditions, yields *trans*- and *cis*-3,5-diaryl-3,4-dihydro-2*H*-pyrrole-2-carbonitriles. The reaction of nitriles with HCl/dioxane/methanol followed by treatment with water produces esters and amides as by-products. In vitro screening of the synthesized compounds against multiple human cancer cell lines revealed that some compounds exhibit a good or high selectivity index. In conclusion, the synthetic schemes presented offer simple and efficient routes for the preparation of the derivatives of substituted 3,4-dihydro-2*H*-pyrrole-2-carboxylic acids, with some compounds exhibiting promising antiproliferative activity.

## 1. Introduction

Cancer cells undergo extensive metabolic reprogramming to preserve the redox and energetic balance while meeting the metabolic demand imposed by rapid proliferation [1,2]. Depending on the cancer type and its tissue of origin, alterations in diverse metabolic pathways have been identified that are involved in the metabolism of the main biological macromolecules: lipids, nucleic acids, carbohydrates, and amino acids, including non-essential amino acids (NEAAs) [3]. Among these macromolecules, amino acids are central to multiple metabolic processes in the cell, serving as an alternate energy source to glucose. It has been reported that amino acid transporters and metabolism are up-regulated in numerous malignancies.

One of the NEAAs, L-proline, has a distinctive structure compared to other proteinogenic amino acids and it is the only amino acid with a secondary amino group in the pyrrolidine ring structure and a carboxylic group in position 2. L-proline metabolism is emerging as a key pathway in the metabolic rewiring that sustains cancer cell proliferation, survival, and metastatic spread [4,5,6]. The terminal reaction in the proline metabolic pathway is the reduction of Δ^1^-pyrroline-5-carboxylate (P5C) into proline which is catalyzed by P5C reductase (PYCR1) [7,8]. Various substances of cancer targets are useful as chemical probes for studying cancer mechanisms in drug discovery.

To the best of our knowledge, the biological activity, including the antiproliferative activity, of disubstituted synthetic analogues of ∆^1^-pyrroline-5-carboxylic acid has not been reported. To fill this gap and in continuation of our effort to develop an efficient method for the synthesis of this analogues, herein, we report the synthesis of a series 3,4-dihydro-2*H*-pyrrole-2-carboxylic acid derivatives—nitriles, esters, and amides. We represent our studies of their antiproliferative activity against some human cancer cell lines. The results obtained are compared with those for Cisplatin as a positive control.

## 2. Results and Discussion

### 2.1. Synthesis

**Scheme 1:**
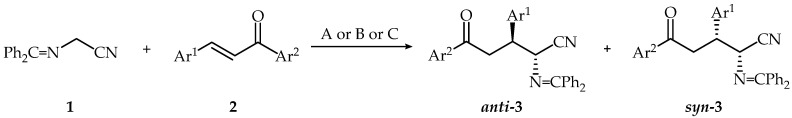
Synthesis of *rel*-(*2R,3S*)-3,5-diaryl-2-((diphenylmethylene)amino)-5-oxo-pentanenitriles (*anti-***3**) and *rel*-(*2R,3R*)-3,5-diaryl-2-((diphenylmethylene)amino)-5-oxo-pentanenitriles (*syn-***3**) obtained as racemates: (A) 33% NaOH, CH_3_CN, 0 °C or (B) 33% NaOH, TEBA, C_6_H_6_, 10 min, r.t. or (C) CaO, Bu_4_NHSO_4_, CH_2_Cl_2_, reflux.

Cyclization of bifunctional compounds—aminoaldehydes and aminoketones [9], oxime esters [10], 5-oxoiminoesters [11], oxoesters, containing a nitro-group [12], and dipolar cycloaddition [13,14]—are widely employed synthetic methods that have been reported to prepare the derivatives of 3,4-dihydro-2*H*-pyrrole-2-carboxylic acid. O’Donnell Schiff bases are useful anionic synthons for ∆^1^-pyrroline synthesis—by diastereoselective or enantioselective 1,4-addition of α-iminoesters and α-iminonitriles to α,β-unsaturated esters or carbonyl compounds, respectively, and the subsequent intramolecular cyclization [15,16,17,18,19,20,21,22,23]. Michael addition of [(diphenylmethylene)amino]acetonitrile (**1**) to some arylmethyleneacetophenones (**2**) was carried out in aqueous conditions by using 33% NaOH and CH_3_CN at 0 °C—the conditions previously described by us [19], in phase-transfer catalysis conditions (PTC)—33% NaOH, TEBA, C_6_H_6_, r.t., or solid CaO, Bu_4_NHSO_4_, CH_2_Cl_2_, under reflux (Scheme 1, Table 1 and Table 2).

As shown in Table 1, the reactions of nitrile **1** and chalcones **2** proceeded with moderate to high yields by using 33% NaOH in CH_3_CN solution at 0 °C. It should be noted that a strong electron-withdrawing substituent as a nitro-group in aromatic ring Ar^1^ (**3d**) or pyridine-3-yl moiety (**3h**) favored Michael addition probably by stabilizing the transition state of the addition reaction. The reaction proceeded in shorter time compared to chalcones with electron-donating substituents in Ar^1^. The electron-donating groups in Ar^1^ increase the transition state energy and the reaction time. The strong electron-donating methoxy group at the position 4 of the aromatic ring Ar^2^ which is conjugated with a carbonyl group by resonance effect lead to the increase in the transition state energy of the reaction and the reaction time. Product **3d**, containing a nitro-group in aromatic ring Ar^1^ was obtained in a high yield; and on the other hand, a product with a strong electron-donating 4-(CH_3_)_2_N group in Ar^1^ (**3c**) obtained in moderate yields. It should be noted that when the reaction mixtures crystalized, high diastereoselectivity was obtained, and the main diastereoisomer was isolated by treatment of crude crystalline products with methanol or by recrystallization. In cases when the reaction mixture did not crystallize, the reaction proceeded with low diastereoselectivity. The oxonitriles **3d** and **3g** were isolated as diastereoisomeric mixtures by column chromatography. We propose that the high diastereoselectivity obtained in the majority of products (**3a** to **3l**) is due to the asymmetric transformation of the second kind, as we have observed previously [24].

A *rel*-(2*R*,3*S*)-configuration was assigned to the major diastereoisomers of oxonitriles **3** (*anti*-**3**) based on a comparison of their ^1^H NMR spectra with those of previously obtained oxonitriles [19]. The chemical shifts for CH_2_ protons for all compounds are in interval 3.65–3.77 and 3.71–3.78 ppm and the coupling constants between H-4 and H-3 atoms are 4.8–5.5 Hz and 8.4–9.0 Hz, respectively. The Michael addition reactions on some substituted chalcones proceeded with high yield and low diastereoselectivity in phase-transfer catalyzed conditions (Method B) for short reaction times (10 min). Under these conditions, the reaction mixtures did not crystalize, and oxonitrides **3** were obtained as diastereoisomeric mixtures, with *rel*-(2*R*,3*R*)-configuration (*syn*-isomers) being the major isomers according to ^1^H NMR (Table 1). It should be noted that the chemical shifts for CH_2_ protons for all compounds with *rel*-(2*R*,3*R*)-configuration (*syn*-isomers) are in the intervals 3.46–3.63 and 3.86–4.00 ppm, and coupling constants between H-4 and H-3 atoms are 5.3–5.4 Hz and 8.1–8.4 Hz, respectively. The crude products were purified by column chromatography, and the final product **3** were isolated as a diastereoisomeric mixture. Despite multiple attempts, we were unable to isolate pure diastereoisomers.

We carried out the reaction of [(diphenylmethylene)amino]acetonitrile (**1**) and three chalcones **2**, which contain a halogen atom and a methyl group on the Ar^1^ ring and an OH group at position 2 and methoxy groups at positions 4 and 6 of the aromatic ring Ar^2^ (Table 2).

Under the described conditions (33% NaOH, CH_3_CN, 0 °C), the reaction was proceeded for an extended period (6 h), and resulted in a low yield product **3m**—23%. Moreover, many by-products were detected (TLC). To optimize the reaction conditions for preparing oxonitrile **3m** with improved yield and diastereoselectivity, we carried out the Michael addition reaction of compound **1** with **2m** using different bases, solvents, catalysts and temperatures. Arylmethyleneacetophenones, containing an OH group on aromatic rings, were obtained and converted to flavanones by using LiOH as a base [25,26,27]. The cyclizations proceeded with high yields attributed to the coordination of lithium ion with a carbonyl oxygen atom. Performing the reaction with LiOH either as a saturated solution or solid in CH_3_CN, CH_2_Cl_2_, ethanol or toluene, in the presence of TEBA as a catalyst) provided **3m** as a 1:1 diasteriomeric mixture in a moderate yield (60%). Calcium ions form more stable complexes with oxygen atoms in ligands compared to lithium ions, which gave us the reason to use calcium oxide as a base. Previous studies have reported the successful use of CaO in the synthesis of arylmethyleneacetophenones containing hydroxy groups in aromatic rings [28]. Performing the reaction in solid–liquid phase-transfer catalysis conditions (CaO, Bu_4_NHSO_4_, CH_2_Cl_2_, reflux), provided **3m** as a mixture of diastereomers confirmed by ^1^H NMR spectroscopy and isolated as an oil in 88% yield. Under these optimized conditions, the products **3n** and **3o** also were obtained as diastereoisomeric mixtures confirmed by ^1^H NMR spectroscopy and isolated in high yields following column chromatography (Table 2). The high yields of **3m**–**o** are most likely due to the formation of a complex between the calcium ions and the carbonyl oxygen atom and the oxygen atom from the deprotonated OH group of the Ar^2^ ring.

**Scheme 2:**
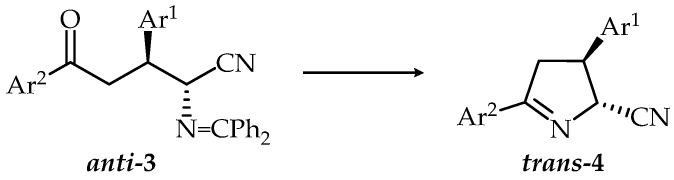
Synthesis of *rel*-(2*R*,3*S*)-3,5-diaryl-3,4-dihydro-2*H*-pyrrole-2-carbonitriles (*trans*-**4**) obtained as racemates: 1. 20% HCl, (C_2_H_5_)_2_O, MeOH, r.t., 2. aq. NH_3_, CH_2_Cl_2_.

It is known that the diphenylmethylene—[29,30] or alkylidene-protecting groups [17,31]—could easily be removed under acidic conditions. Treatment of *anti*-oxonitriles **3** with 20% aqueous HCl in Et_2_O/MeOH solution at room temperature led to hydrolysis of the azomethine group followed by in situ cyclization (Scheme 2, Table 3) to provide the corresponding cyclic products **4** as single diastereoisomers.

Reactions proceeded in different reaction times—30 min for **3c** and 300 min for **3e** and **3f**. Based on the similarity of chemical shifts for H2, H3, and H4 in the ^1^H NMR of dihydropyrrole ring and the values of coupling constants ^3^*J* (H2-H3) of the isolated single diastereoisomers and compound **4a**, that previously reported by us [19], we assigned *trans*-configuration to these diastereoisomers. It was further established that the hydrolytic cyclization reactions of *anti*-3 proceeded without epimerization, as confirmed by ^1^H NMR. This finding aligns with our previous observations [19] and is consistent with reports from other studies [16].

**Scheme 3:**
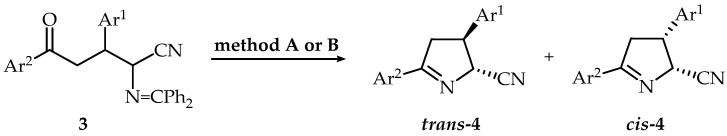
Synthesis of *rel*-(2*R*,3*S*)-3,5-diaryl-3,4-dihydro-2*H*-pyrrole-2-carbonitriles (*trans*-**4**) and *rel*-(2*R*,3*R*)-3,5-diaryl-3,4-dihydro-2*H*-pyrrole-2-carbonitriles (*cis*-**4**) obtained as racemates: (A) 1. 20% HCl, (C_2_H_5_)_2_O, MeOH, r.t., 2. aq. NH_3_, CH_2_Cl_2_; (B) 1. CH_3_COOH, H_2_O, r.t., 2. aq. NH_3_, CH_2_Cl_2_.

The cyclization of some diastereoisomeric mixtures (**3d**, **3g**, **3m**–**o**) was performed under the described conditions. After purification by column chromatography, *trans*-**4g**, *trans*-**4m**–**o**, and *cis*-**4g**, *cis*-**4m**–**o** were isolated as single diastereoisomers. It is worth noting that, in some cases, the isolation of product 4 was challenging due to its high solubility in both organic and aqueous layers. To address the issue, we performed cyclization reactions of some diastereoisomeric mixtures of oxonitriles **3** in mildly acidic conditions with CH_3_COOH. In all cases, the reactions proceeded without epimerization as confirmed by ^1^H NMR and *trans*-**4** and *cis*-**4** were isolated as single diastereoisomers with good yields as oils (Scheme 3, Table 3).

**Scheme 4:**
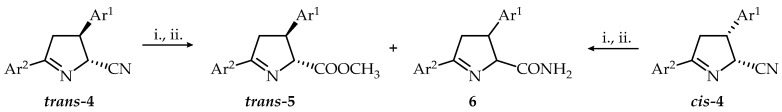
Synthesis of methyl *rel*-(2*R*,3*S*)-3,5-diaryl-3,4-dihydro-2*H*-pyrrole-2-carboxylates (*trans*-**5**) obtained as racemates: (i). 4 M HCl/dioxane/CH_3_OH; (ii). H_2_O, NaHCO_3_.

Pinner reaction is a direct route for the conversion of nitriles to esters [32,33,34,35]. The next step is the conversion of nitriles **4** to methyl esters by using HCl/dioxane in dry MeOH (Scheme 4, Table 4). To the best of our knowledge, this reaction has not been applied to the 3,4-dihydro-2*H*-pyrrole-2-carbonitriles.

Compounds *trans*-**4** were treated with excess of HCl in methanol solution at room temperature and after work up under aqueous conditions, methyl esters **5** with *trans*-configuration confirmed by ^1^H NMR were obtained for different reaction times (120–420 min). In all cases, crude products contained both ***cis***- and *trans*-isomers of the corresponding amides (^1^H NMR). *Trans*-**5** were isolated with moderate yields. During the cyclization of compound *trans*-**4i** we succeeded to isolate not only the ester *trans*-**5**, but also the amide **6i** as a mixture of diastereoisomers. Under the described conditions, the reaction of nitriles *cis*-**4a** and *cis***-4g** proceeds with epimerization over an extended reaction time. Esters *trans*-**5a**, *trans*-**5g** were obtained and isolated with good yields as oils. Starting with the nitrile *cis*-**4m** under the described conditions and over a long reaction time, the amide *cis*-**6m** was obtained and isolated as a crystalline product.

The structures of compounds **3**, **4**, **5**, and **6** were confirmed by IR, ^1^H NMR, ^13^C NMR, and HRMS. The structures of the most abundant fragment ions observed in MS/MS spectra of compounds are proposed (Supplementary Materials).

### 2.2. Antiproliferative Activity

Antiproliferative activity of some compounds was evaluated on cell cultures derived from MDA-MB-231 (triple negative human breast carcinoma). We found that some nitriles, namely **4**, exhibited relatively promising antiproliferative activity (e.g., *cis*-**4m**), while esters **5** demonstrated poor activity (IC_50_ > 4100 µM), regardless of the substituent type on the aromatic rings Ar^1^ or Ar^2^. This finding allowed us to further investigate the antiproliferative activity of selected compounds on additional tumor cell lines. We studied the influence of the stereochemical orientation of the substituents at C2 and C3 in substituted 3,4-dihydro-2*H*-pyrrole-2-carbonitriles. The antiproliferative activity of single diastereomers of some nitriles **4** and the amide *cis*-**6m** were assessed on multiple human cancer cell lines via the MTT assay [36]. For these experiments, the following human cell lines were used: MCF-7 (luminal type A breast carcinoma), MD-MBA-231, H1299 (non-small-cell lung carcinoma cells), A549 (lung alveolar adenocarcinoma), HeLa (cervical cancer), HepG2 (hepatocellular carcinoma), HT-29 (colorectal adenocarcinoma) and PC3 (prostate adenocarcinoma). As a model of normal tissue**,** we used MCF-10A (non-tumorigenic epithelial cells of mammary gland). Table 5 and Table 6 present the values of concentration that inhibits cell viability to 50% (IC_50_) and selectivity index (SI = IC_50_(MCF-10A)/IC_50_(tumor cell line)).

Analysis of the results in Table 5 revealed that the *trans*-**4k** with the methyl group in aromatic ring Ar^2^ shows relativity promising antiproliferative activity (IC_50_ = 11.7 µM) and high SI (15.5) against cells of lung alveolar adenocarcinoma A549. On the other hand, *cis*-**4k** shows significantly poor activity (IC_50_ = 82.2 µM) and very low SI (1.27). Compound *cis*-**4h** has good SI (7.33), but low antiproliferative activity (IC_50_ = 311 µM).

Cisplatin is a well-known chemotherapeutic agent, which has been used for treatment of numerous human cancers by inducing apoptosis in cancer cells [37]. We used Cisplatin as a positive control. From the results in Table 5, it can be seen that for the cell line A549 the IC_50_ value of compound *trans*-**4k** is 3.3 times lower than the IC_50_ of Cisplatin. There is a good linear correlation between the IC_50_ values for the two compounds against four cell lines: MCF-10A, HepG2, HT-29 and PC3. Furthermore, the SI of *trans*-**4k** was 26 times higher than SI of Cisplatin. Based on these results, we suggest that compound *trans*-**4k** is very promising against A549 (lung alveolar adenocarcinoma).

Arylmethyleneacetophenones, containing an OH group at position 2 and/or hydroxy and alkoxy groups at positions 4 and 6 on the aromatic ring Ar^2^ are precursors of flavonoids [38]—compounds with antitumor activity. For this reason, we continued our investigations with compounds **4m**–**o**.

Preliminary experiments of the antiproliferative activity of nitriles **4m**–**o** showed that compounds **4n** and **4o**, containing chlorine or bromine as substituent, against MDA-MB-231 cells, have poor activity—*cis*-**4n** (IC_50_ = 75.8 µM) and *cis*-**4o** (IC_50_ = 35.9 µM), respectively. *Trans*-**4n** and *trans*-**4o** demonstrated very poor activity. Based on these findings, we proceeded with further experiments on the stereoisomers of the fluorine-containing nitrile **4m**.

As can be seen from Table 6 the high activity (IC_50_ = 16 µM) and high SI (10.5) of the nitrile *cis*-**4m** against MDA-MB-231 cell line were observed. This compound possesses moderate activity against H1299 (IC_50_ = 25.4 µM) and HT-29 (IC_50_ = 19.6 µM) cell lines and good SI—6.68 and 8.86, respectively. In contrast, *trans*-**4m** exhibited poor activity and a poor selectivity index across all tumor cell lines. The amide *cis*-**6m** unlike the nitrile *cis*-**4m** has poor activity against all tumor cell lines. It should be noted that against the HT-29 cells, this compound exhibits good SI (6.67). These data indicate that the nitrile group at position 2 in 3,5-diaryl-3,4-dihydro-2*H*-pyrroles is essential for their biological activity. From the data in Table 5 and Table 6 for the IC_50_ values of the compound *cis*-**4m** and Cisplatin for the cell lines PC3, H1299 and MDA-MB-231, it can be seen that the IC_50_ values of *cis*-**4m** are 2.5, 5 and 9 times higher, respectively. There is a good linear correlation between the IC_50_ values for the two compounds against cell lines: MCF-7, MDA-MB-231, H1299, HepG2 and HT-29. Comparing the SI values it can be seen that for the cell lines MDA-MB-231, H1299 the SI values of the two compounds are similar, while for the cell line HT-29 the SI of *cis*-**4m** is 3 times higher.

According to the data presented in Table 5 and Table 6, we can suggest that the most resistant cell lines to all compounds are MCF-7 and HeLa cell lines. Compound *cis*-**4m** has good IC_50_ values and SI for PC3, H1299 and MDA-MB-231 cell lines. According to the data in Table 5, for Cisplatin and compound *trans*-**4k** we can propose, that its IC_50_ is 3.3 times lower than IC_50_ of Cisplatin and is 26 times higher than SI of Cisplatin. We can assume that this compound is very promising against cells of lung alveolar adenocarcinoma A549.

## 3. Materials and Methods

### 3.1. General Information

Benzophenonimine [39], [(diphenylmethylene)amino]acetonitrile [30], and the derivatives of benzylideneacetophenone **2a**–**l** [40,41,42] and chalcones **2m**–**o** [43] were obtained using methods described in the literature. The hydrochloride of aminoacetonitrile (Fluka, Seelze, Germany), 1,3-diphenyl-2-propen-1-one (benzylideneacetophenone (chalcone)) (Merck, Darmstadt, Germany), substituted acetophenones (Merck) and aldehydes (Fluka), NaOH (Sigma-Aldrich, St. Louis, MO, USA), CaO (Fluka), solid LiOH·H_2_O (Acros, Newark, NJ, USA), quaternary ammonium salts (benzyltriethylammonium chloride (TEBA) and tetrabutylammonium hydrogen sulfate (Bu_4_NHSO_4_) (Fluka), HCl (Sigma-Aldrich), acetic acid (Merck), and a 4M solution of hydrogen chloride in dioxane (Fluka), as well as the solvents used are commercial products and were used without further purification. For thin-layer chromatography, aluminum plates (Merck) with silica gel 60 F_254_ were used. Silica gel 60 (0.063–0.200 mm) was used for column chromatography. Melting points were determined on a Boetius micromelting point apparatus and were uncorrected. Infrared spectra (FT-IR) were acquired on a Nicolet 6700 FT-IR Thermo Scientific infrared spectrophotometer (Thermo Fisher Scientific, Waltham, MA, USA). NMR spectra (^1^H NMR, ^13^C NMR, ^1^H-^1^H COSY, HSQC, HMBC and 2D-NOESY) were recorded on a Bruker Avance DRX 250 (^1^H-250.13 MHz and ^13^C-62.90 MHz), Bruker Avance II+ 600 (^1^H-600.01 MHz and ^13^C-150.87 MHz), and Bruker Avance III HD 500 (^1^H-500.13 MHz and ^13^C-125.76 MHz) spectrometers (Bruker BioSpin GmbH, Rheinstetten, Germany). CDCl_3_ or DMSO-*d*_6_ were used as solvents. Chemical shifts (*δ*) are reported in parts per million (ppm). ^1^H NMR spectra were referenced to the tetramethylsilane (TMS) as an internal standard in CDCl_3_ or residual solvent signal at 2.50 ppm in DMSO-*d*_6_. ^13^C NMR spectra were calibrated according to TMS as an internal standard or the carbon atom signal (at 77.0 ppm in CDCl_3_ or at 39.52 ppm in DMSO-*d*_6_). Coupling constants (*J*) were measured in hertz (Hz).

#### 3.1.1. LC-HRMS Analyses

The analyses were performed using a Q Exactive hybrid quadrupole-Orbitrap mass spectrometer (Thetmo Scientific Co., Waltham, MA, USA) equipped with TurboFlow^®^ LC system, heated electrospray model HESI II on IonMax^®^ (Thetmo Scientific Co., USA).

The chromatographic separation of the analytes was conducted using an HPLC column NUCLEODUR C18 Gravity (MACHEREY-NAGEL GmbH & Co. KG, Dueren, Germany, 100 × 2 mm i.d., 1.8 μm) using the following mobile phases: A: 0.1% formic acid in water and B: 0.1% formic acid in acetonitrile at flow rate of 300 μL/min and gradient as follows: 0% B for a minute, 100% B for 12 min, 100–0% B for 1 min, and % B for 2 min. The injection volume was 10.0 μL. Only the elution fraction between 0 and 15 min of the gradient was injected in the mass spectrometer.

Full-scan spectra over the *m*/*z* range 100–500 were acquired in positive ion mode at resolution settings of 70,000. All MS parameters were optimized for sensitivity to the target analytes using the instrument control software program (Build 2926, version 2.9). Q Exactive parameters were: spray voltage 4.0 kV, sheath gas flow rate 32, auxiliary gas flow rate 10, spare gas flow rate 3 L/min, capillary temperature 320 °C, probe heater temperature 300 °C and S-lens RF level 50. All Ion Fragmentation mode of operation of the mass analyzer was used for compounds identification. Optimized values of the collision energy were HCD 25%. All MS parameters were optimized for sensitivity to the target analytes using the instrument control software program. Data acquisition and processing were carried out with the Xcalibur 2.4^®^ software package (Thermo Scientific Co., USA). Calculations of theoretical *m*/*z* values were made by the Mass Frontier 5.1 Software program (Thermo Scientific Co., USA).

In the Supplementary Materials are given the UPLC-DAD chromatograms of the compounds presented in Table 5 and Table 6.

#### 3.1.2. In Vitro Antiproliferative Activity

The antiproliferative activity testing was performed on cell cultures from several human cancer cell lines the MTT-dye reduction assay, described by Mosmann [36]. Cell lines were obtained from the American Type Cultures Collection (ATCC, Manassas, VA, USA). In this article we used the following cell lines: MCF-10A (ATCC, CRL-10317™) Non-tumorigenic epithelial cells of mammary gland, MCF-7 (ATCC, HTB-22™) Luminal type A breast carcinoma, MDA-MB-231 (ATCC, HTB-26™) Triple negative breast carcinoma, H1299 (ATCC, CRL-5803™) Non-small-cell lung carcinoma, A549 (ATCC, CRM-CCL-185™) Llung alveolar adenocarcinoma, HeLa (ATCC, CCL-2™) Cervical cancer, Hep G2 (ATCC, HB-8065™) Hepatocellular carcinoma, HT-29 (ATCC, HTB-38™) Colorectal adenocarcinoma, PC-3 (ATCC, CRL-1435™) Prostate adenocarcinoma. The cells were cultured in a cell culture laboratory at the Institute of Experimental Morphology, Pathology and Anthropology with a Museum—Bulgarian Academy of Sciences. They were cultured in Dulbecco’s Modified Eagle’s medium, high glucose (DMEM 4.5 g/L glucose), supplied with 10% fetal bovine serum and antibiotics in usual concentrations in a humidified atmosphere with 5% CO_2_ at 37.5 °C. In the case of MCF-10A cells, epidermal growth factor, insulin and cholera toxin were added in concentrations corresponding to the cell bank instructions. Cells were plated at a density of 1 × 10^3^ cells per well in a 96-well flat-bottomed microplates and allowed to adhere for 24 h before treatment with the test compounds, dissolved in DMSO, and further diluted in culture medium to the final concentrations. A concentration ranges from 5 µM to 4.1 mM was tested over an incubation period of 72 h. Cisplatin (Sigma-Aldrich) was used as a positive control in a concentration range 50 nM to 100 µM. All experiments were performed in triplicates. The MTT-formazan absorption was measured using a microplate reader (TECAN, Sunrise^TM^, Groedig/Salzburg, Austria) at 570 nm. Antiproliferative activities were expressed as IC_50_ values (concentrations required for 50% inhibition of cell growth), calculated using non-linear regression analysis (GraphPad Prizm8 Software). The statistical analysis involved One-way ANOVA followed by Bonferroni’s post hoc test *p* < 0.05 was accepted as the lowest level of statistical significance.

### 3.2. Synthesis and Characterization

#### 3.2.1. Synthesis of 3,5-Diaryl-2-[(diphenylmethylene)amino]-5-oxonitriles (**3a**–**o**), General Procedure

Method A: 33% aqueous NaOH, CH_3_CN, 0 °C

An aqueous solution of NaOH (33%, 0.75 mL, 8.4 mmol), cooled at 0 °C, was added dropwise to a mixture of 0.55 g (2.5 mmol) of [(diphenylmethylene)amino]acetonitrile (**1**) and 2.5 mmol of the corresponding arylmethyleneacetophenone (**2a**–**h**) in 1.25 mL CH_3_CN. The reaction mixture was stirred at the same temperature for 10 min (**3d**), 20 min (**3h**), 30 min (**3a**, **3b**, **3c**, **3f**), 60 min (**3e**) or 240 min (**3g**). Water (50 mL) was added, and the solid product was filtered, washed with water to neutralize, and dried. The crude products **3a**, **3b**, **3e**, **3f**, were stirred with 15 mL of cold CH_3_OH to give white crystals. Product **3c** was recrystallized from CH_3_CN and 3h from 2-propanol–hexane. For compounds **3d**, and **3g**, water (50 mL) was added to the reaction mixture, the resulting emulsion was extracted with dichloromethane (3 × 25 mL), washed with water to neutral and dried (Na_2_SO_4_). The oily residue obtained after removing of the solvent was purified by column chromatography.

Method B: 33% aqueous NaOH, TEBA, C_6_H_6_, room temperature

An aqueous sodium hydroxide NaOH solution (33%, 0.75 mL, 8.4 mmol) was added dropwise to a solution of 0.55 g (2.5 mmol) of [(diphenylmethylene)amino]acetonitrile (**1**) and 2.5 mmol of the corresponding arylmethyleneacetophenone (**2a**, **2b**, **2e**, **2f**, **2h**–**l**) and 0.03 g (0.13 mmol) benzyltriethylammonium chloride (TEBA) in 1.25 mL of C_6_H_6_. The reaction mixture was stirred at room temperature for 10 min. Water (50 mL) was added, and the resulting emulsion was extracted with dichloromethane (3 × 25 mL). The combined organic layers were washed with water to neutral and dried (Na_2_SO_4_). The oily residue obtained after removing of the solvent was purified by column chromatography (SiO_2_, a mobile phase (petroleum ether: acetone or petroleum ether: ethyl acetate with different polarity)) to give **3a**, **3b**, **3e**, **3f**, **3h**–**l**.

Method C: solid CaO, Bu_4_NHSO_4_, CH_2_Cl_2_, reflux.

1 mmol of the corresponding arylmethyleneacetophenone (**2m**–**o**) and 0.034 g (0.1 mmol) Bu_4_NHSO_4_ in dry CH_2_Cl_2_ (1.20 mL), CaO (0.561 g, 10 mmol) were added to a stirred solution of 0.220 g (1 mmol) [(diphenylmethylene)amino]acetonitrile (**1**). The reaction mixture was refluxed for 125 min (**3m**), 130 min (**3n**), and 335 min (**3o**). After that, water (~30 mL) was added, and acetic acid was used to dissolve the solid Ca(OH)_2_ (pH~7). The resulting emulsion was extracted with dichloromethane (3 × 25 mL). The combined extracts were washed with water and dried (Na_2_SO_4_). After the solvent evaporation, the oily product obtained was purified by column chromatography (SiO_2_, mobile phase (petroleum ether: acetone = 10:1) to give the compounds **3m**–**o** as colorless oils.

#### 3.2.2. Synthesis of 3,5-Diaryl-3,4-dihydro-2*H*-pyrrole-2-carbonitriles (Δ^1^-Pyrroline-5-carbonitriles) (**4a**–**h**, **4m**–**o**), General Procedure

Method A: 6.25 mL 20% HCl were added to a suspension of 1 mmol of the corresponding 3,5-diaryl-2-[(diphenylmethylene)amino]-5-oxopentanenitrile (**3a**–**h**, **3m**–**o**) in 10 mL diethyl ether and 1 mL methanol. The reaction mixture was stirred at room temperature until the complete disappearance of the starting **3** (30 min (**3c**), 120 min (**3a**, **3m**–**o**), 180 min (**3b**), 210 min (**3d**, **3h**), 300 min (**3e**, **3f**) or 24 h (**3g**)), and worked up as follows:

Work up (A) (the hydrochlorides of **4a**, **4b**, **4d**–**f**, **4m**–**o** were soluble in both the aqueous and organic layers): the organic solvents were removed under the reduced pressure and 15 mL CH_2_Cl_2_ and 6.28 mL (0.084 mol) 25% NH_3_ were added to the water residue and stirred for 3 h at room temperature. Two layers were separated, and the aqueous layer was extracted with CH_2_Cl_2_ (3 × 15 mL). The combined organic extracts were washed with water to neutral, and dried (Na_2_SO_4_), and evaporated under reduced pressure. The resulting oily product was purified by column chromatography (**4a**, **4b**, **4d**, **4m**–**o**). The compounds **4e** and **4f** were obtained by filtration of the hydrochloride salts, washed with hexane, and stirred the crystals in 15 mL CH_2_Cl_2_ with 6.28 mL 25% NH_3_ at room temperature. Than it was extracted (3 × 15 mL CH_2_Cl_2_), washed to neutral, dried (Na_2_SO_4_), and evaporated to dryness under reduced pressure. These compounds required no further purification.

Work up (B) (the hydrochloride salts of **4c**, **4g**, **4h** are soluble in the aqueous layer): The two layers were separated, and the aqueous layer was washed with 20 mL of diethyl ether to remove benzophenone obtained, then 15 mL CH_2_Cl_2_ and 6.28 mL 25% NH_3_ (0.084 mol) were added, and the mixture was stirred for 3 h at room temperature. After extraction, drying (Na_2_SO_4_), and evaporation of the organic solvent, the resulting oily product was purified by column chromatography (**4c**, **4g**). Compound **4h** was isolated as a chromatographically pure substance, requiring no further purification.

Method B: Glacial acetic acid (5.71 mL, 0.100 mmol) and water (1 mL) were added to 1 mmol of the starting 2-amino-5-oxo-pentanenitrile **3a**, **3b**, **3e**–**l** (diastereoisomeric mixture). The reaction mixture was stirred at room temperature until the complete disappearance of the starting compound (TLC). Dichloromethane (15 mL), and 25% NH_3_ (6.28 mL, 0.084 mol) were added to alkaline. The two layers were separated, and the aqueous layer was extracted with dichloromethane (3 × 15 mL). The combined organic extracts were washed with water to neutral, dried (Na_2_SO_4_), and evaporated to dryness under reduced pressure. The resulting crude oily product was purified by column chromatography (SiO_2_, mobile phase—petroleum ether: acetone or cyclohexane: ethyl acetate with variable polarity).

#### 3.2.3. Reaction of 3,5-Diaryl-3,4-dihydro-2*H*-pyrrole-2-carbonitriles with 4 M HCl/Dioxane in Dry Methanol, General Procedure

To 1 mmol of the starting 3,5-diaryl-3,4-dihydro-2*H*-pyrrole-2-carbonitrile in 5 mL dry methanol, 3.75 mL (15 equivalents) or 5.75 mL (23 equivalents) of a 4 M hydrogen chloride solution in dioxane were added (15 equivalents for *trans*-**4a**, *trans*-**4b**, **4d** (diastereoisomeric mixture), *trans*-**4e**, *trans*-**4f**, *trans*-**4i**–**l**, and 23 equivalents for *cis*-**4a**, *trans*-**4c**, *trans*-**4g**, *cis*-**5g**). For carbonitriles *trans*-**4m** or *cis*-**4m** (0.4 mmol), 2 mL of absolute methanol and 2.30 mL (23 equivalents) of a 4 M hydrogen chloride solution in dioxane were used. The reaction mixture was stirred at room temperature until the complete disappearance of the starting compound (TLC), and the solvents were removed under reduced pressure. Dichloromethane (15 mL) and a saturated NaHCO_3_ solution to alkaline were added. Two layers were separated, and the aqueous layer was extracted with dichloromethane (2 × 15 mL). The combined organic extracts were washed with water until neutral, dried (Na_2_SO_4_), and evaporated to dryness under reduced pressure. The resulting crude oily product was purified by column chromatography (SiO_2_, using as a mobile phase petroleum ether: acetone or petroleum ether: ethyl acetate with different polarity).

## 4. Conclusions

In conclusion, we present a simple and efficient route for synthesizing the derivatives of 3,5-disubstituted 3,4-dihydro-2*H*-pyrrole-2-carboxylic acids. Michael addition of aminoacetonitrile with a diphenylmethylene-protecting group to arylmethyleneacetophenones was performed in basic conditions, including with CaO as a base and PTC in some cases. The formation of a complex between the calcium ions and the carbonyl oxygen atom and the oxygen atom from the deprotonated OH group of the Ar^2^ ring significantly increased in the yields of the products **3m**–**o** and decreased the reaction time. The substituted 2-amino-5-oxonitriles were converted to *trans*- and *cis*-3,5-diaryl-3,4-dihydro-2*H*-pyrrole-2-carbonitriles. The direct conversion of cyclic nitriles to esters with HCl/dioxane/methanol is reported. Amides were obtained as by-products after treatment with water. In vitro screening of selected compounds for antiproliferative activity against various human tumor cell lines was conducted. The compound *rel*-(*2R*,*3S*)-5-(4-methylphenyl)-3-phenyl-3,4-dihydro-2*H*-pyrrole-2-carbonitrile (*trans*-**4k**) shows most potent, selective against cells of lung alveolar adenocarcinoma A549—its IC_50_ value is 3.3 times lower than the IC_50_ of Cisplatin and its SI is 26 times higher than SI of Cisplatin. Compound *rel*-(*2R*,*3S*)-3-(3-fluoro-4-methylphenyl)-5-(2-hydroxy-4,6-dimethoxyphenyl)-3,4-dihydro-2*H*-pyrrole-2-carbonitrile (*cis*-**4m**) possesses moderate activity against H1299 and HT-29, relatively high antiproliferative activity against MDA-MB-231, and a good selectivity index for these cell lines. Given its promising activity, ***cis*-4m** represents a strong candidate for further development as an antiproliferative agent and *trans*-**4k** is very promising against cells of lung alveolar adenocarcinoma A549. In our future studies, we plan to determine the influence of the most active substances on the cell cycle and the induction of apoptosis in tumor cells.

## Figures and Tables

**Table 1:** Yields and diastereoisomeric ratio of compounds **3a**–**l**.

			Method A ^a^			Method B ^b^	
3	Ar^1^	Ar^2^	Reaction Time (min)	Yield ^c^ (%)	D.R. ^d^ *anti*:*syn*	Yield ^e^ (%)	D.R. ^d^ *anti*:*syn*
**a**	C_6_H_5_	C_6_H_5_	30	84 ^f^	99:1	92	40:60
**b**	4-CH_3_OC_6_H_4_	C_6_H_5_	30	84	92:8	92	39:61
**c**	4-(CH_3_)_2_NC_6_H_4_	C_6_H_5_	30	75	88:12	-	-
**d**	4-NO_2_C_6_H_4_	C_6_H_5_	10	94 ^e^	82:18	-	-
**e**	C_6_H_5_	4-CH_3_OC_6_H_4_	60	87	93:7	89	44:56
**f**	4-ClC_6_H_4_	4-CH_3_OC_6_H_4_	30	85	94:6	91	42:58
**g**	4-FC_6_H_4_	3,4,5-(CH_3_O)_3_C_6_H_2_	240	87 ^e^	49:51	-	-
**h**	pyridine-3-yl	C_6_H_5_	20	67	82:18	84	40:60
**i**	4-CH_3_C_6_H_4_	C_6_H_5_	30	85 ^f^		91	44:56
**j**	4-ClC_6_H_4_	C_6_H_5_	30	85 ^f^		95	40:60
**k**	C_6_H_5_	4-CH_3_C_6_H_4_	30	85 ^f^		85	53:47
**l**	C_6_H_5_	4-ClC_6_H_4_	30	89 ^f^		84	37:63

^a^ Method A (33% NaOH, CH_3_CN, 0 °C); ^b^ Method B (33% NaOH, TEBA, C_6_H_6_, r.t., 10 min); ^c^ yield of *anti*-isomer obtained by treatment with methanol (**3a**, **3b**, **3e**, **3f**) or recrystallization; ^d^ D.R.—diastereoisomeric ratio *anti*:*syn* determined by ^1^H NMR of the crude product; ^e^ yield of diastereoisomeric mixture after column chromatography; ^f^ previously obtained by us [19].

**Table 2:** Yields and diastereoisomeric ratio of compounds **3m**–**o** (Ar^2^ = 2-HO-4,6-(CH_3_O)_2_C_6_H_2_).

Method C ^a^				
3	Ar^1^	Reaction Time (min)	Yield ^b^ (%)	D.R. ^c^ (*anti*:*syn*)
**m**	3-F-4-CH_3_-C_6_H_3_	125	88	44:56
**n**	3-Cl-4-CH_3_-C_6_H_3_	130	91	44:56
**o**	3-Br-4-CH_3_-C_6_H_3_	335	90	45:55

^a^ Method C (CaO, Bu_4_NHSO_4_, CH_2_Cl_2_, reflux); ^b^ yield of diastereoisomeric mixture after column chromatography; ^c^ determined by ^1^H NMR of the crude product.

**Table 3:** Yields and diastereoisomeric ratio of compounds **4a**–**o**.

			Method A ^a^		Method B ^b^	
4	Ar^1^	Ar^2^	Reaction Time (min)	Yield ^c^ (%)	Reaction Time (min)	Yield ^d^ (%) *trans, cis*
**a**	-C_6_H_5_	-C_6_H_5_	120	96	150	39, 46
**b**	-C_6_H_4_(4-OCH_3_)	-C_6_H_5_	180	91	150	30, 43
**c**	-C_6_H_4_(4-(N(CH_3_)_2_)	-C_6_H_5_	30	75	-	-
**d**	-C_6_H_4_(4-NO_2_)	-C_6_H_5_	210	86 ^e^	-	-
**e**	-C_6_H_5_	-C_6_H_4_(4-OCH_3_)	300	78	180	41, 49
**f**	-C_6_H_4_(4-Cl)	-C_6_H_4_(4-OCH_3_)	300	92	360	38, 49
**g**	-C_6_H_4_(4-F)	-C_6_H_2_(3,4,5-OCH_3_)_3_	1440	28, 38 ^d^	360	51, 41
**h**	pyridine-3-yl	-C_6_H_5_	210	86	400	51, 30
**i**	-C_6_H_4_(4-CH_3_)	-C_6_H_5_	-	-	270	36, 43
**j**	-C_6_H_4_(4-Cl)	-C_6_H_5_	-	-	180	45, 43
**k**	-C_6_H_5_	-C_6_H_4_(4-CH_3_)	-	-	180	50, 36
**l**	-C_6_H_5_	-C_6_H_4_(4-Cl)	-	-	205	34, 51
**m**	-C_6_H_3_(3-F-4-CH_3_)	-C_6_H_2_(2-OH-4,6-(OCH_3_)_2_)	120	37, 55 ^d^	-	-
**n**	-C_6_H_3_(3-Cl-4-CH_3_)	-C_6_H_2_(2-OH-4,6-(OCH_3_)_2_)	120	45, 51 ^d^	-	-
**o**	-C_6_H_3_(3-Br-4-CH_3_)	-C_6_H_2_(2-OH-4,6-(OCH_3_)_2_)	120	35, 51 ^d^	-	-

^a^ Method A (1. 20% HCl, (C_2_H_5_)_2_O, MeOH, r.t., 2. aq. NH_3_, CH_2_Cl_2_); ^b^ Method B (1. CH_3_COOH, H_2_O, r.t., 2. aq. NH_3_, CH_2_Cl_2_); ^c^ yield of *trans*-isomer isolated after column chromatography; ^d^ yield of *trans*- and *cis*-diastereoisomers isolated as single isomers after column chromatography; ^e^ yield of diastereoisomeric mixture after column chromatography.

**Table 4:** Yields of compounds *trans*-**5a**–**g**, *trans*-**5i**–**m** and **6m**.

5	Ar^1^	Ar^2^	Reaction Time (min)	Yield ^a^ (%)
a	-C_6_H_5_	-C_6_H_5_	300 1440	59 64 ^b^
b	-C_6_H_4_(4-OCH_3_)	-C_6_H_5_	270	68
c	-C_6_H_4_(4-(N(CH_3_)_2_)	-C_6_H_5_	150	73
d	-C_6_H_4_(4-NO_2_)	-C_6_H_5_	150	39 ^c^
e	-C_6_H_5_	-C_6_H_4_(4-OCH_3_)	270	80
f	-C_6_H_4_(4-Cl)	-C_6_H_4_(4-OCH_3_)	240	71
g	-C_6_H_4_(4-F)	-C_6_H_2_(3,4,5-OCH_3_)_3_	170 1620	- 53 ^b^
i	-C_6_H_4_(4-CH_3_)	-C_6_H_5_	240	64
j	-C_6_H_4_(4-Cl)	-C_6_H_5_	240	63
k	-C_6_H_5_	-C_6_H_4_(4-CH_3_)	420	64
l	-C_6_H_5_	-C_6_H_4_(4-Cl)	240	62
m	-C_6_H_3_(3-F-4-CH_3_)	-C_6_H_2_(2-OH-4,6-(OCH_3_)_2_)	120	68
6m	-C_6_H_3_(3-F-4-CH_3_)	-C_6_H_2_(2-OH-4,6-(OCH_3_)_2_)	1620	56 ^d^

^a^ Yields of *trans*-**5**, obtained by the methanolysis of *trans*-**4** and isolated after column chromatography; ^b^ yields of *trans*-**5** obtained by the methanolysis of *cis*-**4** and isolated after column chromatography ^c^ yield of *trans*-**5**, obtained by the methanolysis of **4** (diastereoisomeric mixture, D.R. *anti*:*syn* = 78:22) and isolated after column chromatography; ^d^ yield of *cis*-**6m** isolated after column chromatography, obtained by the methanolysis of *cis*-**4m**.

**Table 5:** In vitro investigations of the antiproliferative activity of some nitriles. IC_50_ [µM] = concentration that inhibits cell proliferation to 50% (IC_50_); SI = IC_50_(MCF-10A)/IC_50_(tumor cell line).

Cell Line	Cisplatin *	*trans*-4k	*cis*-4k	*cis*-4e	*cis*-4h
	IC_50_ [µM] ± SD (SI)	IC_50_ [µM] ± SD (SI)	IC_50_ [µM] ± SD (SI)	IC_50_ [µM] ± SD (SI)	IC_50_ [µM] ± SD (SI)
MCF-10A	23.2 ± 1.4 (1)	182 ± 5.8 (1)	104 ± 6 (1)	427 ± 12 (1)	2277 ± 178 (1)
MCF-7	45.8 ± 3.2 (0.50)	568 ± 57 (0.32)	374 ± 6 (0.28)	1766 ± 232 (0.24)	1377 ± 255 (1.65)
MDA-MB-231	1.8 ± 0.1 (12.7)	313 ± 20 (0.58)	2562 ± 107 (0.04)	>3619 (<0.12)	>4110 (<0.55)
H1299	5.0 ± 0.9 (4.62)	354 ± 15 (0.51)	205 ± 6 (0.51)	840 ± 43 (0.51)	2984 ± 62 (0.76)
A549	38.6 ± 4.7 (0.60)	11.7 ± 0.8 (15.5)	82.2 ± 5.8 (1.27)	341 ± 26 (1.25)	311 ± 19 (7.33)
HeLa	19.6 ± 3.2 (1.18)	84.1 ± 2.3 (2.16)	169 ± 10 (0.61)	158 ± 6.5 (2.69)	2219 ± 136 (1.03)
HepG2	19.1 ± 0.4 (1.22)	159 ± 4.6 (1.14)	235 ± 15 (0.44)	554 ± 34 (0.77)	2996 ± 107 (0.76)
HT-29	8.2 ± 0.3 (2.84)	136 ± 7.7 (1.33)	853 ± 46 (0.12)	308 ± 8 (1.39)	1640 ± 132 (1.39)
PC3	3.4 ± 0.5 (6.93)	121 ± 13 (1.50)	297 ± 25 (0.35)	478 ± 58 (0.80)	1915 ± 70 (1.19)

* Positive control.

**Table 6:** In vitro investigations of the antiproliferative activity of nitriles and amide-containing hydroxy and methoxy groups in Ar^2^. IC_50_ [µM] = concentration that inhibits cell proliferation to 50% (IC_50_); SI = IC_50_(MCF-10A)/IC_50_(tumor cell line).

Cell Line	*trans*-4m	*cis*-4m	*cis*-6m
	IC_50_ [µM] ± SD (SI)	IC_50_ [µM] ± SD (SI)	IC_50_ [µM] ± SD (SI)
MCF-10A	79.3 ± 2.5 (1)	170 ± 12 (1)	429 ± 28 (1)
MCF-7	149 ± 12 (0.53)	185 ± 26 (0.92)	923 ± 37 (0.46)
MDA-MB-231	717 ± 73 (0.11)	16.0 ± 0.7 (10.5)	1989 ± 40 (0.22)
H1299	36.4 ± 2.0 (2.18)	25.4 ± 2.3 (6.68)	1876 ± 51 (0.23)
A549	32.2 ± 1.1 (2.46)	97.6 ± 13 (1.74)	136 ± 5.4 (3.16)
HeLa	1168 ± 42 (0.07)	224 ± 15 (0.75)	1180 ± 37 (0.36)
HepG2	44.6 ± 2.8 (1.78)	117 ± 4.8 (1.45)	117 ± 6.2 (3.67)
HT-29	28.1 ± 2.0 (2.82)	19.6 ± 1.8 (8.66)	64.3 ± 4.8 (6.67)
PC3	44.3 ± 5.4 (1.79)	115 ± 4.8 (1.47)	125 ± 7.6 (3.44)

## Data Availability

Data are contained within this article and Supplementary Materials.

## Supplementary Materials

The following supporting information can be downloaded at: https://www.mdpi.com/article/10.3390/molecules30071602/s1. Description of physical and spectral data of compounds **3**, **4**, **5**, and **6**; Figures S1–S228: Spectra of compounds (IR, ^1^H NMR,^13^C NMR, HRMS); Figures S229–S235: UPLC-DAD chromatograms of the compounds presented in Table 5 and Table 6. Figure S236: UPLC-DAD chromatogram of the compound **3m**.
