# Supplementary material for: Synthesis and Evaluation of the Antiproliferative Activity of the Derivatives of 3,5-Diaryl-3,4-dihydro-2H-pyrrole-2-carboxylic Acids
Source: Molecules. 2025 Apr 3;30(7):1602. doi: 10.3390/molecules30071602 (PMC11990662; doi:10.3390/molecules30071602)
Supplement: Supplementary file 1 [file molecules-30-01602-s001.zip › Tasheva_2025_Molecules_Supplementary Materials.pdf]

Supplementary Materials:

Synthesis and Evaluation of Antiproliferative Activity of the Derivatives of 3,5-Diaryl-3,4-dihydro-2*H*-pyrrole-2-carboxylic Acids

Vesela Mihaylova [1], Ivan Iliev [2], Anelia Vasileva [3], Elizabeth Mazzio [4], Bereket Mochona [5], Nelly Mateeva [5] and Donka Tasheva [1,*]

[1] Department of Organic Chemistry and Pharmacognosy, Faculty of Chemistry and Pharmacy, Sofia University "St. Kliment Ohridski", 1 J. Bourchier Blvd., 1164 Sofia, Bulgaria; ohvmm@chem.uni-sofia.bg (V.M.)
[2] Institute of Experimental Morphology, Pathology and Anthropology with Museum, Bulgarian Academy of Sciences, Sofia, Bulgaria; taparsky@abv.bg (I.I.)
[3] Department of Medical Chemistry and Biochemistry, Medical University of Sofia, 2, Zdrave Str., Sofia, 1431, Bulgaria; avasileva@medfac.mu-sofia.bg (A.V.)
[4] College of Pharmacy and Pharmaceutical Science, Florida A&M University, 1415 South M.L. King, Jr. Blvd., Tallahassee FL 32307; elizabeth.mazzio@famu.edu (E.M.)
[5] Department of Chemistry, Florida A&M University, 1530 South M.L.King, Jr. Blvd. Tallahassee FL 32307; nelly.mateeva@famu.edu (N.M.); bereket.mochona@famu.edu (B.M.)
* Correspondence: ohdt@chem.uni-sofia.bg; (D.T.) Tel.: (+35928161437)

Description of physical and spectral data of compounds **3**, **4**, **5**, and **6**

3,5-Diaryl-2-2-((Diphenylmethylene)amino)-5-oxo-pentanenitriles

2-((Diphenylmethylene)amino)-5-oxo-3,5-diphenylpentanenitrile (**3a**): Following the general procedure of Method A, after stirring with 15 mL of cold CH_3OH for 30 min, white crystals of **3a** (0.90 g, 84%) were obtained as an individual diastereoisomer, mp 136–138 °C. Following the general procedure of Method B, after purification by column chromatography (SiO_2, petroleum ether: acetone = 15:1), a pale yellow oil of **3a** (0.98 g, 92%) was isolated as a diastereoisomeric mixture, which then crystallized. IR (KBr): 2240 (νCN), 1686 (νC=O), 1618 (νC=N), 1596, 1578, 1494, 1448 (νC=C), 750, 698 (γC-H) cm^{-1}. ^{1}H NMR (500.13 MHz, $CDCl_3$) δ (ppm): *anti*-**3a:** 3.74, 3.85 (2dd, 2H, ^2J = 17.4 Hz, ^3J = 9.1 Hz, ^3J = 4.8 Hz, CH_2CO), 3.98–4.04 (m, 1H, H-3), 4.51 (d, 1H, ^3J = 5.4 Hz, H-2), 6.80–6.81 (m, 2H, Ar), 7.20–7.30 (m, 3H, Ar), 7.34–7.52 (m, 8H, Ar), 7.54–7.58 (m, 3H, Ar), 7.61–7.62 (m, 2H, Ar), 7.95–7.99 (m, 2H, Ar). *syn*-**3a:** ^{1}H NMR (500.13 MHz, $CDCl_3$) δ (ppm): **3**.63, 4.00 (2dd, 2H, ^2J = 17.1 Hz, ^3J = 8.1 Hz, ^3J = 5.4 Hz, CH_2CO), 3.91 (dt, 1H, ^3J = 8.1 Hz, ^3J = 5.4 Hz, H-3), 4.51 (d, 1H, ^3J = 5.4 Hz, H-2), 7.08-7.10 (m, 2H, Ar), 7.20–7.30 (m, 8H, Ar), 7.34–7.52 (m, 8H, Ar), 7.95–7.99 (m, 2H, Ar). ^{13}C NMR (125.76 MHz, $CDCl_3$) δ (ppm): *anti*-**3a:** 39.68 (C-3), 45.19 (C-4), 57.95 (C-2), 118.47 (CN), 127.08, 127.53, 128.10, 128.20, 128.54, 128.66, 128.85, 129.06, 129.20, 131.31, 133.24, 134.98, 136.79, 138.25, 139.02, 174.21 (C=N), 197.33 (C-5). *syn*-**3a:** 39.29 (C-3), 45.07 (C-4), 58.72 (C-2), 118.47 (CN), 127.30, 127.60, 128.14, 128.26, 128.50, 128.63, 128.97, 129.04, 129.42, 131.24, 133.26, 135.03, 136.84, 138.21, 139.06, 173.68 (C=N), 197.33 (C-5). HRMS (ESI) (diastereoisomeric mixture): calculated for $C_{30}H_{24}N_2O$ [M + H]$^+$ *m/z* 429.1961, found 429.1956.

2-((Diphenylmethylene)amino)-3-(4-methoxyphenyl)-5-oxo-5-phenylpentanenitrile (**3b**): Following the general procedure of Method A, after stirring with 15 mL of cold CH_3OH for 60 min, white crystals of **3b** (0.97 g, 84%) as an individual diastereoisomer, mp 153–155 °C. Following the general procedure of Method B, after purification by column chromatography (SiO_2, petroleum ether: acetone = 6:1), a colorless oil of **3b** (1.06 g, 92%) was isolated as a diastereoisomeric mixture, which then crystallized. IR (KBr): 2241 (νCN), 1689 (νC=O), 1613 (νC=N), 1596, 1580, 1515, 1448 (νC=C), 836, 742, 701 (γC-H) cm^{-1}. ^{1}H NMR (600.01 MHz, $CDCl_3$) δ (ppm): *anti*-**3b:** 3.70, 3.78 (2dd, 2H, ^2J = 17.3 Hz, ^3J = 9.1 Hz, ^3J = 4.8 Hz, CH_2CO), 3.75 (s, 3H, CH_3O), 3.97–4.00 (m, 1H, H-3), 4.50 (d, 1H, ^3J = 5.5 Hz, H-2), 6.77-6.81 (m, 2H, Ar), 6.88–6.90 (m, 2H, Ar), 7.10–7.15 (m, 2H, Ar), 7.35–7.38 (m 2H, Ar), 7.40–7.50 (m, 6H, Ar), 7.52–7.58 (m, 1H, Ar), 7.63–7.64 (m, 2H, Ar), 7.94–7.96 (m, 2H, Ar). *syn*-**3b:** 3.59, 3.97 (2dd, 2H, ^2J = 17.1 Hz, ^3J = 8.3 Hz, ^3J = 5.4 Hz, CH_2CO), 3.74 (s, 3H, CH_3O), 3.89 (dt, 1H, ^3J = 8.3 Hz, ^3J = 5.4 Hz, H-3), 4.51 (d, 1H, ^3J = 5.6 Hz, H-2), 6.77–6.81 (m, 2H, Ar), 7.10–7.15 (m, 2H, Ar), 7.16–7.18 (m, 2H, Ar), 7.28–7.31 (m, 2H, Ar), 7.40–7.50 (m, 6H, Ar), 7.52–7.58 (m, 3H, Ar), 7.97–7.98 (m, 2H, Ar). ^{13}C NMR (150.87 MHz, $CDCl_3$) δ (ppm): *anti*-**3b:** 39.93 (C-3), 44.48 (C-4), 55.21 (OCH_3), 58.07 (C-2), 113.91, 118.45 (CN), 127.16, 128.11, 128.29, 128.63, 128.89, 129.34, 129.57, 130.07, 130.91, 131.47, 133.24, 134.88, 136.73, 138.06, 158.88, 174.42 (C=N), 197.50 (C-5). *syn*-**3b:** 39.44 (C-3), 44.27 (C-4), 55.19 (OCH_3), 58.73 (C-2), 114.03, 118.45 (CN), 127.36, 128.18, 128.20, 128.63, 128.99, 129.18, 129.27, 129.49, 130.94, 131.44, 133.26, 134.85, 136.80, 137.94, 158.90, 173.93 (C=N), 197.50 (C-5). ^{1}H NMR and ^{13}C NMR spectra of compound **3b** as an individual diastereoisomer were recorded on a Bruker Avance III HD 500 (^{1}H-500.13 MHz and ^{13}C-125.76 MHz). HRMS (ESI) (individual diastereoisomer): calculated for $C_{31}H_{26}N_2O_2$ [M+H]$^+$ *m/z* 459.2067, found 459.2098.

rel-(2R,3S)-3-(4-(Dimethylamino)phenyl)-2-((diphenylmethylene)amino)-5-oxo-5-phenylpentanenitrile (*anti*-**3c**): Following the general procedure of Method A, after two recrystallizations from acetonitrile, whitish crystals of **3c** (0.89 g, 75%) were obtained as an individual diastereoisomer, mp 178–180 °C. IR (KBr): 2239 (νCN), 1691 (νC=O), 1614 (νC=N), 1596, 1577, 1524, 1447 (νC=C), 815, 761, 700 (γC-H) cm^{-1}. ^{1}H NMR (500.13 MHz, $CDCl_3$) δ (ppm): 2.88 (s, 6H, $N(CH_3)_2$), 3.65, 3.71 (2dd, 2H, ^2J = 17.1 Hz, ^3J = 8.7 Hz, ^3J = 5.4 Hz, CH_2CO), 3.94 (dt, 1H, ^3J = 8.7 Hz, ^3J = 5.4 Hz, H-3), 4.46 (d, 1H, ^3J = 5.5 Hz, H-2), 6.59–6.62 (m, 2H, Ar), 6.88–6.89 (m, 2H, Ar), 7.04–7.07 (m, 2H, Ar), 7.34–7.37 (m, 2H, Ar), 7.38–7.46 (m, 6H, Ar), 7.52–7.56 (m, 1H, Ar), 7.62–7.65 (m, 2H, Ar), 7.93–7.95 (m, 2H, Ar). ^{13}C NMR (125.76 MHz, $CDCl_3$) δ (ppm): 40.11 (C-3), 40.56 ($N(CH_3)_2$), 44.60 (C-4), 58.45 (C-2), 112.59, 118.74 (CN), 126.54, 127.21, 128.14, 128.22, 128.56, 128.82,

129.07, 129.12, 131.19, 133.06, 135.14, 136.92, 138.41, 149.96, 173.87 (C=N), 197.70 (C-5). HRMS (ESI) (individual diastereoisomer): calculated for $C_{32}H_{29}N_3O$ $[M+H]^+$ *m/z* 472.2383, found 472.2395.

2-((Diphenylmethylene)amino)-3-(4-nitrophenyl)-5-oxo-5-phenylpentanenitrile (**3d**): Following the general procedure of Method A, after purification by column chromatography (SiO_2, petroleum ether: acetone = 7:1), a pale-yellow oil of **3d** (1.11 g, 94%) was isolated as a diastereoisomeric mixture, which then crystallized. IR (KBr): 2241 (νCN), 1686 (νC=O), 1615 (νC=N), 1597, 1578, 1492, 1447 (νC=C), 1522, 1347 (νNO_2), 857, 752, 697 (γC-H) cm^{-1}. ^{1}H NMR (500.13 MHz, $CDCl_3$) δ (ppm): *anti*-**3d: 3**.77, 3.88 (2dd, 2H, ^2J = 17.9 Hz, ^3J = 9.0 Hz, ^3J = 4.9 Hz, CH_2CO), 4.05–4.09 (m, 1H, H-3), 4.57 (d, 1H, ^3J = 5.0 Hz, H-2), 6.94–6.95 (m, 2H, Ar), 7.35–7.38 (m, 2H, Ar), 7.43–7.51 (m, 8H, Ar), 7.57–7.61 (m, 3H, Ar), 7.93–7.96 (m, 2H, Ar), 8.14–8.17 (m, 2H, Ar). *syn*-**3d:** 3.63, 3.96 (2dd, 2H, ^2J = 17.5 Hz, ^3J = 8.3 Hz, ^3J = 5.4 Hz, CH_2CO), 4.03 (dt, 1H, ^3J = 8.3 Hz, ^3J = 5.4 Hz, H-3), 4.55 (d, 1H, ^3J = 5.4 Hz, H-2), 7.08–7.10 (m, 2H, Ar), 7.31–7.34 (m, 2H, Ar), 7.43–7.51 (m, 8H, Ar), 7.53–7.55 (m, 2H, Ar), 7.57–7.61 (m, 1H, Ar), 7.93–7.96 (m, 2H, Ar), 8.14–8.17 (m, 2H, Ar). ^{13}C NMR (125.76 MHz, $CDCl_3$) δ (ppm): *anti*-**3d:** 39.64 (C-3), 44.80 (C-4), 57.16 (C-2), 117.81 (CN), 123.62, 126.99, 128.06, 128.42, 128.80, 129.06, 129.11, 129.45, 129.62, 131.71, 133.69, 134.71, 136.28, 137.86, 146.77, 147.31, 174.93 (C=N), 196.51 (C-5). *syn*-**3d:** 39.28 (C-3), 44.83 (C-4), 57.39 (C-2), 117.88 (CN), 123.80, 127.17, 128.12, 128.32, 128.80, 129.06, 129.16, 129.58, 129.71, 131.64, 133.69, 134.75, 136.31, 137.86, 146.65, 147.37, 174.93 (C=N), 196.51 (C-5). HRMS (ESI) (diastereoisomeric mixture): calculated for $C_{30}H_{23}N_3O_3$ $[M+H]^+$ *m/z* 474.1812, found 474.1829.

2-((Diphenylmethylene)amino)-5-(4-methoxyphenyl)-5-oxo-3-phenylpentanenitrile (**3e**): Following the general procedure of Method A, after stirring with 15 mL of cold CH_3OH for 60 min, white crystals of **3e** (1.00 g, 87%) were obtained as an individual diastereoisomer, mp 144–146 °C. Following the general procedure of Method B, after purification by column chromatography (SiO_2, petroleum ether: acetone = 6:1), a colorless oil of **3e** (1.02 g, 89%) was isolated as a diastereoisomeric mixture which then crystallized. IR (KBr): 2242 (νCN), 1672 (νC=O), 1627 (νC=N), 1595, 1574, 1508, 1448 (νC=C), 844, 765, 700 (γC-H) cm^{-1}. ^{1}H NMR (500.13 MHz, $CDCl_3$) δ (ppm): *anti*-**3e:** 3.67, 3.79 (2dd, 2H, ^2J = 17.2 Hz, ^3J = 9.1 Hz, ^3J = 4.9 Hz, CH_2CO), 3.86 (s, 3H, CH_3O), 3.98–4.02 (m, 1H, H-3), 4.51 (d, 1H, ^3J = 5.2 Hz, H-2), 6.81–6.82 (m, 2H, Ar), 6.91–6.94 (m, 2H, Ar), 7.18–7.26 (m, 5H, Ar), 7.34–7.49 (m, 6H, Ar), 7.59–7.62 (m, 2H, Ar), 7.93–7.98 (m, 2H, Ar). *syn*-**3e:** 3.57, 3.93 (2dd, 2H, ^2J = 16.1 Hz, ^3J = 7.3 Hz, ^3J = 5.5 Hz, CH_2CO), 3.87 (s, 3H, CH_3O), 3.90 (dt, 1H, ^3J = 7.5 Hz, ^3J =5.5 Hz, H-3), 4.51 (d, 1H, ^3J = 5.2 Hz, H-2), 6.91–6.94 (m, 2H, Ar), 7.08–7.10 (m, 2H, Ar), 7.18–7.26 (m, 5H, Ar), 7.27–7.30 (m, 2H, Ar), 7.34–7.49 (m, 4H, Ar), 7.50–7.53 (m, 2H, Ar), 7.93–7.98 (m, 2H, Ar). ^{13}C NMR (125.76 MHz, $CDCl_3$) δ (ppm): *anti*-**3e:** 39.28 (C-3), 45.32 (C-4), 55.49 (OCH_3), 58.00 (C-2), 113.76, 118.51 (CN), 127.10, 127.48, 128.25, 128.50, 128.52, 128.85, 129.06, 129.19, 129.91, 130.40, 131.28, 135.00, 138.30, 139.13, 163.58, 174.12 (C=N), 195.81 (C-5). *syn*-**3e:** 38.86 (C-3), 45.15 (C-4), 55.51 (OCH_3), 58.71 (C-2), 113.76, 118.55 (CN), 127.32, 127.54, 128.14, 128.28, 128.63, 128.96, 129.05, 129.41, 129.98, 130.49, 131.21, 135.04, 138.26, 139.20, 163.62, 173.62 (C=N), 195.82 (C-5). HRMS (ESI) (individual diastereoisomer): calculated for $C_{31}H_{26}N_2O_2$ $[M+H]^+$ *m/z* 459.2067, found 459.2096.

3-(4-Chlorophenyl)-2-((diphenylmethylene)amino)-5-(4-methoxyphenyl)-5-oxopentanenitrile (**3f**): Following the general procedure of Method A, after stirring with 15 mL of cold CH_3OH for 60 min, white crystals of **3f** (1.05 g, 85%) were obtained as an individual diastereoisomer, mp 128–130 °C. Following the general procedure of Method B, after purification by column chromatography (SiO_2, petroleum ether: acetone = 10:1), a pale yellow oil of **3f** (1.12 g, 91%) was isolated as a diastereoisomeric mixture, which then crystallized. IR (KBr): 2244 (νCN), 1677 (νC=O), 1622 (νC=N), 1600, 1572, 1492, 1446 (νC=C), 829, 757, 698 (γC-H) cm^{-1}. ^{1}H NMR (500.13 MHz, $CDCl_3$) δ (ppm): *anti*-**3f:** 3.65, 3.74 (2dd, 2H, ^2J = 17.4 Hz, ^3J = 9.1 Hz, ^3J = 5.0 Hz, CH_2CO), 3.87 (s, 3H, CH_3O), 3.93–3.97 (m, 1H, H-3), 4.50 (d, 1H, ^3J = 5.1 Hz, H-2), 6.88–6.94 (m, 4H, Ar), 7.16–7.21 (m, 2H, Ar), 7.22–7.25 (m, 2H, Ar), 7.34–7.38 (m, 2H, Ar), 7.40–7.46 (m, 4H, Ar), 7.60–7.62 (m, 2H, Ar), 7.91–7.96 (m, 2H, Ar). *syn*-**3f:** 3.49–3.55 (m, 1H, CH_2CO), 3.85–3.90 (m, 2H, CH_2CO, H-3), 3.87 (s, 3H, CH_3O), 4.48 (d, 1H, ^3J = 5.1 Hz, H-2), 7.07–7.10 (m, 4H, Ar), 7.16–7.21 (m, 2H, Ar), 7.22–7.25 (m, 2H, Ar), 7.29–7.32 (m, 2H, Ar), 7.40–7.46 (m, 1H, Ar), 7.47–7.50 (m, 3H, Ar), 7.52–7.54 (m, 2H, Ar), 7.91–7.96 (m, 2H, Ar). ^{13}C NMR (125.76 MHz, $CDCl_3$) δ (ppm): *anti*-**3f:** 39.22 (C-3), 44.68 (C-4), 55.51 (OCH_3), 57.73 (C-2), 113.82, 118.27 (CN), 127.07, 128.31, 128.63, 128.95, 129.05, 129.34, 129.71, 129.89, 130.38, 131.44,

133.31, 134.88, 137.70, 138.14, 163.71, 174.38 (C=N), 195.48 (C-5). *syn*-**3f:** 38.87 (C-3), 44.56 (C-4), 55.52 (OCH_3), 58.22 (C-2), 113.82, 118.33 (CN), 127.25, 128.21, 128.78, 129.02, 129.04, 129.50, 129.68, 129.77, 130.46, 131.36, 133.40, 134.93, 137.74, 138.13, 163.75, 174.00 (C=N), 195.50 (C-5). HRMS (ESI) (individual diastereoisomer): calculated for $C_{31}H_{25}ClN_2O_2$ $[M+H]^+$ *m/z* 493.1677, found 493.1713.

2-((Diphenylmethylene)amino)-3-(4-fluorophenyl)-5-oxo-5-(3,4,5-trimethoxyphenyl)pentanenitrile (**3g**): Following the general procedure of Method A, after purification by column chromatography (SiO_2, petroleum ether: acetone = 5:1), a colorless oil of **3g** (1.16 g, 87%) was isolated as a diastereoisomeric mixture, which then crystallized. IR (KBr): 2241 (νCN), 1678 (νC=O), 1615 (νC=N), 1606, 1584, 1510, 1447 (νC=C), 834, 783, 753, 698 (γC-H) cm^{-1}. ^{1}H NMR (500.13 MHz, $CDCl_3$) δ (ppm): *anti*-**3g:** 3.71–3.99 (m, 12H, CH_2CO, H-3, CH_3O), 4.50 (d, 1H, ^3J = 4.9 Hz, H-2), 6.86–7.61 (m, 16H, Ar). *syn*-**3g:** 3.46 (dd, 1H, ^2J = 16.9 Hz, ^3J = 8.4 Hz, CH_2CO), 3.71–3.99 (m, 11H, CH_2CO, H-3, CH_3O), 4.47 (d, 1H, ^3J = 5.3 Hz, H-2), 6.86–7.61 (m, 16H, Ar). ^{13}C NMR (125.76 MHz, $CDCl_3$) δ (ppm): *anti*-**3g:** 39.62 (C-3), 44.94 (C-4), 56.41 (OCH_3), 57.86 (C-2), 60.98 (OCH_3), 105.74, 115.44 (d, ^2J $_{C\text{-}F}$ = 21.0 Hz, C-3′), 118.36 (CN), 127.05, 128.33, 128.95, 129.05, 129.38, 130.06 (d, ^3J $_{C\text{–}F}$ = 8.0 Hz, C-2′), 131.49, 131.98, 134.68, 134.89, 134.91 (d, ^4J $_{C\text{–}F}$ = 3.7 Hz, C-1′), 138.14, 142.94, 153.14, 162.15 (d, ^1J $_{C\text{–}F}$ = 246.4 Hz, C-4′), 174.52 (C=N), 196.12 (C-5). *syn*-**3g:** 39.14 (C-3), 44.84 (C-4), 56.37 (OCH_3), 58.61 (C-2), 60.98 (OCH_3), 105.74, 115.60 (d, ^2J $_{C\text{–}F}$ = 21.1 Hz, C-3′), 118.41 (CN), 127.27, 128.19, 129.03, 129.05, 129.54, 129.80 (d, ^3J $_{C\text{–}F}$ = 8.1 Hz, C-2′), 131.39, 131.86, 134.70, 134.81 (d, ^4J $_{C\text{–}F}$ = 3.2 Hz, C-1′), 134.94, 138.09, 142.88, 153.14, 162.15 (d, ^1J $_{C\text{–}F}$ = 246.4 Hz, C-4′), 173.95 (C=N), 196.05 (C-5). HRMS (ESI) (diastereoisomeric mixture): calculated for $C_{33}H_{29}FN_2O_4$ $[M+H]^+$ *m/z* 537.2184, found 537.2217.

2-((Diphenylmethylene)amino)-5-oxo-5-phenyl-3-(pyridin-3-yl)pentanenitrile (**3h**): Following the general procedure of Method A, after recrystallization from 2-propanol–hexane, white crystals of **3h** (0.72 g, 67%) were obtained as an individual diastereoisomer, mp 140–142 °C. Following the general procedure of Method B, after purification by column chromatography (SiO_2, petroleum ether: acetone = 5:1), a pale yellow oil of **3h** (0.90 g, 84%) was isolated as a diastereoisomeric mixture, which then crystallized. IR (KBr): 2237 (νCN), 1687 (νC=O), 1624 (νC=N), 1594, 1575, 1489, 1446 (νC=C), 1000 (νC-N), 759, 702 (γC-H) cm^{-1}. ^{1}H NMR (500.13 MHz, $CDCl_3$) δ (ppm): *anti*-**3h:** 3.77, 3.85 (2dd, 2H, ^2J = 17.8 Hz, ^3J = 8.6 Hz, ^3J = 5.3 Hz, CH_2CO), 3.97–4.01 (m, 1H, H-3), 4.56 (d, 1H, ^3J = 4.9 Hz, H-2), 6.92–6.93 (m, 2H, Ar), 7.22 (dd, 1H, ^3J = 7.8 Hz, ^3J = 4.8 Hz, pyridine H-5′), 7.34–7.37 (m, 2H, Ar), 7.43–7.48 (m, 6H, Ar), 7.56–7.62 (m, 3H, Ar), 7.64 (dt, 1H, ^3J = 7.9 Hz, ^4J = 1.9 Hz, pyridine H-4′), 7.93–7.96 (m, 2H, Ar), 8.50 (dd, 1H, ^3J = 4.8 Hz, ^4J = 1.5 Hz, pyridine H-6′), 8.53 (d, 1H, ^4J = 1.9 Hz, pyridine H-2′). *syn*-**3h:** 3.55–3.61 (m, 1H, CH_2CO), 3.93–4.01 (m, 2H, CH_2CO, H-3), 4.54 (d, 1H, ^3J = 5.1 Hz, H-2), 7.08–7.10 (m, 2H, Ar), 7.24 (dd, 1H, ^3J = 7.7 Hz, ^3J = 4.8 Hz, pyridine H-5′), 7.29–7.32 (m, 2H, Ar), 7.41–7.50 (m, 6H, Ar), 7.53–7.62 (m, 3H, Ar), 7.69 (dt, 1H, ^3J = 7.9 Hz, ^4J = 1.9 Hz, pyridine H-4′), 7.93–7.96 (m, 2H, Ar), 8.48–8.53 (m, 2H, pyridine H-2′, H-6′). ^{13}C NMR (125.76 MHz, $CDCl_3$) δ (ppm): *anti*-**3h:** 39.41 (C-3), 42.71 (C-4), 57.35 (C-2), 117.99 (CN), 123.34, 126.98, 128.06, 128.36, 128.74, 129.05, 129.09, 129.46, 131.57, 133.54, 134.76, 134.85, 135.98, 136.45, 137.97, 148.92, 150.25, 174.78 (C=N), 196.72 (C-5). *syn*-**3h:** 39.04 (C-3), 42.75 (C-4), 57.69 (C-2), 118.04 (CN), 123.46, 127.15, 128.14, 128.26, 128.74, 129.06, 129.13, 129.64, 131.50, 133.55, 134.75, 134.86, 135.84, 136.48, 137.97, 149.02, 150.14, 174.54 (C=N), 196.71 (C-5). HRMS (ESI) (individual diastereoisomer): calculated for $C_{29}H_{23}N_3O$ $[M+H]^+$ *m/z* 430.1914, found 430.1932.

2-((Diphenylmethylene)amino)-3-(4-methylphenyl)-5-oxo-5-phenylpentanenitrile (**3i**): Following the general procedure of Method B, after purification by column chromatography (SiO_2, petroleum ether: acetone = 20:1), a pale-yellow oil of **3i** (1.01 g, 91%) was isolated as a diastereoisomeric mixture, which then crystallized. IR (KBr): 2240 (νCN), 1685 (νC=O), 1617 (νC=N), 1596, 1578, 1515, 1447 (νC=C), 814, 763, 697 (γC-H) cm^{-1}. ^{1}H NMR (500.13 MHz, $CDCl_3$) δ (ppm): *anti*-**3i:** 2.28 (s, 3H, CH_3), 3.69, 3.78 (2dd, 2H, ^2J = 17.3 Hz, ^3J = 9.0 Hz, ^3J = 4.9 Hz, CH_2CO), 3.95–4.01 (m, 1H, H-3), 4.48 (d, 1H, ^3J = 5.3 Hz, H-2), 6.84–6.86 (m, 2H, Ar), 7.04–7.14 (m, 5H, Ar), 7.34–7.37 (m, 2H, Ar), 7.38–7.48 (m, 4H, Ar), 7.54–7.58 (m, 2H, Ar), 7.61–7.63 (m, 2H, Ar), 7.93–7.96 (m, 2H, Ar). *syn*-**3i:** 2.27 (s, 3H, CH_3), 3.62, 4.00 (2dd, 2H, ^2J = 17.2 Hz, ^3J = 8.3 Hz, ^3J = 5.3 Hz, CH_2CO), 3.85 (dt, 1H, ^3J = 8.3 Hz, ^3J = 5.3 Hz, H-3), 4.48 (d, 1H, ^3J = 5.3 Hz, H-2), 7.04–7.14 (m, 5H, Ar), 7.27–7.30 (m, 2H, Ar), 7.38–7.48 (m, 8H, Ar), 7.51–7.54 (m, 2H, Ar), 7.98–8.00 (m, 2H, Ar). ^{13}C NMR (125.76 MHz, $CDCl_3$) δ (ppm): *anti*-**3i:** 21.07 (CH_3), 39.83 (C-3), 44.92 (C-4), 58.11 (C-2), 118.54 (CN), 127.14,

128.11, 128.25, 128.32, 128.62, 128.85, 129.07, 129.20, 129.39, 131.28, 133.19, 135.03, 135.91, 136.81, 137.13, 138.30, 174.11 (C=N), 197.41 (C-5). *syn*-**3i:** 21.04 (CH_3), 39.25 (C-3), 44.71 (C-4), 58.88 (C-2), 118.54 (CN), 127.29, 128.07, 128.14, 128.22, 128.62, 128.97, 129.05, 129.22, 129.36, 131.21, 133.21, 135.07, 136.01, 136.89, 137.24, 138.24, 173.56 (C=N), 197.47 (C-5). HRMS (ESI) (diastereoisomeric mixture): calculated for $C_{31}H_{26}N_2O$ $[M+H]^+$ *m/z* 443.2118, found 443.2121.

3-(4-Chlorophenyl)-2-((diphenylmethylene)amino)-5-oxo-5-phenylpentanenitrile (**3j**): Following the general procedure of Method B, after purification by column chromatography (SiO_2, petroleum ether: ethyl acetate = 15:1), a pale-yellow oil of **3j** (1.10 g, 95%) was isolated as a diastereoisomeric mixture, which then crystallized. IR (KBr): 2241 (νCN), 1686 (νC=O), 1617 (νC=N), 1596, 1578, 1493, 1448 (νC=C), 829, 755, 696 (γC-H) cm^{-1}. ^1H NMR (500.13 MHz, $CDCl_3$) δ (ppm): *anti*-**3j:** 3.72, 3.81 (2dd, 2H, ^2J = 17.6 Hz, ^3J = 9.1 Hz, ^3J = 4.9 Hz, CH_2CO), 3.93–3.99 (m, 1H, H-3), 4.50 (d, 1H, ^3J = 5.3 Hz, H-2), 6.88–6.89 (m, 2H, Ar), 7.05–7.11 (m, 2H, Ar), 7.16–7.21 (m, 2H, Ar), 7.22–7.26 (m, 1H, Ar), 7.35–7.38 (m, 2H, Ar), 7.40–7.50 (m, 4H, Ar), 7.55–7.59 (m, 2H, Ar), 7.60–7.62 (m, 2H, Ar), 7.93–7.97 (m, 2H, Ar). *syn*-**3j:** 3.58, 3.95 (2dd, 2H, ^2J = 17.2 Hz, ^3J = 8.2 Hz, ^3J = 5.4 Hz, CH_2CO), 3.88 (dt, 1H, ^3J = 8.2 Hz, ^3J = 5.4 Hz, H-3), 4.48 (d, 1H, ^3J = 5.4 Hz, H-2), 7.08–7.10 (m, 2H, Ar), 7.16–7.21 (m, 2H, Ar), 7.22–7.26 (m, 2H, Ar), 7.29–7.32 (m, 2H, Ar), 7.40–7.50 (m, 7H, Ar) , 7.51–7.54 (m, 2H, Ar), 7.93–7.97 (m, 2H, Ar). ^{13}C NMR (125.76 MHz, $CDCl_3$) δ (ppm): *anti*-**3j:** 39.64 (C-3), 44.55 (C-4), 57.68 (C-2), 118.22 (CN), 127.05, 128.08, 128.33, 128.67, 128.97, 129.06, 129.36, 129.88, 131.47, 133.39, 133.48, 134.87, 136.60, 137.60, 138.10, 174.48 (C=N), 197.03 (C-5). *syn*-**3j:** 39.30 (C-3), 44.47 (C-4), 58.24 (C-2), 118.26 (CN), 127.24, 128.16, 128.21, 128.70, 128.82, 129.04, 129.53, 129.66, 131.39, 133.41, 133.42, 134.92, 136.64, 137.60, 138.08, 174.05 (C=N), 197.03 (C-5). HRMS (ESI) (diastereoisomeric mixture): calculated for $C_{30}H_{23}ClN_2O$ $[M+H]^+$ *m/z* 463.1572, found 463.1575.

2-((Diphenylmethylene)amino)-5-(4-methylphenyl)-5-oxo-3-phenylpentanenitrile (**3k**): Following the general procedure of Method B, after purification by column chromatography (SiO_2, petroleum ether: acetone = 20:1), a colorless oil of **3k** (0.94 g, 85%) was isolated as a diastereoisomeric mixture, which then crystallized. IR (KBr): 2239 (νCN), 1682 (νC=O), 1622 (νC=N), 1608, 1574, 1493, 1446 (νC=C), 817, 760, 700 (γC-H) cm^{-1}. ^1H NMR (500.13 MHz, $CDCl_3$) δ (ppm): *anti*-**3k:** 2.40 (s, 3H, CH_3), 3.70, 3.81 (2dd, 2H, ^2J = 17.4 Hz, ^3J = 9.1 Hz, ^3J = 4.9 Hz, CH_2CO), 3.99–4.02 (m, 1H, H-3), 4.51 (d, 1H, ^3J = 4.9 Hz, H- 2), 6.81–6.82 (m, 2H, Ar), 7.18–7.26 (m, 7H, Ar), 7.34–7.37 (m, 2H, Ar), 7.37–7.48 (m, 4H, Ar), 7.59–7.62 (m, 2H, Ar), 7.84–7.89 (m, 2H, Ar). *syn*-**3k:** 2.41 (s, 3H, CH_3), 3.60, 3.96 (2dd, 2H, ^2J = 16.8 Hz, ^3J = 8.0 Hz, ^3J = 5.4 Hz, CH_2CO), 3.90 (dt, 1H, ^3J = 8.0 Hz, ^3J = 5.4 Hz, H-3), 4.51 (d, 1H, ^3J = 5.4 Hz, H-2), 7.08–7.09 (m, 2H, Ar), 7.18–7.26 (m, 7H, Ar), 7.27–7.30 (m, 2H, Ar), 7.37–7.48 (m, 4H, Ar), 7.50–7.53 (m, 2H, Ar), 7.84–7.89 (m, 2H, Ar). ^{13}C NMR (125.76 MHz, $CDCl_3$) δ (ppm): *anti*-**3k:** 21.66 (CH_3), 39.54 (C-3), 45.24 (C- 4), 57.98 (C-2), 118.49 (CN), 127.10, 127.49, 128.25, 128.28, 128.52, 128.85, 129.06, 129.20, 129.30, 131.29, 134.34, 135.00, 138.25, 139.09, 144.05, 174.15 (C=N), 196.94 (C-5). *syn*-**3k:** 21.66 (CH_3), 39.14 (C-3), 45.07 (C-4), 58.68 (C-2), 118.52 (CN), 127.32, 127.56, 128.14, 128.23, 128.32, 128.63, 128.96, 129.05, 129.41, 131.22, 134.40, 135.05, 138.28, 139.15, 144.09, 173.65 (C=N), 196.94 (C-5). HRMS (ESI) (diastereoisomeric mixture): calculated for $C_{31}H_{26}N_2O$ $[M+H]^+$ *m/z* 443.2118, found 443.2137.

5-(4-Chlorophenyl)-2-((diphenylmethylene)amino)-5-oxo-3-phenylpentanenitrile (**3l**): Following the general procedure of Method B, after purification by column chromatography (SiO_2, petroleum ether: acetone = 15:1), a pale-yellow oil of **3l** (0.97 g, 84%) was isolated as a diastereoisomeric mixture, which then crystallized. IR (KBr): 2240 (νCN), 1687 (νC=O), 1618 (νC=N), 1591, 1572, 1489, 1446 (νC=C), 833, 763, 700 (γC-H) cm^{-1}. ^1H NMR (500.13 MHz, $CDCl_3$) δ (ppm): *anti*-**3l:** 3.72, 3.82 (2dd, 2H, ^2J = 17.4 Hz, ^3J = 9.0 Hz, ^3J = 4.9 Hz, CH_2CO), 3.97–4.01 (m, 1H, H-3), 4.50 (d, 1H, J = 5.1 Hz, H-2), 6.78–6.79 (m, 2H, Ar), 7.17–7.30 (m, 6H, Ar), 7.34–7.46 (m, 5H, Ar), 7.46–7.50 (m, 2H, Ar), 7.59–7.62 (m, 2H, Ar), 7.88–7.91 (m, 2H, Ar). *syn*-**3l:** 3.57, 3.99 (2dd, 2H, ^2J = 17.1 Hz, ^3J = 8.0 Hz, ^3J = 5.5 Hz, CH_2CO), 3.89 (dt, 1H, ^3J = 8.0 Hz,^3J = 5.5 Hz, H-3), 4.49 (d, 1H, J = 5.5 Hz, H-2), 7.09–7.11 (m, 2H, Ar), 7.17–7.30 (m, 6H, Ar), 7.34–7.46 (m, 5H, Ar), 7.46–7.50 (m, 4H, Ar), 7.91–7.93 (m, 2H, Ar). ^{13}C NMR (125.76 MHz, $CDCl_3$) δ (ppm): *anti*-**3l:** 39.62 (C-3), 45.19 (C-4), 57.85 (C-2), 118.40 (CN), 127.04, 127.62, 128.28, 128.59, 128.86, 129.00, 129.05, 129.23, 129.47, 129.57, 131.37, 134.94, 135.09, 138.19, 138.87, 139.72, 174.35 (C=N), 196.18 (C-5). *syn*-**3l:** 39.22 (C-3), 45.14 (C-4), 58.80 (C-2), 118.40 (CN), 127.27, 127.70, 128.15, 128.17, 128.44, 128.72, 128.96, 129.03, 129.53, 129.64, 131.30, 134.98,

135.17, 138.14, 138.87, 139.74, 173.74 (C=N), 196.20 (C-5). HRMS (ESI) (diastereoisomeric mixture): calculated for $C_{30}H_{23}ClN_2O$ [M+H]$^+$ *m/z* 463.1572, found 463.1569.

2-((Diphenylmethylene)amino)-3-(3-fluoro-4-methylphenyl)-5-(2-hydroxy-4,6-dimethoxyphenyl)-5-oxopentanenitrile (**3m**): Following the general procedure of Method C, after purification by column chromatography (SiO_2, petroleum ether: acetone = 10:1), a colorless oil of **3m** (0.473 g, 88%) was isolated as a diastereoisomeric mixture, which then crystallized. IR (KBr): 3434 (νOH), 2241 (νCN), 1620 (νC=O), 1620 (νC=N), 1595, 1578, 1510, 1444 (νC=C), 823, 783, 735, 698 (γC-H) cm^{-1}. ^{1}H NMR (500.13 MHz, $CDCl_3$) δ (ppm): *anti*-**3m:** 2.23 (s, 3H, CH_3), 3.65, 3.73 (2dd, 2H, ^2J = 17.5 Hz, ^3J = 8.4 Hz, ^3J = 5.7 Hz, CH_2CO), 3.80 (s, 3H, CH_3O), 3.78-3.86 (m, 1H, H-3), 3.90 (s, 3H, CH_3O), 4.49 (d, 1H, ^3J = 5.4 Hz, H-2), 5.93 (d, 1H, ^4J = 2.1 Hz, Ar), 6.01 (d, 1H, ^4J = 2.1 Hz, Ar), 6.87–6.98 (m, 3H, Ar), 7.05–7.10 (m, 1H, Ar), 7.31–7.36 (m, 1H, Ar), 7.41–7.49 (m, 6H, Ar), 7.60–7.61 (m, 2H, Ar), 13.57 (s, 1H, OH). *syn*-**3m:** 2.21 (s, 3H, CH_3), 3.59, 3.90 (2dd, 2H, ^2J = 17.4 Hz, ^3J = 8.6 Hz, ^3J = 5.3 Hz, CH_2CO), 3.78–3.86 (m, 1H, H-3), 3.81 (s, 3H, CH_3O), 3.90 (s, 3H, CH_3O), 4.47 (d, 1H, ^3J = 5.7 Hz, H-2), 5.94 (d, 1H, ^4J = 2.1 Hz, Ar), 6.02 (d, 1H, ^4J = 2.1 Hz, Ar), 6.87–6.98 (m, 3H, Ar), 7.05–7.10 (m, 3H, Ar), 7.31–7.36 (m, 3H, Ar), 7.41–7.49 (m, 2H, Ar), 7.54–7.56 (m, 2H, Ar), 13.57 (s, 1H, OH). ^{13}C NMR (125.76 MHz, $CDCl_3$) δ (ppm): *anti*-**3m:** 14.25 (CH_3), 44.76 (C-3), 45.22 (C-4), 55.58 (OCH_3), 55.76 (OCH_3), 58.05 (C-2), 90.96, 93.72, 105.80, 115.11 (d, $^2J_{C–F}$ = 22.7 Hz, C-2′), 118.41 (CN), 123.81 (d, $^2J_{C–F}$ = 16.8 Hz, C-4′), 124.04 (d, $^4J_{C–F}$ = 3.0 Hz, C-6′), 127.18, 128.25, 128.89, 129.06, 129.31, 131.36 (d, $^3J_{C–F}$ = 5.7 Hz, C-5′), 135.04, 138.31, 139.09 (d, $^3J_{C–F}$ = 7.0 Hz, C-1′), 161.08 (d, $^1J_{C–F}$ = 244.5 Hz, C-3′), 162.55, 166.18, 167.63, 173.85 (C=N), 201.83 (C-5). *syn*-**3m:** 14.27 (CH_3), 44.64 (C-3), 44.88 (C-4), 55.58 (OCH_3), 55.82 (OCH_3), 58.20 (C-2), 90.96, 93.69, 105.82, 114.88 (d, $^2J_{C–F}$ = 22.7 Hz, C-2′), 118.46 (CN), 123.84 (d, $^2J_{C–F}$ = 17.1 Hz, C-4′), 123.95 (d, $^4J_{C–F}$ = 3.2 Hz, C-6′), 127.32, 128.20, 128.98, 129.04, 129.44, 131.28 (d, $^3J_{C–F}$ = 6.1 Hz, C-5′), 135.07, 138.35, 139.12 (d, $^3J_{C–F}$ = 6.9 Hz, C-1′), 161.11 (d, $^1J_{C–F}$ = 244.7 Hz, C-3′), 162.70, 166.20, 167.61, 173.82 (C=N), 201.78 (C-5). HRMS (ESI) (diastereoisomeric mixture): calculated for $C_{33}H_{29}FN_2O_4$ [M+H]$^+$ *m/z* 537.2184, found 537.2180.

3-(3-Chloro-4-methylphenyl)-2-((diphenylmethylene)amino)-5-(2-hydroxy-4,6-dimethoxyphenyl)-5-oxopentanenitrile (**3n**): Following the general procedure of Method C, after purification by column chromatography (SiO_2, petroleum ether: acetone = 10:1), a colorless oil of **3n** (0.503 g, 91%) was isolated as a diastereoisomeric mixture, which then crystallized. IR (KBr): 3430 (νOH), 2241 (νCN), 1620 (νC=O), 1620 (νC=N), 1596, 1580, 1496, 1443 (νC=C), 822, 783, 736, 698 (γC-H) cm^{-1}. ^{1}H NMR (500.13 MHz, $CDCl_3$) δ (ppm): *anti*-**3n:** 2.33 (s, 3H, CH_3), 3.66, 3.75 (2dd, 2H, ^2J = 17.3 Hz, ^3J = 8.4 Hz, ^3J = 5.5 Hz, CH_2CO), 3.79–3.83 (m, 1H, H-3), 3.80 (s, 3H, OCH_3), 3.90 (s, 3H, OCH_3), 4.49 (d, 1H, ^3J = 5.2 Hz, H-2), 5.93 (d, 1H, ^4J = 2.4 Hz, Ar), 6.01 (d, 1H, ^4J = 2.4 Hz, Ar), 6.94–6.96 (m, 2H, Ar), 7.03–7.08 (m, 2H, Ar), 7.11–7.14 (m, 1H, Ar), 7.20 (d, 1H, ^4J = 1.7 Hz, Ar), 7.30–7.37 (m, 2H, Ar), 7.40–7.46 (m, 3H, Ar), 7.60–7.62 (m, 2H, Ar), 13.57 (s, 1H, OH). *syn*-**3n:** 2.30 (s, 3H, CH_3), 3.56, 3.87 (2dd, 2H, ^2J = 17.2 Hz, ^3J = 8.6 Hz, ^3J = 5.1 Hz, CH_2CO), 3.79–3.83 (m, 1H, H-3), 3.81 (s, 3H, OCH_3), 3.90 (s, 3H, OCH_3), 4.46 (d, 1H, ^3J = 6.0 Hz, H-2), 5.94 (d, 1H, ^4J = 2.4 Hz, Ar), 6.02 (d, 1H, ^4J = 2.4 Hz, Ar), 7.03–7.08 (m, 2H, Ar), 7.11–7.14 (m, 1H, Ar), 7.15 (d, 1H, ^4J = 1.7 Hz, Ar), 7.30–7.37 (m, 2H, Ar), 7.40–7.46 (m, 2H, Ar), 7.47–7.50 (m, 3H, Ar), 7.53–7.56 (m, 2H, Ar), 13.56 (s, 1H, OH). ^{13}C NMR (125.76 MHz, $CDCl_3$) δ (ppm): *anti*-**3n:** 19.69 (CH_3), 44.64 (C-3), 45.01 (C-4), 55.58 (OCH_3), 55.78 (OCH_3), 57.97 (C-2), 90.95, 93.72, 105.78, 118.36 (CN), 126.78, 127.16, 128.24, 128.81, 128.99, 129.06, 129.32, 130.81, 131.30, 134.19, 135.00, 135.06, 138.29, 138.65, 162.55, 166.20, 167.63, 173.87 (C=N), 201.77 (C-5). *syn*-**3n:** 19.69 (CH_3), 44.64 (C-3), 44.96 (C-4), 55.58 (OCH_3), 55.83 (OCH_3), 58.10 (C-2), 90.95, 93.68, 105.80, 118.44 (CN), 127.02, 127.33, 128.19, 128.89, 129.04, 129.30, 129.44, 130.87, 131.25, 134.26, 135.06, 135.06, 138.33, 138.71, 162.67, 166.21, 167.60, 173.85 (C=N), 201.64 (C-5). HRMS (ESI) (diastereoisomeric mixture): calculated for $C_{33}H_{29}ClN_2O_4$ [M+H]$^+$ *m/z* 553.1889, found 553.1913.

3-(3-Bromo-4-methylphenyl)-2-((diphenylmethylene)amino)-5-(2-hydroxy-4,6-dimethoxyphenyl)-5-oxopentanenitrile (**3o**): Following the general procedure of Method C, after purification by column chromatography (SiO_2, petroleum ether: acetone = 10:1), a colorless oil of **3o** (0.538 g, 90%) was isolated as a diastereoisomeric mixture, which then crystallized. IR (KBr): 3434 (νOH), 2242 (νCN), 1620 (νC=O), 1620 (νC=N), 1596, 1581, 1492, 1443(νC=C), 822, 783, 735, 698 (γC-H) cm^{-1}. ^{1}H NMR (500.13 MHz, $CDCl_3$) δ (ppm): *anti*-**3o:** 2.35 (s, 3H, CH_3), 3.67 (dd, 1H, ^2J = 16.8 Hz, ^3J = 5.0 Hz, CH_2CO), 3.78–3.83 (m, 2H, CH_2CO,

H-3), 3.80 (s, 3H, OCH_3), 3.90 (s, 3H, OCH_3), 4.49 (d, 1H, ^3J = 4.9 Hz, H-2), 5.93 (d, 1H, ^4J = 2.4 Hz, Ar), 6.01 (d, 1H, ^4J = 2.4 Hz, Ar), 6.93–6.95 (m, 2H, Ar), 7.10–7.15 (m, 2H, Ar), 7.30–7.37 (m, 3H, Ar), 7.42–7.46 (m, 1H, Ar), 7.47–7.50 (m, 3H, Ar), 7.59–7.62 (m, 2H, Ar), 13.57 (s, 1H, OH). *syn*-**3o:** 2.32 (s, 3H, CH_3), 3.55, 3.86 (2dd, 2H, ^2J = 16.9 Hz, ^3J = 8.4 Hz, ^3J = 5.1 Hz, CH_2CO), 3.78–3.83 (m, 1H, H-3), 3.81 (s, 3H, OCH_3), 3.90 (s, 3H, OCH_3), 4.45 (d, 1H, ^3J = 5.9 Hz, H-2), 5.94 (d, 1H, ^4J = 2.4 Hz, Ar), 6.02 (d, 1H, ^4J = 2.4 Hz, Ar), 7.03–7.05 (m, 2H, Ar), 7.10–7.15 (m, 2H, Ar), 7.30–7.37 (m, 2H, Ar), 7.39–7.41 (m, 1H, Ar), 7.42–7.46 (m, 3H, Ar), 7.47–7.50 (m, 1H, Ar), 7.54–7.56 (m, 2H, Ar), 13.56 (s, 1H, OH). ^{13}C NMR (125.76 MHz, $CDCl_3$) δ (ppm): *anti*-**3o:** 22.54 (CH_3), 44.55 (C-3), 44.93 (C-4), 55.58 (OCH_3), 55.78 (OCH_3), 57.95 (C-2), 90.96, 93.72, 105.79, 118.34 (CN), 124.76, 127.37, 127.73, 128.25, 128.90, 129.08, 129.32, 130.63, 131.30, 132.65, 134.99, 136.91, 138.29, 138.82, 162.55, 166.22, 167.63, 173.87 (C=N), 201.76 (C-5). *syn*-**3o:** 22.52 (CH_3), 44.58 (C-3), 44.99 (C-4), 55.58, 55.83 (OCH_3), 58.07 (C-2), 90.96, 93.69, 105.79, 118.44 (CN), 124.84, 127.15, 127.33, 128.20, 129.01, 129.05, 129.44, 130.70, 131.25, 132.06, 135.06, 136.91, 138.33, 138.90, 162.66, 166.22, 167.61, 173.87 (C=N), 201.60 (C-5). HRMS (ESI) (diastereoisomeric mixture): calculated for $C_{33}H_{29}BrN_2O_4$ $[M+H]^+$ *m/z* 597.1383, found 597.1401.

3,5-Diaryl-3,4-dihydro-2H-pyrrole-2-carbonitriles (Δ¹-Pyrroline-5-carbonitriles)

rel-(2R,3S)-3,5-Diphenyl-3,4-dihydro-2H-pyrrole-2-carbonitrile (*trans*-**4a**): Following the general procedure of Method A and Work up (A), after purification by column chromatography (SiO_2, petroleum ether: acetone = 15:1), a colorless oil of *trans*-**4a** (0.237 g, 96%) was obtained as an individual diastereoisomer. Following the general procedure of Method B, after purification by column chromatography (SiO_2, petroleum ether: acetone with variable polarity), a colorless oil of *trans*-**4a** (0.096 g, 39%) was obtained as an individual diastereoisomer.

rel-(2R,3R)-3,5-Diphenyl-3,4-dihydro-2H-pyrrole-2-carbonitrile (*cis*-**4a**): Following the general procedure of Method B, after purification by column chromatography (SiO_2, petroleum ether: acetone with variable polarity), white crystals of *cis*-**4*a*** (0.113 g, 46%) as an individual diastereoisomer was obtained, mp 107–109 °C. IR (KBr): 2244 (νCN), 1608 (νC=N), 1602, 1574, 1496, 1450 (νC=C), 764, 696 (γC-H) cm^{-1}. ^{1}H NMR (250.13 MHz, $CDCl_3$) δ (ppm): 3.42 (ddd, 1H, ^2J = 17.3 Hz, ^3J = 6.6 Hz, ^4J = 1.5 Hz, H-4a), 3.52 (ddd, 1H, ^2J = 17.3 Hz, ^3J = 8.3 Hz, ^4J = 1.5 Hz, H-4b), 3.95 (td, 1H, ^3J = 8.3 Hz, ^3J = 6.7, Hz, H-3), 5.32 (dt, 1H, ^3J = 8.3 Hz, ^4J = 1.5 Hz, H-2), 7.22–7.29 (m, 2H, Ar), 7.30-7.40 (m, 3H, Ar), 7.44–7.58 (m, 3H, Ar), 7.91–7.96 (m, 2H, Ar). ^{13}C NMR (62.90 MHz, $CDCl_3$) δ (ppm): 42.43 (C-4), 46.18 (C-3), 67.37 (C-2), 117.01 (CN), 127.52, 128.06, 128.21, 128.79, 128.97, 131.96, 132.81, 138.69, 177.76 (C-5). HRMS (ESI): calculated for $C_{17}H_{14}N_2$ $[M+H]^+$ *m/z* 247.1230, found 247.1246.

rel-(2R,3S)-3-(4-Methoxyphenyl)-5-phenyl-3,4-dihydro-2H-pyrrole-2-carbonitrile (*trans*-**4b**): Following the general procedure of Method A and Work up (A), after purification by column chromatography (SiO_2, petroleum ether: acetone = 8:1), a pale-yellow oil of *trans*-**4b** (0.252 g, 91%) was obtained as an individual diastereoisomer. Following the general procedure of Method B, after purification by column chromatography (SiO_2, petroleum ether: acetone with variable polarity), a pale-yellow oil of *trans*-**4b** (0.083 g, 30%) was obtained as an individual diastereoisomer. IR (KBr): 2243 (νCN), 1609 (νC=N), 1575, 1514, 1449 (νC=C), 827, 763, 692 (γC-H) cm^{-1}. ^{1}H NMR (500.13 MHz, $CDCl_3$) δ (ppm): 3.21 (ddd, 1H, ^2J = 17.4 Hz, ^3J = 7.6 Hz, ^4J = 1.7 Hz, H-4a), 3.65 (ddd, 1H, ^2J = 17.4 Hz, ^3J = 9.4 Hz, ^4J = 1.7 Hz, H-4b), 3.81 (s, 3H, CH_3O), 3.90 (dt, 1H, ^3J = 9.3 Hz, ^3J = 7.4 Hz, H-3), 4.87 (dt, 1H, ^3J = 7.1 Hz, ^4J = 1.7 Hz, H-2), 6.88–6.91 (m, 2H, Ar), 7.18–7.21 (m, 2H, Ar), 7.44–7.48 (m, 2H, Ar), 7.51–7.54 (m, 1H, Ar), 7.89–7.91 (m, 2H, Ar). ^{13}C NMR (125.76 MHz, $CDCl_3$) δ (ppm): 43.74 (C-4), 48.29 (C-3), 55.37 (CH_3O), 69.14 (C-2), 114.58, 119.33 (CN), 127.82, 128.17, 128.76, 131.92, 132.03, 132.78, 159.17, 176.82 (C-5). HRMS (ESI): calculated for $C_{18}H_{16}N_2O$ $[M+H]^+$ *m/z* 277.1335, found 277.1352.

rel-(2R,3R)-3-(4-Methoxyphenyl)-5-phenyl-3,4-dihydro-2H-pyrrole-2-carbonitrile (*cis*-**4b**): Following the general procedure of Method B, after purification by column chromatography (SiO_2, petroleum ether: acetone with variable polarity), white crystals of *cis*-**4b** (0.119 g, 43%) were obtained as an individual diastereoisomer, mp 137–139 °C. IR (KBr): 2247 (νCN), 1607 (νC=N), 1575, 1514, 1452 (νC=C), 797, 767, 693 (γC-H) cm^{-1}. ^{1}H NMR (500.13 MHz, $CDCl_3$) δ (ppm): 3.38 (ddd, 1H, ^2J = 17.2 Hz, ^3J = 6.3 Hz, ^4J = 1.4 Hz, H-

4a), 3.49 (ddd, 1H, ^2J = 17.2 Hz, ^3J = 8.7 Hz, ^4J = 1.4 Hz, H-4b), 3.80 (s, 3H, CH_3O), 3.92 (td, 1H, ^3J = 8.4 Hz, ^3J = 6.2 Hz, H-3), 5.29 (dt, 1H, ^3J = 8.2 Hz, ^4J = 1.4 Hz, H-2), 6.87–6.90 (m, 2H, Ar), 7.16–7.19 (m, 2H, Ar), 7.46–7.50 (m, 2H, Ar), 7.52–7.56 (m, 1H, Ar), 7.91–7.94 (m, 2H, Ar). ^{13}C NMR (125.76 MHz, $CDCl_3$) δ (ppm): 42.55 (C-4), 45.50 (C-3), 55.26 (CH_3O), 67.41 (C-2), 114.32, 117.09 (CN), 128.25, 128.62, 128.80, 130.58, 132.02, 132.76, 159.27, 177.91 (C-5). HRMS (ESI): calculated for $C_{18}H_{16}N_2O$ [M+H]$^+$ *m/z* 277.1335, found 277. 1330.

rel-(2R,3S)-3-(4-(Dimethylamino)phenyl)-5-phenyl-3,4-dihydro-2H-pyrrole-2-carbonitrile (*trans*-**4c**): Following the general procedure of Method A (in diethyl ether, without the addition of methanol) and Work up (B), after purification by column chromatography (SiO_2, petroleum ether: acetone = 10:1), white crystals of *trans*-**4c** (0.217 g, 75%) were obtained as an individual diastereoisomer, mp 119–121 °C. IR (KBr): 2243 (νCN), 1613 (νC=N), 1574, 1524, 1447 (νC=C), 827, 763, 692 (γC-H) cm^{-1}. ^{1}H NMR (500.13 MHz, $CDCl_3$) δ (ppm): 2.94 (s, 6H, $(CH_3)_2N$), 3.21 (ddd, 1H, ^2J = 17.4 Hz, ^3J = 7.7 Hz, ^4J = 1.7 Hz, H-4a), 3.62 (ddd, 1H, ^2J = 17.4 Hz, ^3J = 9.4 Hz, ^4J = 1.7 Hz, H-4b), 3.86 (dt, 1H, ^3J = 9.3 Hz, ^3J = 7.5 Hz, H-3), 4.86 (dt, 1H, ^3J = 7.2 Hz, ^4J = 1.7 Hz, H-2), 6.69–6.72 (m, 2H, Ar), 7.12–7.15 (m, 2H, Ar), 7.44–7.47 (m, 2H, Ar), 7.50–7.54 (m, 1H, Ar), 7.88–7.91 (m, 2H, Ar). ^{13}C NMR (125.76 MHz, $CDCl_3$) δ (ppm): 40.55 ($(CH_3)_2N$), 43.67 (C-4), 48.29 (C-3), 69.18 (C-2), 112.95, 119.55 (CN), 127.41, 127.46, 128.15, 128.72, 131.80, 132.94, 150.14, 176.91 (C-5). HRMS (ESI): calculated for $C_{19}H_{19}N_3$ [M+H]$^+$ *m/z* 290.1652, found 290.1649.

3-(4-Nitrophenyl)-5-phenyl-3,4-dihydro-2H-pyrrole-2-carbonitrile (**4d**): Following the general procedure of Method A and Work up (A), after purification by column chromatography (SiO_2, petroleum ether: acetone = 10:1), pale yellow crystals of **4d** (0.251 g, 86%) were obtained as a diastereoisomeric mixture, mp 139–145 °C. IR (KBr): 2246 (νCN), 1607 (νC=N), 1575, 1515, 1448 (νC=C), 1515, 1343 (νNO_2), 851, 770, 694 (γC-H) cm^{-1}. ^{1}H NMR (500.13 MHz, $CDCl_3$) δ (ppm): *trans*-**4d:** 3.28 (ddd, 1H, ^2J = 17.4 Hz, ^3J = 7.3 Hz, ^4J = 1.6 Hz, H-4a), 3.77 (ddd, 1H, ^2J = 17.4 Hz, ^3J = 9.5 Hz, ^4J = 1.6 Hz, H-4b), 4.06 (dt, 1H, ^3J = 9.3 Hz, ^3J = 7.1 Hz, H-3), 4.96 (dt, 1H, ^3J = 6.9 Hz, ^4J = 1.6 Hz, H-2), 7.45–7.52 (m, 4H, Ar), 7.54–7.58 (m, 1H, Ar), 7.90–7.92 (m, 2H, Ar), 8.23–8.26 (m, 2H, Ar). *cis*-**4d:** 3.44 (ddd, 1H, ^2J = 17.3 Hz, ^3J = 5.7 Hz, ^4J = 1.4 Hz, H-4a), 3.61 (ddd, 1H, ^2J = 17.3 Hz, ^3J = 8.8 Hz, ^4J = 1.4 Hz, H-4b), 4.06–4.11 (m, 1H, H-3), 5.39 (dt, 1H, ^3J = 8.2 Hz, ^4J = 1.4 Hz, H-2), 7.42–7.44 (m, 2H, Ar), 7.45–7.52 (m, 2H, Ar), 7.56–7.59 (m, 1H, Ar), 7.92–7.94 (m, 2H, Ar), 8.22–8.24 (m, 2H, Ar). ^{13}C NMR (125.76 MHz, $CDCl_3$) δ (ppm): *trans*-**4d:** 43.66 (C-4), 48.60 (C-3), 68.64 (C-2), 118.52 (CN), 124.56, 127.83, 128.27, 128.90, 132.24, 132.33, 147.38, 147.59, 176.48 (C-5). *cis*-**4d:** 42.64 (C-4), 45.87 (C-3), 67.04 (C-2), 118.52 (CN), 124.27, 128.60, 128.94, 132.24, 132.33, 132.35, 147.38, 147.59, 176.48 (C-5). HRMS (ESI) (diastereoisomeric mixture): calculated for $C_{17}H_{13}N_3O_2$ [M+H]$^+$ *m/z* 292.1081, found 292.1085.

rel-(2R,3S)-5-(4-Methoxyphenyl)-3-phenyl-3,4-dihydro-2H-pyrrole-2-carbonitrile (*trans*-**4e**): Following the general procedure of Method A and Work up (A) white crystals of *trans*-**4e** (0.216 g, 78%) were obtained as an individual diastereoisomer, mp 89–91 °C. Following the general procedure of Method B, after purification by column chromatography (SiO_2, petroleum ether: acetone with variable polarity), white crystals of *trans*-**4e** (0.113 g, 41%) were obtained as an individual diastereoisomer, mp 89–91 °C. IR (KBr): 2244 (νCN), 1601 (νC=N), 1570, 1515, 1455 (νC=C), 839, 763, 702 (γC-H) cm^{-1}. ^{1}H NMR (500.13 MHz, $CDCl_3$) δ (ppm): 3.22 (ddd, 1H, ^2J = 17.3 Hz, ^3J = 7.5 Hz, ^4J = 1.8 Hz, H-4a), 3.65 (ddd, 1H, ^2J = 17.3 Hz, ^3J = 9.5 Hz, ^4J = 1.7 Hz, H-4b), 3.87 (s, 3H, CH_3O), 3.91 (dt, 1H, ^3J = 9.4 Hz, ^3J = 7.2 Hz, H-3), 4.90 (dt, 1H, ^3J = 6.9 Hz, ^4J = 1.7 Hz, H-2), 6.95–6.97 (m, 2H, Ar), 7.25–7.27 (m, 2H, Ar), 7.29–7.32 (m, 1H, Ar), 7.34–7.38 (m, 2H, Ar), 7.84–7.87 (m, 2H, Ar). ^{13}C NMR (125.76 MHz, $CDCl_3$) δ (ppm): 43.66 (C-4), 48.93 (C-3), 55.45 (CH_3O), 68.93 (C-2), 114.08, 119.46 (CN), 125.47, 126.75, 127.78, 129.23, 130.01, 140.39, 162.57, 175.93 (C-5). HRMS (ESI): calculated for $C_{18}H_{16}N_2O$ [M+H]$^+$ *m/z* 277.1335, found 277.1352.

rel-(2R,3R)-5-(4-Methoxyphenyl)-3-phenyl-3,4-dihydro-2H-pyrrole-2-carbonitrile (*cis*-**4e**): Following the general procedure of Method B, after purification by column chromatography (SiO_2, petroleum ether: acetone with variable polarity), white crystals of *cis*-**4e** (0.135 g, 49%) were obtained as an individual diastereoisomer, mp 82–84 °C. IR (KBr): 2240 (νCN), 1602 (νC=N), 1570, 1511, 1452 (νC=C), 835, 745, 698 (γC-H) cm^{-1}. ^{1}H NMR (500.13 MHz, $CDCl_3$) δ (ppm): 3.39 (ddd, 1H, ^2J = 17.1 Hz, ^3J = 6.4 Hz, ^4J = 1.3 Hz, H-4a), 3.47 (ddd, 1H, ^2J = 17.1 Hz, ^3J = 8.6 Hz, ^4J = 1.3 Hz, H-4b), 3.88 (s, 3H, CH_3O), 3.93 (td, 1H, ^3J = 8.4 Hz, ^3J = 6.4 Hz, H-3), 5.29 (dt, 1H, ^3J = 8.2 Hz, ^4J = 1.3 Hz H-2), 6.96–6.99 (m, 2H, Ar), 7.25–7.26 (m, 2H, Ar), 7.29–

7.32 (m, 1H, Ar), 7.34–7.38 (m, 2H, Ar), 7.86–7.89 (m, 2H, Ar). ^{13}C NMR (125.76 MHz, $CDCl_3$) δ (ppm): 42.23 (C-4), 46.26 (C-3), 55.47 (CH_3O), 67.21 (C-2), 114.10, 117.24 (CN), 125.57, 127.54, 127.99, 128.94, 130.02, 138.79, 162.60, 176.95 (C-5). HRMS (ESI): calculated for $C_{18}H_{16}N_2O$ $[M+H]^+$ *m/z* 277.1335, found 277.1335.

rel-(2R,3S)-3-(4-Chlorophenyl)-5-(4-methoxyphenyl)-3,4-dihydro-2H-pyrrole-2-carbonitrile (*trans*-**4f**): Following the general procedure of Method A and Work up (A) a pale-yellow oil of *trans*-**4f** (0.286 g, 92%) was obtained as an individual diastereoisomer, which then crystallized. Following the general procedure of Method B, after purification by column chromatography (SiO_2, petroleum ether: acetone with variable polarity), a pale-yellow oil of *trans*-**4f** (0.118 g, 38%) was obtained as an individual diastereoisomer. IR (KBr): 2244 (νCN), 1606 (νC=N), 1570, 1514, 1463 (νC=C), 833 (γC-H) cm^{-1}. ^{1}H NMR (500.13 MHz, $CDCl_3$) δ (ppm): 3.17 (ddd, 1H, ^2J = 17.3 Hz, ^3J = 7.4 Hz, ^4J = 1.6 Hz, H-4a), 3.65 (ddd, 1H, ^2J = 17.3 Hz, ^3J = 9.4 Hz, ^4J = 1.6 Hz, H-4b), 3.87 (s, 3H, CH_3O), 3.89 (dt, 1H, ^3J = 9.4 Hz, ^3J = 7.2 Hz, H-3), 4.85 (dt, 1H, ^3J = 6.8 Hz, ^4J = 1.6 Hz, H-2), 6.95–6.98 (m, 2H, Ar), 7.19–7.21 (m, 2H, Ar), 7.32–7.35 (m, 2H, Ar), 7.83–7.86 (m, 2H, Ar). ^{13}C NMR (125.76 MHz, $CDCl_3$) δ (ppm): 43.56 (C-4), 48.37 (C-3), 55.47 (CH_3O), 68.79 (C-2), 114.12, 119.18 (CN), 125.29, 128.12, 129.38, 130.03, 133.68, 138.80, 162.67, 175.84 (C-5). HRMS (ESI): calculated for $C_{18}H_{15}ClN_2O$ $[M+H]^+$ *m/z* 311.0946, found 311.0967.

rel-(2R,3R)-3-(4-Chlorophenyl)-5-(4-methoxyphenyl)-3,4-dihydro-2H-pyrrole-2-carbonitrile (*cis*-**4f**): Following the general procedure of Method B, after purification by column chromatography (SiO_2, petroleum ether: acetone with variable polarity) and reprecipitation from CH_2Cl_2–hexane, white crystals of *cis*-**4f** (0.152 gp 49%) were obtained as an individual diastereoisomer, mp 125–127 °C. IR (KBr): 2246 (νCN), 1601 (νC=N), 1570, 1513, 1466 (νC=C), 837 (γC-H) cm^{-1}. ^{1}H NMR (500.13 MHz, $CDCl_3$) δ (ppm): 3.33 (ddd, 1H, ^2J = 17. 1 Hz, ^3J = 6.1 Hz, ^4J = 1.4 Hz, H-4a), 3.48 (ddd, 1H, ^2J = 17.1 Hz, ^3J = 8.7 Hz, ^4J = 1.4 Hz, H-4b), 3.88 (s, 3H, CH_3O), 3.91 (td, 1H, ^3J = 8.5 Hz, ^3J = 6.1 Hz, H-3), 5.28 (dt, 1H, ^3J = 8.2 Hz, ^4J = 1.4 Hz, H-2), 6.96–6.99 (m, 2H, Ar), 7.17–7.20 (m, 2H, Ar), 7.31–7.34 (m, 2H, Ar), 7.85–7.88 (m, 2H, Ar). ^{13}C NMR (125.76 MHz, $CDCl_3$) δ (ppm): 42.37 (C-4), 45.67 (C-3), 55.48 (CH_3O), 67.06 (C-2), 114.15, 117.10 (CN), 125.36, 128.90, 129.14, 130.04, 133.86, 137.38, 162.71, 176.75 (C-5). HRMS (ESI): calculated for $C_{18}H_{15}ClN_2O$ $[M+H]^+$ *m/z* 311.0946, found 311.0941.

rel-(2R,3S)-3-(4-Fluorophenyl)-5-(3,4,5-trimethoxyphenyl)-3,4-dihydro-2H-pyrrole-2-carbonitrile (*trans*-**4g**): Following the general procedure of Method A and Work up (B), after purification by column chromatography (SiO_2, petroleum ether: acetone with variable polarity), white crystals of *trans*-**4g** (0.099 g, 28%) were obtained as an individual diastereoisomer mp 154–156 °C. Following the general procedure of Method B, after purification by column chromatography (SiO_2, petroleum ether: acetone with variable polarity), white crystals of *trans*-**4g** (0.181 g, 51%) were obtained as an individual diastereoisomer, mp 154–156 °C. IR (KBr): 2245 (νCN), 1604 (νC=N), 1577, 1512, 1465 (νC=C), 852, 830, 732 (γC-H) cm^{-1}. ^{1}H NMR (500.13 MHz, $CDCl_3$) δ (ppm): 3.20 (ddd, 1H, ^2J = 17.3 Hz, ^3J = 7.5 Hz, ^4J = 1.7 Hz, H-4a), 3.67 (ddd, 1H, ^2J = 17.3 Hz, ^3J = 9.4 Hz, ^4J = 1.7 Hz, H-4b), 3.92 (s, 3H, CH_3O), 3.92 (s, 6H, CH_3O), 3.90-3.95 (m, 1H, H-3), 4.88 (dt, 1H, ^3J = 7.0 Hz, ^4J = 1.7 Hz, H-2), 7.04–7.09 (m, 2H, Ar), 7.13 (s, 2H, Ar), 7.22–7.26 (m, 2H, Ar). ^{13}C NMR (125.76 MHz, $CDCl_3$) δ (ppm): 43.84 (C-4), 48.38 (C-3), 56.36 (CH_3O), 61.01 (CH_3O), 68.93 (C-2), 105.58, 116.19 (d, $^2J_{C-F}$ = 21.6 Hz, C-3′), 119.07 (CN), 127.94, 128.36 (d, $^3J_{C-F}$ = 8.1 Hz, C-2′), 135.85 (d, $^4J_{C-F}$ = 3.4 Hz, C-1′), 141.61, 153.31, 162.27 (d, $^1J_{C-F}$ = 247.4 Hz, C-4′), 176.19 (C-5). HRMS (ESI): calculated for $C_{20}H_{19}FN_2O_3$ $[M+H]^+$ *m/z* 355.1452, found 355.1475.

rel-(2R,3R)-3-(4-Fluorophenyl)-5-(3,4,5-trimethoxyphenyl)-3,4-dihydro-2H-pyrrole-2-carbonitrile (*cis*-**4g**): Following the general procedure of Method A and Work up (B), after purification by column chromatography (SiO_2, petroleum ether: acetone with variable polarity), white crystals of *cis*-**4g** (0.135 g, 38%) were obtained as an individual diastereoisomer, mp 129–132 °C. Following the general procedure of Method B, after purification by column chromatography (SiO_2, petroleum ether: acetone with variable polarity), white crystals of *cis*-**4g** (0.145 g, 41%) were obtained as an individual diastereoisomer, mp 129–132 °C. IR (KBr): 2246 (νCN), 1606 (νC=N), 1578, 1510, 1457 (νC=C), 852, 826, 797 (γC-H) cm^{-1}. ^{1}H NMR (500.13 MHz, $CDCl_3$) δ (ppm): 3.36 (ddd, 1H, ^2J = 17.1 Hz, ^3J = 6.0 Hz, ^4J = 1.5 Hz, H-4a), 3.50 (ddd, 1H, ^2J = 17.1 Hz, ^3J = 8.7 Hz, ^4J = 1.5 Hz, H-4b), 3.92 (s, 3H, CH_3O), 3.93 (s, 6H, CH_3O), 3.91–3.97 (m, 1H, H-3), 5.29

(dt, 1H, ^3J = 8.2 Hz, ^4J = 1.5 Hz, H-2), 7.03–7.08 (m, 2H, Ar), 7.16 (s, 2H, Ar), 7.21–7.25 (m, 2H, Ar). ^{13}C NMR (125.76 MHz, $CDCl_3$) δ (ppm): 42.69 (C-4), 45.61 (C-3), 56.38 (CH_3O), 61.01 (CH_3O), 67.26 (C-2), 105.60, 115.94 (d, $^2J_{C–F}$ = 21.5 Hz, C-3′), 116.98 (CN), 128.00, 129.16 (d, $^3J_{C–F}$ = 8.2 Hz, C-2′), 134.53 (d, $^4J_{C–F}$ = 3.4 Hz, C-1′), 141.63, 153.34, 162.40 (d, $^1J_{C–F}$ = 246.9 Hz, C-4′), 177.08 (C-5). HRMS (ESI): calculated for $C_{20}H_{19}FN_2O_3$ $[M+H]^+$ *m/z* 355.1452, found 355.1476.

rel-(2R,3S)-5-Phenyl-3-(pyridin-3-yl)-3,4-dihydro-2H-pyrrole-2-carbonitrile (*trans*-**4h**): Following the general procedure of Method A and Work up (B) white crystals of *trans*-**4h** (0.213 g, 86%) were obtained as an individual diastereoisomer, mp 115–117 °C. Following the general procedure of Method B, after purification by column chromatography (SiO_2, petroleum ether: acetone with variable polarity), white crystals of *trans*-**4h** (0.126 g, 51%) were obtained as an individual diastereoisomer, mp 115–117 °C. IR (KBr): 2248 (νCN), 1607 (νC=N), 1576, 1495, 1445 (νC=C), 1028 (νC-N), 768, 694 (γC-H) cm^{-1}. ^{1}H NMR (500.13 MHz, $CDCl_3$) δ (ppm): 3.26 (ddd, 1H, ^2J = 17.4 Hz, ^3J = 7.4 Hz, ^4J = 1.7 Hz, H-4a), 3.74 (ddd, 1H, ^2J = 17.4 Hz, ^3J = 9.5 Hz, ^4J = 1.7 Hz, H-4b), 3.97 (dt, 1H, ^3J = 9.4 Hz, ^3J = 7.1 Hz, H-3), 4.94 (dt, 1H, ^3J = 6.9 Hz, ^4J = 1.7 Hz, H-2), 7.32 (dd, 1H, ^3J = 8.0 Hz, ^3J = 4.8 Hz, pyridine H-5′), 7.46–7.49 (m, 2H, Ar), 7.53–7.56 (m, 1H, Ar), 7.59 (dt, 1H, ^3J = 8.0 Hz, ^4J = 2.0 Hz, pyridine H-4′), 7.89–7.91 (m, 2H, Ar), 8.58–8.60 (m, 2H, pyridine H-2′, H-6′). ^{13}C NMR (125.76 MHz, $CDCl_3$) δ (ppm): 43.47 (C-4), 46.46 (C-3), 68.58 (C-2), 118.66 (CN), 124.01, 128.24, 128.85, 132.21, 132.39, 134.06, 135.67, 148.52, 149.48, 176.66 (C-5). HRMS (ESI): calculated for $C_{16}H_{13}N_3$ $[M+H]^+$ *m/z* 248.1182, found 248.1184.

rel-(2R,3R)-5-Phenyl-3-(pyridin-3-yl)-3,4-dihydro-2H-pyrrole-2-carbonitrile (*cis*-**4h**): Following the general procedure of Method B, after purification by column chromatography (SiO_2, petroleum ether: acetone with variable polarity) and reprecipitation from acetone–hexane, white crystals of *cis*-**4h** (0.074 g, 30%) were obtained as an individual diastereoisomer, mp 110–112 °C. IR (KBr): 2251 (νCN), 1608 (νC=N), 1573, 1494, 1450 (νC=C), 1027 (νC-N), 769, 693 (γC-H) cm^{-1}. ^{1}H NMR (500.13 MHz, $CDCl_3$) δ (ppm): 3.40 (ddd, 1H, ^2J = 17.3 Hz, ^3J = 6.0 Hz, ^4J = 1.4 Hz, H-4a), 3.57 (ddd, 1H, ^2J = 17.3 Hz, ^3J = 8.8 Hz, ^4J = 1.4 Hz, H-4b), 3.99 (td, 1H, ^3J = 8.5 Hz, ^3J = 6.0 Hz, H-3), 5.36 (dt, 1H, ^3J = 8.2 Hz, ^4J = 1.4 Hz, H-2), 7.31 (dd, 1H, ^3J = 8.0, ^3J = 4.8 Hz, pyridine H-5′), 7.47–7.50 (m, 2H, Ar), 7.54–7.57 (m, 1H, Ar), 7.61 (dt, 1H, ^3J = 8.0 Hz, ^4J = 1.8 Hz, pyridine H-4′), 7.91–7.94 (m, 2H, Ar), 8.54 (d, 1H, ^4J = 2.2 Hz, pyridine H-6′), 8.58 (dd, 1H, ^3J = 4.8 Hz, ^4J = 1.5 Hz, pyridine H-2′). ^{13}C NMR (125.76 MHz, $CDCl_3$) δ (ppm): 42.50 (C-4), 43.68 (C-3), 67.05 (C-2), 116.73 (CN), 123.80, 128.24, 128.87, 132.21, 132.46, 134.42, 134.69, 149.27, 149.65, 177.47 (C-5). HRMS (ESI): calculated for $C_{16}H_{13}N_3$ $[M+H]^+$ *m/z* 248.1182, found 248.1180.

rel-(2R,3S)-3-(4-Methylphenyl)-5-phenyl-3,4-dihydro-2H-pyrrole-2-carbonitrile (*trans*-**4i**): Following the general procedure of Method B, after purification by column chromatography (SiO_2, petroleum ether: acetone with variable polarity), a colorless oil of *trans*-**4i** (0.094 g, 36%) was obtained as an individual diastereoisomer.

rel-(2R,3R)-3-(4-Methylphenyl)-5-phenyl-3,4-dihydro-2H-pyrrole-2-carbonitrile (*cis*-**4i**): Following the general procedure of Method B, after purification by column chromatography (SiO_2, petroleum ether: acetone with variable polarity) and reprecipitation from CH_2Cl_2–hexane, white crystals of *cis*-**4i** (0.112 g, 43%) were obtained as an individual diastereoisomer, mp 109–110 °C. IR (KBr): 2245 (νCN), 1615 (νC=N), 1576, 1517, 1449 (νC=C), 787, 763, 691 (γC-H) cm^{-1}. ^{1}H NMR (500.13 MHz, $CDCl_3$) δ (ppm): 2.34 (s, 3H, CH_3), 3.40 (ddd, 1H, ^2J = 17.2 Hz, ^3J = 6.4 Hz, ^4J = 1.4 Hz, H-4a), 3.49 (ddd, 1H, ^2J = 17.2 Hz, ^3J = 8.6 Hz, ^4J = 1.4Hz, H-4b), 3.92 (td, 1H, ^3J = 8.5 Hz, ^3J = 6.4 Hz, H-3), 5.30 (dt, 1H, ^3J = 8.3 Hz, ^4J = 1.4 Hz, H-2), 7.13–7.17 (m, 4H, Ar), 7.46–7.49 (m, 2H, Ar), 7.52–7.55 (m, 1H, Ar), 7.91–7.94 (m, 2H, Ar). ^{13}C NMR (125.76 MHz, $CDCl_3$) δ (ppm): 21.13 (CH_3), 42.46 (C-4), 45.84 (C-3), 67.44 (C-2), 117.11 (CN), 127.37, 128.20, 128.77, 129.64, 131.92, 132.86, 135.61, 137.74, 177.82 (C-5). HRMS (ESI): calculated for $C_{18}H_{16}N_2$ $[M+H]^+$ *m/z* 261.1386, found 261.1386.

rel-(2R,3S)-3-(4-Chlorophenyl)-5-phenyl-3,4-dihydro-2H-pyrrole-2-carbonitrile (*trans*-**4j**): Following the general procedure of Method B, after purification by column chromatography (SiO_2, cyclohexane: ethyl acetate with variable polarity), a colorless oil of *trans*-**4j** (0.126 g, 45%) was obtained as an individual diastereoisomer.

rel-(2R,3R)-3-(4-Chlorophenyl)-5-phenyl-3,4-dihydro-2H-pyrrole-2-carbonitrile (*cis*-**4j**): Following the general procedure of Method B, after purification by column chromatography (SiO_2, cyclohexane: ethyl acetate with variable polarity), a whitish oil of *cis*-**4j** (0.121 g, 43%) was obtained as an individual diastereoisomer. IR (capillary layer): 2245 (νCN), 1609 (νC=N), 1575, 1494, 1448 (νC=C), 829, 766, 692 (γC-H) cm^{-1}. ^{1}H NMR (500.13 MHz, $CDCl_3$) δ (ppm): 3.37 (ddd, 1H, ^2J = 17.3 Hz, ^3J = 6.0 Hz, ^4J = 1.5 Hz, H-4a), 3.52 (ddd, 1H, ^2J = 17.3 Hz, ^3J = 8.7 Hz, ^4J = 1.5 Hz, H-4b), 3.93 (td, 1H, ^3J = 8.5 Hz, ^3J = 6.0 Hz, H-3), 5.31 (dt, 1H, ^3J = 8.2 Hz, ^4J = 1.5 Hz, H-2), 7.17–7.20 (m, 2H, Ar), 7.32–7.34 (m, 2H, Ar), 7.46–7.50 (m, 2H, Ar), 7.53–7.57 (m, 1H, Ar), 7.91–7.93 (m, 2H, Ar). ^{13}C NMR (125.76 MHz, $CDCl_3$) δ (ppm): 42.57 (C-4), 45.58 (C-3), 67.22 (C-2), 116.87 (CN), 128.22, 128.84, 128.89, 129.17, 132.10, 132.60, 133.93, 137.26, 177.57 (C-5). HRMS (ESI): calculated for $C_{17}H_{13}ClN_2$ $[M+H]^+$ *m/z* 281.0840, found 281.0840.

rel-(2R,3S)-5-(4-Methylphenyl)-3-phenyl-3,4-dihydro-2H-pyrrole-2-carbonitrile (*trans*-**4k**): Following the general procedure of Method B, after purification by column chromatography (SiO_2, petroleum ether: acetone with variable polarity), white crystals of *trans*-**4k** (0.130 g, 50%) were obtained as an individual diastereoisomer, mp 74–76 °C.

rel-(2R,3R)-5-(4-Methylphenyl)-3-phenyl-3,4-dihydro-2H-pyrrole-2-carbonitrile (*cis*-**4k**): Following the general procedure of Method B, after purification by column chromatography (SiO_2, petroleum ether: acetone with variable polarity), white crystals of *cis*-**4k** (0.094 g, 36%) were obtained as an individual diastereoisomer, mp 113–115 °C. IR (KBr): 2250 (νCN), 1604 (νC=N), 1566, 1497, 1455 (νC=C), 822, 744, 696 (γC-H) cm^{-1}. ^{1}H NMR (500.13 MHz, $CDCl_3$) δ (ppm): 2.43 (s, 3H, CH_3), 3.41 (ddd, 1H, ^2J= 17.3 Hz, ^3J = 6.4 Hz, ^4J = 1.4 Hz, H-4a), 3.49 (ddd, 1H, ^2J = 17.3 Hz, ^3J = 8.6 Hz, ^4J = 1.4 Hz, H-4b), 3.94 (td, 1H, ^3J = 8.4 Hz, ^3J = 6.4 Hz, H-3), 5.31 (dt, 1H, ^3J = 8.1 Hz, ^4J = 1.4 Hz, H-2), 7.24–7.32 (m, 5H, Ar), 7.34–7.37 (m, 2H, Ar), 7.81–7.82 (m, 2H, Ar). ^{13}C NMR (125.76 MHz, $CDCl_3$) δ (ppm): 21.60 (CH_3), 42.36 (C-4), 46.19 (C-3), 67.29 (C-2), 117.12 (CN), 127.53, 128.02, 128.21, 128.95, 129.50, 130.13, 138.77, 142.55, 177.63 (C-5). HRMS (ESI): calculated for $C_{18}H_{16}N_2$ $[M+H]^+$ *m/z* 261.1386, found 261.1385.

rel-(2R,3S)-5-(4-Chlorophenyl)-3-phenyl-3,4-dihydro-2H-pyrrole-2-carbonitrile (*trans*-**4l**): Following the general procedure of Method B, after purification by column chromatography (SiO_2, petroleum ether: acetone with variable polarity), a colorless oil of *trans*-**4l** (0.095 g, 34%) was obtained as an individual diastereoisomer.

rel-(2R,3R)-5-(4-Chlorophenyl)-3-phenyl-3,4-dihydro-2H-pyrrole-2-carbonitrile (*cis*-**4l**): Following the general procedure of Method B, after purification by column chromatography (SiO_2, petroleum ether: acetone with variable polarity), white crystals of *cis*-**4l** (0.143 g, 51%) were obtained as an individual diastereoisomer, mp 109–111 °C. IR (KBr): 2246 (νCN), 1605 (νC=N), 1596, 1496, 1455 (νC=C), 829, 746, 697 (γC-H) cm^{-1}. ^{1}H NMR (500.13 MHz, $CDCl_3$) δ (ppm): 3.39 (ddd, 1H, ^2J = 17.3 Hz, ^3J = 6.3 Hz, ^4J = 1.5 Hz, H-4a), 3.49 (ddd, 1H, ^2J = 17.3 Hz, ^3J = 8.7 Hz, ^4J = 1.5 Hz, H-4b), 3.96 (td, 1H, ^3J = 8.5 Hz, ^3J = 6.3 Hz, H-3), 5.32 (dt, 1H, ^3J = 8.3 Hz, ^4J = 1.5 Hz, H-2), 7.23–7.25 (m, 2H, Ar), 7.30–7.34 (m, 1H, Ar), 7.35–7.39 (m, 2H, Ar), 7.44-7.47 (m, 2H, Ar), 7.85–7.88 (m, 2H, Ar). ^{13}C NMR (125.76 MHz, $CDCl_3$) δ (ppm): 42.38 (C-4), 46.23 (C-3), 67.39 (C-2), 116.82 (CN), 127.47, 128.15, 129.02, 129.11, 129.52, 131.21, 138.26, 138.45, 176.67 (C-5). HRMS (ESI): calculated for $C_{17}H_{13}ClN_2$ $[M+H]^+$ *m/z* 281.0840, found 281.0837.

rel-(2R,3S)-3-(3-Fluoro-4-methylphenyl)-5-(2-hydroxy-4,6-dimethoxyphenyl)-3,4-dihydro-2H-pyrrole-2-carbonitrile (*trans*-**4m**): Following the general procedure of Method A and Work up (A), after purification by column chromatography (SiO_2, petroleum ether: acetone with variable polarity), whitish crystals of *trans*-**4m** (0.131 g, 37%) were obtained as an individual diastereoisomer, mp 147–149 °C. IR (KBr): 3437 (νOH), 2244 (νCN), 1620 (νC=N), 1594, 1516, 1446 (νC=C), 859, 818 (γC-H) cm^{-1}. ^{1}H NMR (500.13 MHz, $CDCl_3$) δ (ppm): 2.26 (s, 3H, CH_3), 3.36 (ddd, 1H, ^2J = 18.5 Hz, ^3J = 7.0 Hz, ^4J = 1.4 Hz, H-4a), 3.75 (dt, 1H, ^3J = 9.3 Hz, ^3J = 7.1 Hz, H-3), 3.78 (s, 3H, CH_3O), 3.83 (s, 3H, CH_3O), 3.89 (ddd, 1H, ^2J = 18.5 Hz, ^3J = 9.4 Hz, ^4J = 1.4 Hz, H-4b), 4.79 (dt, 1H, ^3J = 6.8 Hz, ^4J = 1.4 Hz, H-2), 5.95 (d, 1H, ^4J = 2.3 Hz, Ar), 6.18 (d, 1H, ^4J = 2.3 Hz, Ar), 6.89 (d, 1H, $^3J_{H\text{–}F}$ = 10.5 Hz, Ar), 6.92–6.94 (m, 1H, Ar), 7.17 (t, 1H, ^3J = 7.8 Hz, Ar), 14.11 (s, 1H, OH). ^{13}C NMR (125.76 MHz, $CDCl_3$) δ (ppm): 14.23 (d, $^4J_{C\text{–}F}$ = 3.3 Hz, CH_3), 47.11 (d, $^4J_{C\text{–}F}$ = 1.4 Hz, C-3), 47.61 (C-4), 55.47 (CH_3O), 55.51 (CH_3O), 65.12 (C-2), 90.61, 93.84, 100.60, 113.43 (d, $^2J_{C\text{–}F}$ = 22.8 Hz, C-2′), 118.92 (CN), 122.29 (d, $^4J_{C\text{–}F}$ = 3.3 Hz, C-6′), 124.47 (d, $^2J_{C\text{–}F}$ = 17.1 Hz, C-4′), 132.20 (d, $^3J_{C\text{–}F}$ = 5.5 Hz, C-5′),

139.86 (d, $^3J_{C–F}$ = 7.0 Hz, C-1′), 161.39, 161.50 (d, $^1J_{C–F}$ = 246.1 Hz, C-3′), 164.73, 165.37, 179.00 (C-5). HRMS (ESI): calculated for $C_{20}H_{19}FN_2O_3$ [M+H]$^+$ *m/z* 355.1452, found 355.1460.

rel-(2R,3R)-3-(3-Fluoro-4-methylphenyl)-5-(2-hydroxy-4,6-dimethoxyphenyl)-3,4-dihydro-2H-pyrrole-2-carbonitrile (*cis*-**4m**): Following the general procedure of Method A and Work up (A), after purification by column chromatography (SiO_2, petroleum ether: acetone with variable polarity), whitish crystals of *cis*-**4m** (0.195 g, 55%) were obtained as an individual diastereoisomer, mp 175–177 °C. IR (KBr): 3433 (νOH), 2246 (νCN), 1615 (νC=N), 1582, 1511, 1465 (νC=C), 831, 796 (γC-H) cm^{-1}. ^{1}H NMR (500.13 MHz, $CDCl_3$) δ (ppm): 2.26 (s, 3H, CH_3), 3.57 (dd, 1H, ^2J = 18.2 Hz, ^3J = 6.3 Hz, H-4a), 3.69 (dd, 1H, ^2J = 18.2 Hz, ^3J = 8.7 Hz, H-4b), 3.74-3.78 (m, 1H, H-3), 3.80 (s, 3H, CH_3O), 3.83 (s, 3H, CH_3O), 5.19 (d, 1H, ^3J = 8.2 Hz, H-2), 5.97 (d, 1H, ^4J = 2.3 Hz, Ar), 6.19 (d, 1H, ^4J = 2.3 Hz, Ar), 6.91 (d, 1H, $^3J_{H–F}$ = 10.6 Hz, Ar), 6.94–6.95 (m, 1H, Ar), 7.17 (t, 1H, ^3J = 7.9 Hz, Ar), 14.15 (s, 1H, OH). ^{13}C NMR (125.76 MHz, $CDCl_3$) δ (ppm): 14.32 (d, $^4J_{C–F}$ = 3.4 Hz, CH_3), 44.45 (d, $^4J_{C–F}$ = 1.4 Hz, C-3), 46.11 (C-4), 55.50 (CH_3O), 55.52 (CH_3O), 63.43 (C-2), 90.63, 93.86, 100.72, 114.40 (d, $^2J_{C–F}$ = 23.0 Hz, C-2′), 116.81 (CN), 123.08 (d, $^4J_{C–F}$ = 3.3 Hz, C-6′), 124.62 (d, $^2J_{C–F}$ = 17.1 Hz, C-4′), 131.94 (d, $^3J_{C–F}$ = 5.5 Hz, C-5′), 138.10 (d, $^3J_{C–F}$ = 7.2 Hz, C-1′), 161.29 (d, $^1J_{C–F}$ = 245.7 Hz, C-3′), 161.43, 164.76, 165.36, 179.89 (C-5). HRMS (ESI): calculated for $C_{20}H_{19}FN_2O_3$ [M+H]$^+$ *m/z* 355.1452, found 355.1461.

rel-(2R,3S)-3-(3-Chloro-4-methylphenyl)-5-(2-hydroxy-4,6-dimethoxyphenyl)-3,4-dihydro-2H-pyrrole-2-carbonitrile (*trans*-**4n**): Following the general procedure of Method A and Work up (A), after purification by column chromatography (SiO_2, petroleum ether: acetone with variable polarity), white crystals of *trans*-**4n** (0.167 g, 45%) were obtained as an individual diastereoisomer, mp 132–134 °C. IR (KBr): 3437 (νOH), 2244 (νCN), 1618 (νC=N), 1591, 1501, 1442 (νC=C), 820, 795 (γC-H) cm^{-1}. ^{1}H NMR (500.13 MHz, $CDCl_3$) δ (ppm): 2.36 (s, 3H, CH_3), 3.36 (ddd, 1H, ^2J = 18.6 Hz, ^3J = 7.1 Hz, ^4J = 1.5 Hz, H-4a), 3.73 (dt, 1H, ^3J = 9.5 Hz, ^3J = 7.0 Hz, H-3), 3.78 (s, 3H, CH_3O), 3.83 (s, 3H, CH_3O), 3.89 (ddd, 1H, ^2J = 18.6 Hz, ^3J = 9.4 Hz, ^4J = 1.5 Hz, H-4b), 4.79 (dt, 1H, ^3J = 6.9 Hz, ^4J = 1.5 Hz, H-2), 5.95 (d, 1H, ^4J = 2.3 Hz, Ar), 6.18 (d, 1H, ^4J = 2.3 Hz, Ar), 7.04 (dd, 1H, ^3J = 7.8 Hz, ^4J = 1.7 Hz, Ar), 7.22 (d, 1H, ^3J = 7.8 Hz, Ar), 7.23 (d, 1H, ^3J = 1.7 Hz, Ar), 14.09 (s, 1H, OH). ^{13}C NMR (125.76 MHz, $CDCl_3$) δ (ppm): 19.70 (CH_3), 46.98 (C-3), 47.69 (C-4), 55.48 (CH_3O), 55.52 (CH_3O), 65.14 (C-2), 90.62, 93.85, 100.59, 118.89 (CN), 125.16, 127.39, 131.66, 135.04, 135.74, 139.51, 161.39, 164.74, 165.38, 178.94 (C-5). HRMS (ESI): calculated for $C_{20}H_{19}ClN_2O_3$ [M+H]$^+$ *m/z* 371.1157, found 371.1171.

rel-(2R,3R)-3-(3-Chloro-4-methylphenyl)-5-(2-hydroxy-4,6-dimethoxyphenyl)-3,4-dihydro-2H-pyrrole-2-carbonitrile (*cis*-**4n**): Following the general procedure of Method A and Work up (A), after purification by column chromatography (SiO_2, petroleum ether: acetone with variable polarity), white crystals of *cis*-**4n** (0.189 g, 51%) were obtained as an individual diastereoisomer, mp 188–190 °C. IR (KBr): 3437 (νOH), 2244 (νCN), 1617 (νC=N), 1585, 1498, 1466 (νC=C), 877, 823 (γC-H) cm^{-1}. ^{1}H NMR (500.13 MHz, $CDCl_3$) δ (ppm): 2.36 (s, 3H, CH_3), 3.54–3.59 (m, 1H, H-4a), 3.67–3.77 (m, 2H, H-3, H-4b), 3.80 (s, 3H, CH_3O), 3.83 (s, 3H, CH_3O), 5.18–5.20 (m, 1H, H-2), 5.97 (d, 1H, ^4J = 2.3 Hz, Ar), 6.19 (d, 1H, ^4J = 2.3 Hz, Ar), 7.07 (dd, 1H, ^3J = 7.9 Hz, ^4J = 1.5 Hz, Ar), 7.23 (d, 1H, ^3J = 7.9 Hz, Ar), 7.25 (d, 1H, ^3J = 1.5 Hz, Ar), 14.14 (s, 1H, OH). ^{13}C NMR (125.76 MHz, $CDCl_3$) δ (ppm): 19.76 (CH_3), 44.31 (C-3), 46.08 (C-4), 55.51 (CH_3O), 55.52 (CH_3O), 63.39 (C-2), 90.64, 93.85, 100.72, 116.77 (CN), 125.79, 128.47, 131.45, 134.68, 135.87, 137.67, 161.42, 164.76, 165.37, 179.88 (C-5). HRMS (ESI): calculated for $C_{20}H_{19}ClN_2O_3$ [M+H]$^+$ *m/z* 371.1157, found 371.1171.

rel-(2R,3S)-3-(3-Bromo-4-methylphenyl)-5-(2-hydroxy-4,6-dimethoxyphenyl)-3,4-dihydro-2H-pyrrole-2-carbonitrile (*trans*-**4o**): Following the general procedure of Method A and Work up (A), after purification by column chromatography (SiO_2, petroleum ether: acetone with variable polarity), whitish crystals of *trans*-**4o** (0.145 g, 35%) were obtained as an individual diastereoisomer, mp 134–136 °C. IR (KBr): 3430 (νOH), 2249 (νCN), 1614 (νC=N), 1586, 1496, 1462 (νC=C), 882, 825 (γC-H) cm^{-1}. ^{1}H NMR (500.13 MHz, $CDCl_3$) δ (ppm): 2.39 (s, 3H, CH_3), 3.35 (ddd, 1H, ^2J = 18.6 Hz, ^3J = 7.2 Hz, ^4J = 1.3 Hz, H-4a), 3.72 (dt, 1H, ^3J = 9.5 Hz, ^3J = 7.1 Hz, H-3), 3.78 (s, 3H, CH_3O), 3.83 (s, 3H, CH_3O), 3.89 (ddd, 1H, ^2J = 18.6 Hz, ^3J = 9.5 Hz, ^4J = 1.3 Hz, H-4b), 4.80 (dt, 1H, ^3J = 7.0 Hz, ^4J = 1.3 Hz, H-2), 5.95 (d, 1H, ^4J = 2.3 Hz, Ar), 6.18 (d, 1H, ^4J = 2.3 Hz, Ar), 7.09 (dd, 1H, ^3J = 7.8 Hz, ^4J = 1.7 Hz, H-6′), 7.22 (d, 1H, ^3J = 7.8 Hz, H-5′), 7.43 (d, 1H, ^3J = 1.7 Hz, H-2′), 14.07 (s, 1H, OH). ^{13}C NMR (125.76 MHz, $CDCl_3$) δ (ppm): 22.54 (CH_3), 46.88 (C-3), 47.74 (C-4), 55.48 (CH_3O), 55.51 (CH_3O), 65.16 (C-2), 90.61, 93.84, 100.58, 118.88 (CN), 125.52, 125.83, 130.64, 131.48, 137.61,

139.67, 161.38, 164.74, 165.38, 178.90 (C-5). HRMS (ESI): calculated for $C_{20}H_{19}BrN_2O_3$ $[M+H]^+$ *m/z* 415.0652, found 415.0665.

rel-(2R,3R)-3-(3-Bromo-4-methylphenyl)-5-(2-hydroxy-4,6-dimethoxyphenyl)-3,4-dihydro-2H-pyrrole-2-carbonitrile (*cis*-**4o**): Following the general procedure of Method A and Work up (A), after purification by column chromatography (SiO_2, petroleum ether: acetone with variable polarity), whitish crystals of *cis*-**4o** (0.212 g, 51%) were obtained as an individual diastereoisomer, mp 191–193 °C. IR (KBr): 3430 (νOH), 2245 (νCN), 1617 (νC=N), 1583, 1496, 1466 (νC=C), 878, 826 (γC-H) cm^{-1}. ^1H NMR (500.13 MHz, $CDCl_3$) δ (ppm): 2.39 (s, 3H, CH_3), 3.53–3.59 (m, 1H, H-4a), 3.67–3.76 (m, 2H, H-3, H-4b), 3.80 (s, 3H, CH_3O), 3.83 (s, 3H, CH_3O), 5.17–5.21 (m, 1H, H-2), 5.96 (d, 1H, ^4J = 2.4 Hz, Ar), 6.19 (d, 1H, ^4J = 2.4 Hz, Ar), 7.12 (dd, 1H, ^3J = 7.8 Hz, ^4J = 1.6 Hz, H-6′), 7.23 (d, 1H, ^3J = 7.8 Hz, H-5′), 7.44 (d, 1H, ^4J = 1.6 Hz, H-2′), 14.14 (s, 1H, OH). ^{13}C NMR (125.76 MHz, $CDCl_3$) δ (ppm): 22.60 (CH_3), 44.20 (C-3), 46.09 (C-4), 55.51 (CH_3O), 55.52 (CH_3O), 63.39 (C-2), 90.63, 93.85, 100.72, 116.76 (CN), 125.18, 126.40, 131.28, 131.76, 137.74, 137.82, 161.42, 164.77, 165.37, 179.87 (C-5). HRMS (ESI): calculated for $C_{20}H_{19}BrN_2O_3$ $[M+H]^+$ *m/z* 415.0652, found 415.0668.

Methyl rel-(2R,3S)-3,5-diaryl-3,4-dihydro-2H-pyrrole-2-carboxylate

Methyl rel-(2R,3S)-3,5-diphenyl-3,4-dihydro-2H-pyrrole-2-carboxylate (*trans*-**5a**): Following the general procedure, after purification by column chromatography (SiO_2, petroleum ether: acetone = 20:1), a colorless oil of *trans*-**5a** (0.165 g, 59% from starting compound *trans*-**4a**, and 0.179 g, 64% from starting compound *cis*-**4a**) was obtained as an individual diastereoisomer.

IR (capillary layer): 1741 (νC=O), 1614 (νC=N), 1575, 1495, 1448 (νC=C), 760, 693 (γC-H) cm^{-1}. ^1H NMR (500.13 MHz, $CDCl_3$) δ (ppm): 3.17 (ddd, 1H, ^2J = 17.4 Hz, ^3J = 6.6 Hz, ^4J = 1.7 Hz, H-4a), 3.67 (ddd, 1H, ^2J = 17.4 Hz, ^3J = 9.7 Hz, ^4J = 2.1 Hz, H-4b), 3.79 (s, 3H, $COOCH_3$), 3.90 (dt, 1H, ^3J = 9.6 Hz, ^3J = 6.3 Hz, H-3), 4.96 (dt, 1H, ^3J = 6.1 Hz, ^4J = 1.9 Hz, H-2), 7.22–7.26 (m, 3H, Ar), 7.30–7.34 (m, 2H, Ar), 7.42–7.45 (m, 2H, Ar), 7.46–7.50 (m, 1H, Ar), 7.90–7.93 (m, 2H, Ar). ^{13}C NMR (125.76 MHz, $CDCl_3$) δ (ppm): 44.71 (C-4), 46.29 (C-3), 52.44 ($COO\underline{C}H_3$), 82.58 (C-2), 126.98, 126.99, 128.13, 128.55, 128.92, 131.22, 133.57, 143.20, 172.63 ($\underline{C}OOCH_3$), 175.07 (C-5). HRMS (ESI): calculated for $C_{18}H_{17}NO_2$ $[M+H]^+$ *m/z* 280.1332, found 280.1337.

Methyl rel-(2R,3S)-3-(4-methoxyphenyl)-5-phenyl-3,4-dihydro-2H-pyrrole-2-carboxylate (*trans*-**5b**): Following the general procedure, after purification by column chromatography (SiO_2, petroleum ether: ethyl acetate = 8:1), a yellow oil of *trans*-**5b** (0.210 g, 68%) was obtained as an individual diastereoisomer. IR (capillary layer): 1740 (νC=O), 1612 (νC=N), 1575, 1514, 1448 (νC=C), 830, 764, 694 (γC-H) cm^{-1}. ^1H NMR (500.13 MHz, $CDCl_3$) δ (ppm): 3.13 (ddd, 1H, ^2J = 17.3 Hz, ^3J = 6.7 Hz, ^4J = 1.7, H-4a), 3.64 (ddd, 1H, ^2J = 17.3 Hz, ^3J = 9.6 Hz, ^4J = 2.0, H-4b), 3.78 (s, 3H, $COOCH_3$), 3.79 (s, 3H, CH_3O), 3.85 (dt, 1H, ^3J = 9.6 Hz, ^3J = 6.4 Hz, H-3), 4.91 (dt, 1H, ^3J = 6.2 Hz, ^4J = 1.8 Hz, H-2), 6.84–6.87 (m, 2H, Ar), 7.14–7.17 (m, 2H, Ar), 7.41–7.45 (m, 2H, Ar), 7.46–7.50 (m, 1H, Ar), 7.90–7.92 (m, 2H, Ar). ^{13}C NMR (125.76 MHz, $CDCl_3$) δ (ppm): 44.72 (C-4), 45.65 (C-3), 52.40 ($COO\underline{C}H_3$), 55.31 (CH_3O), 82.62 (C-2), 114.26, 127.98, 128.10, 128.54, 131.19, 133.63, 135.14, 158.55 (C-4′), 172.71 ($\underline{C}OOCH_3$), 175.12 (C-5). HRMS (ESI): calculated for $C_{19}H_{19}NO_3$ $[M+H]^+$ *m/z* 310.1438, found 310.1456.

Methyl rel-(2R,3S)-3-(4-(dimethylamino)phenyl)-5-phenyl-3,4-dihydro-2H-pyrrole-2-carboxylate (*trans*-**5c**): Following the general procedure, after purification by column chromatography (SiO_2, petroleum ether: ethyl acetate = 5:1), an oil of *trans*-**5c** (0.235 g, 73%) was obtained as an individual diastereoisomer. IR (capillary layer): 1741 (νC=O), 1615 (νC=N), 1575, 1523, 1448 (νC=C), 817, 764, 694 (γC-H) cm^{-1}. ^1H NMR (500.13 MHz, $CDCl_3$) δ (ppm): 2.92 (s, 6H, $N(CH_3)_2$), 3.12 (ddd, 1H, ^2J = 17.3 Hz, ^3J = 6.8 Hz, ^4J = 1.7 Hz, H-4a), 3.61 (ddd, 1H, ^2J = 17.3 Hz, ^3J = 9.6 Hz, ^4J = 2.0 Hz, H-4b), 3.77 (s, 3H, $COOCH_3$), 3.81 (dt, 1H, ^3J = 9.6 Hz, ^3J = 6.6 Hz, H-3), 4.90 (dt, 1H, ^3J = 6.2 Hz, ^4J = 1.8 Hz, H-2), 6.68–6.71 (m, 2H, Ar), 7.10–7.13 (m, 2H, Ar), 7.41–7.44 (m, 2H, Ar), 7.45–7.49 (m, 1H, Ar), 7.90–7.92 (m, 2H, Ar). ^{13}C NMR (125.76 MHz, $CDCl_3$) δ (ppm): 40.68 ($N(CH_3)_2$), 44.69 (C-4), 45.64 (C-3), 52.33 ($COO\underline{C}H_3$), 82.58 (C-2), 113.00, 127.64, 128.09, 128.50, 130.77, 131.09, 133.76, 149.67, 172.90 ($\underline{C}OOCH_3$), 175.26 (C-5). HRMS (ESI): calculated for $C_{20}H_{22}N_2O_2$ $[M+H]^+$ *m/z* 323.1754, found 323.1721.

Methyl rel-(2R,3S)-3-(4-nitrophenyl)-5-phenyl-3,4-dihydro-2H-pyrrole-2-carboxylate (*trans*-**5d**): Following the general procedure, after purification by column chromatography (SiO_2, petroleum ether: acetone = 10:1), a

colorless oil of *trans*-**5d** (0.126 g, 39%) was obtained as an individual diastereoisomer. IR (capillary layer): 1739 (νC=O), 1606 (νC=N), 1575, 1519, 1448 (νC=C), 1519, 1347 (νNO_2), 855, 765, 694 (γC-H) cm^{-1}. ^{1}H NMR (500.13 MHz, $CDCl_3$) δ (ppm): 3.19 (ddd, 1H, ^2J = 17.4 Hz, ^3J = 6.6 Hz, ^4J = 1.7 Hz, H-4a), 3.73 (ddd, 1H, ^2J = 17.4 Hz, ^3J = 9.7 Hz, ^4J = 2.1 Hz, H-4b), 3.81 (s, 3H, $COOCH_3$), 4.03 (dt, 1H, ^3J = 9.7 Hz, ^3J = 6.4 Hz, H-3), 4.96 (dt, 1H, ^3J = 6.1 Hz, ^4J = 1.8 Hz, H-2), 7.40–7.43 (m, 2H, Ar), 7.44–7.47 (m, 2H, Ar), 7.49–7.53 (m, 1H, Ar), 7.90–7.93 (m, 2H, Ar), 8.18–8.21 (m, 2H, Ar). ^{13}C NMR (125.76 MHz, $CDCl_3$) δ (ppm): 44.49 (C-4), 46.06 (C-3), 52.69 (COO$\underline{C}H_3$), 82.33 (C-2), 124.24, 128.01, 128.15, 128.68, 131.57, 133.09, 147.03, 150.62, 171.97 ($\underline{C}OOCH_3$), 174.65 (C-5). HRMS (ESI): calculated for $C_{18}H_{16}N_2O_4$ [M+H]$^+$ *m/z* 325.1183, found 325.1186.

Methyl rel-(2R,3S)-5-(4-methoxyphenyl)-3-phenyl-3,4-dihydro-2H-pyrrole-2-carboxylate (*trans*-**5e**): Following the general procedure, after purification by column chromatography (SiO_2, petroleum ether: ethyl acetate = 5:1), a colorless oil of *trans*-**5e** (0.247 g, 80%) was obtained as an individual diastereoisomer. IR (capillary layer): 1740 (νC=O), 1606 (νC=N), 1572, 1514, 1435 (νC=C), 837, 761, 702 (γC-H) cm^{-1}. ^{1}H NMR (500.13 MHz, $CDCl_3$) δ (ppm): 3.14 (ddd, 1H, ^2J = 17.2 Hz, ^3J = 6.6 Hz, ^4J = 1.5 Hz, H-4a), 3.63 (ddd, 1H, ^2J = 17.2 Hz, ^3J = 9.7 Hz, ^4J = 1.9 Hz, H-4b), 3.78 (s, 3H, $COOCH_3$), 3.86 (s, 3H, CH_3O), 3.87 (dt, 1H, ^3J = 9.9 Hz, ^3J = 6.3 Hz, H-3), 4.92 (dt, 1H, ^3J = 6.0 Hz, ^4J = 1.6 Hz, H-2), 6.92–6.95 (m, 2H, Ar), 7.22–7.25 (m, 3H, Ar), 7.30–7.34 (m, 2H, Ar), 7.85–7.88 (m, 2H, Ar). ^{13}C NMR (125.76 MHz, $CDCl_3$) δ (ppm): 44.63 (C-4), 46.37 (C-3), 52.39 (COO$\underline{C}H_3$), 55.39 (CH_3O), 82.42 (C-2), 113.85, 126.37, 126.91, 127.00, 128.89, 129.84, 143.33, 162.02, 172.85 ($\underline{C}OOCH_3$), 174.30 (C-5). HRMS (ESI): calculated for $C_{19}H_{19}NO_3$ [M+H]$^+$ *m/z* 310.1438, found 310.1459.

Methyl rel-(2R,3S)-3-(4-chlorophenyl)-5-(4-methoxyphenyl)-3,4-dihydro-2H-pyrrole-2- carboxylate (*trans*-**5f**): Following the general procedure, after purification by column chromatography (SiO_2, petroleum ether: acetone = 6:1), a colorless oil of *trans*-**5f** (0.244 g, 71%) was obtained as an individual diastereoisomer. IR (capillary layer): 1740 (νC=O), 1606 (νC=N), 1572, 1514, 1435 (νC=C), 833 (γC-H) cm^{-1}. ^{1}H NMR (500.13 MHz, $CDCl_3$) δ (ppm): 3.08 (ddd, 1H, ^2J = 17.2 Hz, ^3J = 6.6 Hz, ^4J = 1.5 Hz, H-4a), 3.62 (ddd, 1H, ^2J = 17.2 Hz, ^3J = 9.7 Hz, ^4J = 1.9 Hz, H-4b), 3.78 (s, 3H, COOC$\underline{H}_3$), 3.85 (dt, 1H, ^3J = 9.7 Hz, ^3J = 6.3 Hz, H-3), 3.86 (s, 3H, CH_3O), 4.87 (dt, 1H, ^3J = 6.0 Hz, ^4J = 1.7 Hz, H-2), 6.92–6.95 (m, 2H, Ar), 7.15–7.18 (m, 2H, Ar), 7.27–7.30 (m, 2H, Ar), 7.83–7.87 (m, 2H, Ar). ^{13}C NMR (125.76 MHz, $CDCl_3$) δ (ppm): 44.51 (C-4), 45.81 (C-3), 52.46 (COO$\underline{C}H_3$), 55.40 (CH_3O), 82.33 (C-2), 113.89, 126.19, 128.39, 129.00, 129.84, 132.68, 141.75, 162.11, 172.61 ($\underline{C}OOCH_3$), 174.14 (C-5). HRMS (ESI): calculated for $C_{19}H_{18}ClNO_3$ [M+H]$^+$ *m/z* 344.1048, found 344.1074.

Methyl rel-(2R,3S)-3-(4-fluorophenyl)-5-(3,4,5-trimethoxyphenyl)-3,4-dihydro-2H- pyrrole-2-carboxylate (*trans*-**5g**): Following the general procedure, after purification by column chromatography (SiO_2, petroleum ether: acetone = 6:1), white crystals of *trans*-**5g** (0.205 g, 53% from starting compound *cis*-**4g**) was obtained as an individual diastereoisomer, mp 124–126 °C. IR (KBr): 1752 (νC=O), 1611 (νC=N), 1602, 1582, 1510, 1459 (νC=C), 834, 766 (γC-H) cm^{-1}. ^{1}H NMR (500.13 MHz, $CDCl_3$) δ (ppm): 3.11 (ddd, 1H, ^2J = 17.2 Hz, ^3J = 6.4 Hz, ^4J = 1.4 Hz, H-4a), 3,65 (ddd, 1H, ^2J = 17.2 Hz, ^3J = 9.6 Hz, ^4J = 1.9 Hz, H-4b), 3.79 (s, 3H, $COOCH_3$), 3.87 (dt, 1H, ^3J = 9.6 Hz, ^3J = 6.1 Hz, H-3), 3.90 (s, 3H, CH_3O), 3.91 (s, 6H, CH_3O), 4.91 (dt, 1H, ^3J = 6.0 Hz, ^4J = 1.8 Hz, H-2), 6.99–7.04 (m, 2H, Ar), 7.14 (s, 2H, Ar), 7.18–7.22 (m, 2H, Ar). ^{13}C NMR (125.76 MHz, $CDCl_3$) δ (ppm): 44.85 (C-4), 45.78 (C-3), 52.48 (COO$\underline{C}H_3$), 56.35 (CH_3O), 60.97 (CH_3O), 82.52 (C-2), 105.48, 115.76 (d, $^2J_{C\text{-}F}$ = 21.3 Hz, C-3′), 128.45 (d, $^3J_{C\text{–}F}$ = 7.8 Hz, C-2′), 128.95, 138.87 (d, $^4J_{C\text{–}F}$ = 3.3 Hz, C-1′), 141.00, 153.21, 161.82 (d, $^1J_{C\text{–}F}$ = 245.7 Hz, C-4′), 172.46 ($\underline{C}OOCH_3$), 174.57 (C-5). HRMS (ESI): calculated for $C_{21}H_{22}FNO_5$ [M+H]$^+$ *m/z* 388.1555, found 388.1582.

Methyl rel-(2R,3S)-3-(4-methylphenyl)-5-phenyl-3,4-dihydro-2H-pyrrole-2-carboxylate (*trans*-**5i**): Following the general procedure, after purification by column chromatography (SiO_2, petroleum ether: acetone with variable polarity), a colorless oil of *trans*-**5i** (0.188 g, 64%) was obtained as an individual diastereoisomer. IR (capillary layer): 1742 (νC=O), 1614 (νC=N), 1575, 1516, 1448 (νC=C), 816, 763, 693 (γC-H) cm^{-1}. ^{1}H NMR (500.13 MHz, $CDCl_3$) δ (ppm): 2.33 (s, 3H, CH_3), 3.15 (ddd, 1H, ^2J = 17.3 Hz, ^3J = 6.7 Hz, ^4J = 1.6 Hz, H-4a), 3.65 (ddd, 1H, ^2J = 17.3 Hz, ^3J = 9.7 Hz, ^4J = 2.0 Hz, H-4b), 3.78 (s, 3H, $COOCH_3$), 3.86 (dt, 1H, ^3J = 9.7 Hz, ^3J = 6.4 Hz, H-3), 4.93 (dt, 1H, ^3J = 6.2 Hz, ^4J = 1.7 Hz, H-2), 7.13-7.15 (m, 4H, Ar), 7.41–7.45 (m, 2H, Ar), 7.46–7.50 (m, 1H, Ar), 7.90–7.92 (m, 2H, Ar). ^{13}C NMR (125.76 MHz, $CDCl_3$) δ (ppm): 21.01 (CH_3), 44.56 (C-4), 45.79 (C-3), 52.52 (COO$\underline{C}H_3$), 81.74 (C-2), 126.81, 128.02, 128.49, 128.67, 129.63, 131.78, 136.78, 139.73,

172.27 ($\underline{C}OOCH_3$), 175.84 (C-5). HRMS (ESI): calculated for $C_{19}H_{19}NO_2$ $[M+H]^+$ *m/z* 294.1489, found 294.1485.

3-(4-Methylphenyl)-5-phenyl-3,4-dihydro-2H-pyrrole-2-carboxamide (**6i**): Following the general procedure, after purification by column chromatography (SiO_2, petroleum ether: acetone with variable polarity), yellow crystals of **6i** (0.072 g, 26%) were obtained as a diastereoisomeric mixture, mp 99–107 °C. IR (KBr): 3451, 3382 (νNH_2), 1681 (νC=O), 1616 (νC=N), 1576, 1514, 1448 (νC=C), 811, 763, 696 (γC-H) cm^{-1}. ^1H NMR (500.13 MHz, $CDCl_3$) δ (ppm): *trans*-**6i:** 2.32 (s, 3H, CH_3), 3.15 (ddd, 1H, ^2J = 17.7 Hz, ^3J = 6.6 Hz, ^4J = 1.8 Hz, H-4a), 3.59 (ddd, 1H, ^2J = 17.7 Hz, ^3J = 9.9 Hz, ^4J = 2.1 Hz, H-4b), 3.88 (dt, 1H, ^3J = 9.9 Hz, ^3J = 6.5 Hz, H-3), 4.84 (d, 1H, ^3J = 6.2 Hz, ^4J = 1.9 Hz, H-2), 5.57 (br. s, 1H, $CONH_2$), 6.53 (br. s, 1H, $CONH_2$), 7.12–7.14 (m, 2H, H-3′), 7.18–7.21 (m, 2H, H-2′), 7.44–7.55 (m, 3H, H-3″, H-4″), 7.94–7.97 (m, 2H, H-2″). *cis*-**6i:** 2.25 (s, 3H, CH_3), 3.33–3.37 (m, 1H, H-4a), 3.42 (ddd, 1H, ^2J = 17.1 Hz, ^3J = 8.1 Hz, ^4J = 2.9 Hz, H-4b), 4.02–4.06 (m, 1H, H-3), 4.96–4.99 (m, 1H, H-2), 5.39 (br. s, 1H, $CONH_2$), 6.76 (br. s, 1H, $CONH_2$), 6.95–6.97 (m, 2H, H-2′), 6.99–7.01 (m, 2H, H-3′), 7.44–7.55 (m, 3H, H-3″, H-4″), 7.94–7.97 (m, 2H, H-2″). ^{13}C NMR (125.76 MHz, $CDCl_3$) δ (ppm): *trans*-**6i:** 21.03 (CH_3), 44.83 (C-4), 45.26 (C-3), 83.06 (C-2), 127.00 (C-3′), 127.92* or 127.97* (C-3″, C-2″), 128.65* or 128.73* (C-3″, C-2″), 129.52 (C-2′), 131.31 (C-4″), 133.49* or 133.54* (C-1″), 136.26 (C-4′), 141.23 (C-1′), 174.27 (C-5), 174.72 ($CONH_2$). *cis*-**6i:** 21.00 (CH_3), 44.56 (C-4), 45.25 (C-3), 79.92 (C-2), 127.34 (C-3′), 127.92* or 127.97* (C-3″, C-2″), 128.65* or 128.73* (C-3″, C-2″), 129.01 (C-2′), 131.45 (C-4″), 133.49* or 133.54* (C-1″), 136.47 (C-4′), 138.51 (C-1′), 172.99 ($CONH_2$), 176.03 (C-5). HRMS (ESI): calculated for $C_{18}H_{18}N_2O$ $[M+H]^+$ m/z 279.1492, found 279.1493.

Methyl rel-(2R,3S)-3-(4-chlorophenyl)-5-phenyl-3,4-dihydro-2H-pyrrole-2-carboxylate (*trans*-**5j**): Following the general procedure, after purification by column chromatography (SiO_2, petroleum ether: acetone = 20:1), a colorless oil of *trans*-**5j** (0.198 g, 63%) was obtained as an individual diastereoisomer. IR (capillary layer): 1741 (νC=O), 1614 (νC=N), 1575, 1494, 1448 (νC=C), 826, 763, 693 (γC-H) cm^{-1}. ^1H NMR (500.13 MHz, $CDCl_3$) δ (ppm): 3.12 (ddd, 1H, ^2J = 17.3 Hz, ^3J = 6.6 Hz, ^4J = 1.7 Hz, H-4a), 3.66 (ddd, 1H, ^2J = 17.3 Hz, ^3J = 9.7 Hz, ^4J = 2.1 Hz, H-4b), 3.79 (s, 3H, $COOCH_3$), 3.88 (dt, 1H, ^3J = 9.6 Hz, ^3J = 6.3 Hz, H-3), 4.91 (dt, 1H, ^3J = 6.1 Hz, ^4J = 1.9 Hz, H-2), 7.16–7.19 (m, 2H, Ar), 7.28–7.30 (m, 2H, Ar), 7.42–7.46 (m 2H, Ar), 7.47–7.51 (m, 1H, Ar), 7.89–7.92 (m, 2H, Ar). ^{13}C NMR (125.76 MHz, $CDCl_3$) δ (ppm): 44.59 (C-4), 45.72 (C-3), 52.51 ($COO\underline{C}H_3$), 82.48 (C-2), 128.11, 128.38, 128.59, 129.04, 131.34, 132.76, 133.40, 141.60, 172.39 ($\underline{C}OOCH_3$), 174.91 (C-5). HRMS (ESI): calculated for $C_{18}H_{16}ClNO_2$ $[M+H]^+$ *m/z* 314.0942, found 314.0955.

Methyl rel-(2R,3S)-5-(4-methylphenyl)-3-phenyl-3,4-dihydro-2H-pyrrole-2-carboxylate (*trans*-**5k**): Following the general procedure, after purification by column chromatography (SiO_2, cyclohexane: ethyl acetate = 10:1), a pale yellow oil of *trans*-**5k** (0.188 g, 64%) was obtained as an individual diastereoisomer. IR (capillary layer): 1741 (νC=O), 1611 (νC=N), 1568, 1513, 1454 (νC=C), 818, 759, 701 (γC-H) cm^{-1}. ^1H NMR (500.13 MHz, $CDCl_3$) δ (ppm): 2.40 (s, 3H, CH_3), 3.15 (ddd, 1H, ^2J = 17.3 Hz, ^3J = 6.6 Hz, ^4J = 1.7 Hz, H-4a), 3.64 (ddd, 1H, ^2J = 17.3 Hz, ^3J = 9.7 Hz, ^4J = 2.0 Hz, H-4b), 3.78 (s, 3H, $COOCH_3$), 3.88 (dt, 1H, ^3J = 9.7 Hz, ^3J = 6.3 Hz, H-3), 4.94 (dt, 1H, ^3J = 6.1 Hz, ^4J = 1.9 Hz, H-2), 7.23–7.26 (m, 5H, Ar), 7.30–7.34 (m, 2H, Ar), 7.79–7.81 (m, 2H, Ar). ^{13}C NMR (125.76 MHz, $CDCl_3$) δ (ppm): 21.53 (CH_3), 44.68 (C-4), 46.29 (C-3), 52.41 ($COO\underline{C}H_3$), 82.51 (C-2), 126.92, 127.00, 128.10, 128.89, 129.25, 130.89, 141.59, 143.29, 172.75 ($\underline{C}OOCH_3$), 174.91 (C-5). HRMS (ESI): calculated for $C_{19}H_{19}NO_2$ $[M+H]^+$ *m/z* 294.1489, found 294.1484.

Methyl rel-(2R,3S)-5-(4-chlorophenyl)-3-phenyl-3,4-dihydro-2H-pyrrole-2-carboxylate (*trans*-**5l**): Following the general procedure, after purification by column chromatography (SiO_2, petroleum ether: acetone = 20:1), a yellow oil of *trans*-**5l** (0.195 g, 62%) was obtained as an individual diastereoisomer. IR (capillary layer): 1742 (νC=O), 1614 (νC=N), 1597, 1493, 1455 (νC=C), 834, 760, 701 (γC-H) cm^{-1}. ^1H NMR (500.13 MHz, $CDCl_3$) δ (ppm): 3.14 (ddd, 1H, ^2J = 17.4 Hz, ^3J = 6.6 Hz,^4J = 1.7 Hz, H-4a), 3.63 (ddd, 1H, ^2J = 17.4 Hz, ^3J = 9.6 Hz, ^4J = 2.1 Hz, H-4b), 3.79 (s, 3H, $COOCH_3$), 3.91 (dt, 1H, ^3J = 9.6 Hz, ^3J = 6.4 Hz, H-3), 4.95 (dt, 1H, ^3J = 6.1 Hz, ^4J = 1.8 Hz, H-2), 7.22–7.27 (m, 3H, H-2′, H-4′), 7.31-7.34 (m, 2H, H-3′), 7.40–7.42 (m, 2H, H-3″), 7.84–7.85 (m, 2H, H-2″). ^{13}C NMR (125.76 MHz, $CDCl_3$) δ (ppm): 44.64 (C-4), 46.34 (C-3), 52.49 ($COO\underline{C}H_3$), 82.54 (C-2), 126.96*, 127.05* (C-2′ or C-3′), 128.82*, 128.95* (C-3″ or C-4′), 129.42 (C-2″), 132.00 (C-1″), 137.38 (C-4″), 142.91 (C-1′), 172.47 ($\underline{C}OOCH_3$), 173.96 (C-5). HRMS (ESI): calculated for $C_{18}H_{16}ClNO_2$ $[M+H]^+$ *m/z* 314.0942, found 314.0954.

Methyl rel-(2R,3S)-3-(3-fluoro-4-methylphenyl)-5-(2-hydroxy-4,6-dimethoxyphenyl)-3,4-dihydro-2H-pyrrole-2-carboxylate (*trans*-**5m**): Following the general procedure, after purification by column chromatography (SiO_2, petroleum ether: acetone = 8:1), a yellow oil of *trans*-**5m** (0.105 g, 68%) was obtained as an individual diastereoisomer. IR (KBr): 3467 (νOH), 1747 (νC=O), 1615 (νC=N), 1589, 1515, 1434 (νC=C), 820 (γC-H) cm^{-1}. ^1H NMR (500.13 MHz, $CDCl_3$) δ (ppm): 2.24 (d, 3H, ^4J = 1.8 Hz, CH_3), 3.31 (ddd, 1H, ^2J = 18.6 Hz, ^3J = 6.0 Hz, ^4J = 1.3 Hz, H-4a), 3.66 (dt, 1H, ^3J = 9.6 Hz, ^3J = 5.9 Hz, H-3), 3.76 (s, 3H, CH_3O), 3.76 (s, 3H, $COOCH_3$), 3.82 (ddd, 1H, ^2J = 18.5 Hz, ^3J = 9.6 Hz, ^4J = 1.6 Hz, H-4b), 3.82 (s, 3H, CH_3O), 4.77 (dt, 1H, ^3J = 6.0 Hz, ^4J = 1.5 Hz, H-2), 5.91 (d, 1H, ^4J = 2.4 Hz, Ar), 6.16 (d, 1H, ^4J = 2.4 Hz, Ar), 6.88 (dd, 1H, $^3J_{H-F}$ = 11.3 Hz, ^4J = 1.5 Hz, H-2′), 6.90 (dd, 1H, ^3J = 8.0 Hz, ^4J = 1.7 Hz, H-6′), 7.12 (t, 1H, ^3J = 7.9 Hz, H-5′), 15.16 (s, 1H, OH). ^{13}C NMR (125.76 MHz, $CDCl_3$) δ (ppm): 14.20 (d, $^4J_{C-F}$ = 3.4 Hz, CH_3), 44.77 (C-4), 48.17 (C-3), 52.49 (COO$\underline{C}H_3$), 55.33 (CH_3O), 55.42 (CH_3O), 77.67 (C-2), 90.03, 93.94, 100.97, 113.63 (d, $^2J_{C-F}$ = 22.6 Hz, C-2′), 122.35 (d, $^4J_{C-F}$ = 3.2 Hz, C-6′), 123.43 (d, $^2J_{C-F}$ = 17.2 Hz, C-4′), 131.83 (d, $^3J_{C-F}$ = 5.5 Hz, C-5′), 142.96 (d, $^3J_{C-F}$ = 7.0 Hz, C-1′), 161.30, 161.42 (d, $^1J_{C-F}$ = 245.3 Hz, C-3′), 164.25, 166.44, 172.18 ($\underline{C}OOCH_3$), 177.37 (C-5). HRMS (ESI): calculated for $C_{21}H_{22}FNO_5$ $[M+H]^+$ *m/z* 388.1555, found 388.1567.

rel-(2R,3R)-3-(3-Fluoro-4-methylphenyl)-5-(2-hydroxy-4,6-dimethoxyphenyl)-3,4-dihydro-2H-pyrrole-2-carboxamide (*cis*-**6m**): Following the general procedure, after purification by column chromatography (SiO_2, petroleum ether: acetone = 1:1), yellow crystals of *cis*-**6m** (0.083 g, 56%) were obtained as an individual diastereoisomer, mp 191–193 °C (with decomposition). IR (KBr): 3429, 3310 (νNH_2), 3338 (νOH), 1668 (νC=O), 1614 (νC=N), 1597, 1515, 1443 (νC=C), 814 (γC-H) cm^{-1}. ^1H NMR (500.13 MHz, DMSO-d_6) δ (ppm): 2.17 (s, 3H, CH_3), 3.39 (dd, 1H, ^2J = 17.8 Hz, ^3J = 8.3, Hz, H-4a), 3.52 (dd, 1H, ^2J = 17.8 Hz, ^3J = 8.7 Hz, H-4b), 3.77 (s, 3H, CH_3O), 3.78 (s, 3H, CH_3O), 3.82 (q, 1H, ^3J = 8.5 Hz, H-3), 4.88 (d, 1H, ^3J = 8.4 Hz, H-2), 5.99 (d, 1H, ^4J = 2.4 Hz, Ar), 6.04 (d, 1H, ^4J = 2.4 Hz, Ar), 6.86 (s, 1H, $CONH_2$), 6.93–6.96 (m, 2H, H-2′, H-6′), 7.15 (t, 1H, ^3J = 8.2 Hz, H-5′), 7.31 (s, 1H, $CONH_2$), 15.34 (s, 1H, OH). ^{13}C NMR (125.76 MHz, DMSO-d_6) δ (ppm): 13.87 (d, $^4J_{C-F}$ = 3.0 Hz, CH_3), 44.23 (C-4), 44.73 (C-3), 55.39 (CH_3O), 55.73 (CH_3O), 73.34 (C-2), 89.49, 94.21, 100.68, 114.54 (d, $^2J_{C-F}$ = 22.1 Hz, C-2′), 122.25 (d, $^2J_{C-F}$ = 16.8 Hz, C-4′), 124.07 (d, $^4J_{C-F}$ = 2.4 Hz, C-6′), 131.08 (d, $^3J_{C-F}$ = 5.7 Hz, C-5′), 139.78 (d, $^3J_{C-F}$ = 7.0 Hz, C-1′), 160.29 (d, $^1J_{C-F}$ = 241.7 Hz, C-3′), 161.34, 164.05, 166.90, 170.46 ($CONH_2$), 177.69 (C-5). HRMS (ESI): calculated for $C_{20}H_{21}FN_2O_4$ $[M+H]^+$ *m/z* 373.1558, found 373.1561.

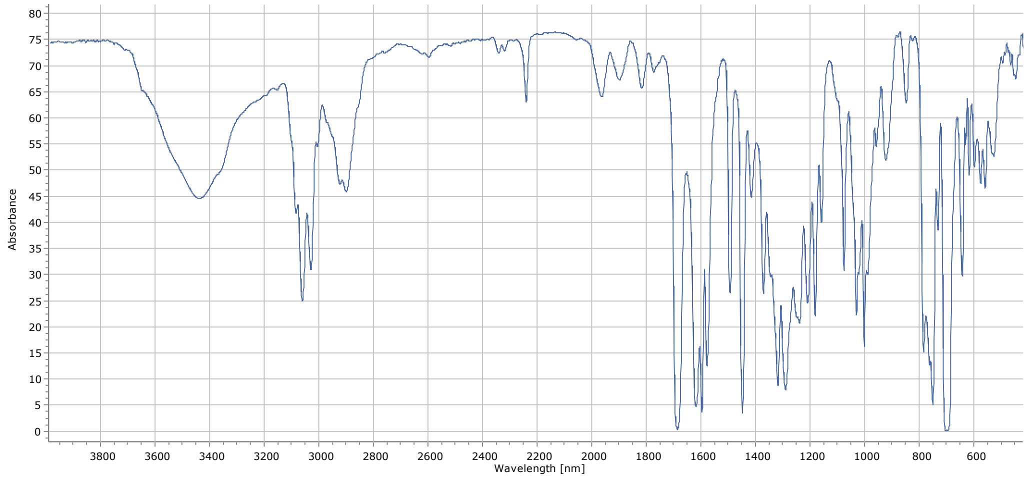

Figure S1: FT-IR spectrum of compound **3a** as a diastereoisomeric mixture (KBr).

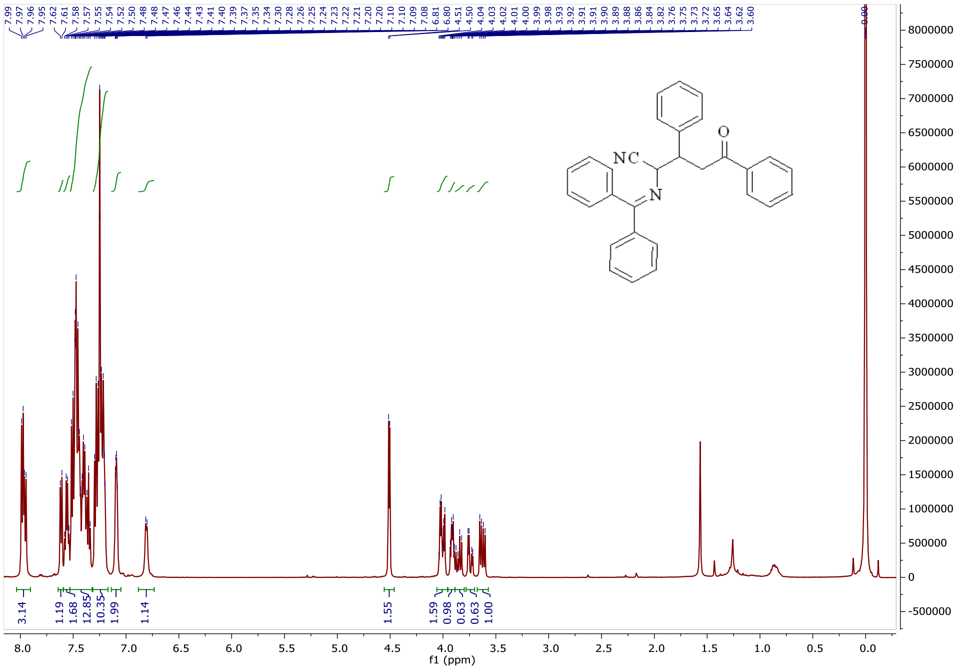

Figure S2: ^{1}H-NMR spectrum of compound **3a** as a diastereoisomeric mixture ($CDCl_3$).

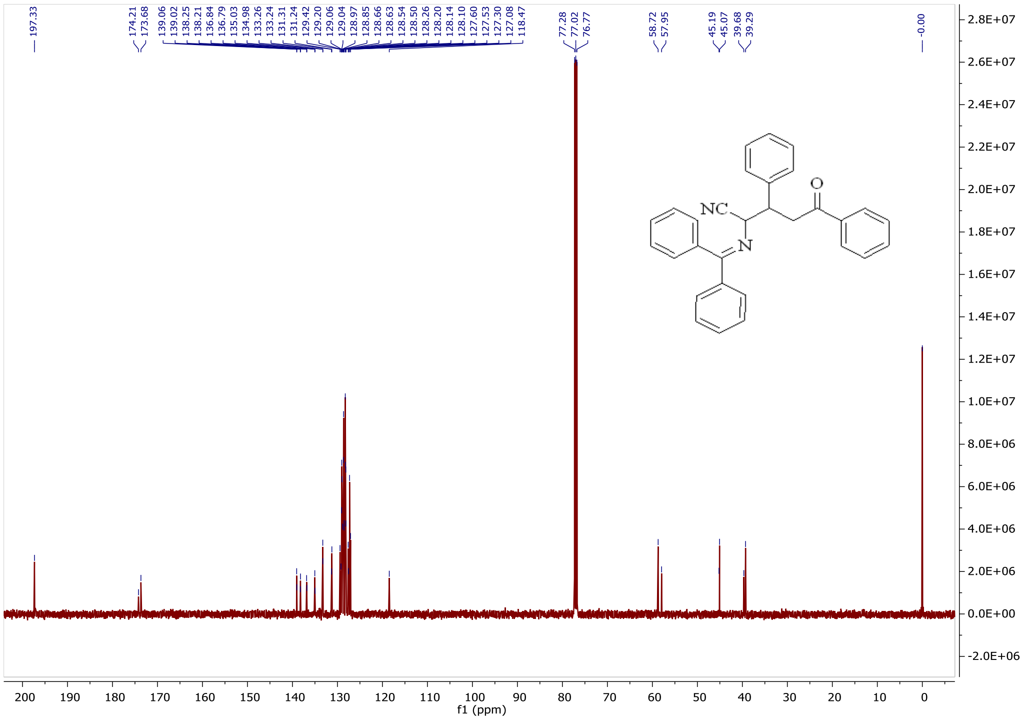

Figure S3: ^{13}C NMR spectrum of compound **3a** as a diastereoisomeric mixture ($CDCl_3$).

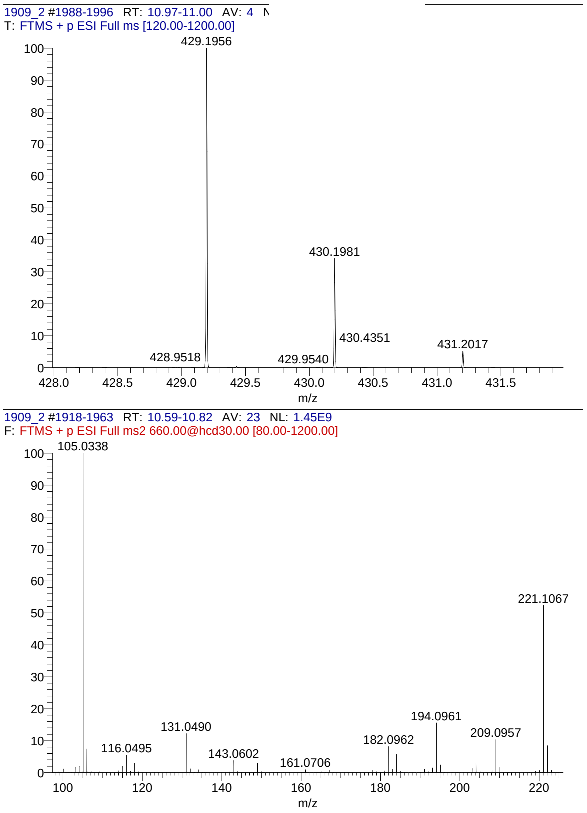

Figure S4: HRMS ESI and HRMS ESI-MS/MS spectrum of compound **3a** as a diastereoisomeric mixture.

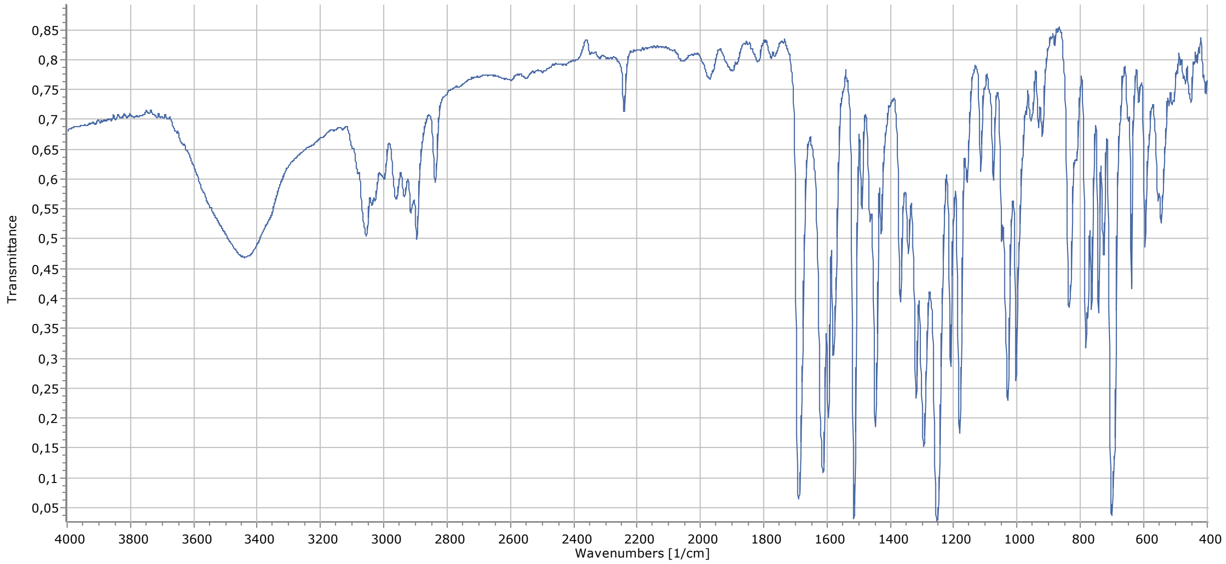

Figure S5: FT-IR spectrum of compound **3b** as an individual diastereoisomer (KBr).

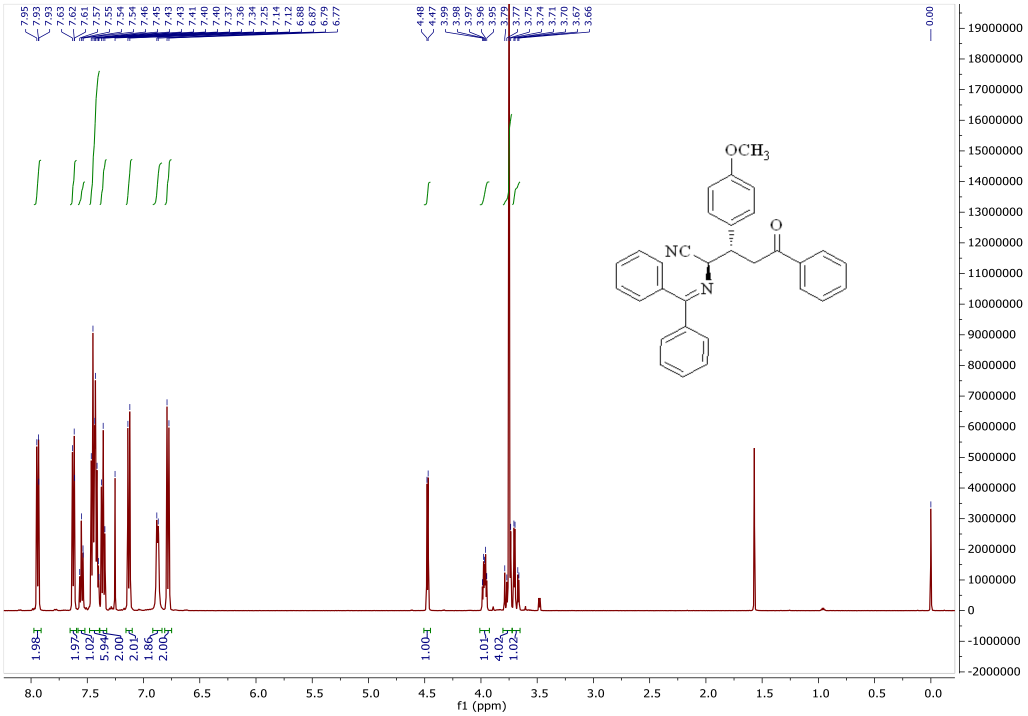

Figure S6: ^{1}H-NMR spectrum of compound **3b** as an individual diastereoisomer ($CDCl_3$).

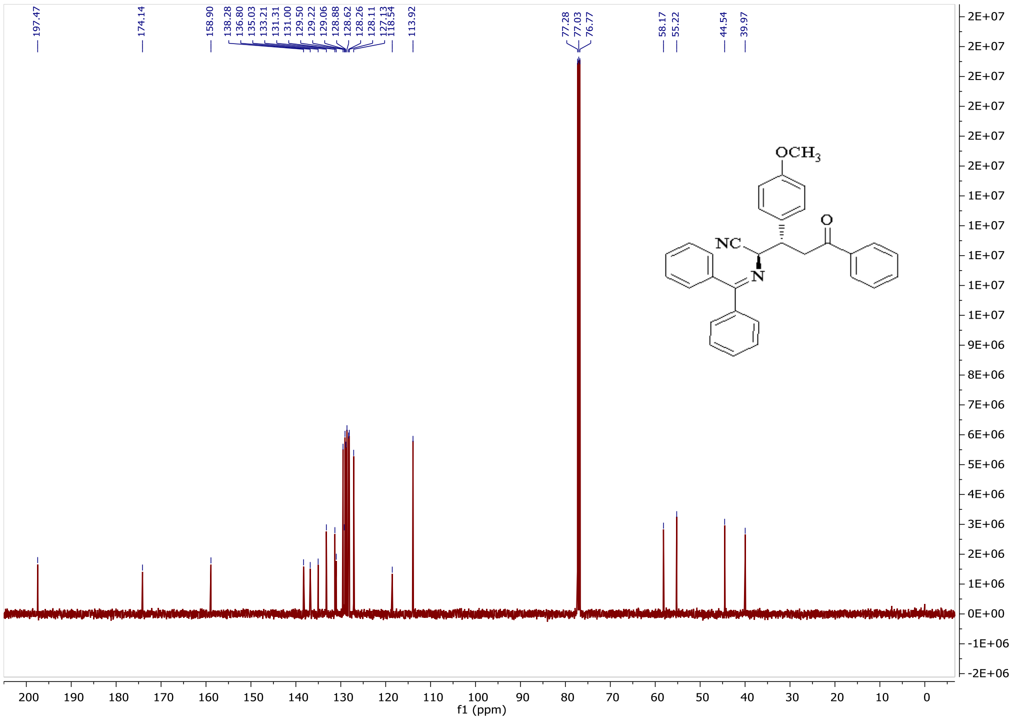

Figure S7: ^{13}C NMR spectrum of compound **3b** as an individual diastereoisomer ($CDCl_3$).

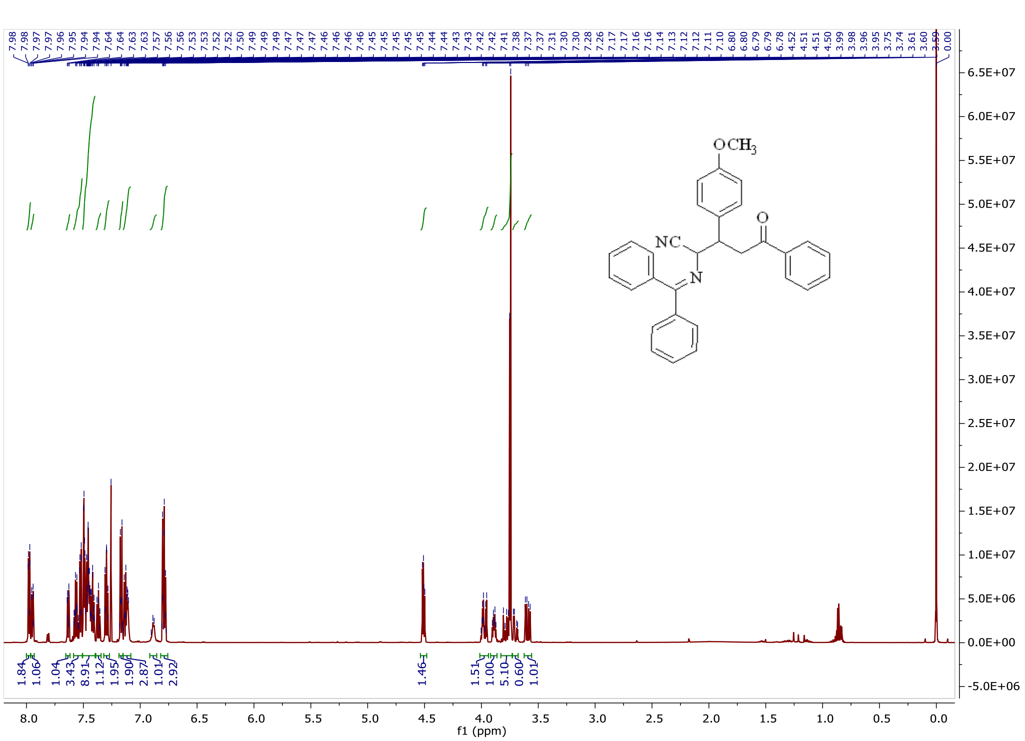

Figure S8: ^{1}H-NMR spectrum of compound **3b** as a diastereoisomeric mixture ($CDCl_3$).

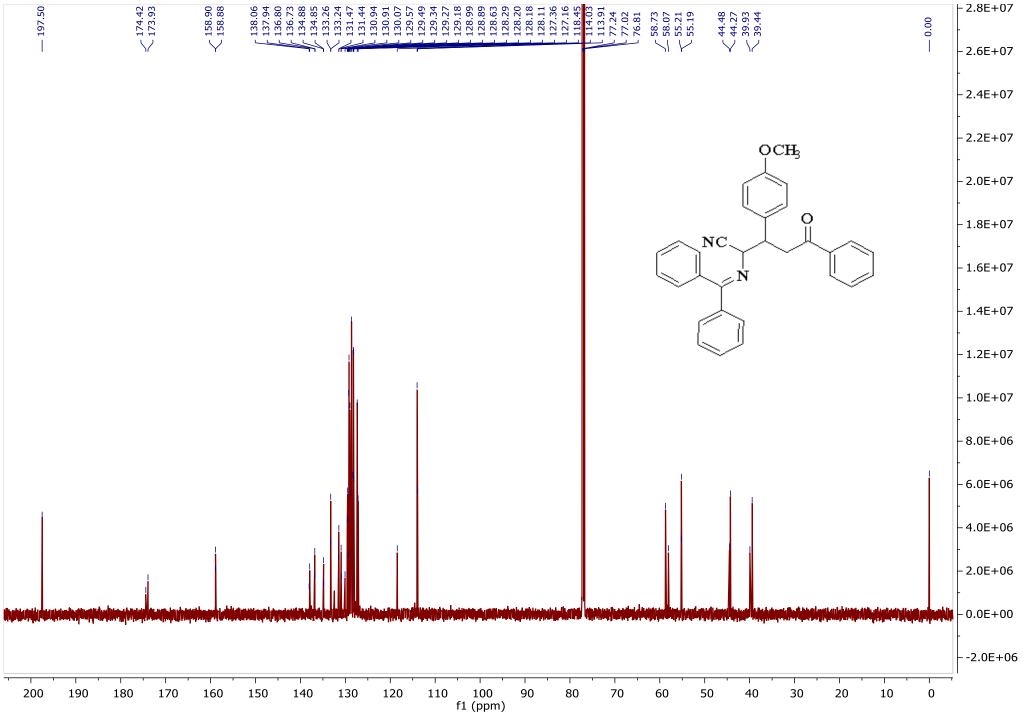

Figure S9: ^{13}C NMR spectrum of compound **3b** as a diastereoisomeric mixture ($CDCl_3$).

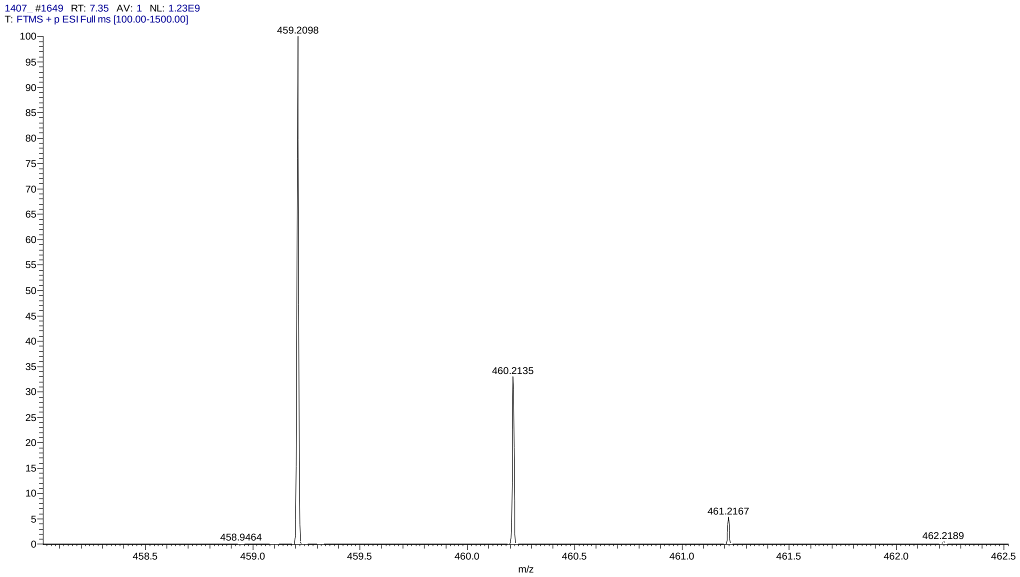

Figure S10: HRMS ESI spectrum of compound **3b** as an individual diastereoisomer.

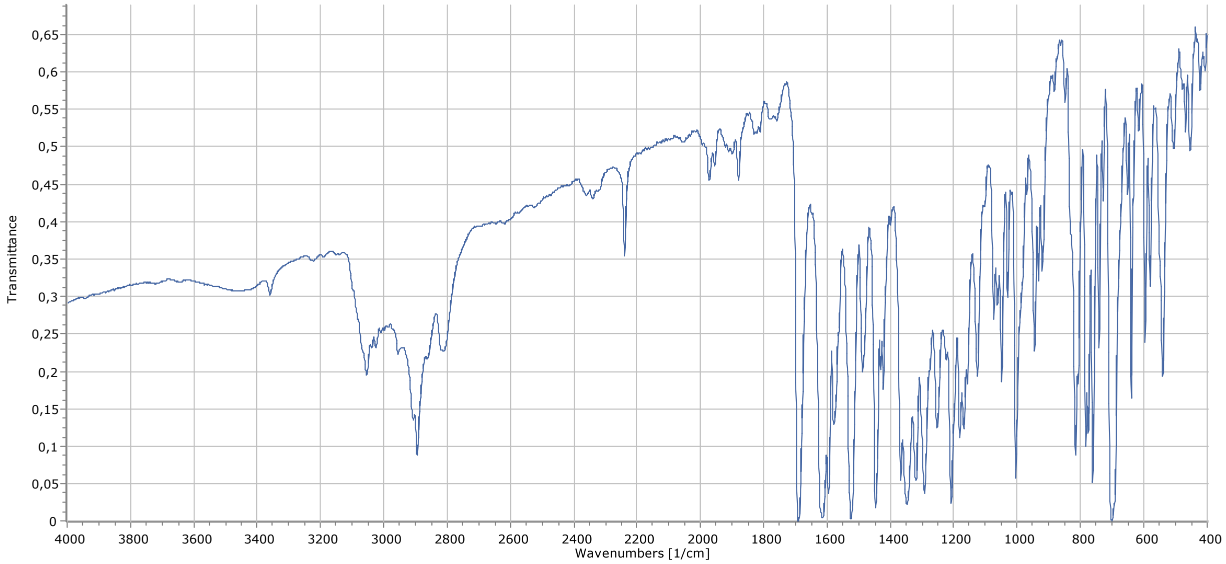

Figure S11: FT-IR spectrum of compound **3c** as an individual diastereoisomer (KBr).

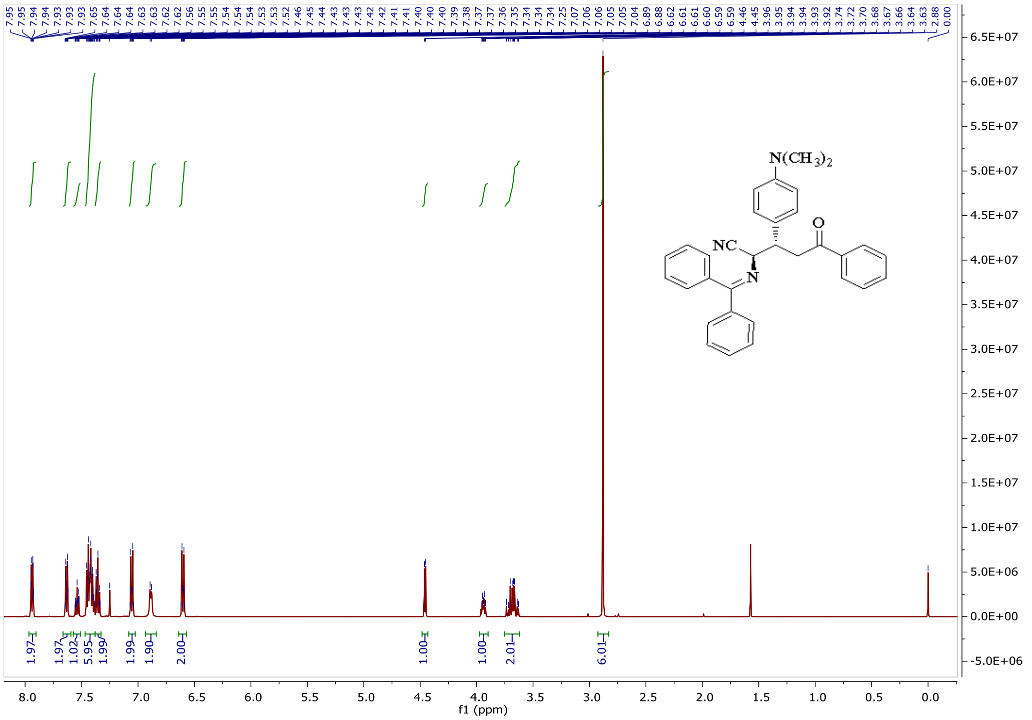

Figure S12: ^{1}H-NMR spectrum of compound **3c** as an individual diastereoisomer ($CDCl_3$).

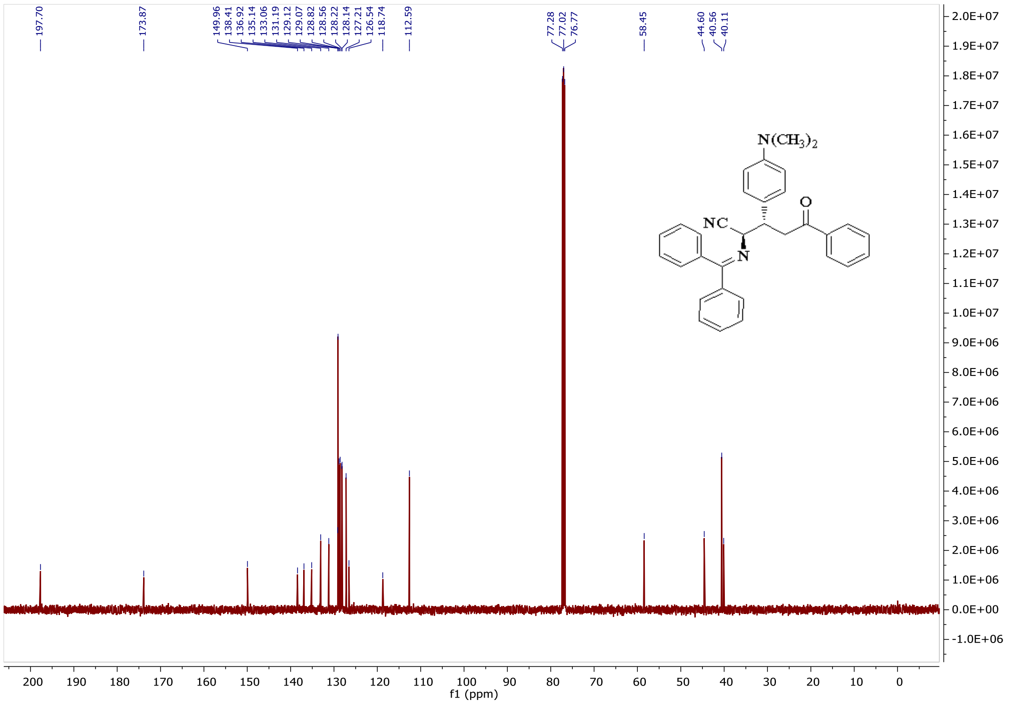

Figure S13: ^{13}C NMR spectrum of compound **3c** as an individual diastereoisomer ($CDCl_3$).

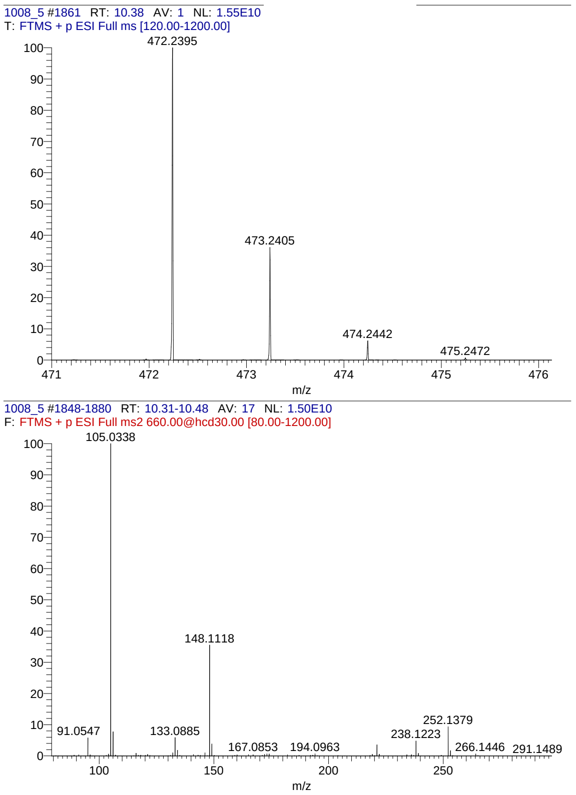

Figure S14: HRMS ESI and HRMS ESI-MS/MS spectrum of compound **3c** as an individual diastereoisomer.

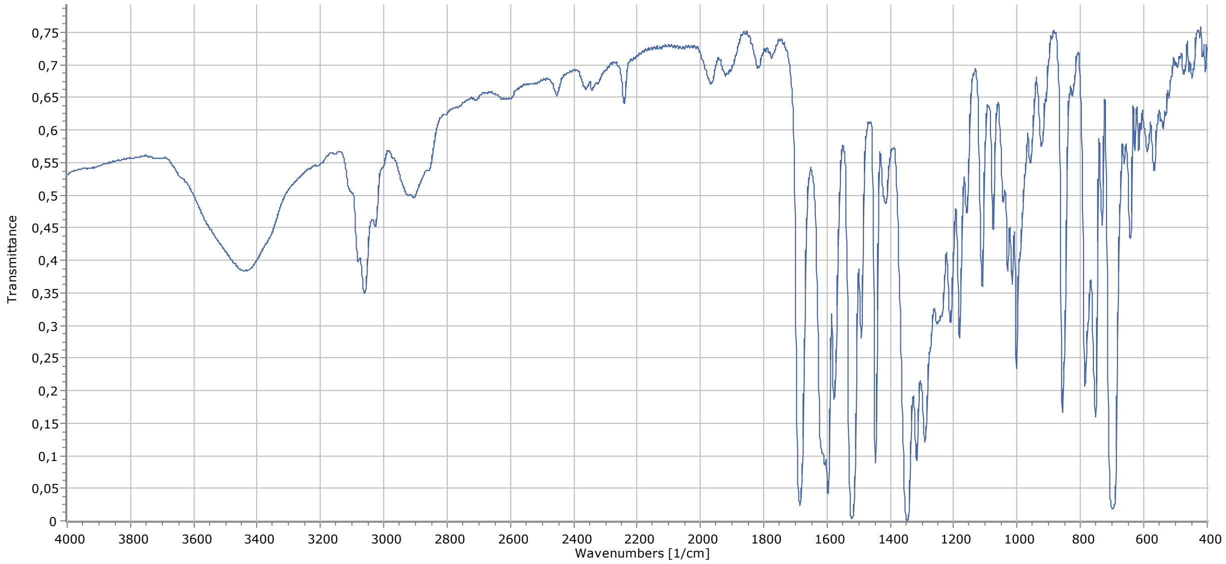

Figure S15: FT-IR spectrum of compound **3d** as a diastereoisomeric mixture (KBr).

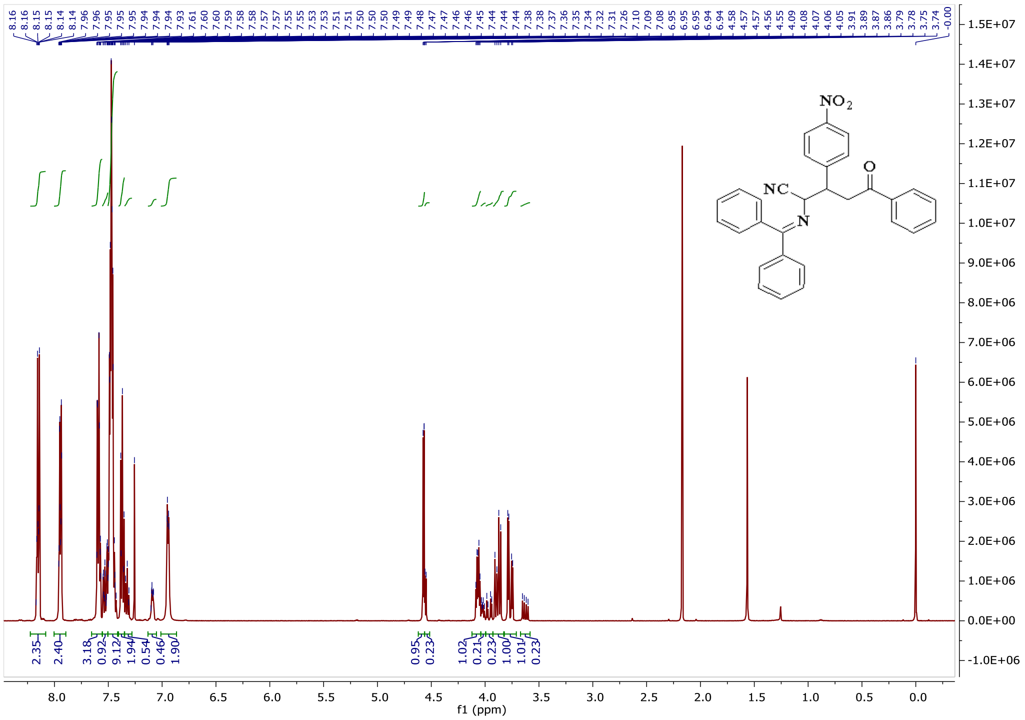

Figure S16: ^{1}H-NMR spectrum of compound **3d** as a diastereoisomeric mixture ($CDCl_3$).

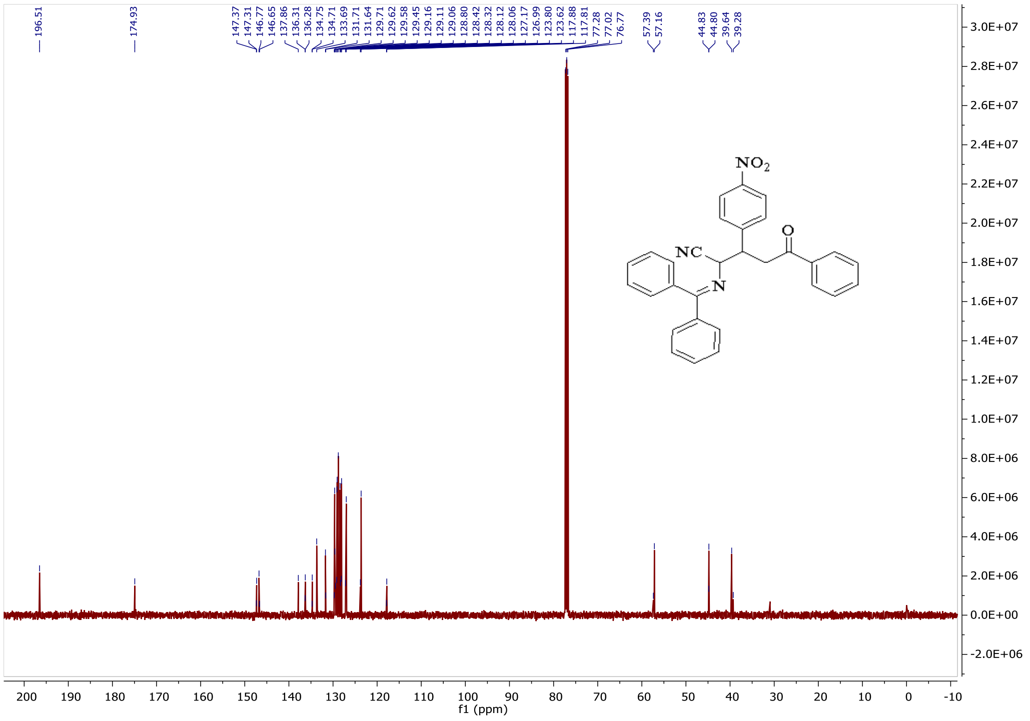

Figure S17: ^{13}C NMR spectrum of compound **3d** as a diastereoisomeric mixture ($CDCl_3$).

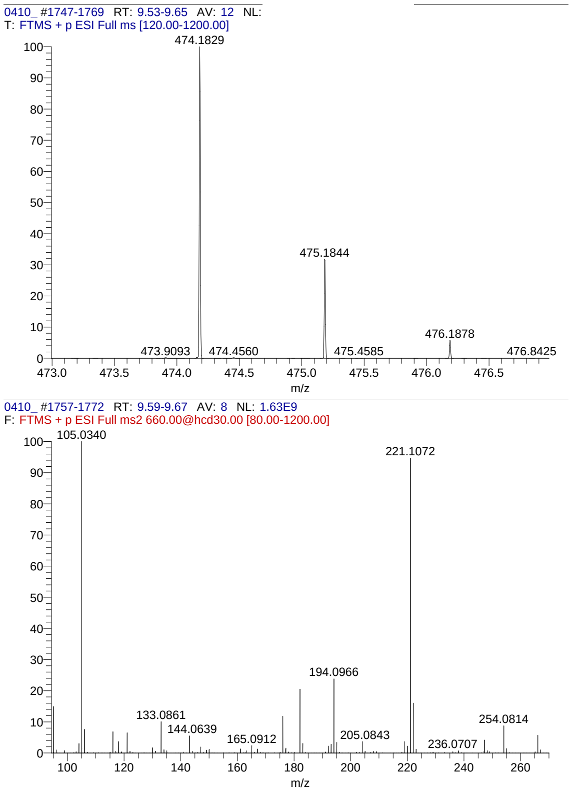

Figure S18: HRMS ESI and HRMS ESI-MS/MS spectrum of compound **3d** as a diastereoisomeric mixture.

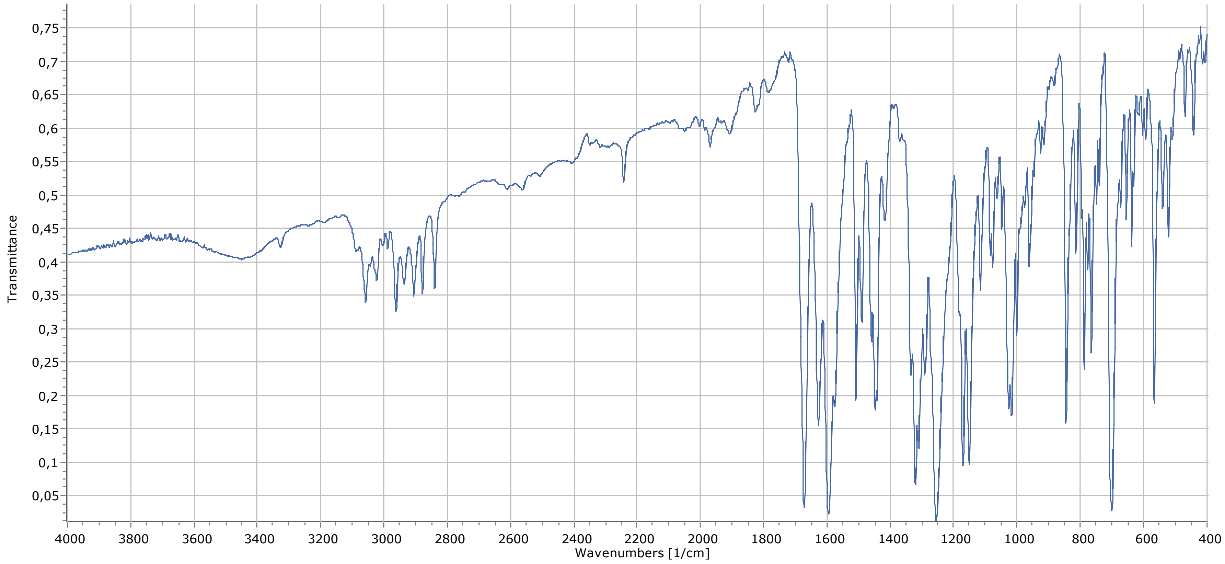

Figure S19: FT-IR spectrum of compound **3e** as an individual diastereoisomer (KBr).

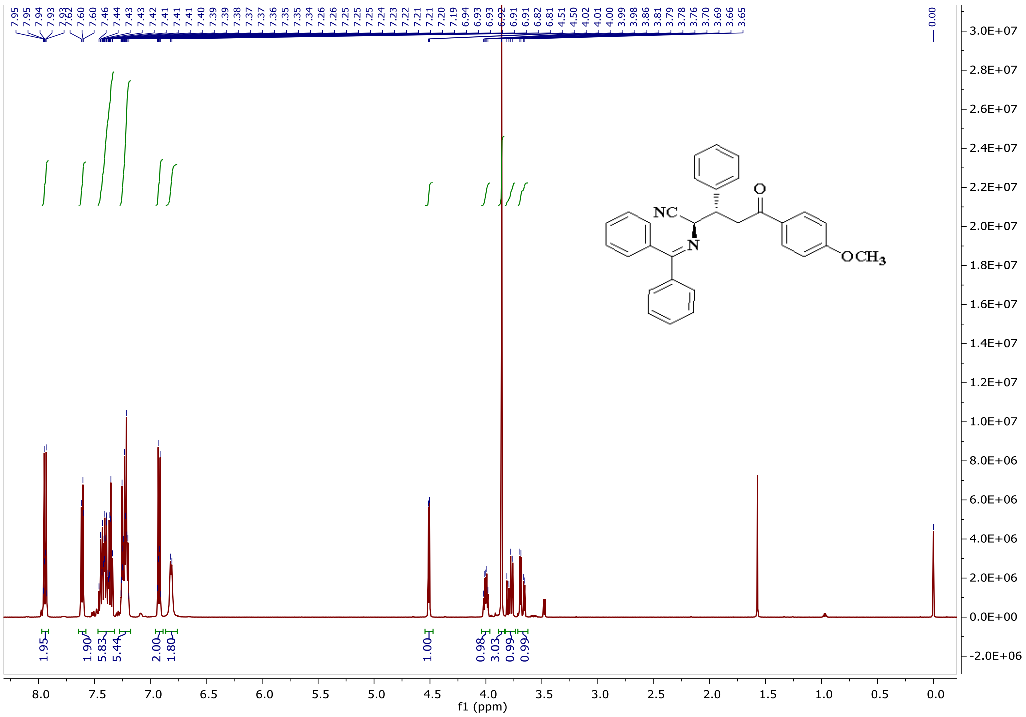

Figure S20: ^{1}H-NMR spectrum of compound **3e** as an individual diastereoisomer ($CDCl_3$).

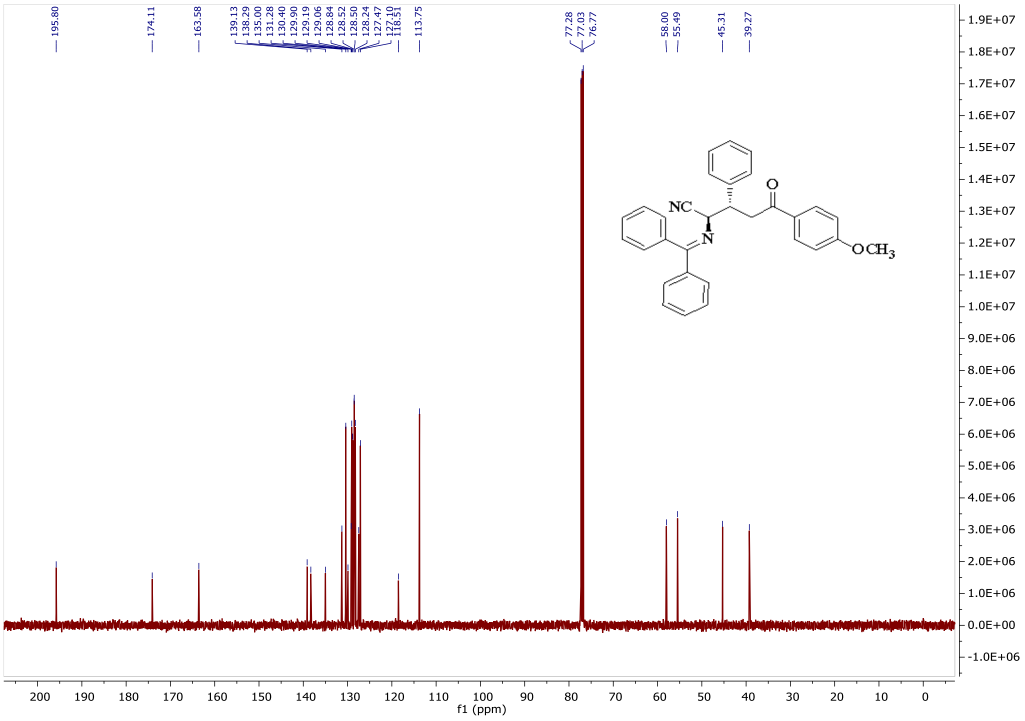

Figure S21: ^{13}C NMR spectrum of compound **3e** as an individual diastereoisomer ($CDCl_3$).

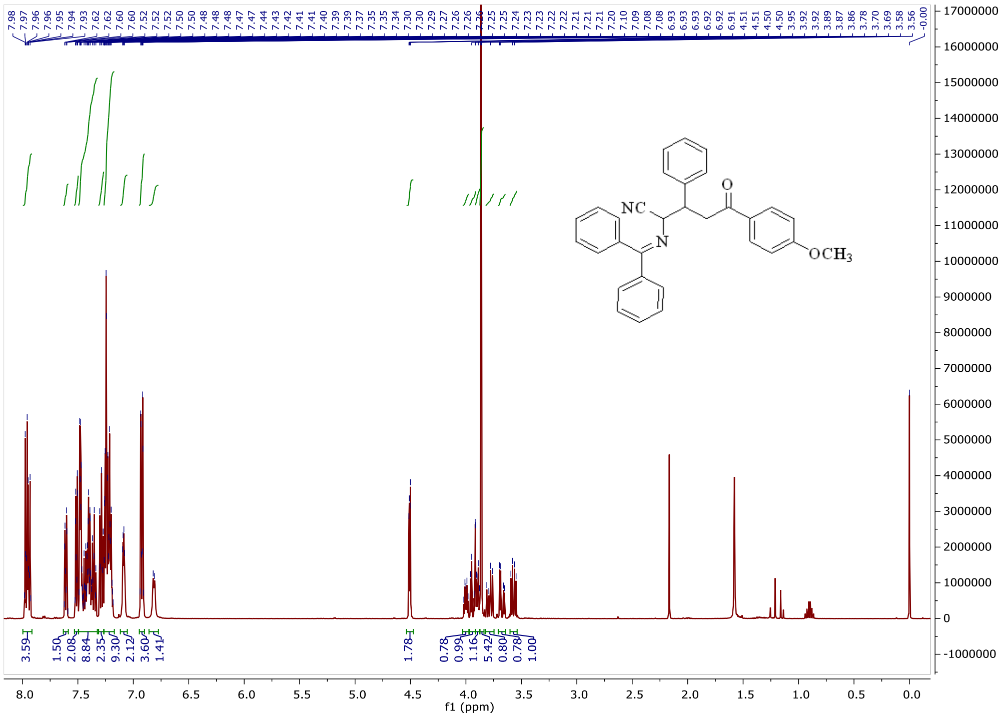

Figure S22: ^{1}H-NMR spectrum of compound **3e** as a diastereoisomeric mixture ($CDCl_3$).

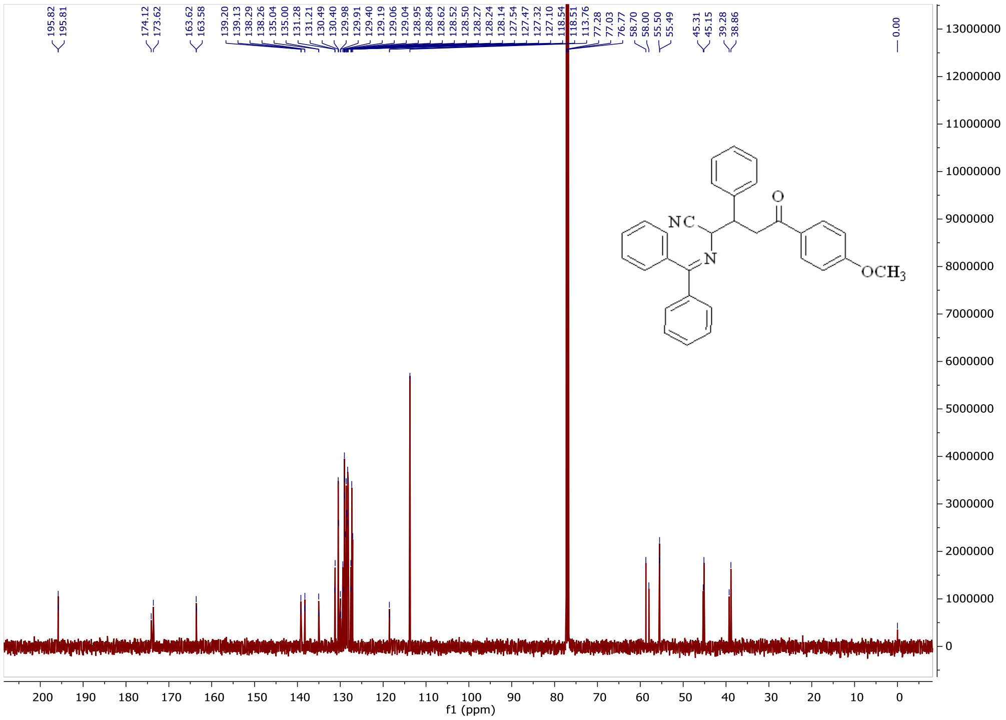

Figure S23: ^{13}C NMR spectrum of compound **3e** as a diastereoisomeric mixture ($CDCl_3$).

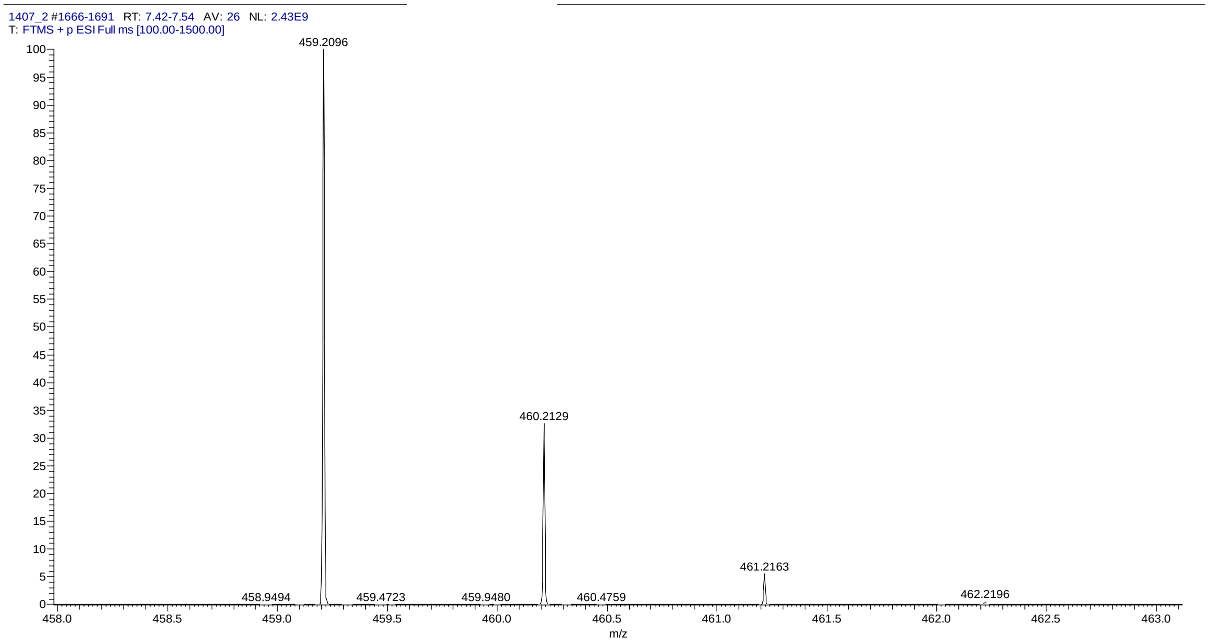

Figure S24: HRMS ESI spectrum of compound **3e** as an individual diastereoisomer.

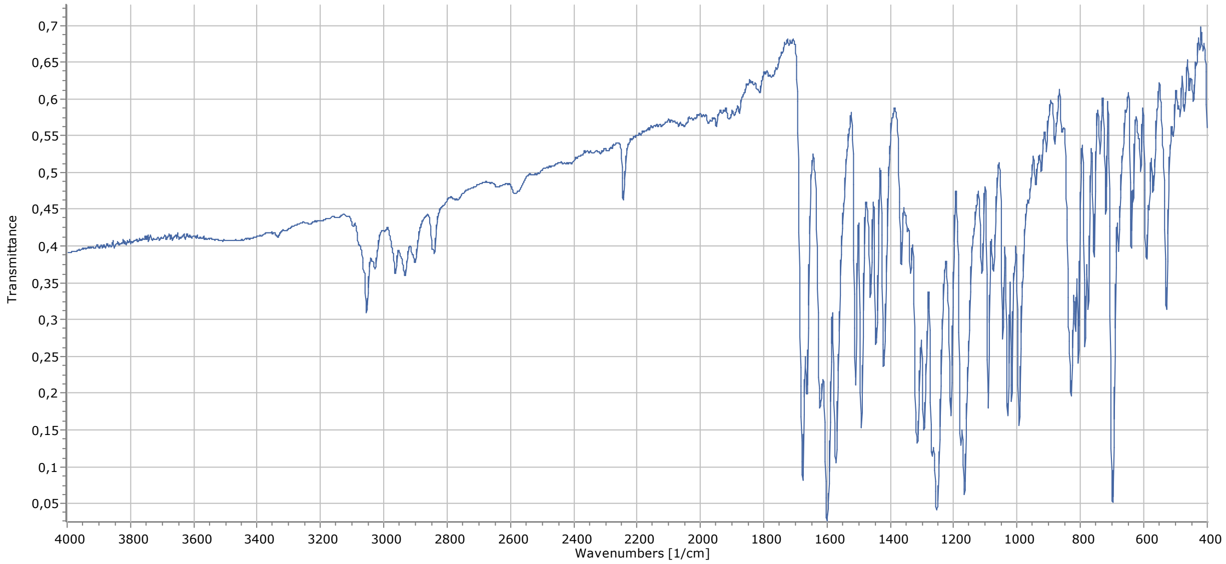

Figure S25: FT-IR spectrum of compound **3f** as an individual diastereoisomer (KBr).

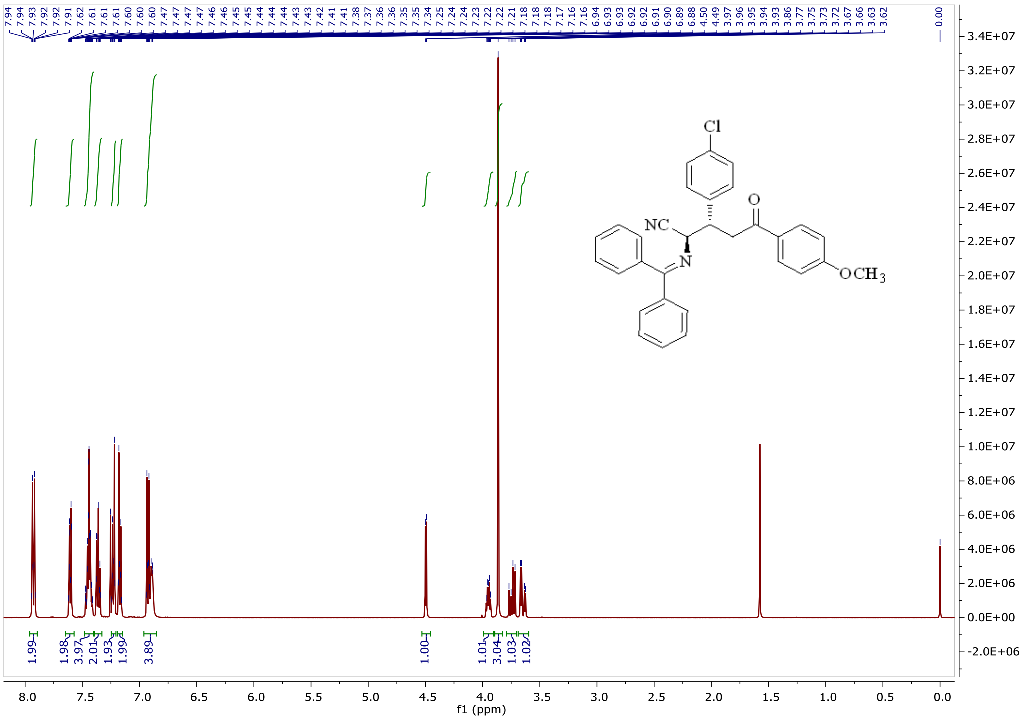

Figure S26: ^{1}H-NMR spectrum of compound **3f** as an individual diastereoisomer ($CDCl_3$).

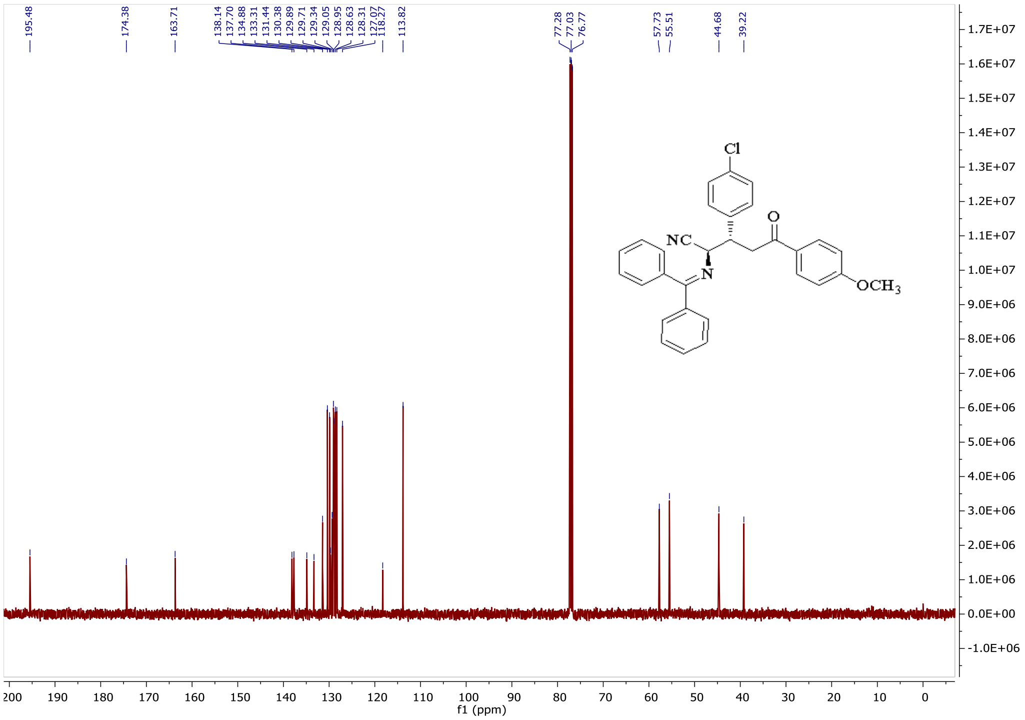

Figure S27: ^{13}C NMR spectrum of compound **3f** as an individual diastereoisomer ($CDCl_3$).

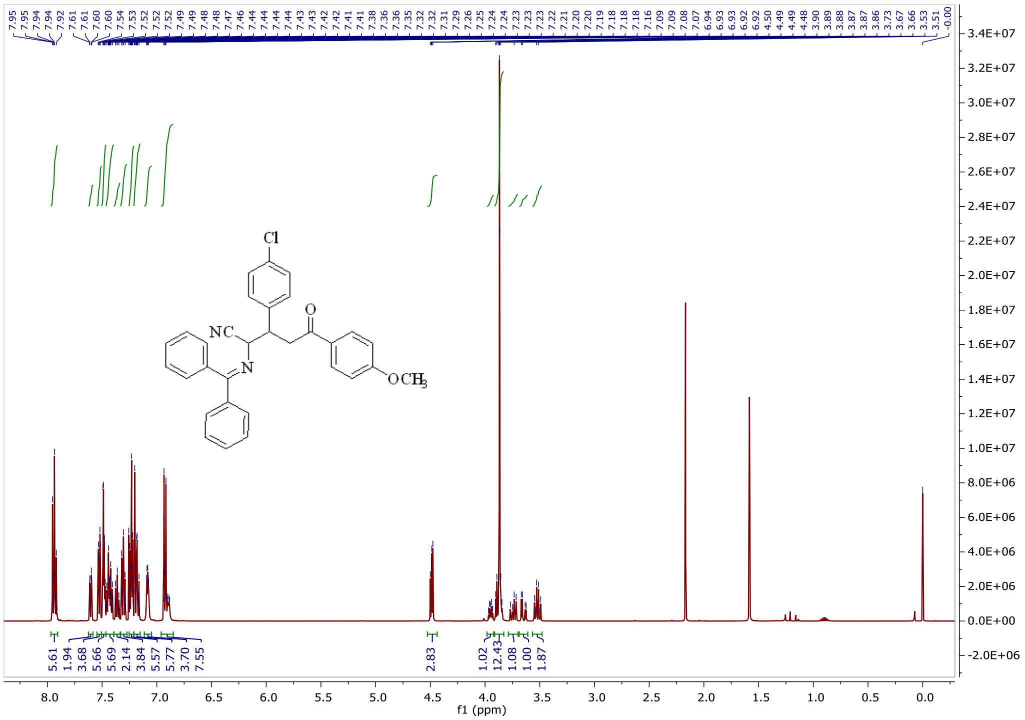

Figure S28: ^{1}H-NMR spectrum of compound **3f** as a diastereoisomeric mixture ($CDCl_3$).

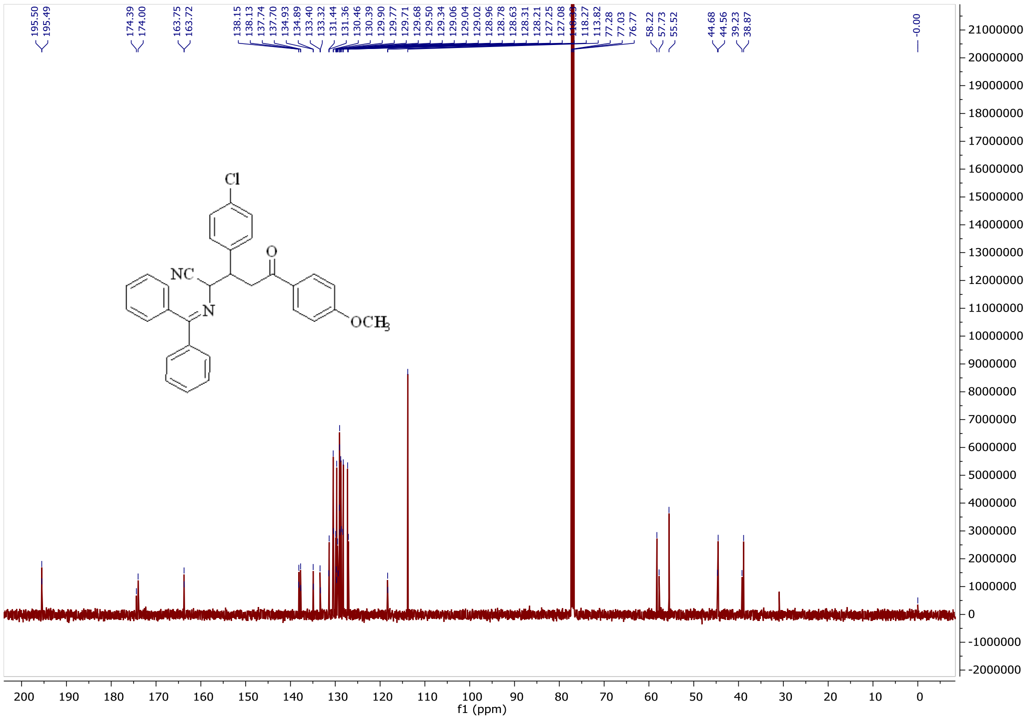

Figure S29: ^{13}C NMR spectrum of compound **3f** as a diastereoisomeric mixture ($CDCl_3$).

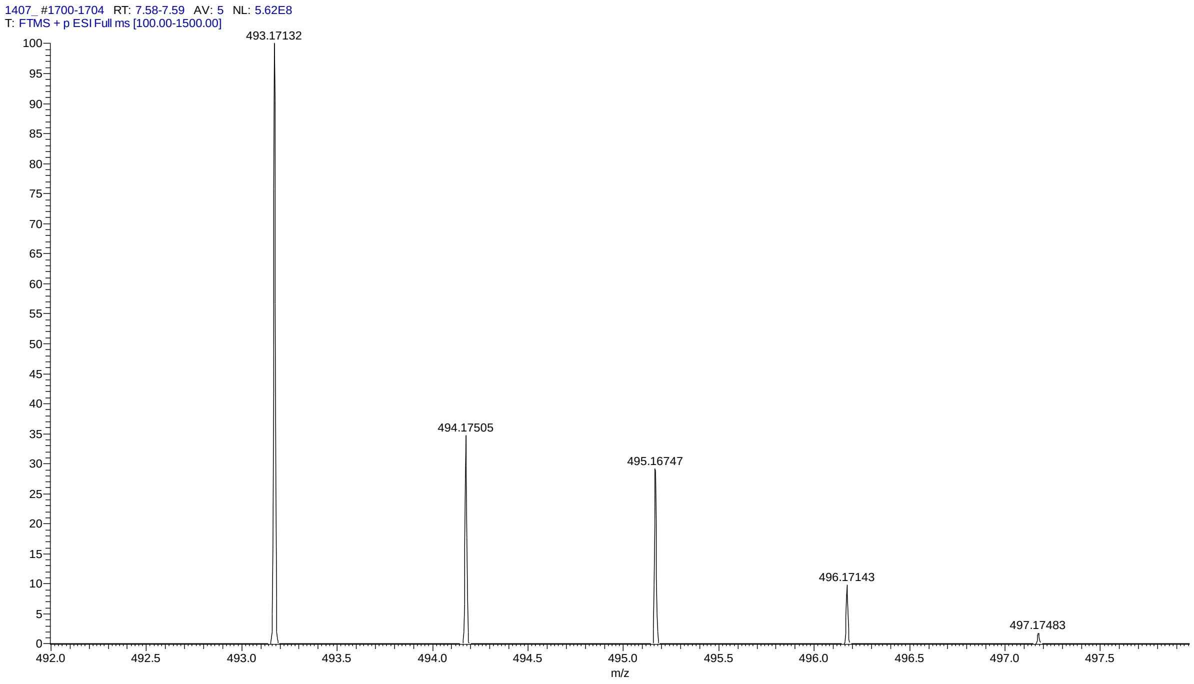

Figure S30: HRMS ESI spectrum of compound **3f** as an individual diastereoisomer.

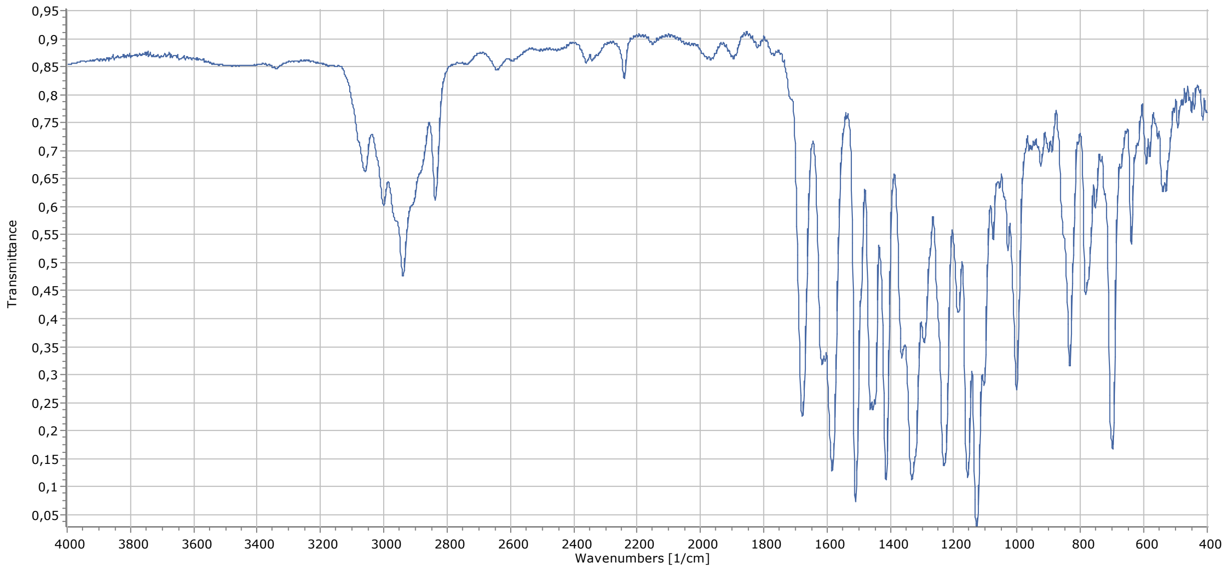

Figure S31: FT-IR spectrum of compound **3g** as a diastereoisomeric mixture (KBr).

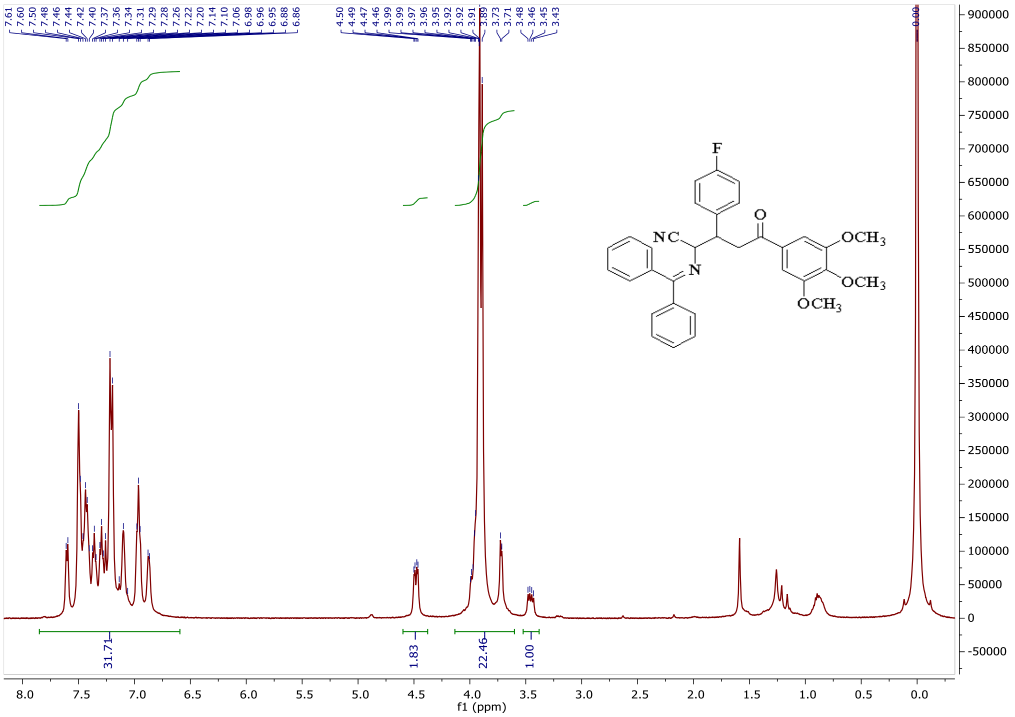

Figure S32: ^{1}H-NMR spectrum of compound **3g** as a diastereoisomeric mixture ($CDCl_3$).

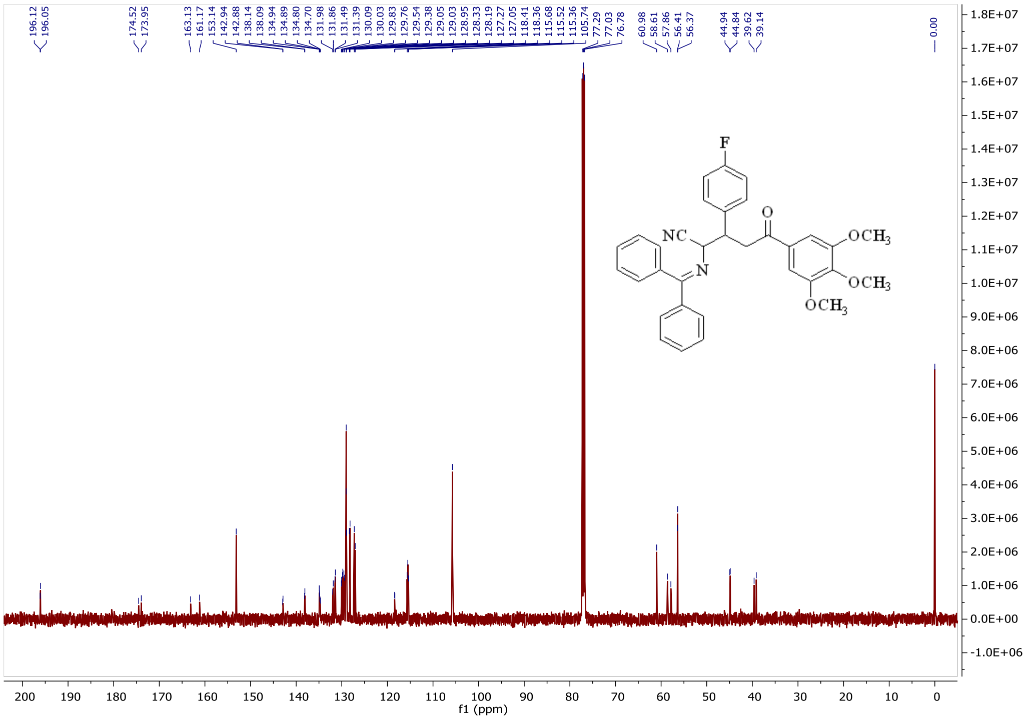

Figure S33: ^{13}C NMR spectrum of compound **3g** as a diastereoisomeric mixture ($CDCl_3$).

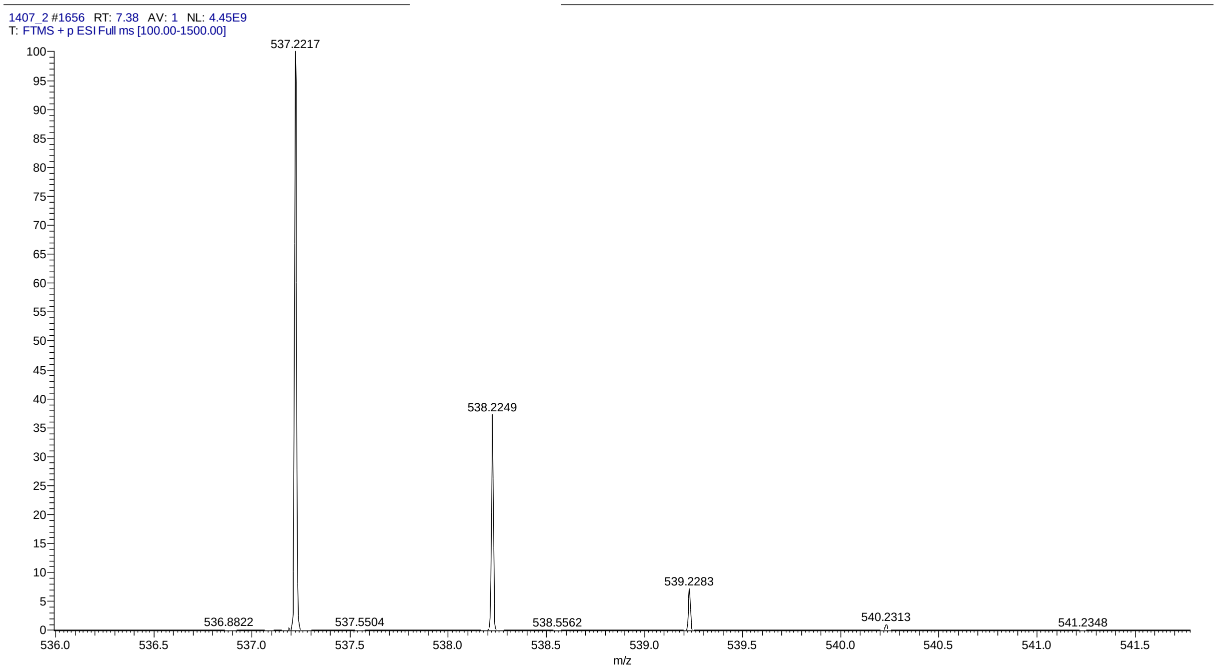

Figure S34: HRMS ESI spectrum of compound **3g** as a diastereoisomeric mixture.

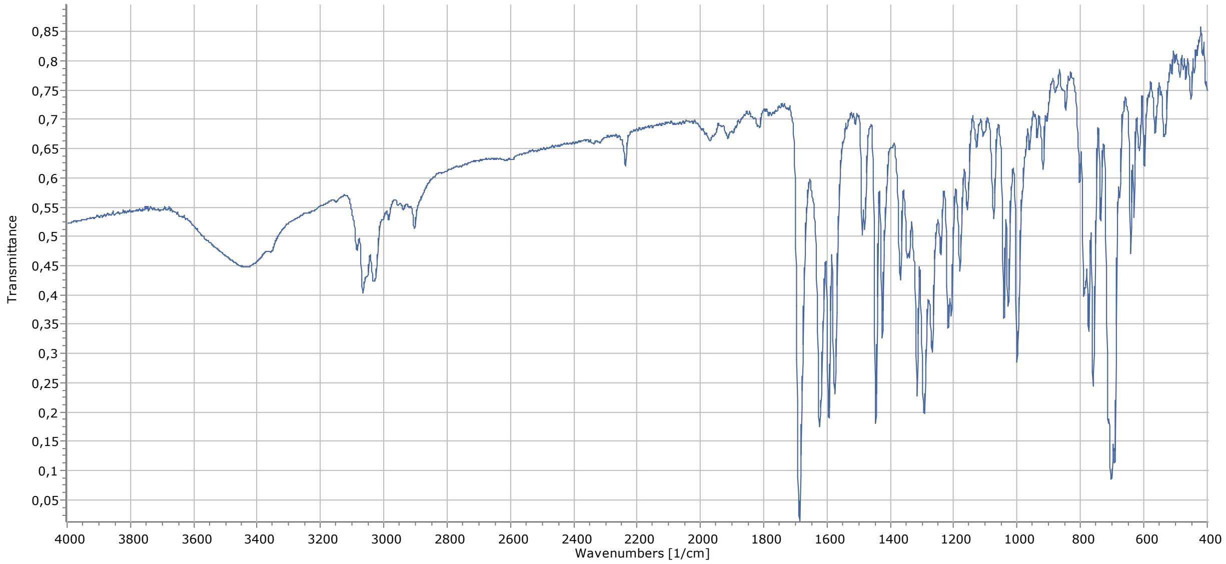

Figure S35: FT-IR spectrum of compound **3h** as an individual diastereoisomer (KBr).

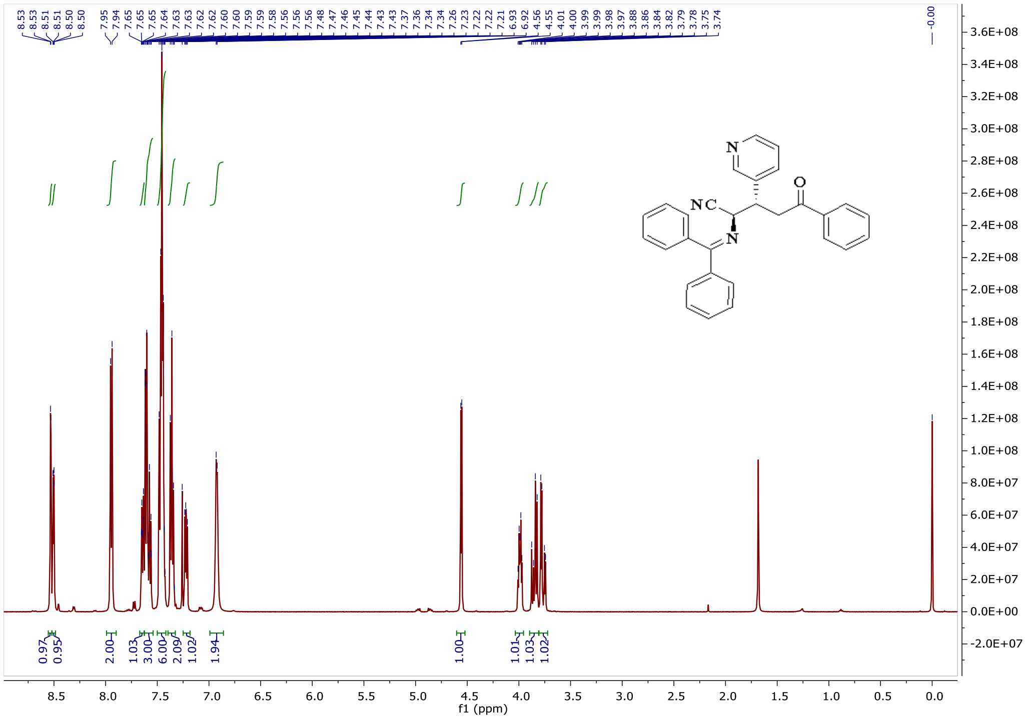

Figure S36: ^{1}H-NMR spectrum of compound **3h** as an individual diastereoisomer ($CDCl_3$).

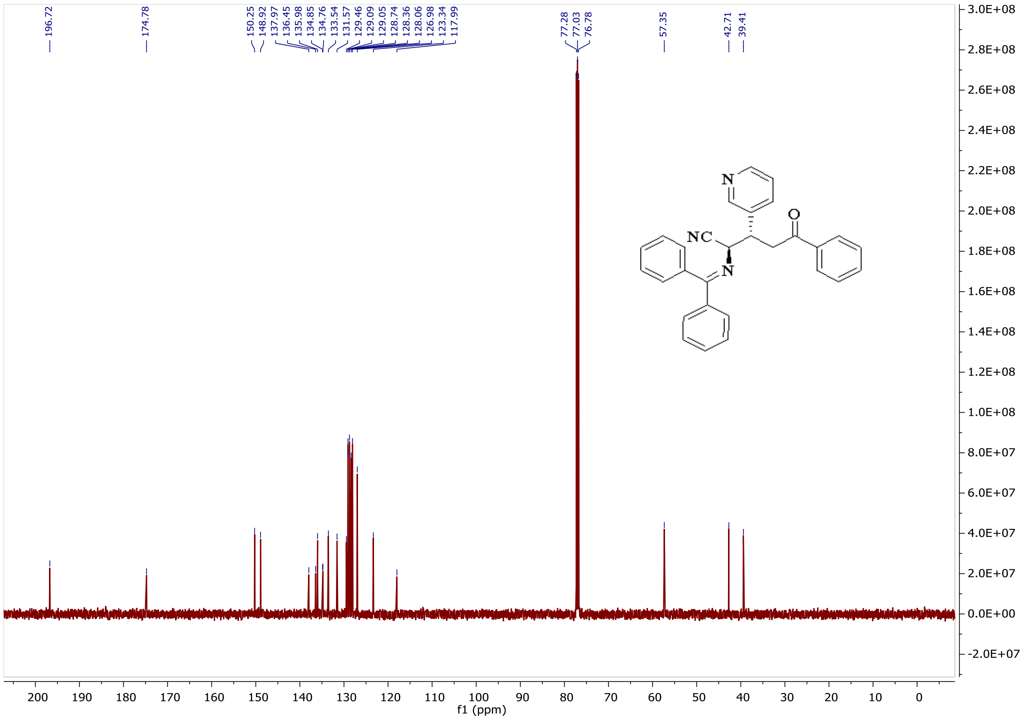

Figure S37: ^{13}C NMR spectrum of compound **3h** as an individual diastereoisomer ($CDCl_3$).

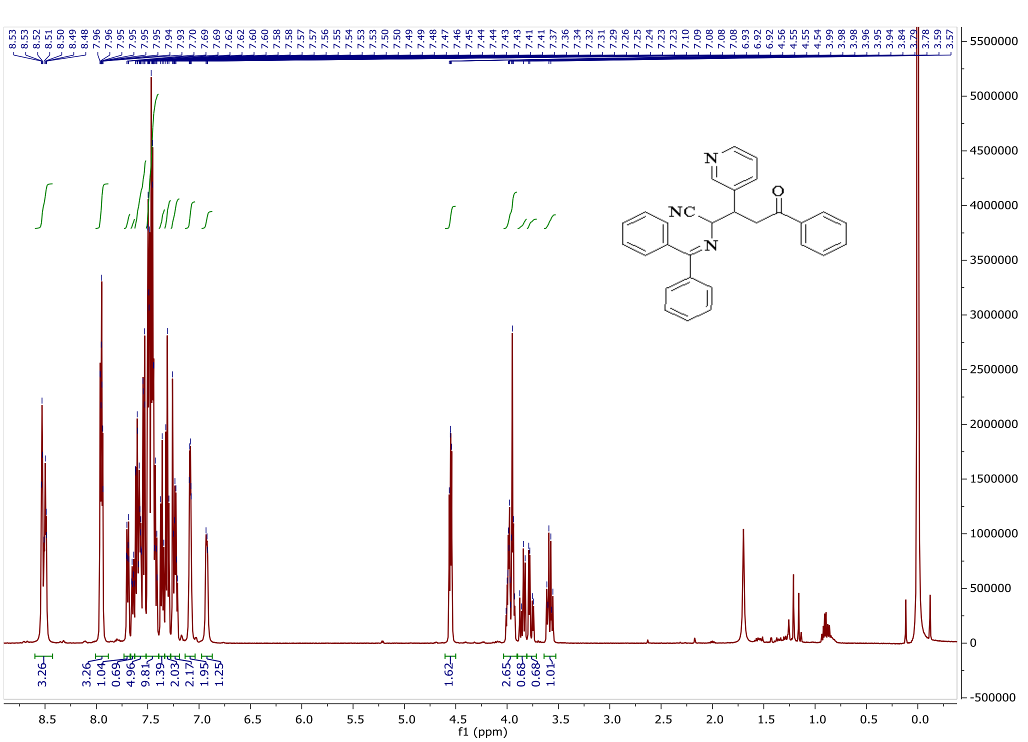

Figure S38: ^{1}H-NMR spectrum of compound **3h** as a diastereoisomeric mixture ($CDCl_3$).

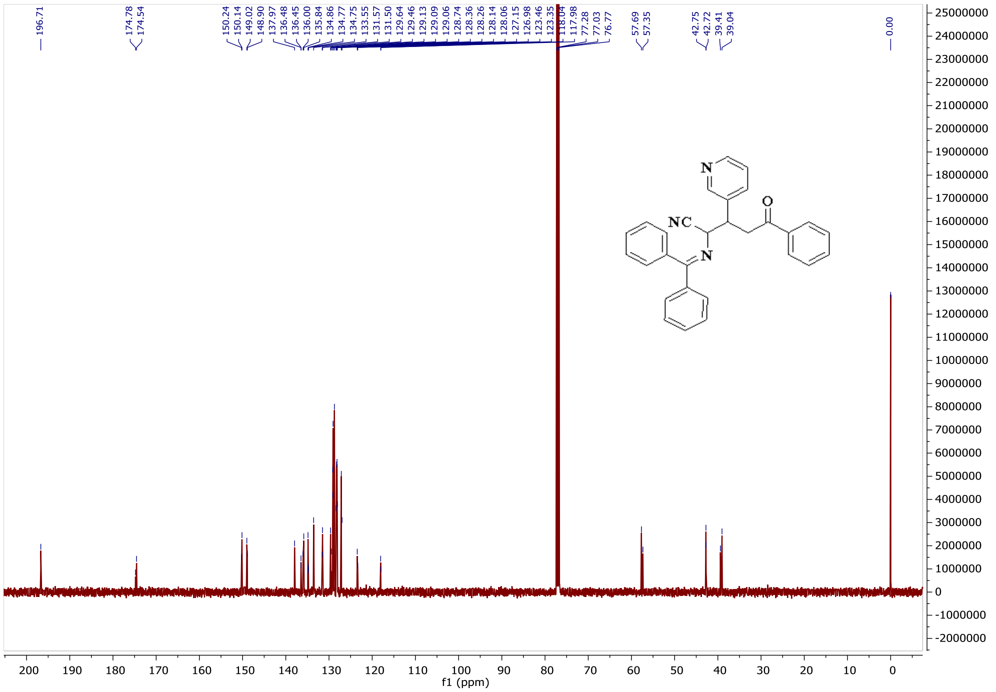

Figure S39: ^{13}C NMR spectrum of compound **3h** as a diastereoisomeric mixture ($CDCl_3$).

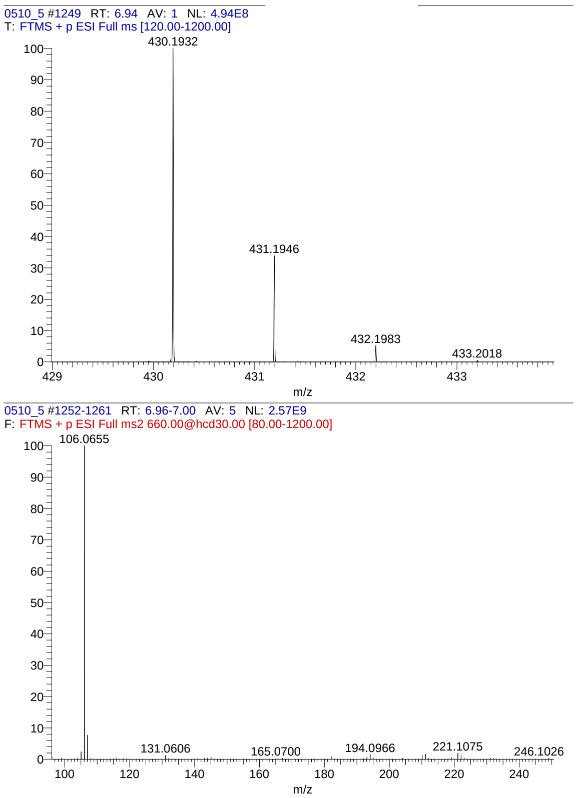

Figure S40: HRMS ESI and HRMS ESI-MS/MS spectrum of compound **3h** as an individual diastereoisomer.

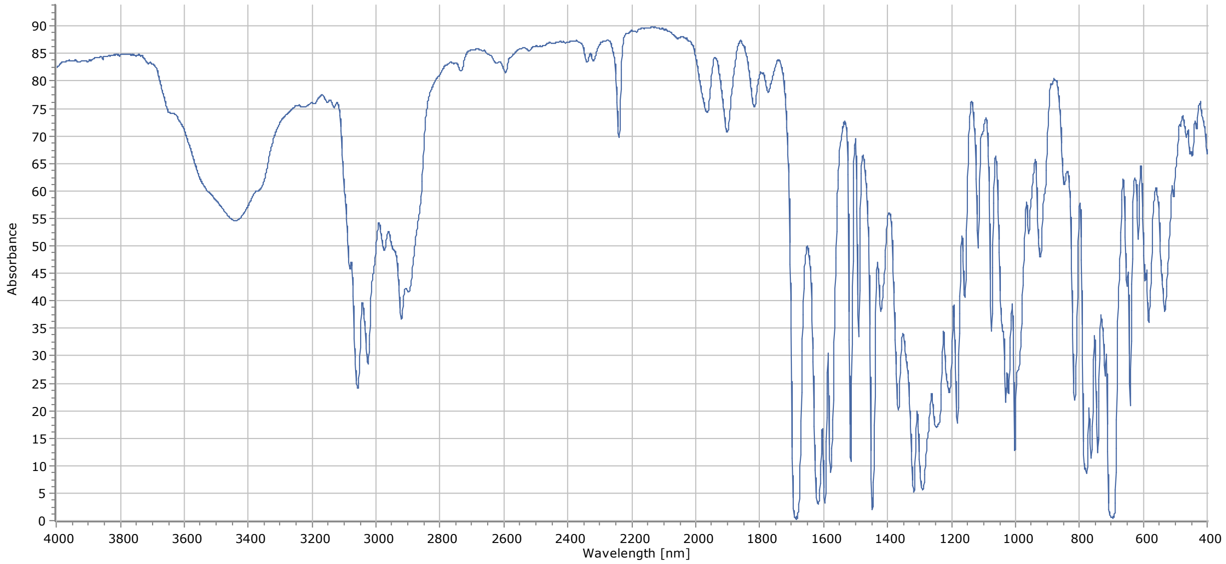

Figure S41: FT-IR spectrum of compound **3i** as a diastereoisomeric mixture (KBr).

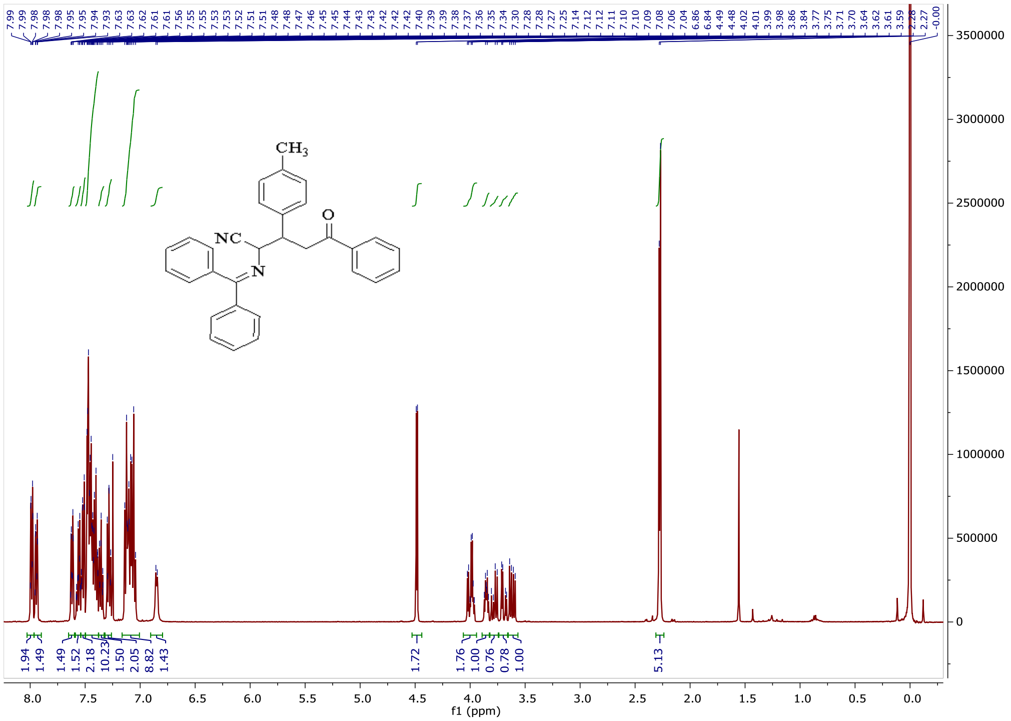

Figure S42: ^{1}H-NMR spectrum of compound **3i** as a diastereoisomeric mixture ($CDCl_3$).

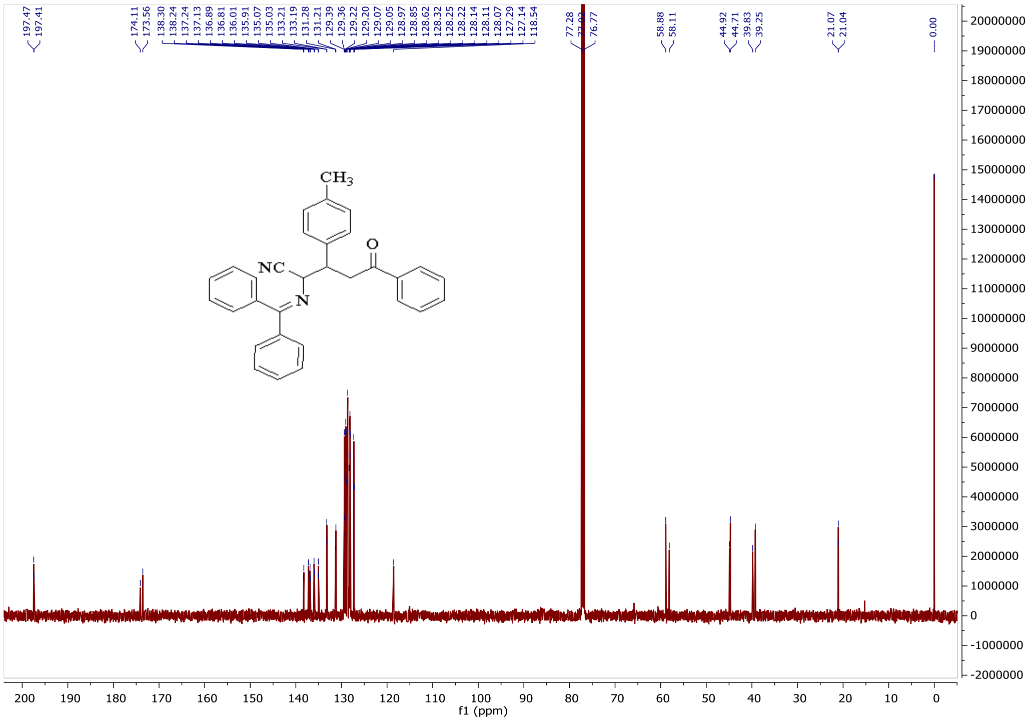

Figure S43: ^{13}C NMR spectrum of compound **3i** as a diastereoisomeric mixture ($CDCl_3$).

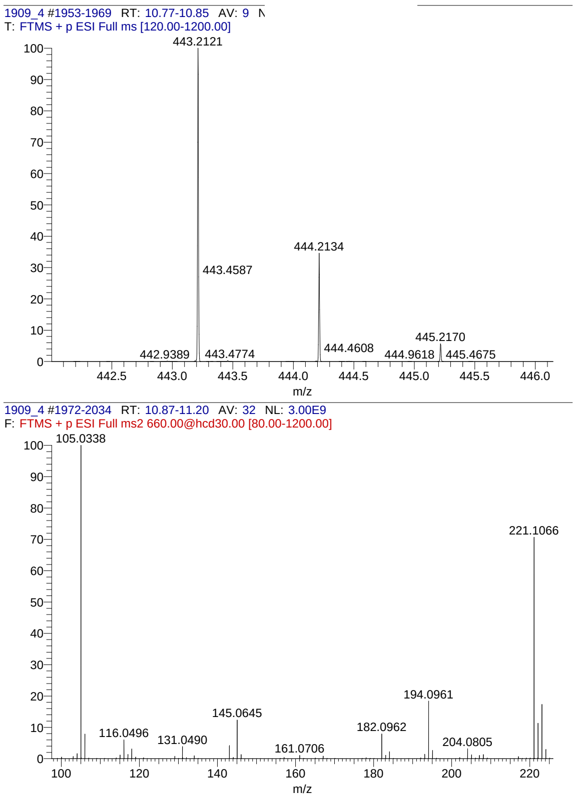

Figure S44: HRMS ESI and HRMS ESI-MS/MS spectrum of compound **3i** as a diastereoisomeric mixture.

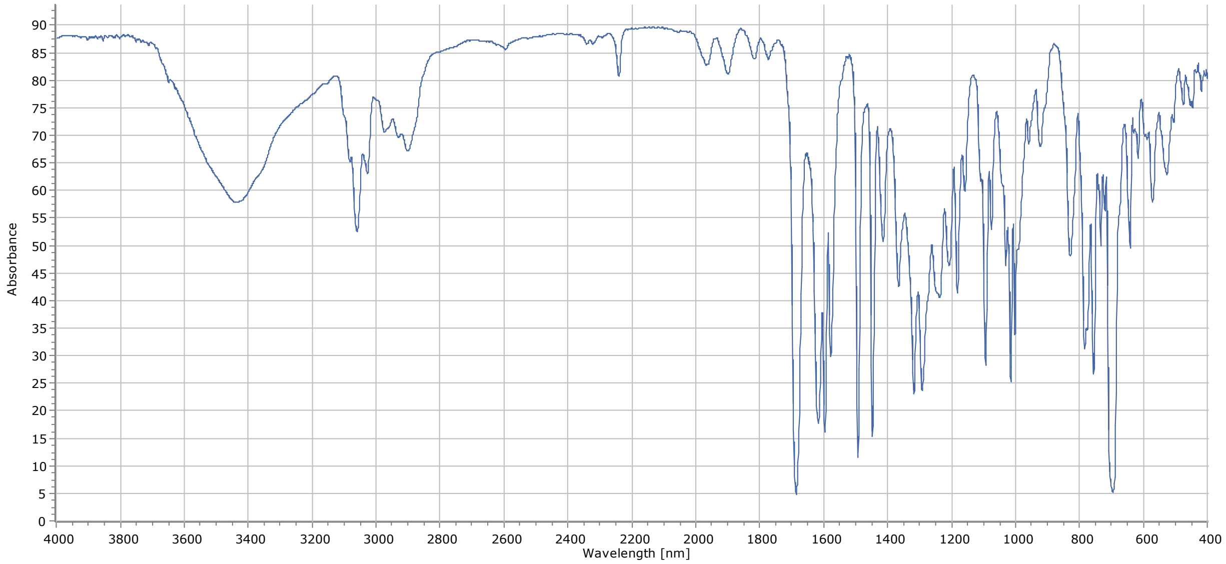

Figure S45: FT-IR spectrum of compound **3j** as a diastereoisomeric mixture (KBr).

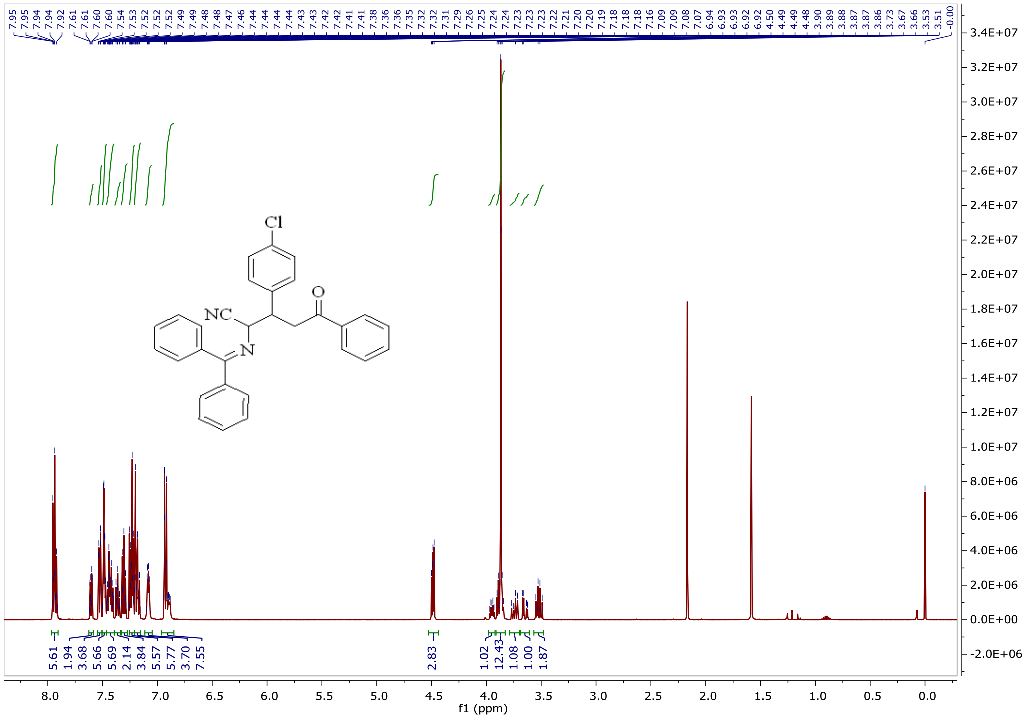

Figure S46: ^{1}H-NMR spectrum of compound **3j** as a diastereoisomeric mixture ($CDCl_3$).

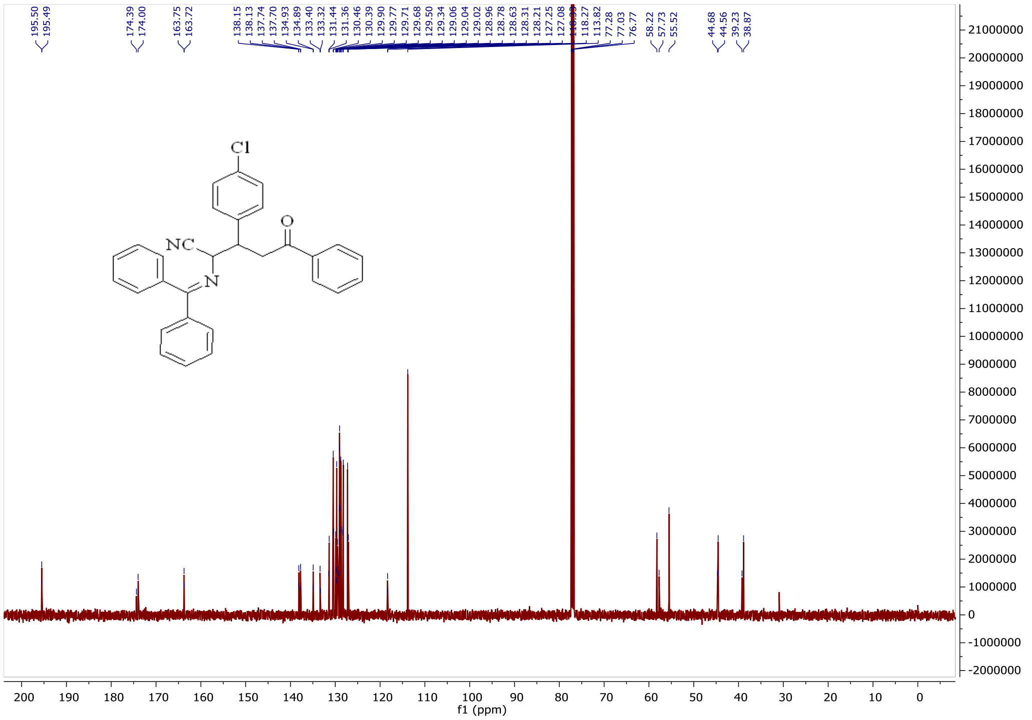

Figure S47: ^{13}C NMR spectrum of compound **3j** as a diastereoisomeric mixture ($CDCl_3$).

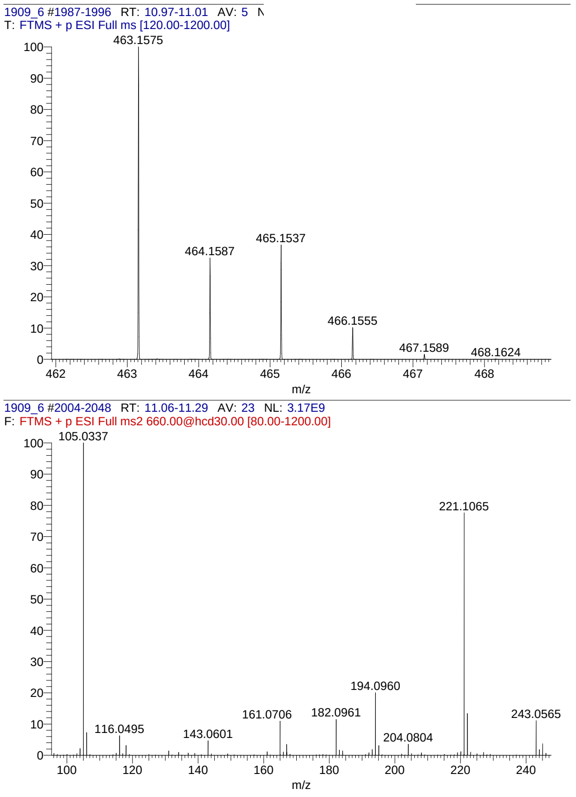

Figure S48: HRMS ESI and HRMS ESI-MS/MS spectrum of compound **3j** as a diastereoisomeric mixture.

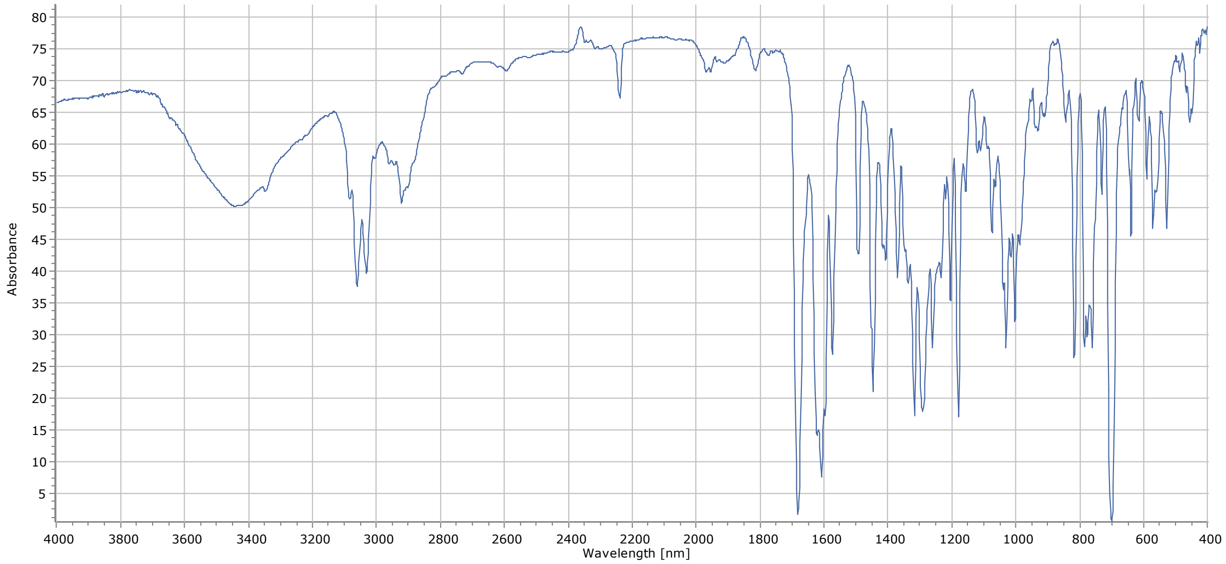

Figure S49: FT-IR spectrum of compound **3k** as a diastereoisomeric mixture (KBr).

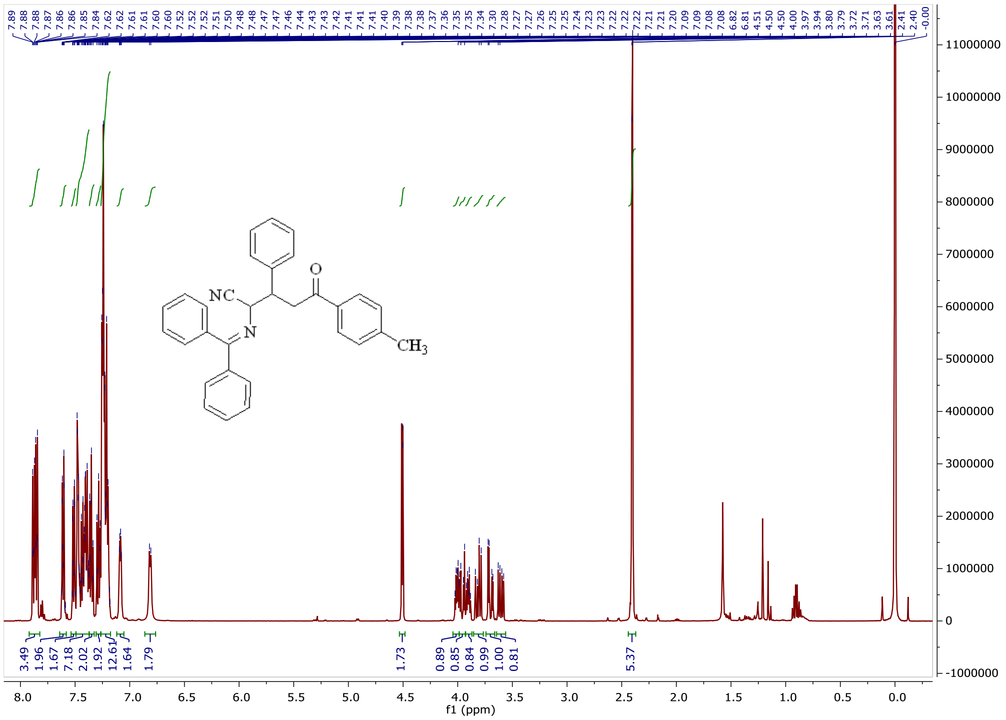

Figure S50: ¹H-NMR spectrum of compound **3k** as a diastereoisomeric mixture ($CDCl_3$).

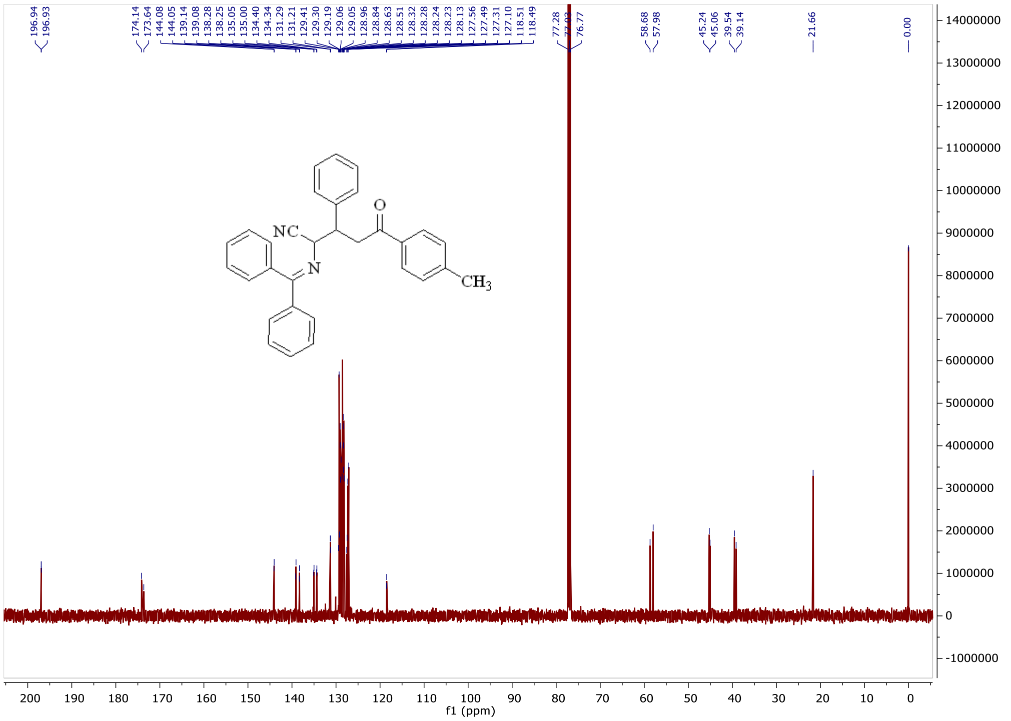

Figure S51: ^{13}C NMR spectrum of compound **3k** as a diastereoisomeric mixture ($CDCl_3$).

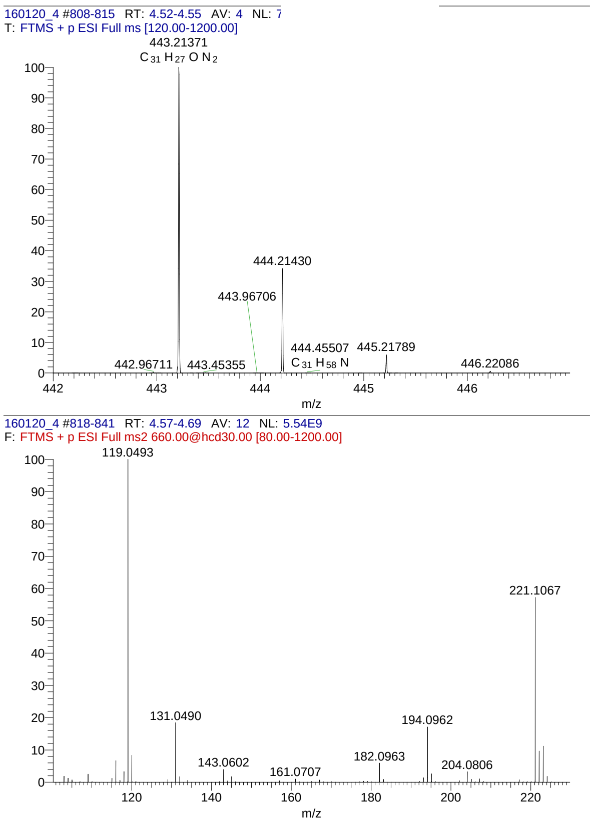

Figure S52: HRMS ESI and HRMS ESI-MS/MS spectrum of compound **3k** as a diastereoisomeric mixture.

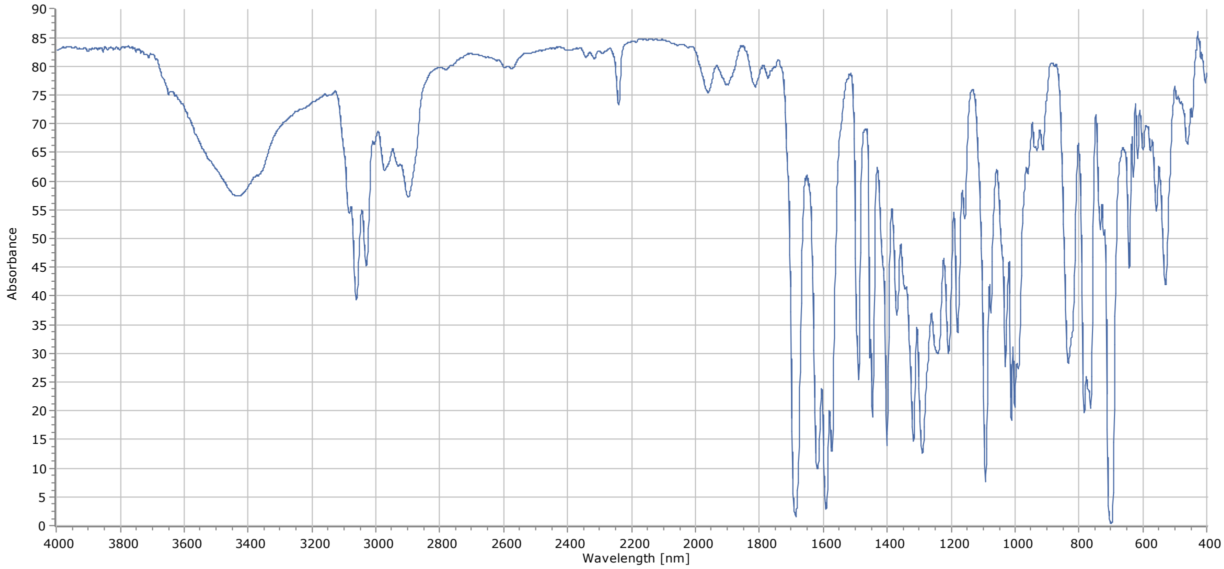

Figure S53: FT-IR spectrum of compound **3l** as a diastereoisomeric mixture (KBr).

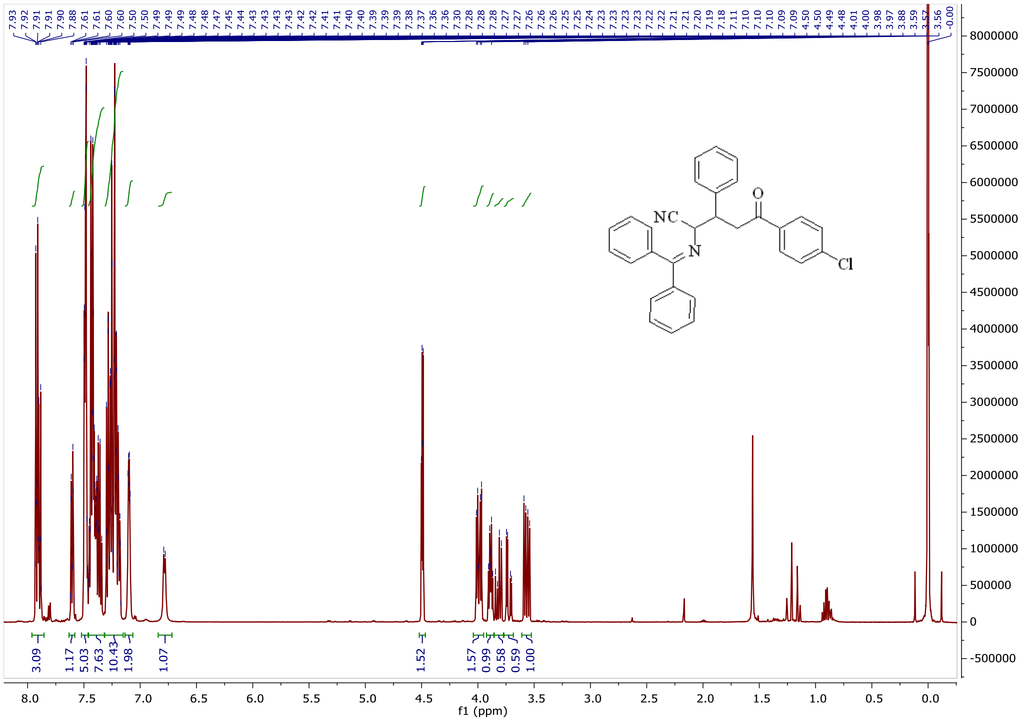

Figure S54: ^{1}H-NMR spectrum of compound **3l** as a diastereoisomeric mixture ($CDCl_3$).

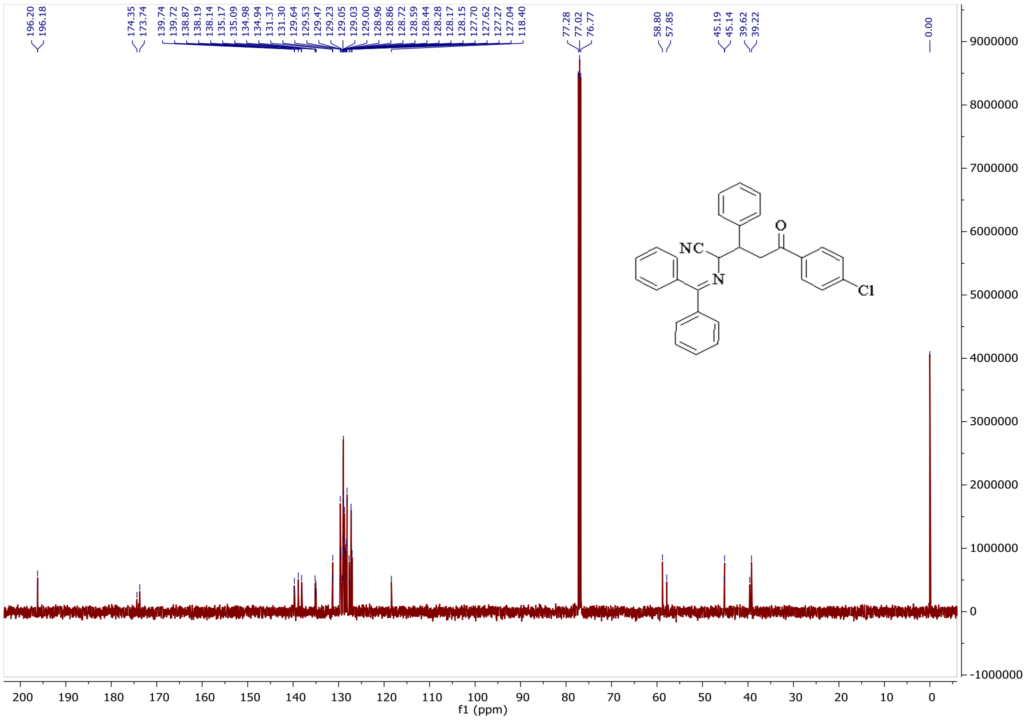

Figure S55: ^{13}C NMR spectrum of compound **3l** as a diastereoisomeric mixture ($CDCl_3$).

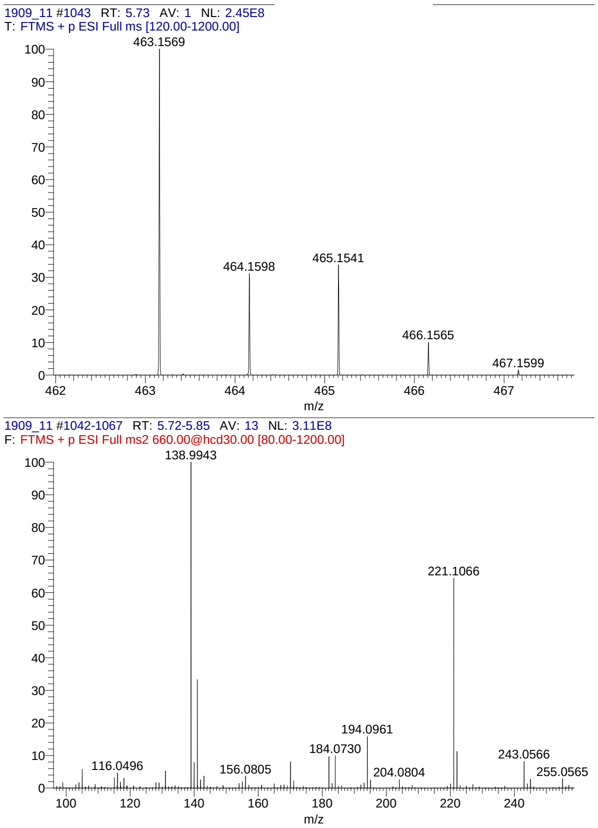

Figure S56: HRMS ESI and HRMS ESI-MS/MS spectrum of compound **3l** as a diastereoisomeric mixture.

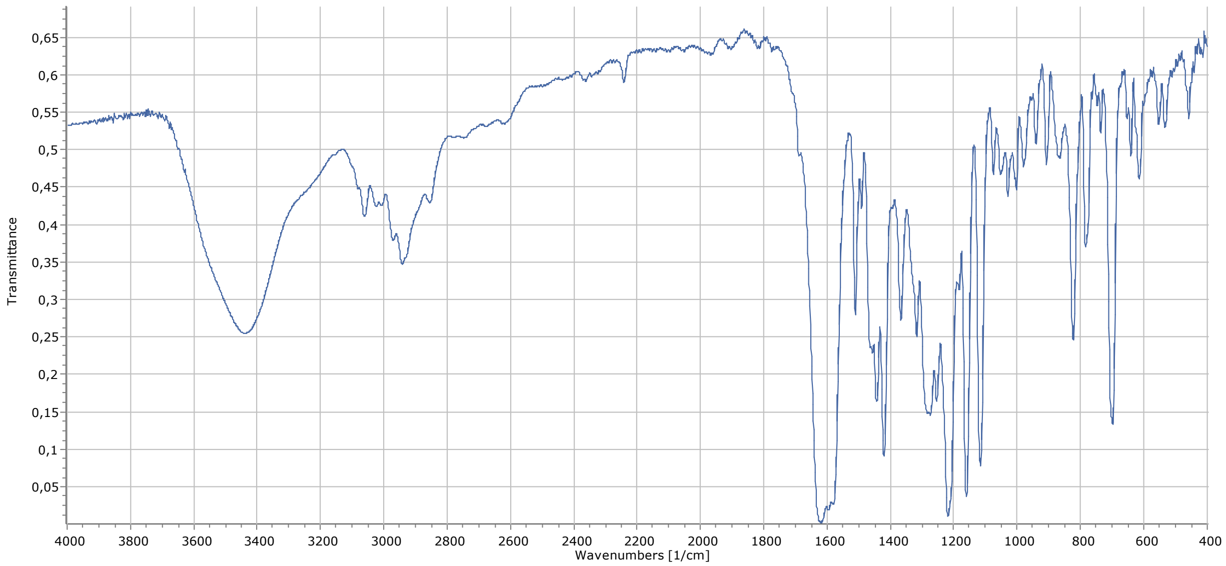

Figure S57: FT-IR spectrum of compound **3m** as a diastereoisomeric mixture (KBr).

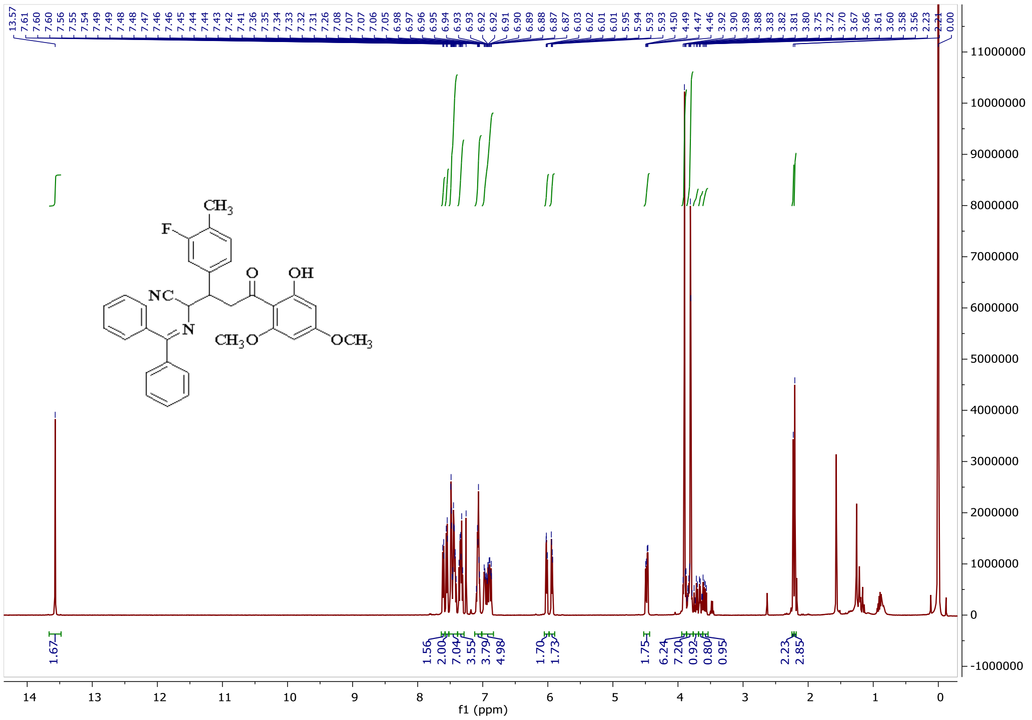

Figure S58: ^{1}H-NMR spectrum of compound **3m** as a diastereoisomeric mixture ($CDCl_3$).

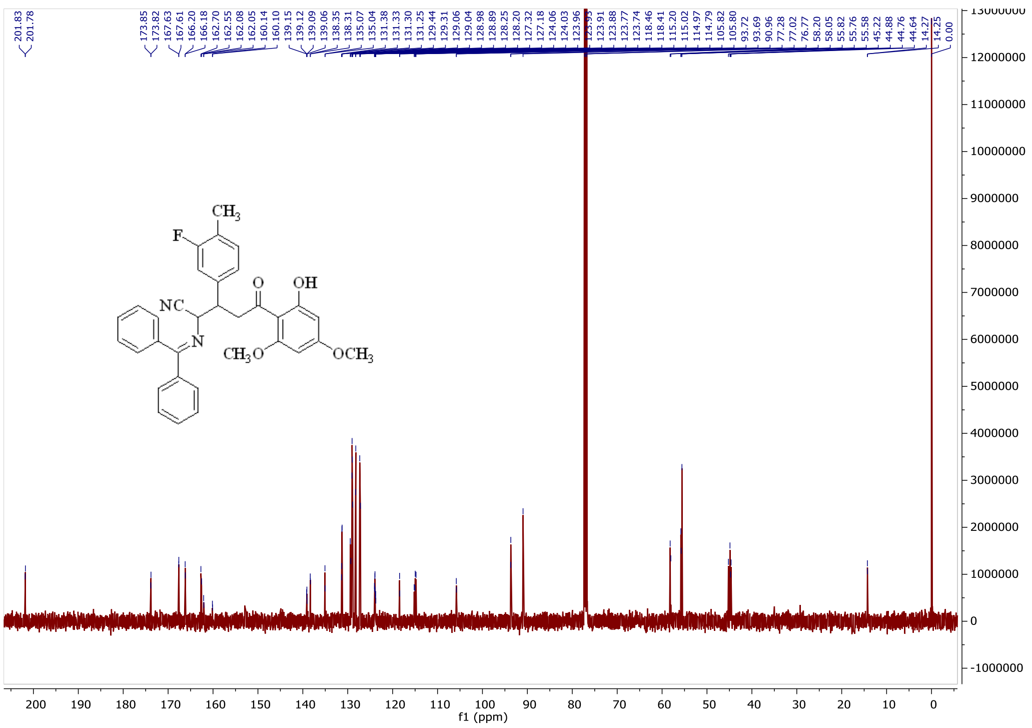

Figure S59: ^{13}C NMR spectrum of compound **3m** as a diastereoisomeric mixture ($CDCl_3$).

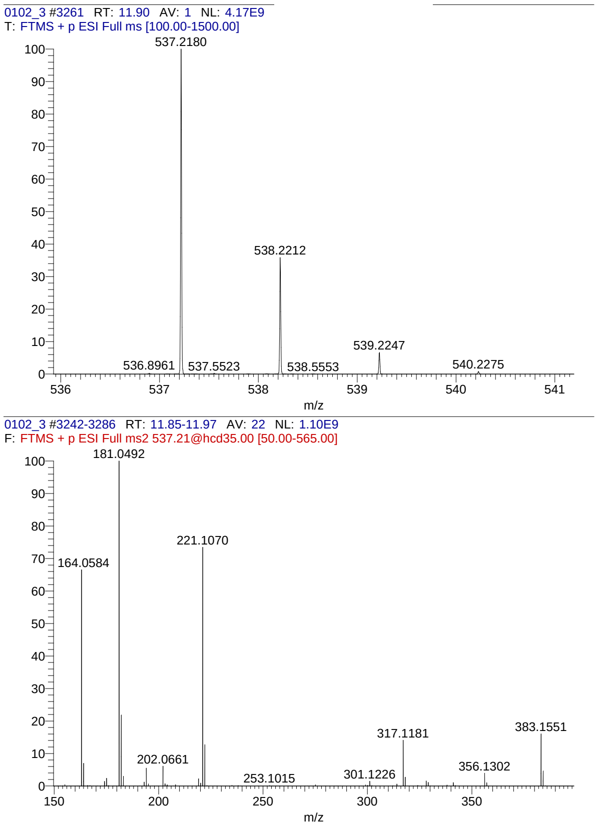

Figure S60: HRMS ESI and HRMS ESI-MS/MS spectrum of compound **3m** as a diastereoisomeric mixture.

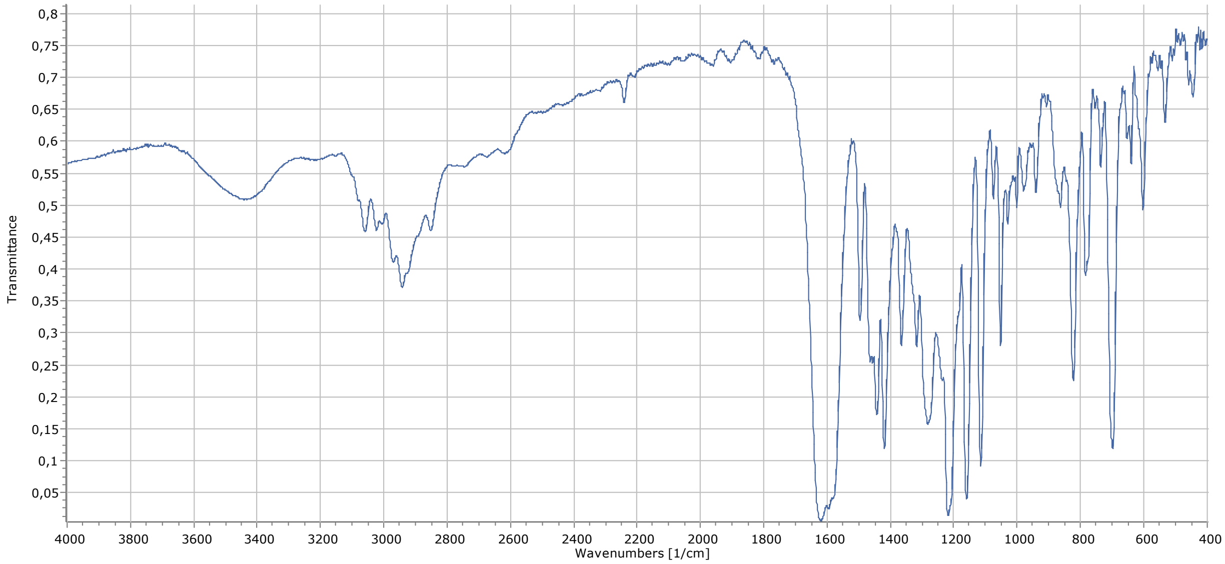

Figure S61: FT-IR spectrum of compound **3n** as a diastereoisomeric mixture (KBr).

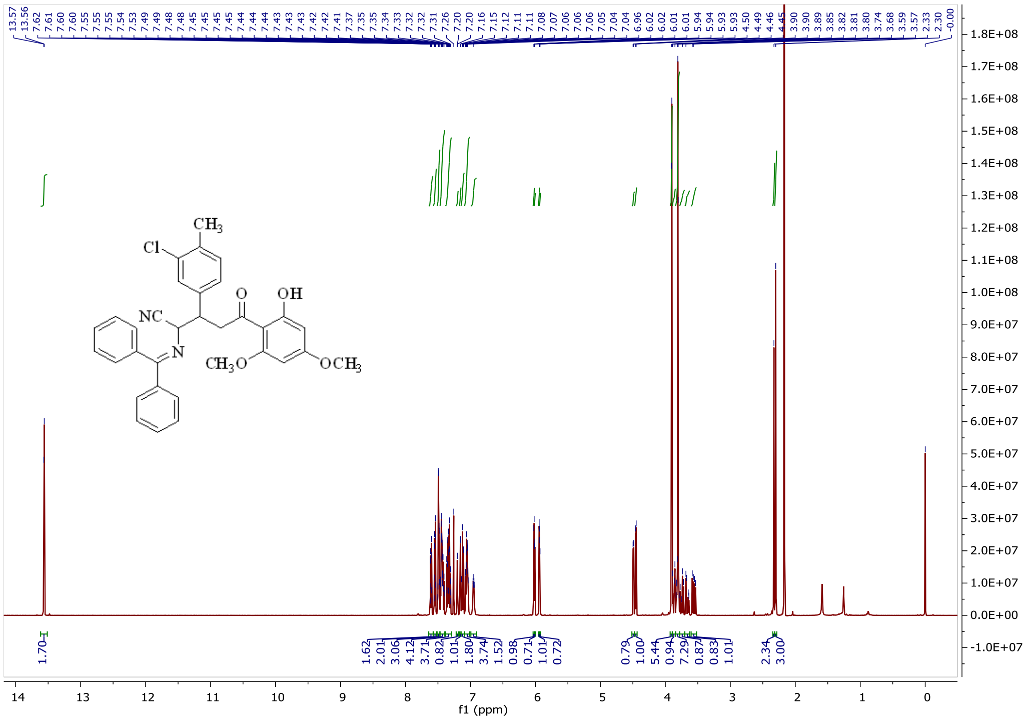

Figure S62: ¹H-NMR spectrum of compound **3n** as a diastereoisomeric mixture ($CDCl_3$).

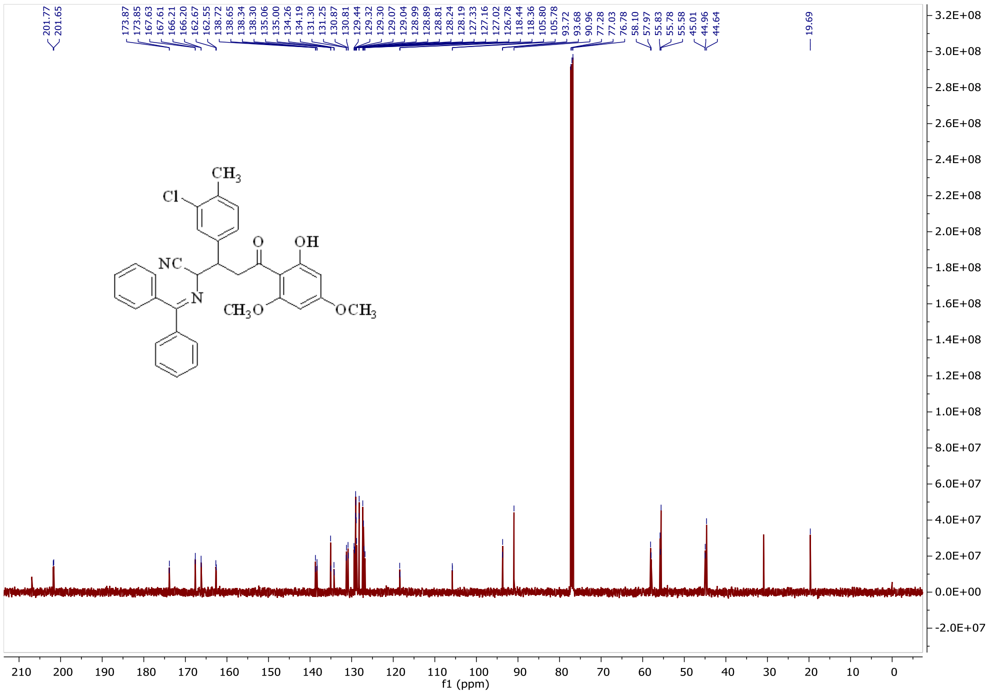

Figure S63: ^{13}C NMR spectrum of compound **3n** as a diastereoisomeric mixture ($CDCl_3$).

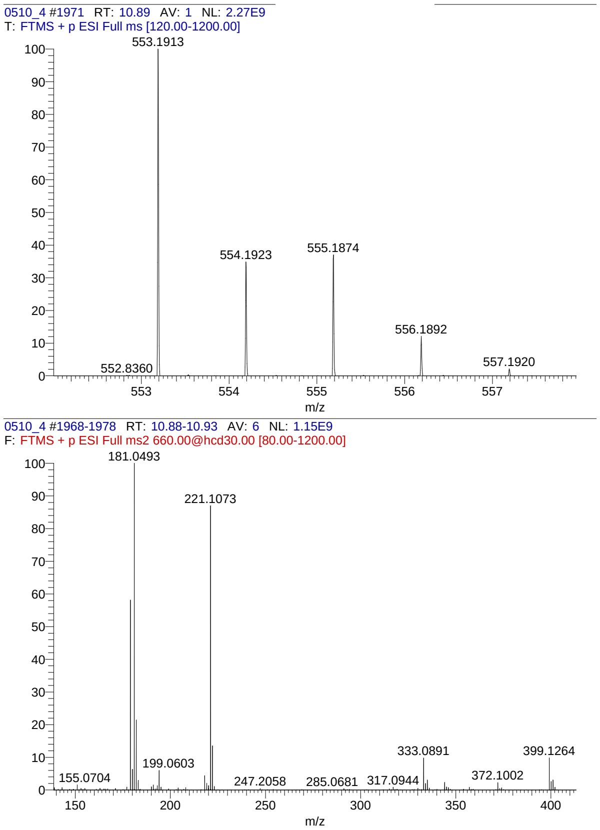

Figure S64: HRMS ESI and HRMS ESI-MS/MS spectrum of compound **3n** as a diastereoisomeric mixture.

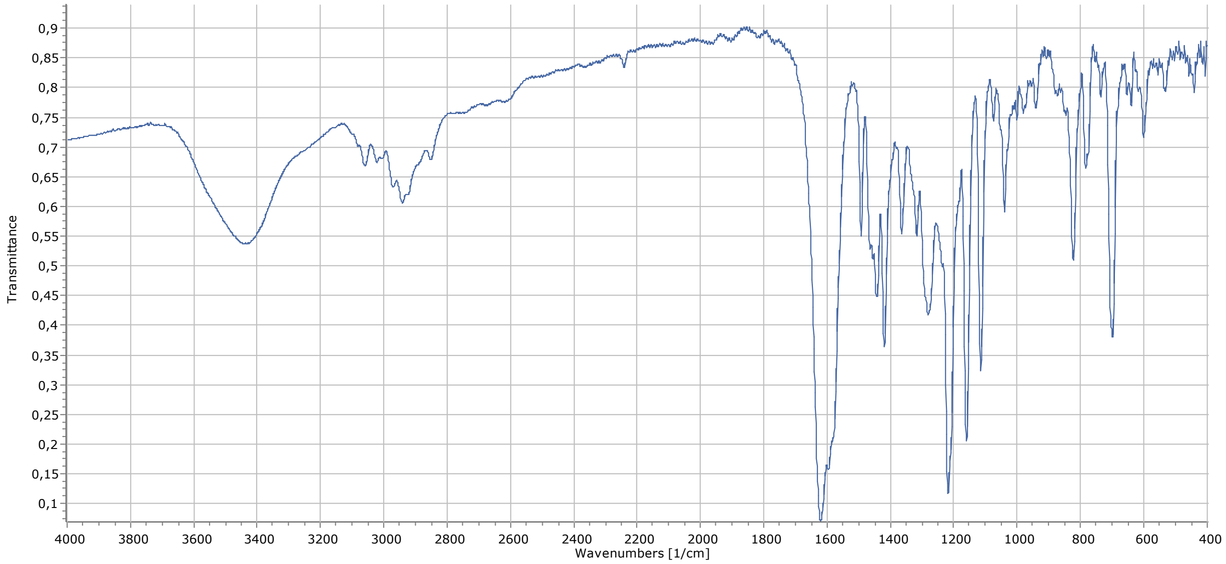

Figure S65: FT-IR spectrum of compound **3o** as a diastereoisomeric mixture (KBr).

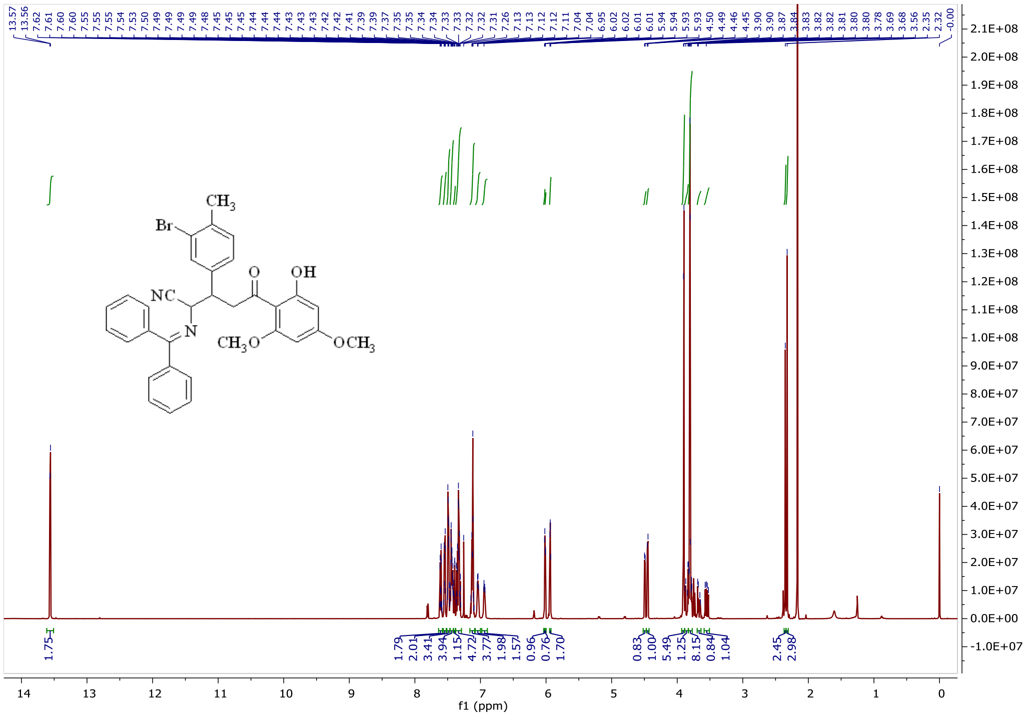

Figure S66: ^{1}H-NMR spectrum of compound **3o** as a diastereoisomeric mixture ($CDCl_3$).

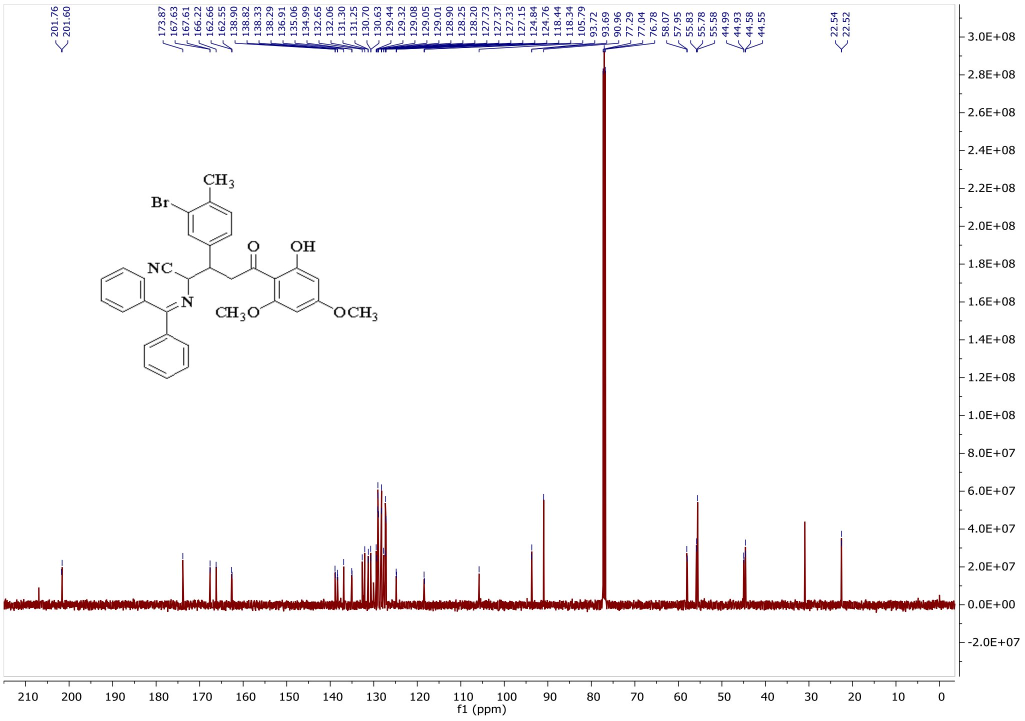

Figure S67: ^{13}C NMR spectrum of compound **3o** as a diastereoisomeric mixture ($CDCl_3$).

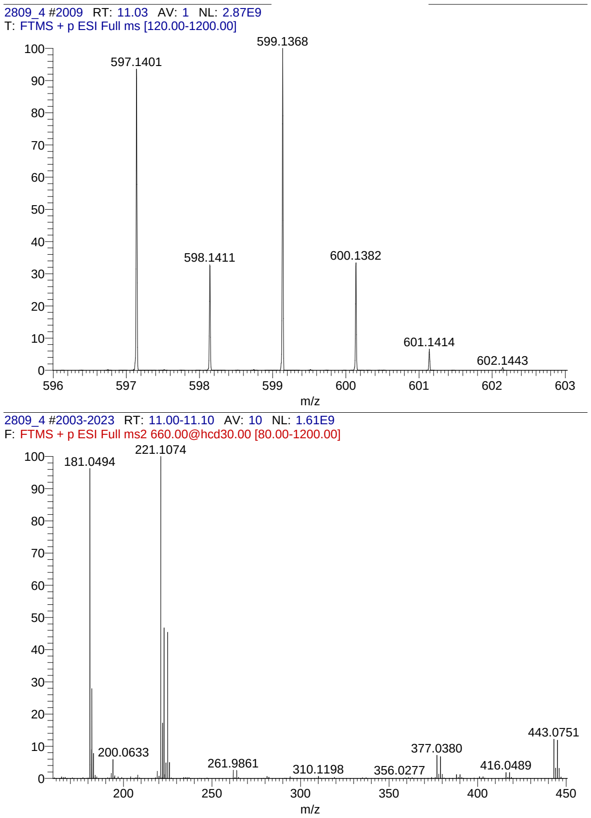

Figure S68: HRMS ESI and HRMS ESI-MS/MS spectrum of compound **3o** as a diastereoisomeric mixture.

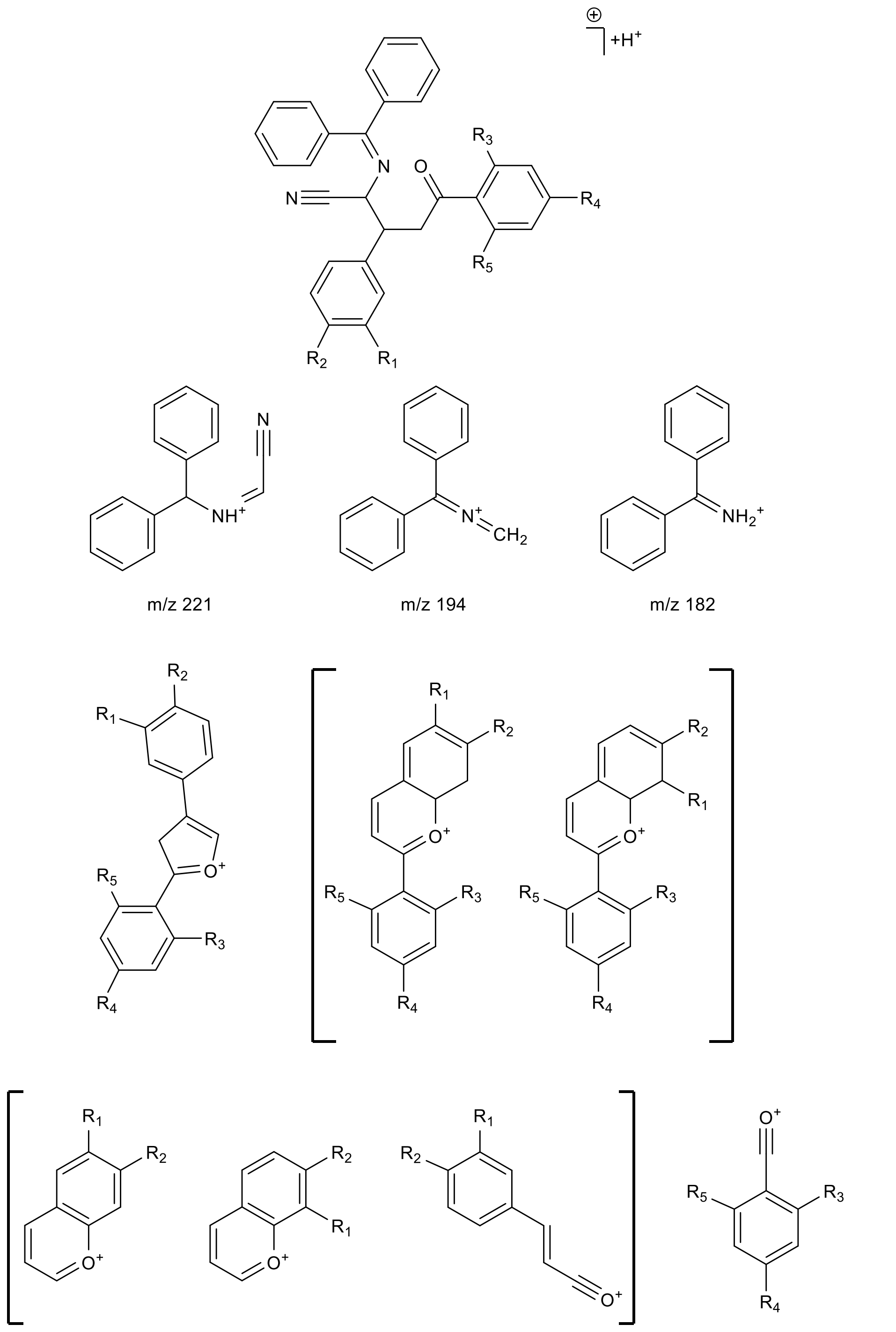

Figure S69: Proposed structures of the most abundant fragment ions observed in a MS/MS spectra of compounds **3a-3o**.

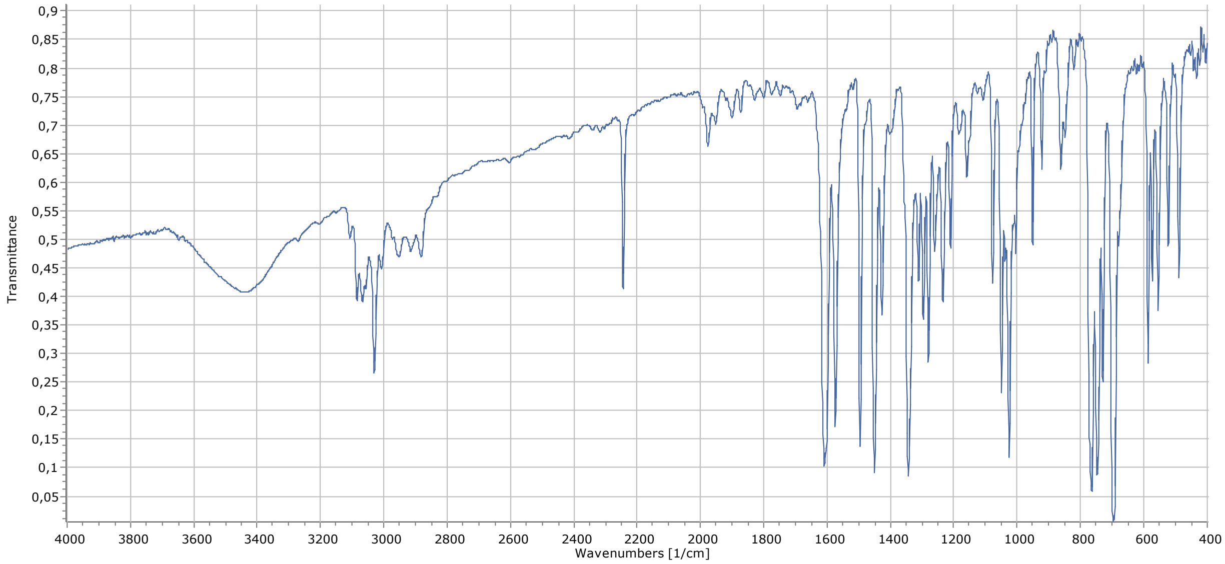

Figure S70: FT-IR spectrum of compound *cis*-**4a** (KBr).

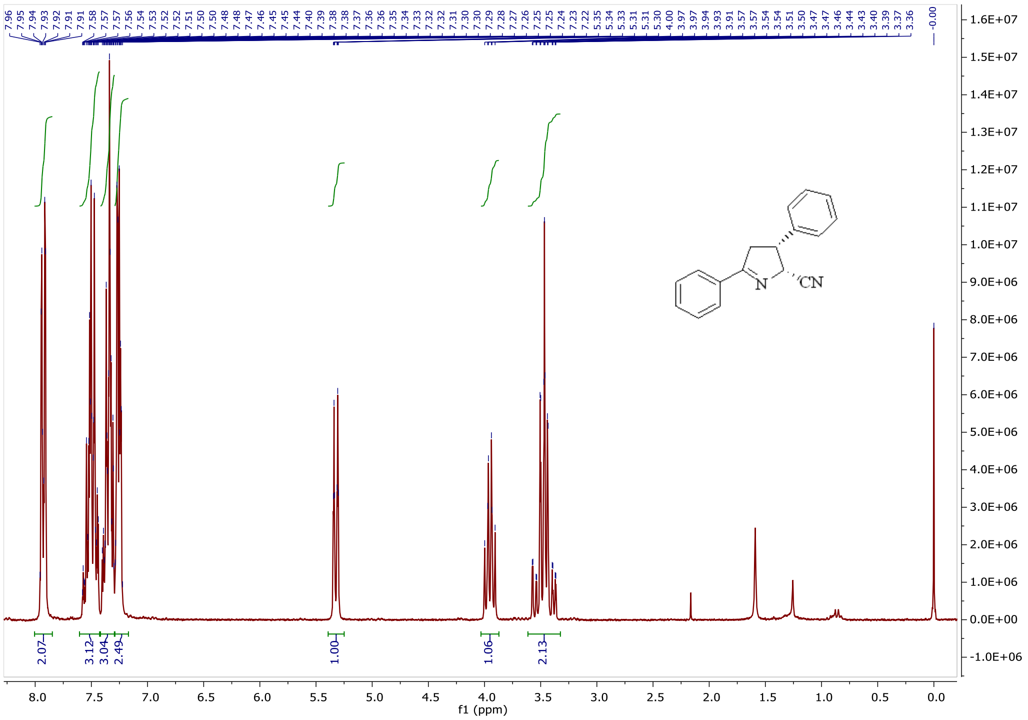

Figure S71: ^{1}H-NMR spectrum of compound *cis*-**4a** ($CDCl_3$).

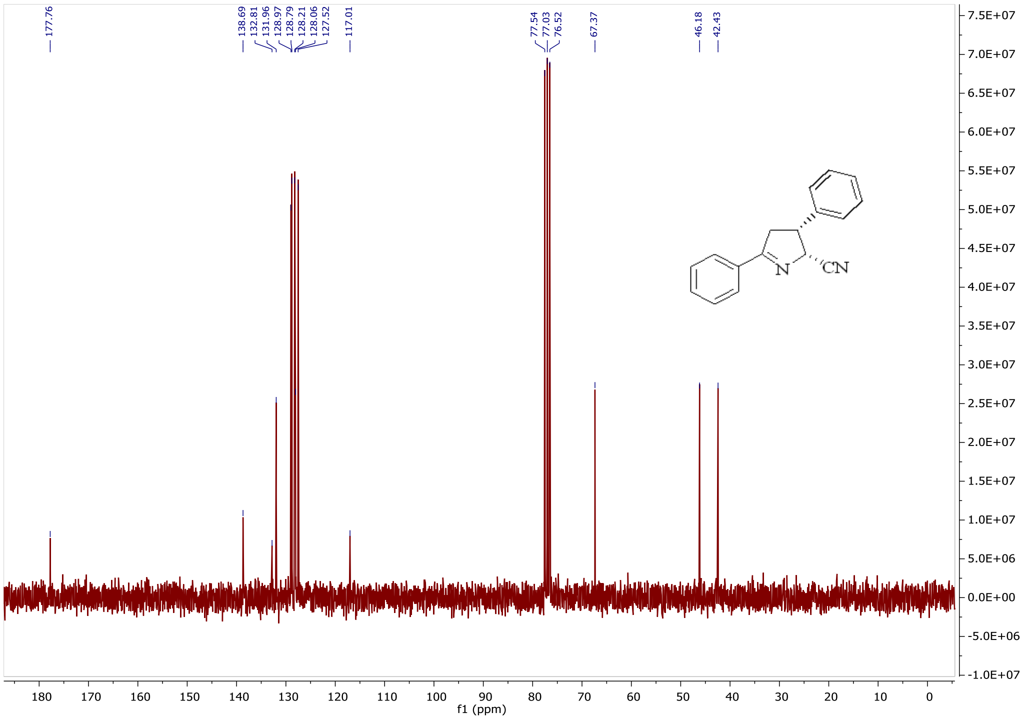

Figure S72: ^{13}C NMR spectrum of compound *cis*-**4a** ($CDCl_3$).

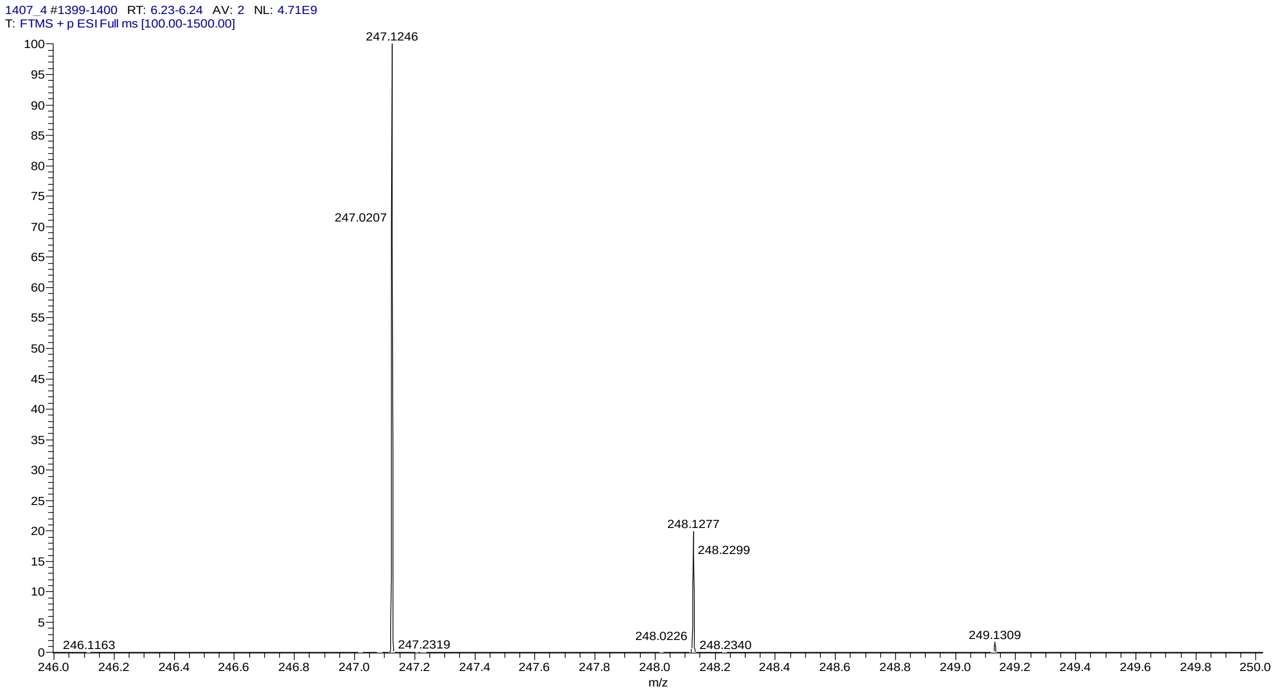

Figure S73: HRMS ESI spectrum of compound *cis*-**4a**.

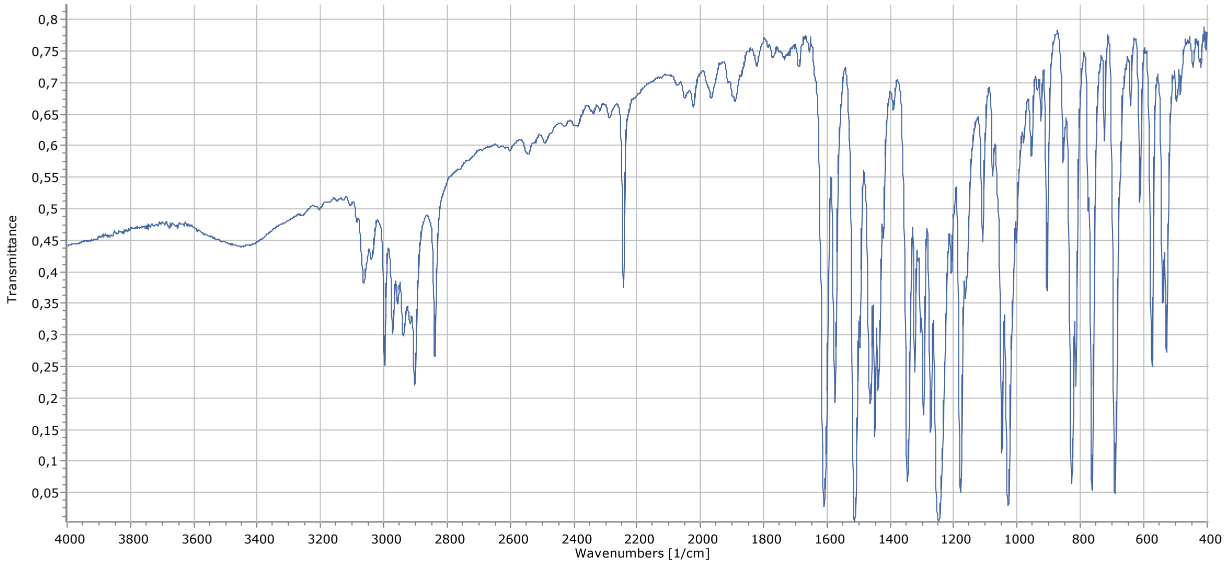

Figure S74: FT-IR spectrum of compound *trans*-**4b** (KBr).

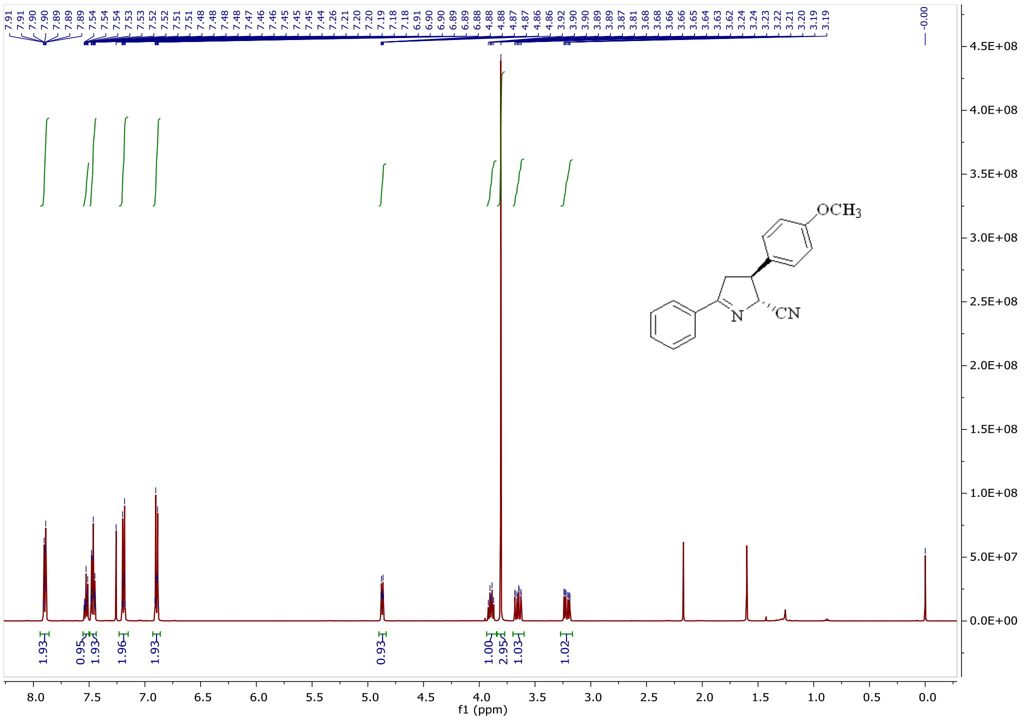

Figure S75: ^{1}H-NMR spectrum of compound *trans*-**4b** ($CDCl_3$).

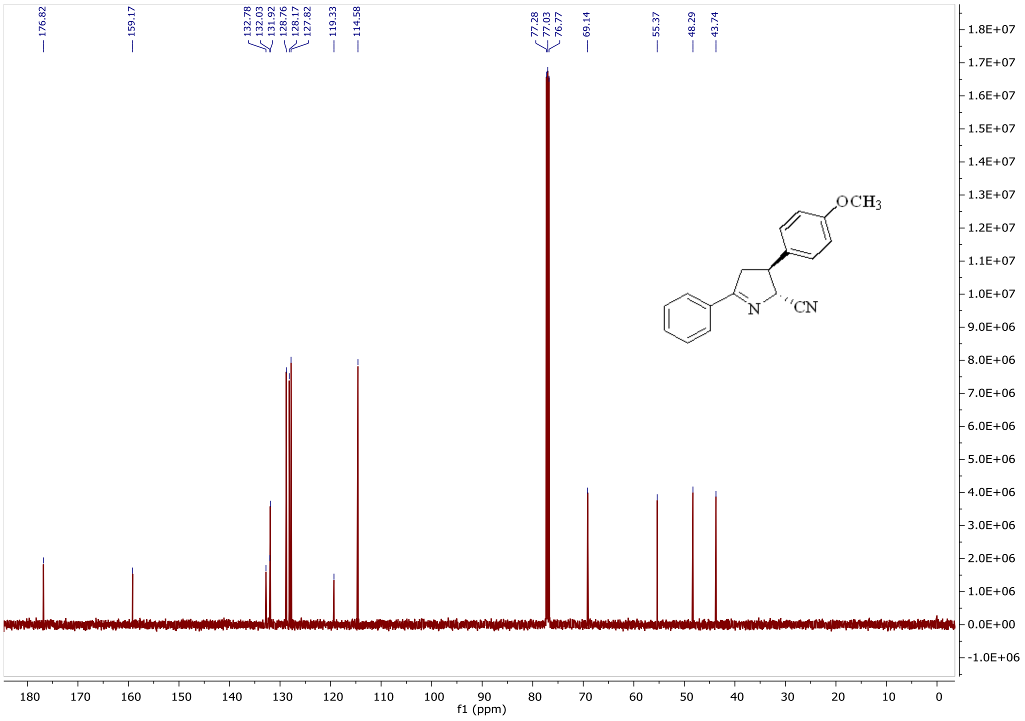

Figure S76: ^{13}C NMR spectrum of compound *trans*-**4b** ($CDCl_3$).

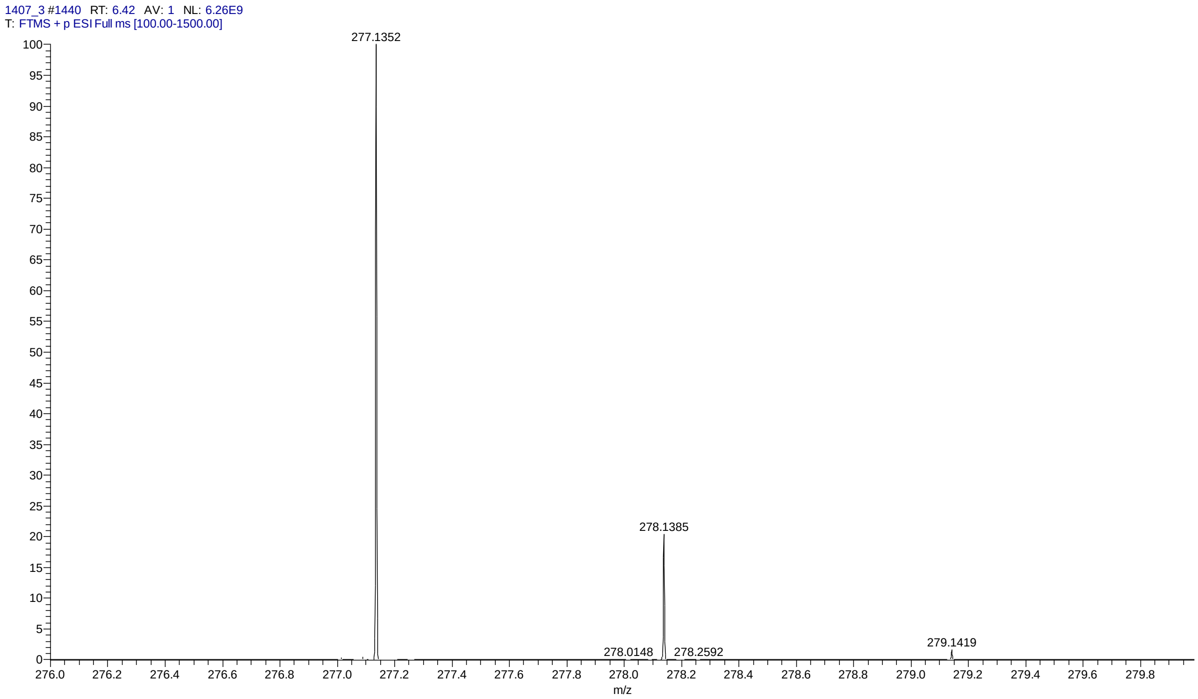

Figure S77: HRMS ESI spectrum of compound *trans*-**4b**.

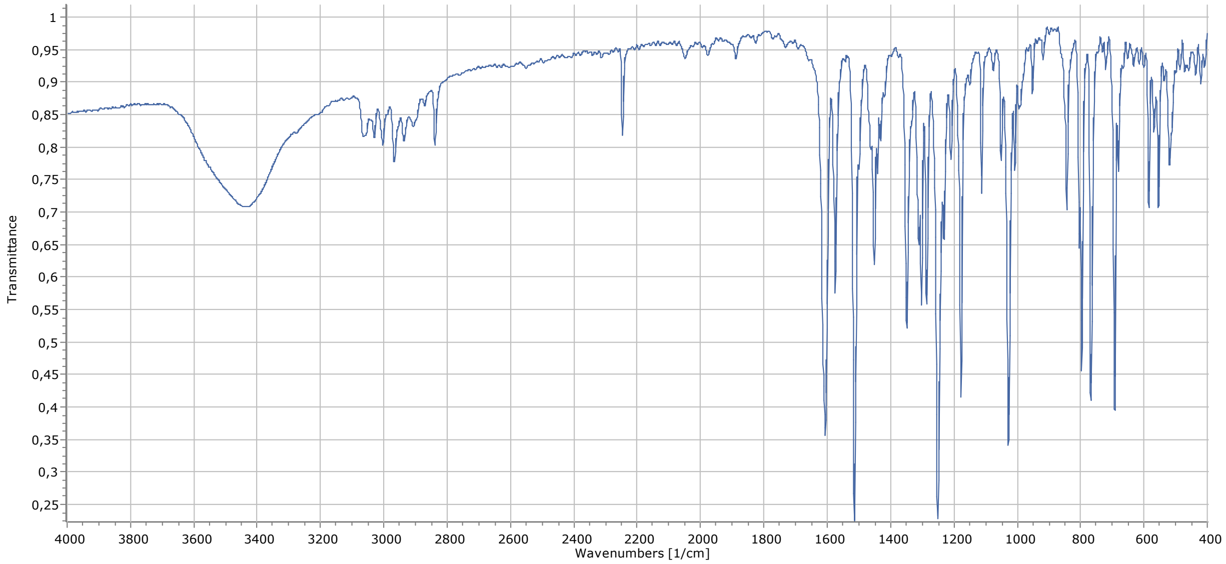

Figure S78: FT-IR spectrum of compound *cis*-**4b** (KBr).

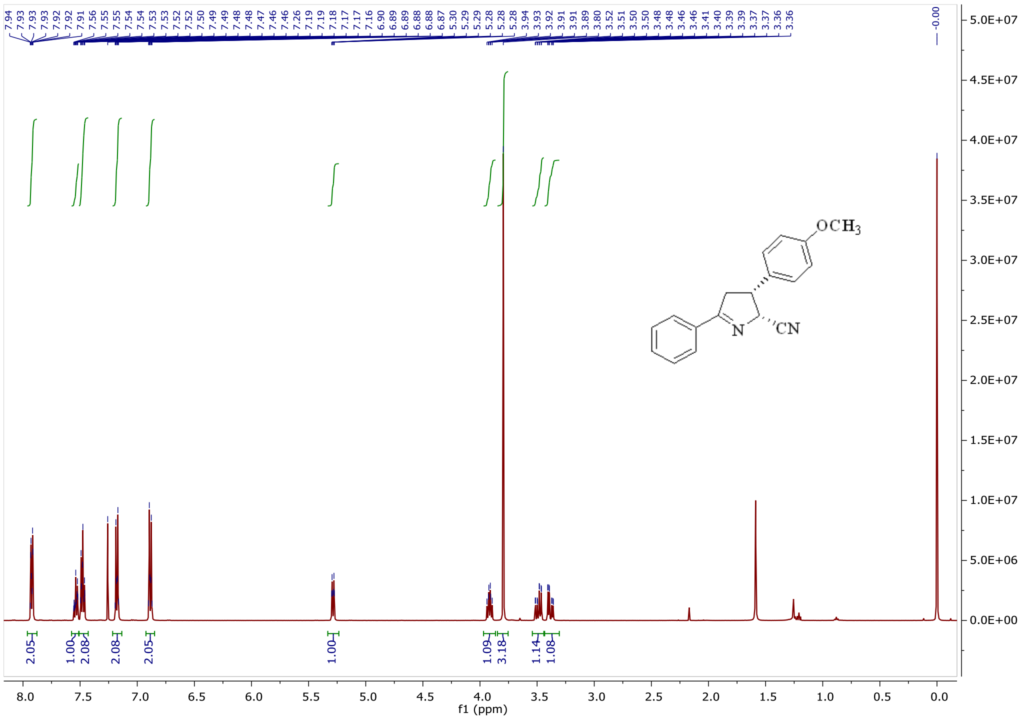

Figure S79: ^{1}H-NMR spectrum of compound *cis*-**4b** ($CDCl_3$).

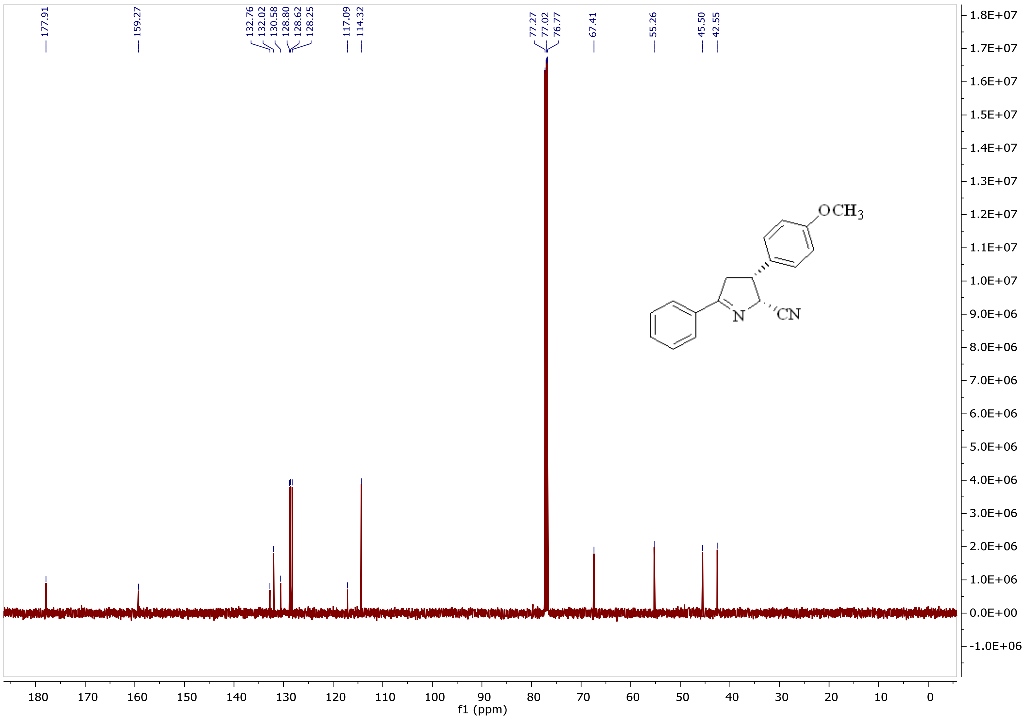

Figure S80: ^{13}C NMR spectrum of compound *cis*-**4b** ($CDCl_3$).

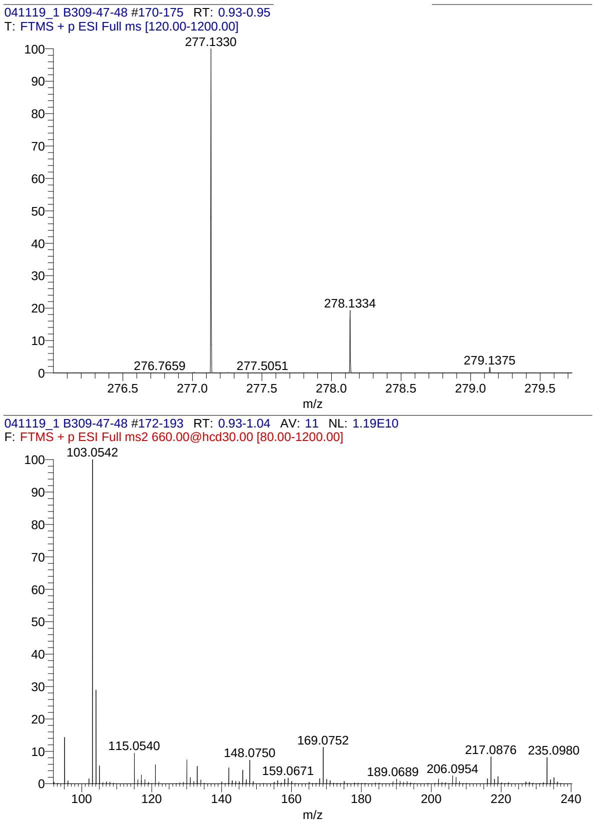

Figure S81: HRMS ESI and HRMS ESI-MS/MS spectrum of compound *cis*-**4b**.

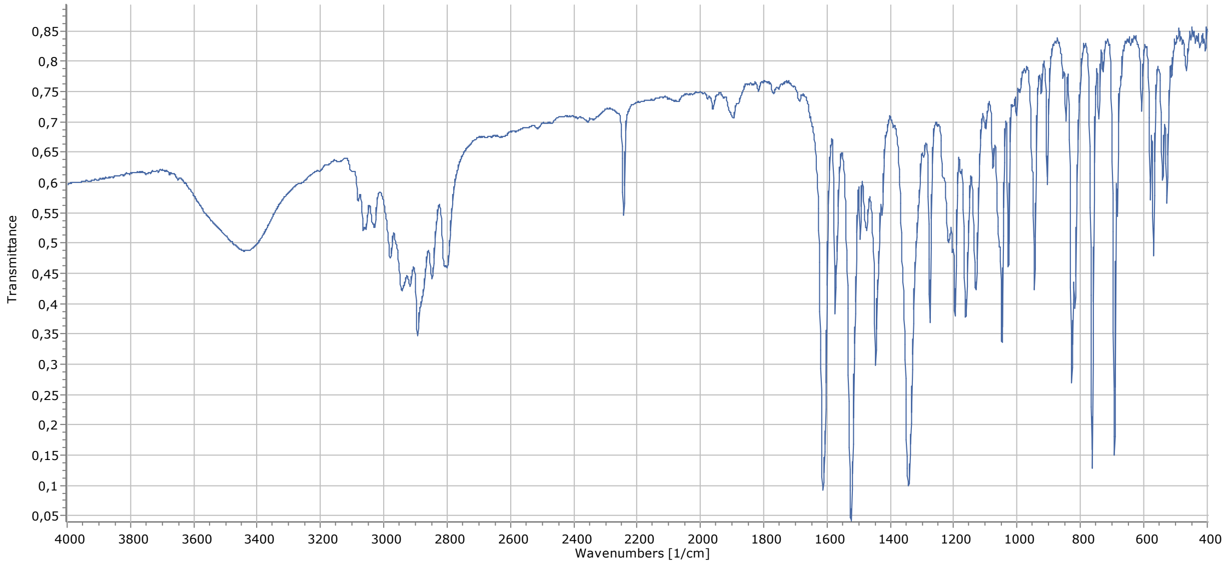

Figure S82: FT-IR spectrum of compound *trans*-**4c** (KBr).

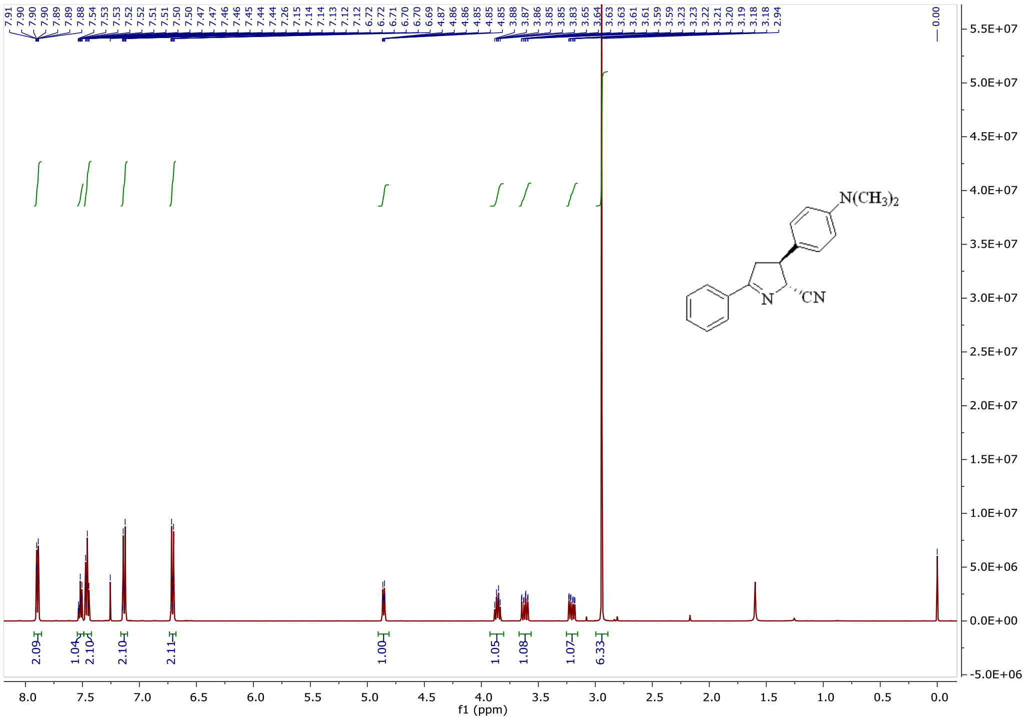

Figure S83: ^{1}H-NMR spectrum of compound *trans*-**4c** ($CDCl_3$).

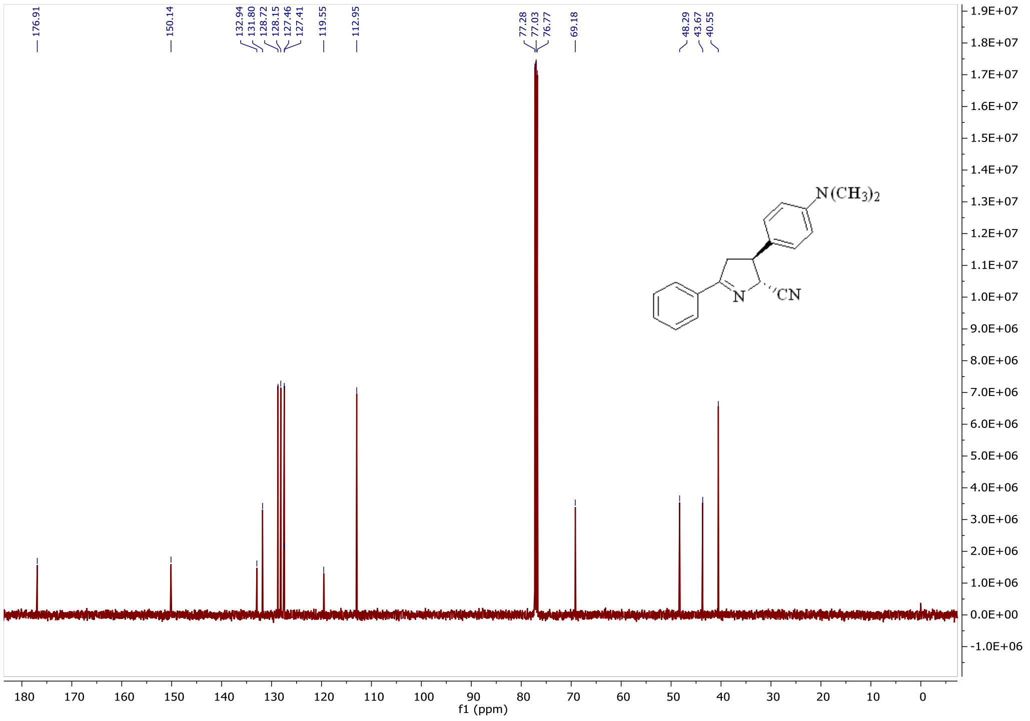

Figure S84: ^{13}C NMR spectrum of compound *trans*-**4c** ($CDCl_3$).

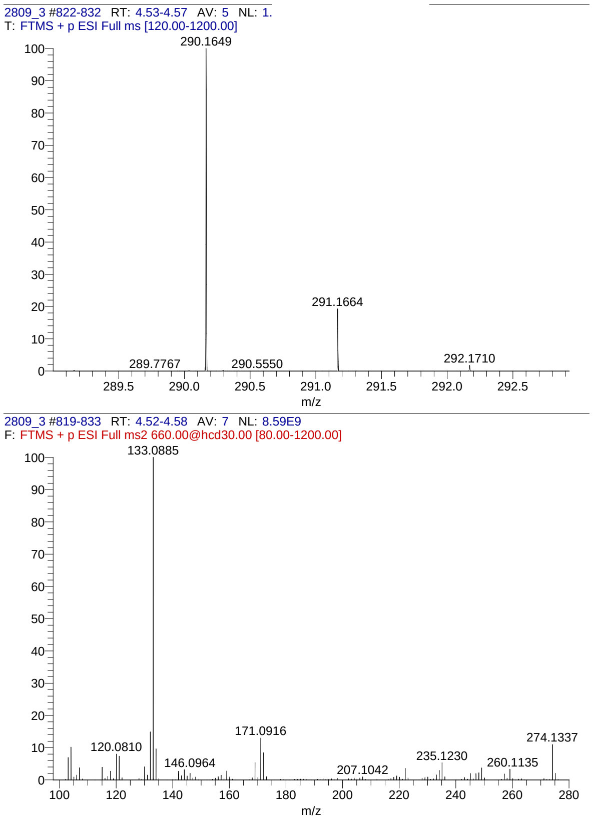

Figure S85: HRMS ESI and HRMS ESI-MS/MS spectrum of compound *trans*-**4c**.

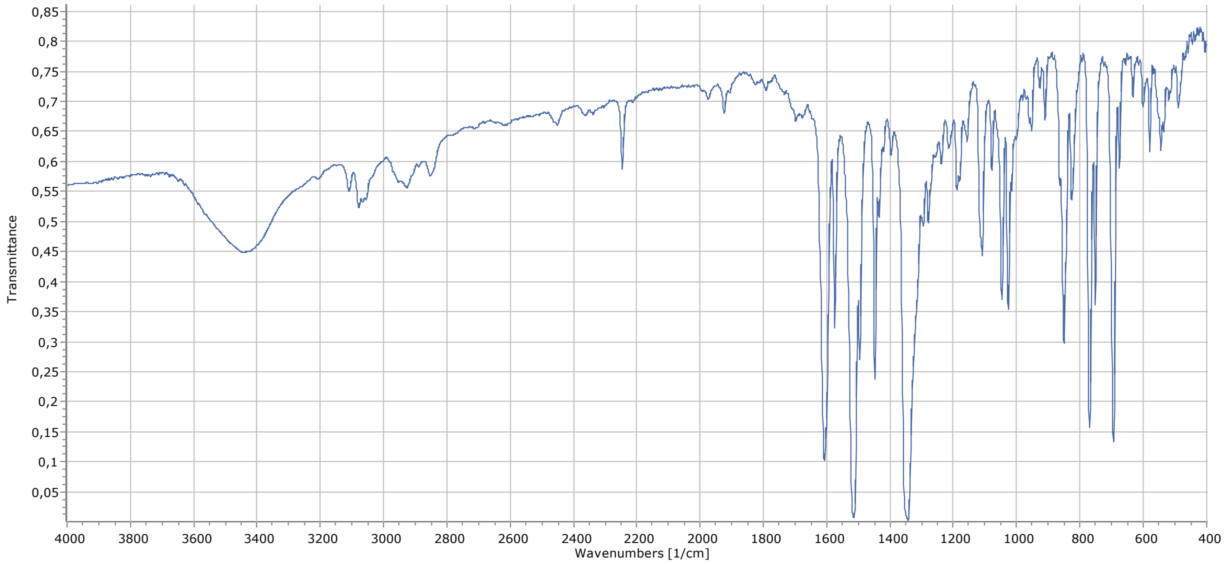

Figure S86: FT-IR spectrum of compound **4d** as a diastereoisomeric mixture (KBr).

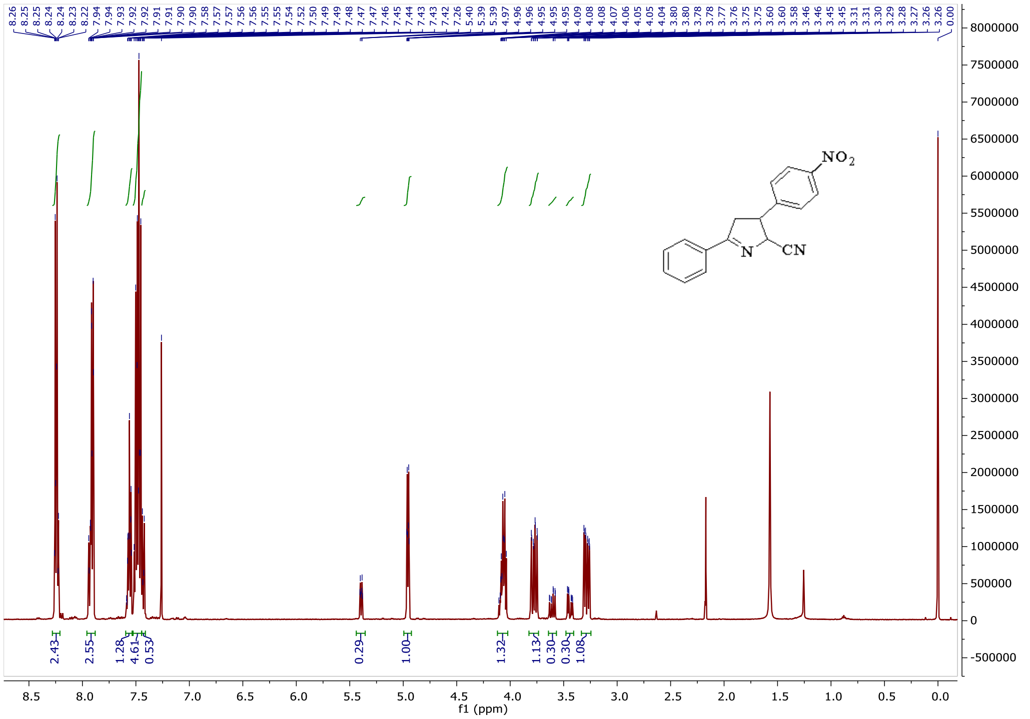

Figure S87: ^{1}H-NMR spectrum of compound **4d** as a diastereoisomeric mixture ($CDCl_3$).

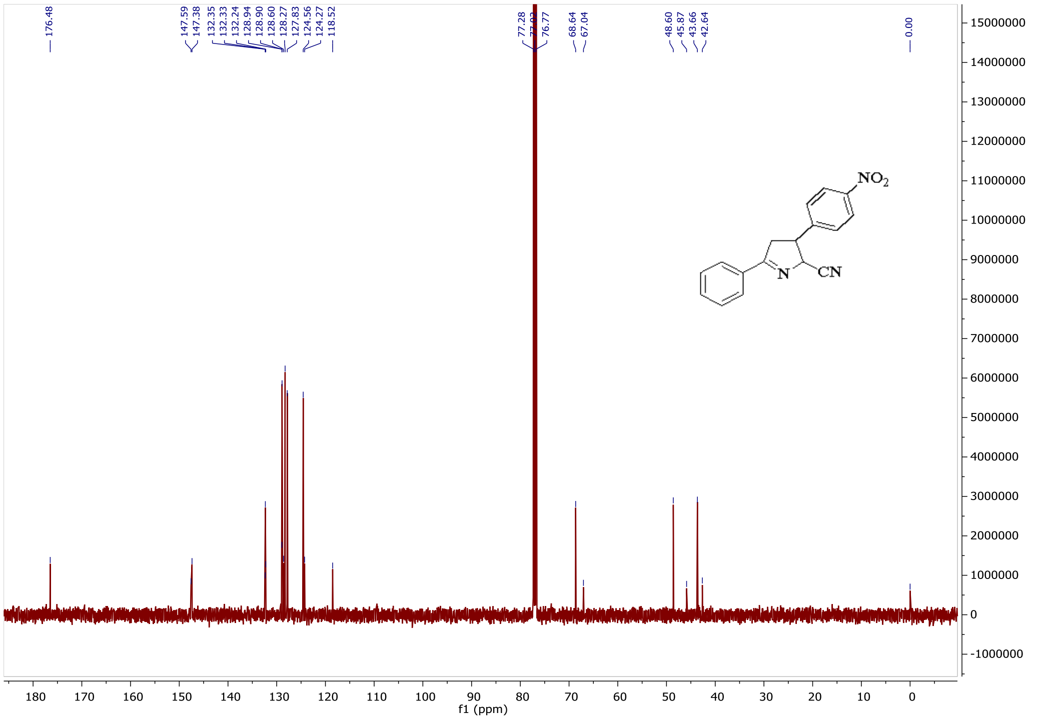

Figure S88: ^{13}C NMR spectrum of compound **4d** as a diastereoisomeric mixture ($CDCl_3$).

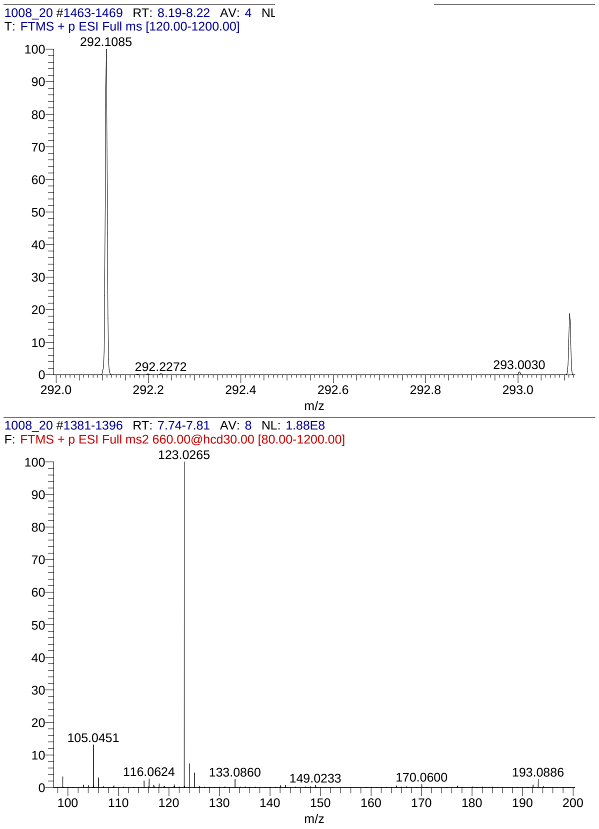

Figure S89: HRMS ESI and HRMS ESI-MS/MS spectrum of compound **4d** as a diastereoisomeric mixture.

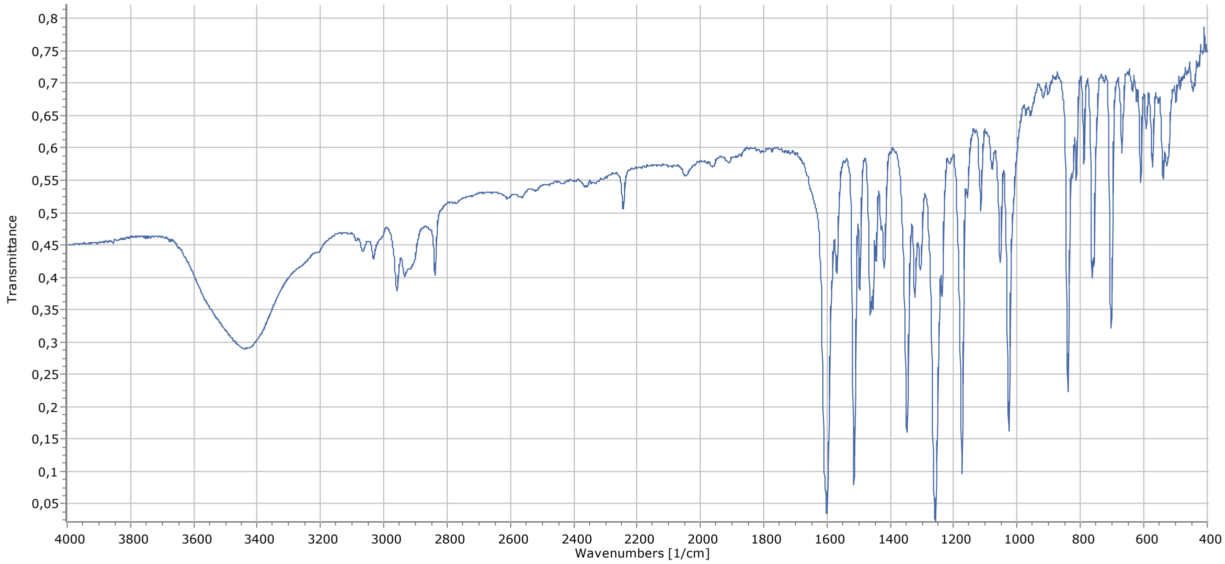

Figure S90: FT-IR spectrum of compound *trans*-**4e** (KBr).

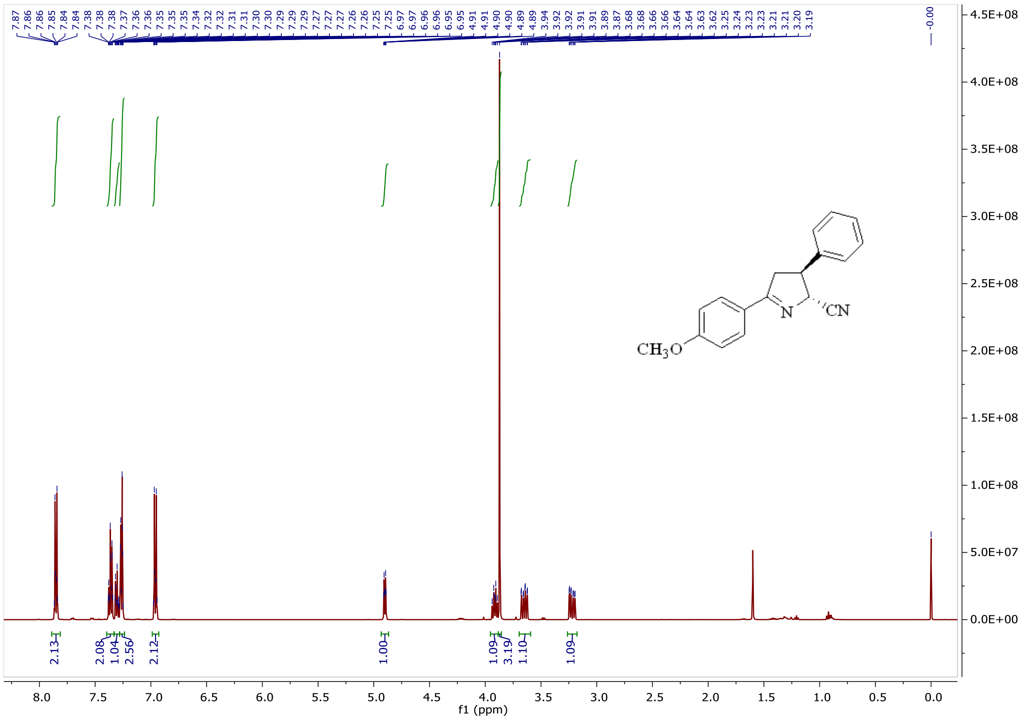

Figure S91: ^{1}H-NMR spectrum of compound *trans*-**4e** ($CDCl_3$).

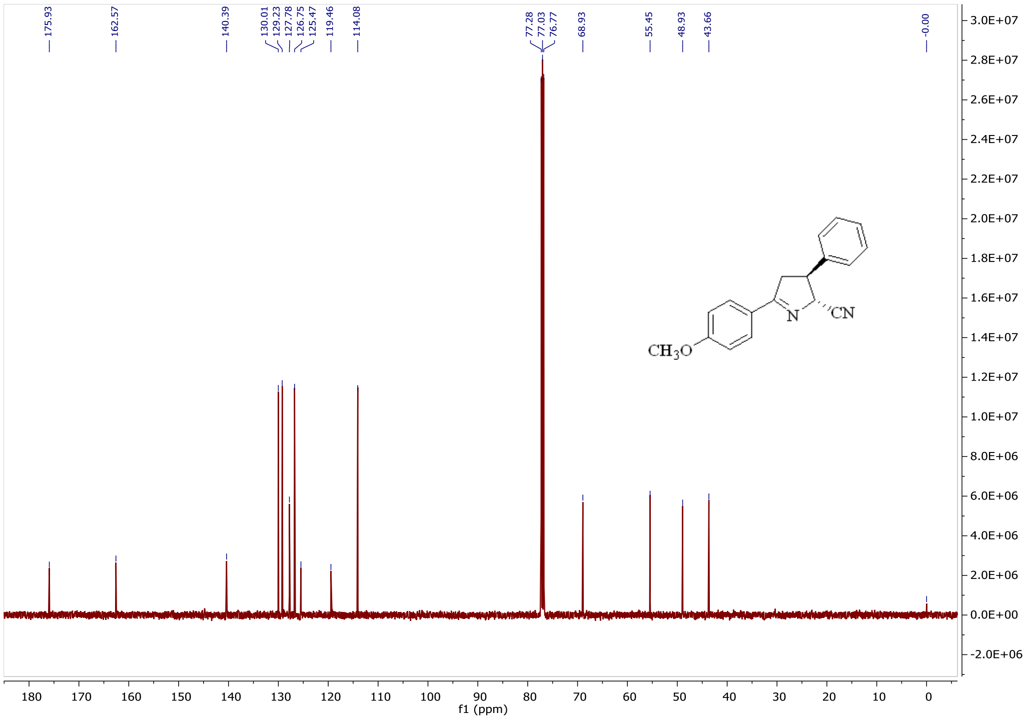

Figure S92: ^{13}C NMR spectrum of compound *trans*-**4e** ($CDCl_3$).

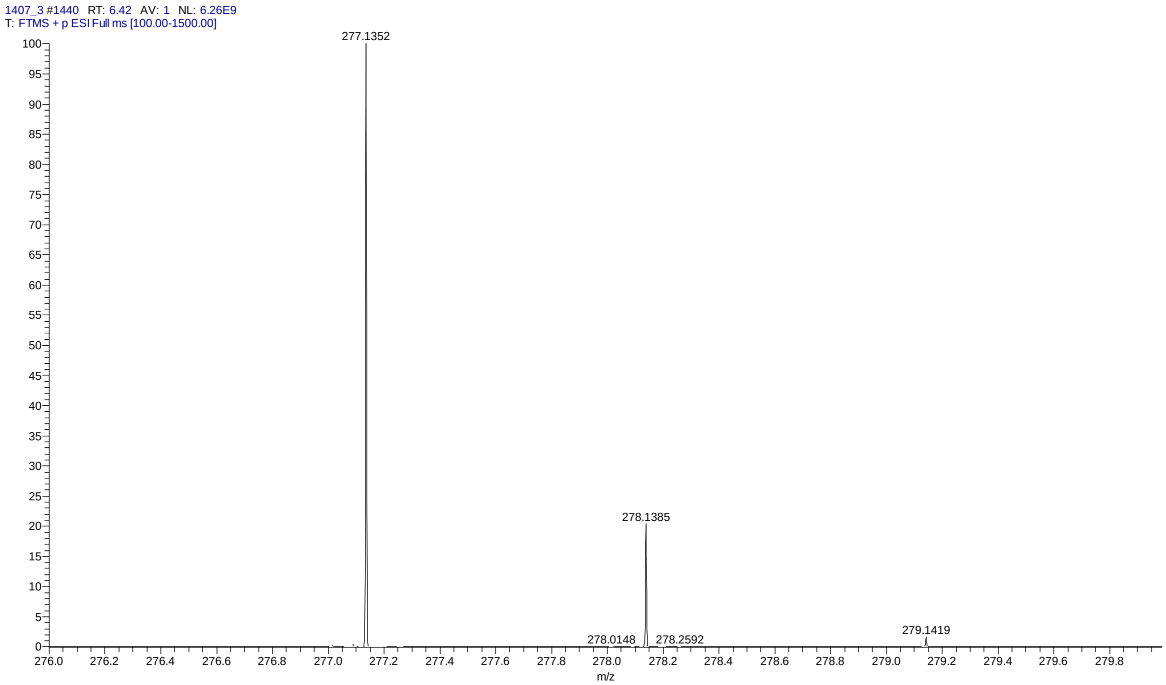

Figure S93: HRMS ESI spectrum of compound *trans*-**4e**.

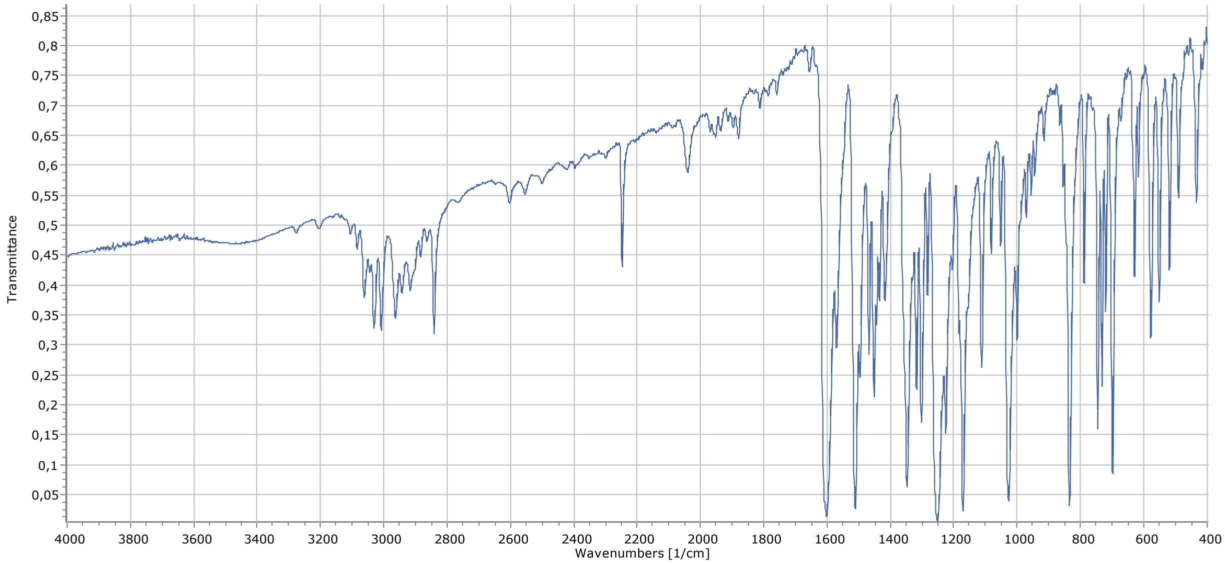

Figure S94: FT-IR spectrum of compound *cis*-**4e** (KBr).

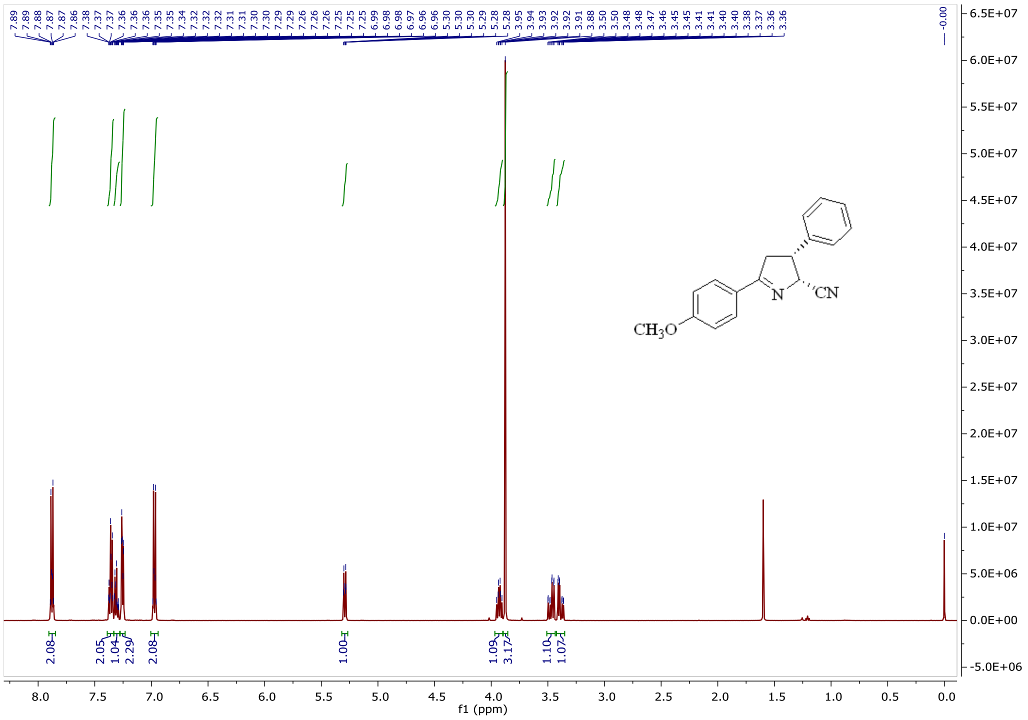

Figure S95: ^{1}H-NMR spectrum of compound *cis*-**4e** ($CDCl_3$).

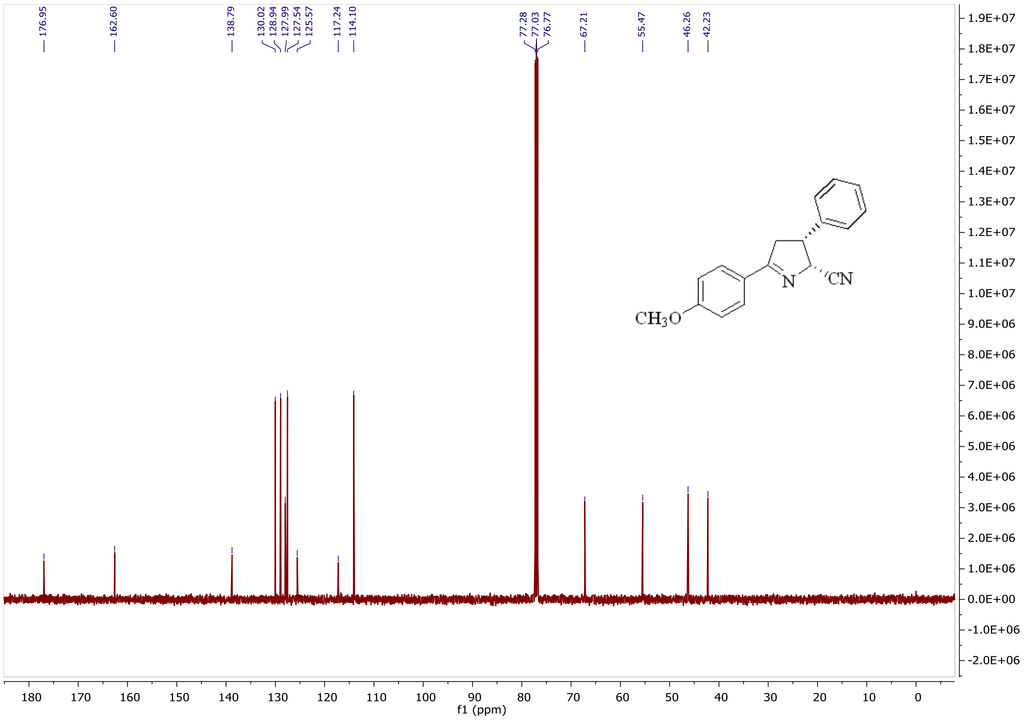

Figure S96: ^{13}C NMR spectrum of compound *cis*-**4e** ($CDCl_3$).

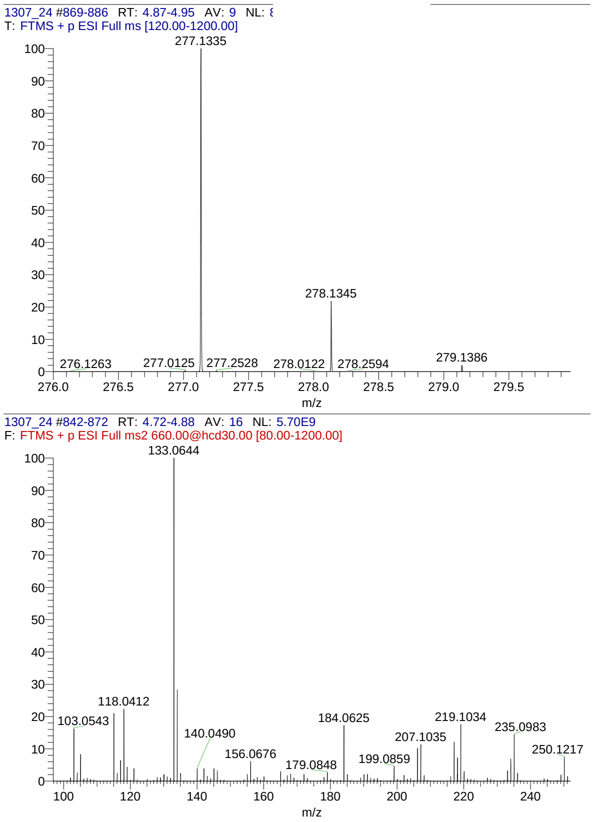

Figure S97: HRMS ESI and HRMS ESI-MS/MS spectrum of compound *cis*-**4e**.

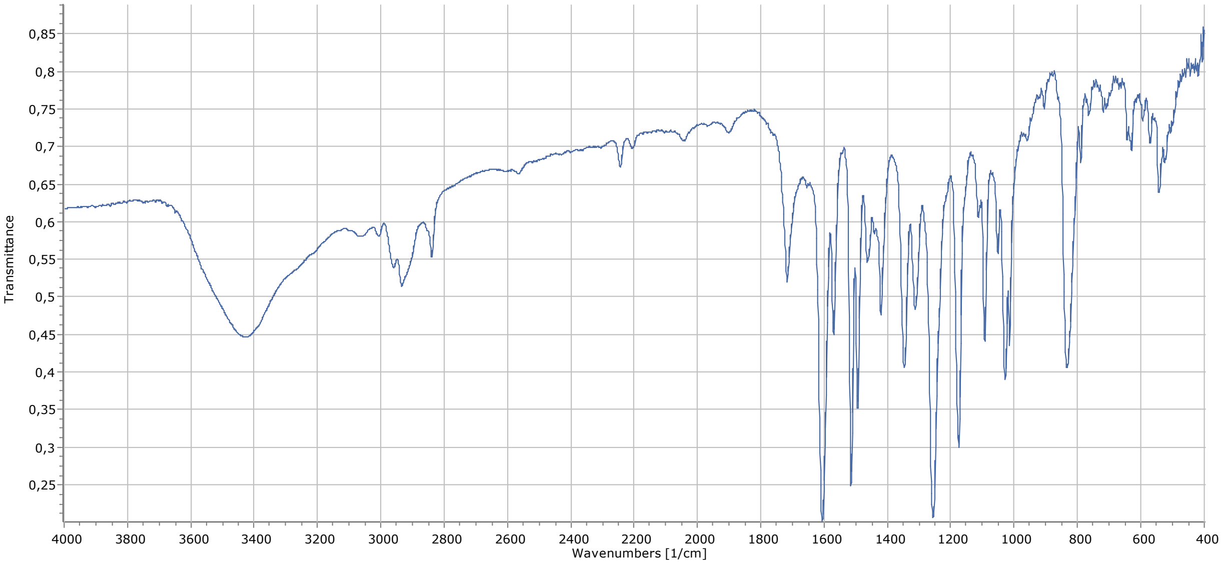

Figure S98: FT-IR spectrum of compound *trans*-**4f** (KBr).

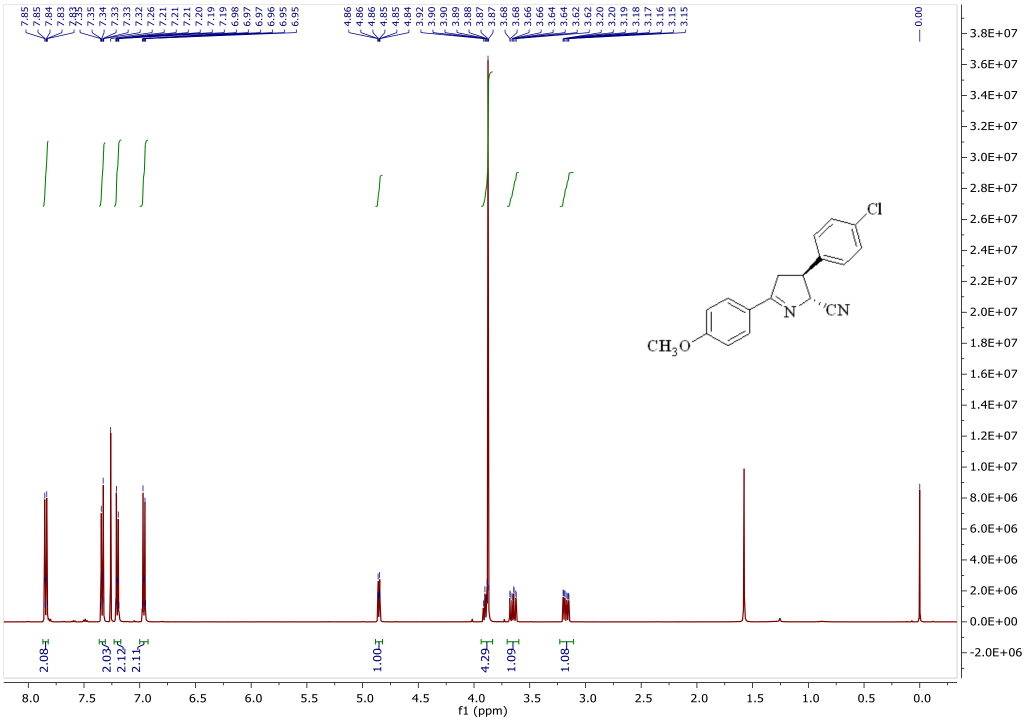

Figure S99: ^{1}H-NMR spectrum of compound *trans*-**4f** ($CDCl_3$).

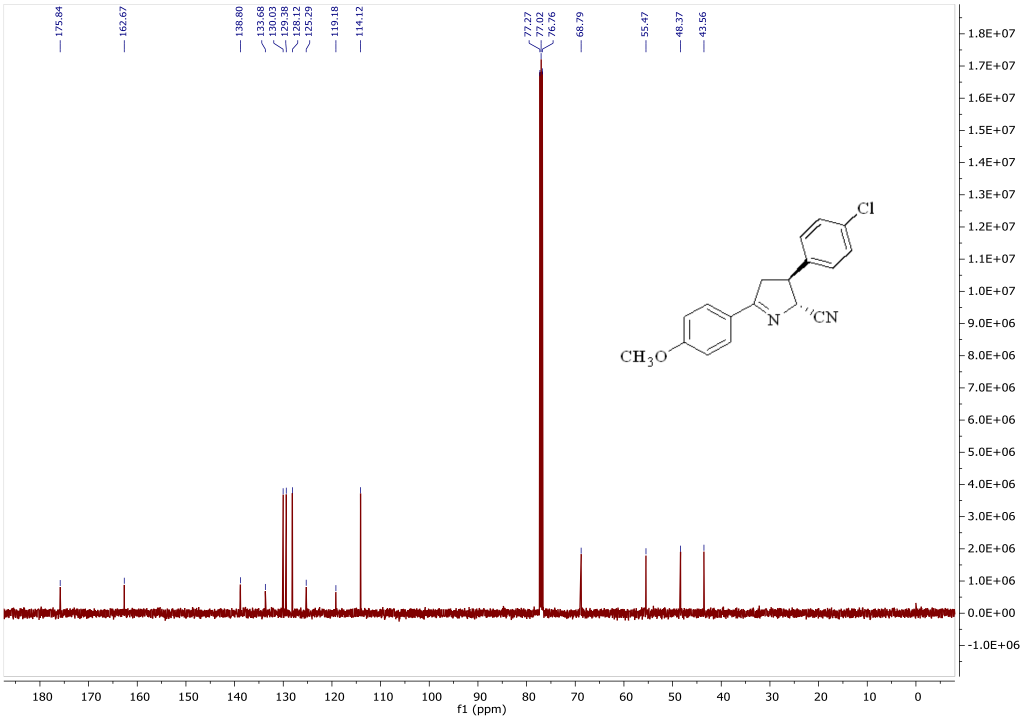

Figure S100: ^{13}C NMR spectrum of compound *trans*-**4f** ($CDCl_3$).

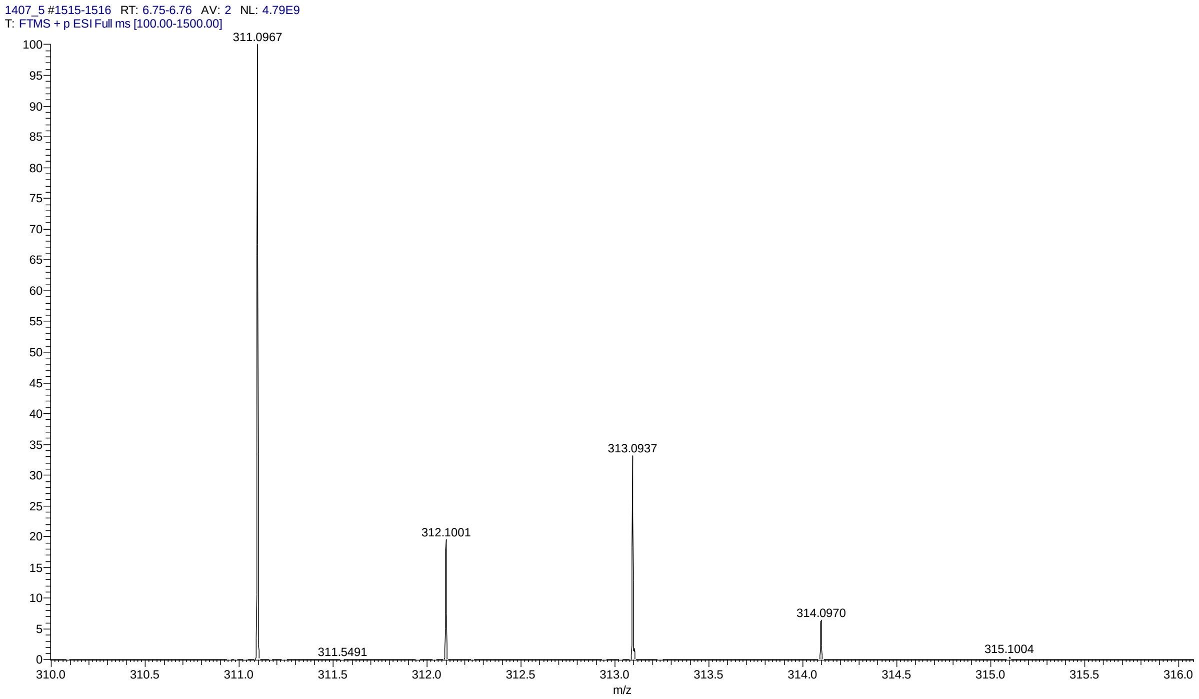

Figure S101: HRMS ESI spectrum of compound *trans*-**4f**.

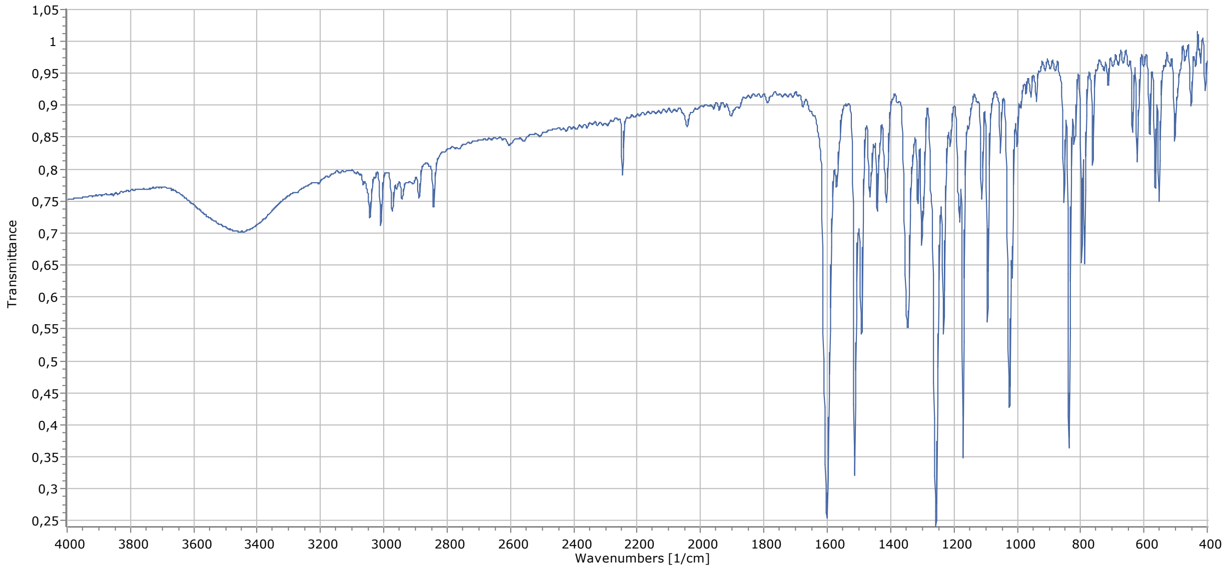

Figure S102: FT-IR spectrum of compound *cis*-**4f** (KBr).

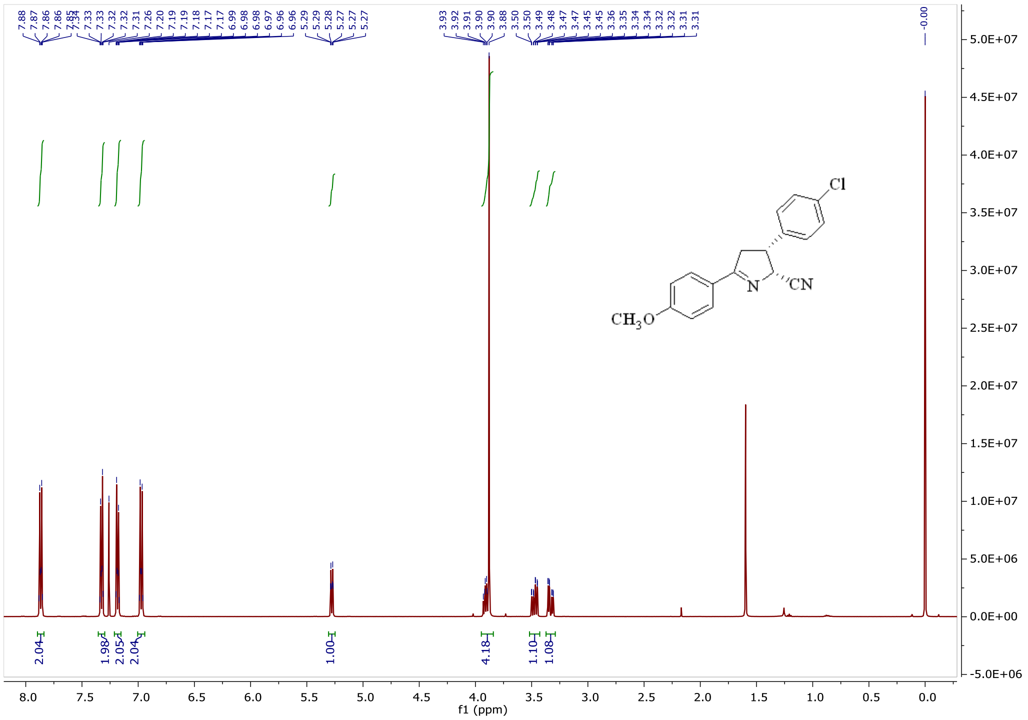

Figure S103: ^{1}H-NMR spectrum of compound *cis*-**4f** ($CDCl_3$).

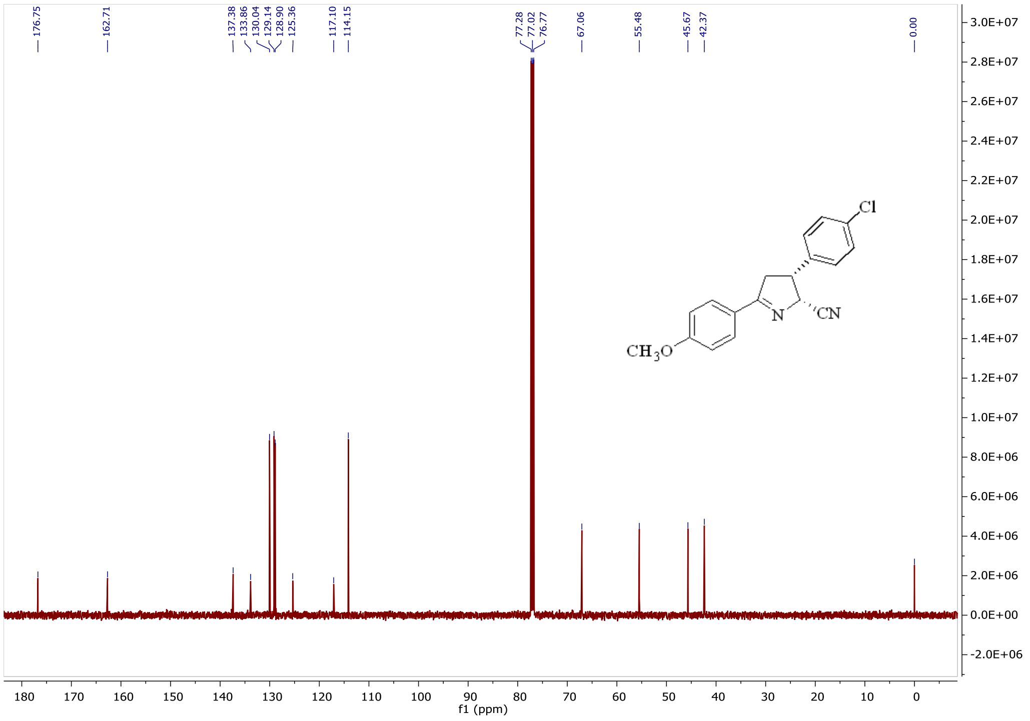

Figure S104: ^{13}C NMR spectrum of compound *cis*-**4f** ($CDCl_3$).

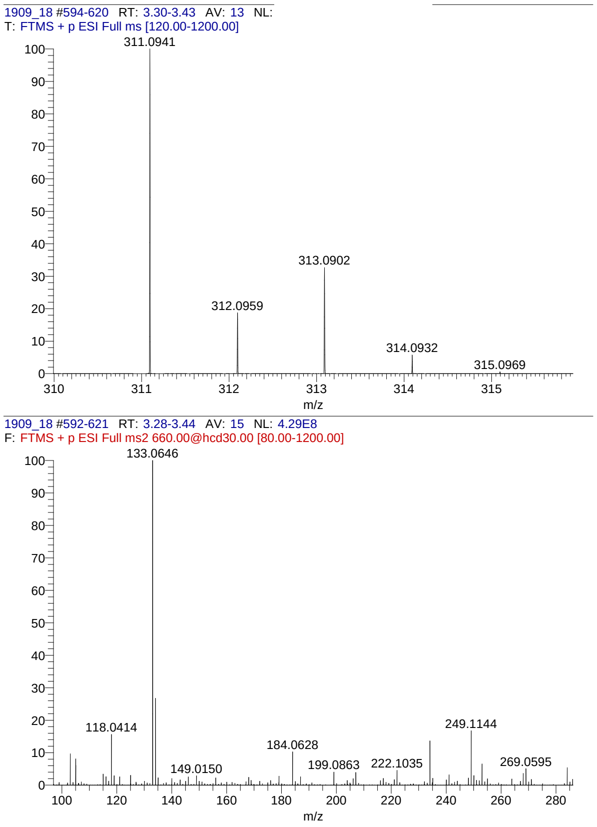

Figure S105: HRMS ESI and HRMS ESI-MS/MS spectrum of compound *cis*-**4f**.

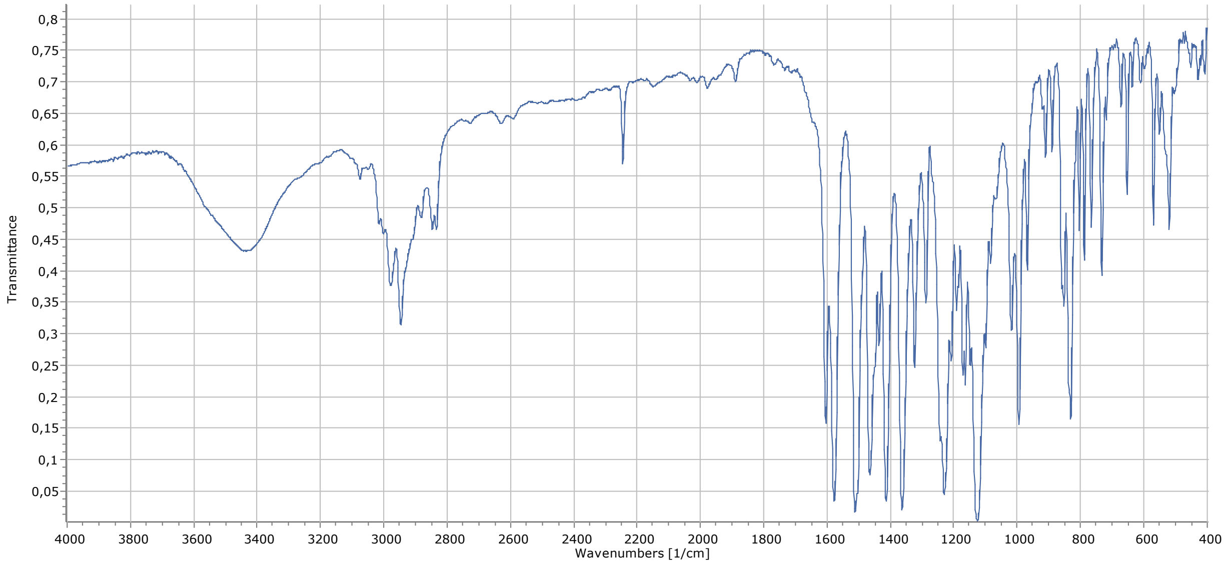

Figure S106: FT-IR spectrum of compound *trans*-**4g** (KBr).

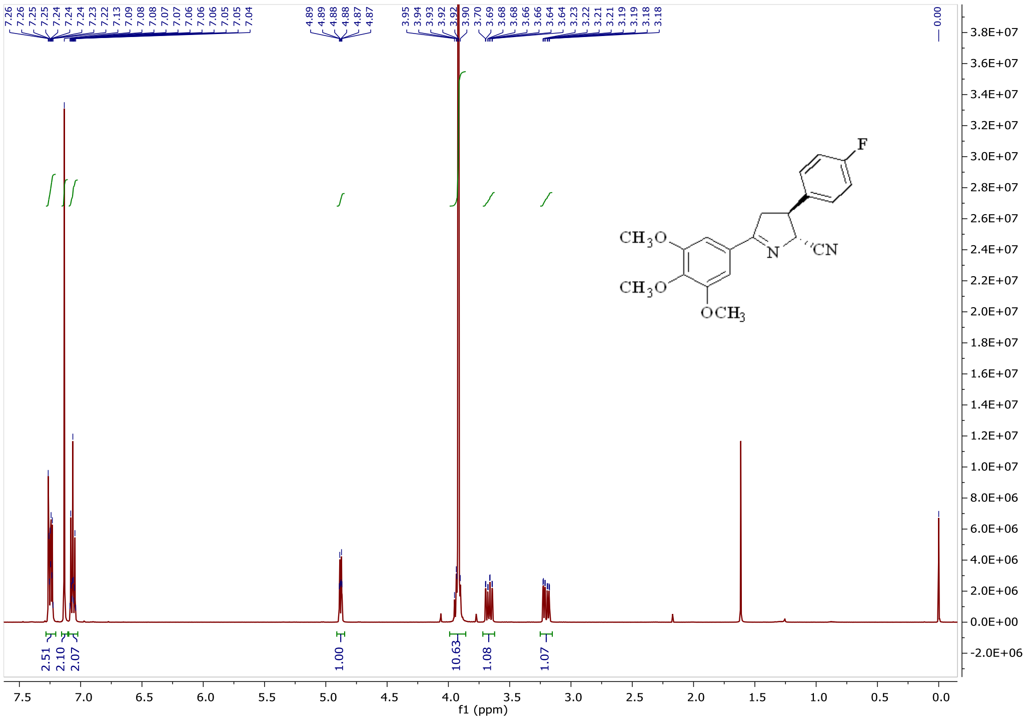

Figure S107: ^{1}H-NMR spectrum of compound *trans*-**4g** ($CDCl_3$).

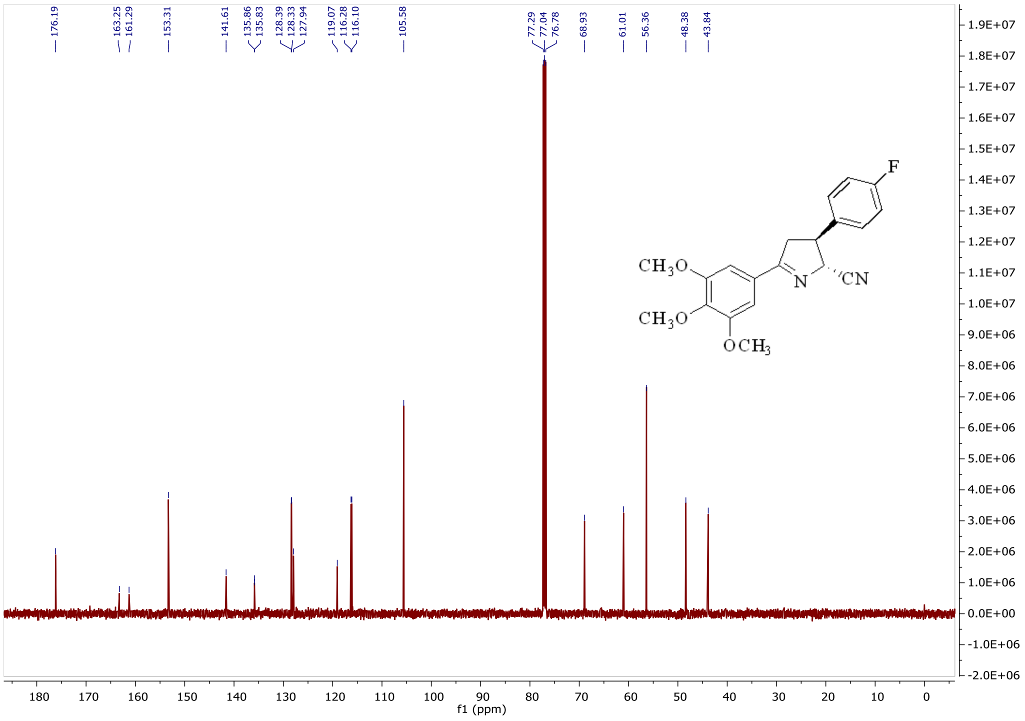

Figure S108: ^{13}C NMR spectrum of compound *trans*-**4g** ($CDCl_3$).

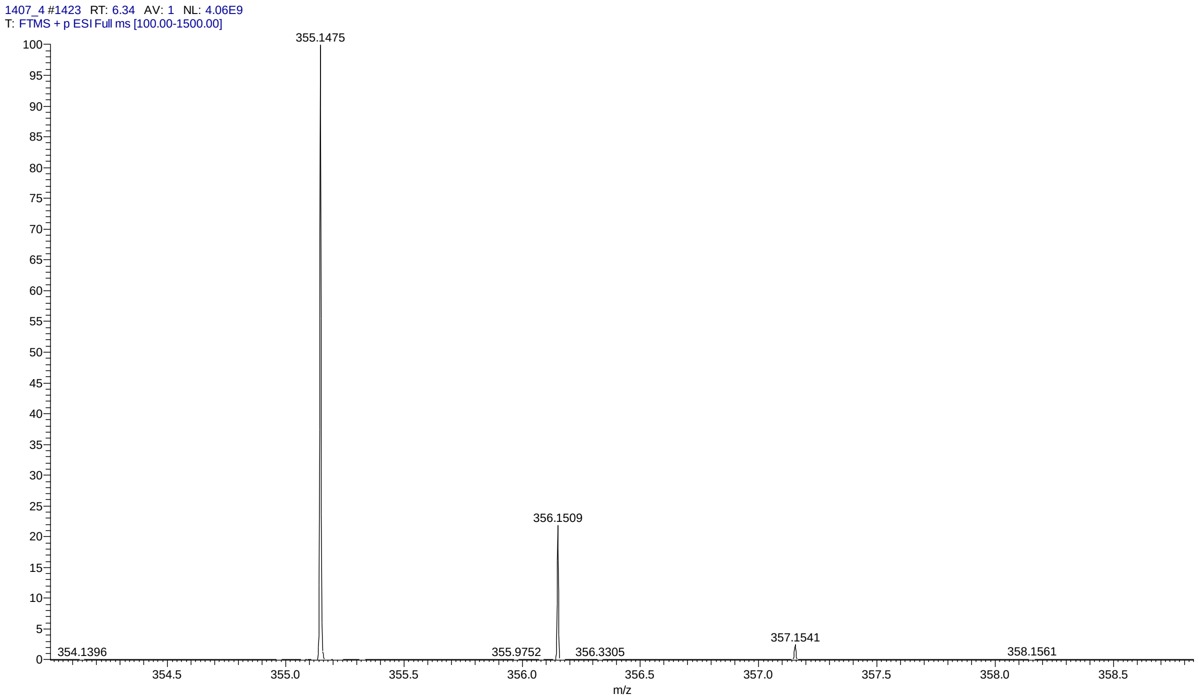

Figure S109: HRMS ESI spectrum of compound *trans*-**4g**.

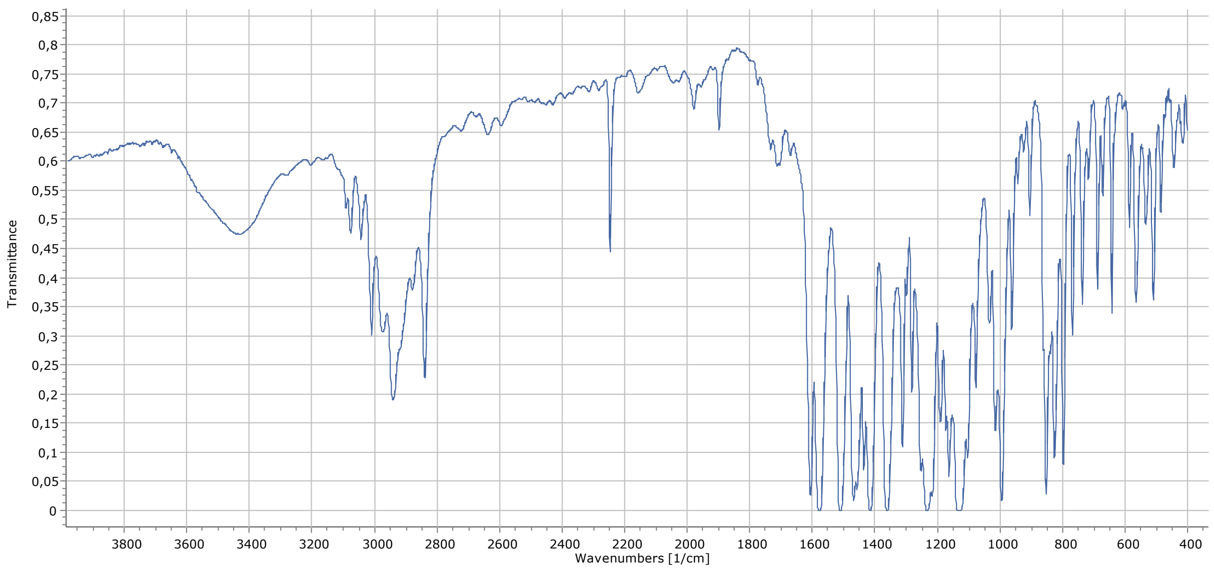

Figure S110: FT-IR spectrum of compound *cis*-**4g** (KBr).

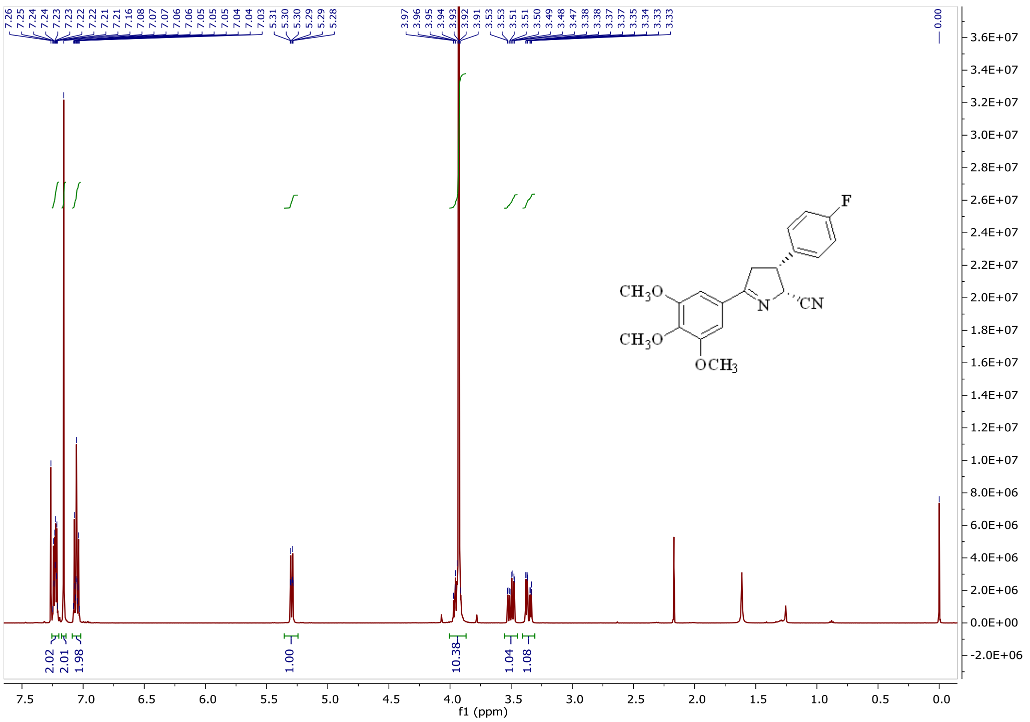

Figure S111: ^{1}H-NMR spectrum of compound *cis*-**4g** ($CDCl_3$).

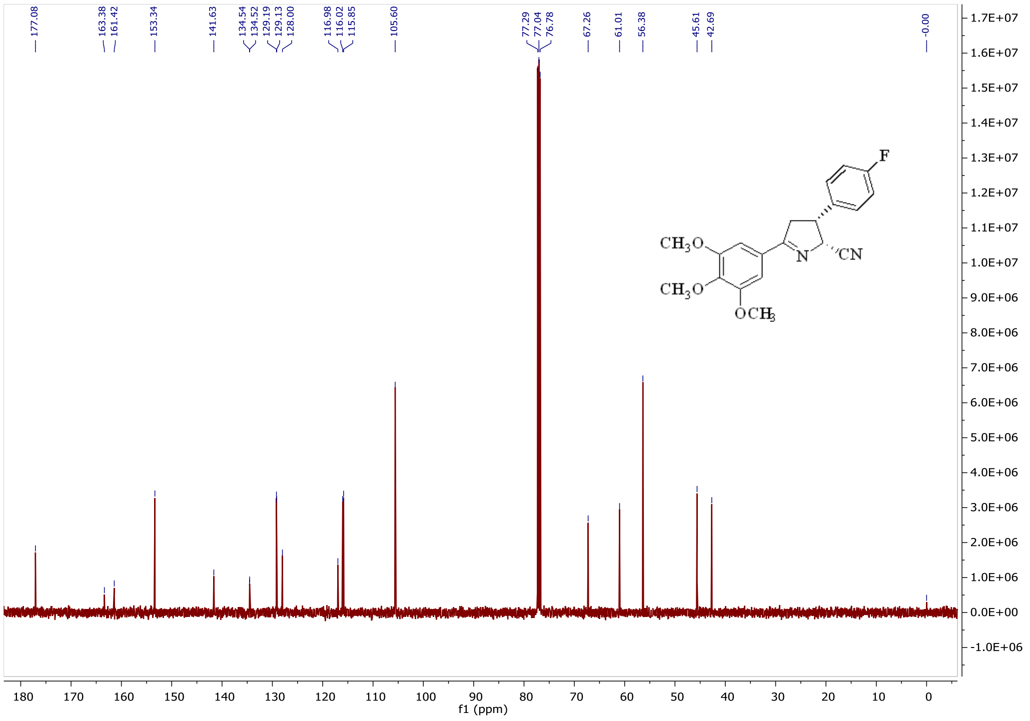

Figure S112: ^{13}C NMR spectrum of compound *cis*-**4g** ($CDCl_3$).

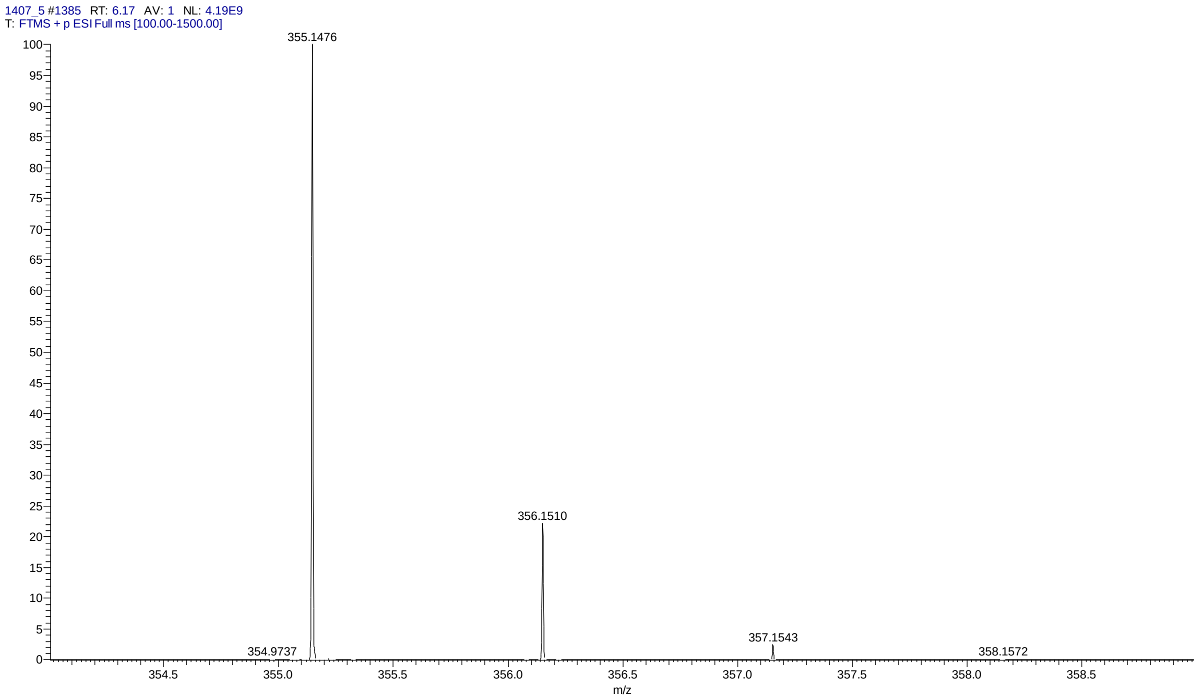

Figure S113: HRMS ESI spectrum of compound *cis*-**4g**.

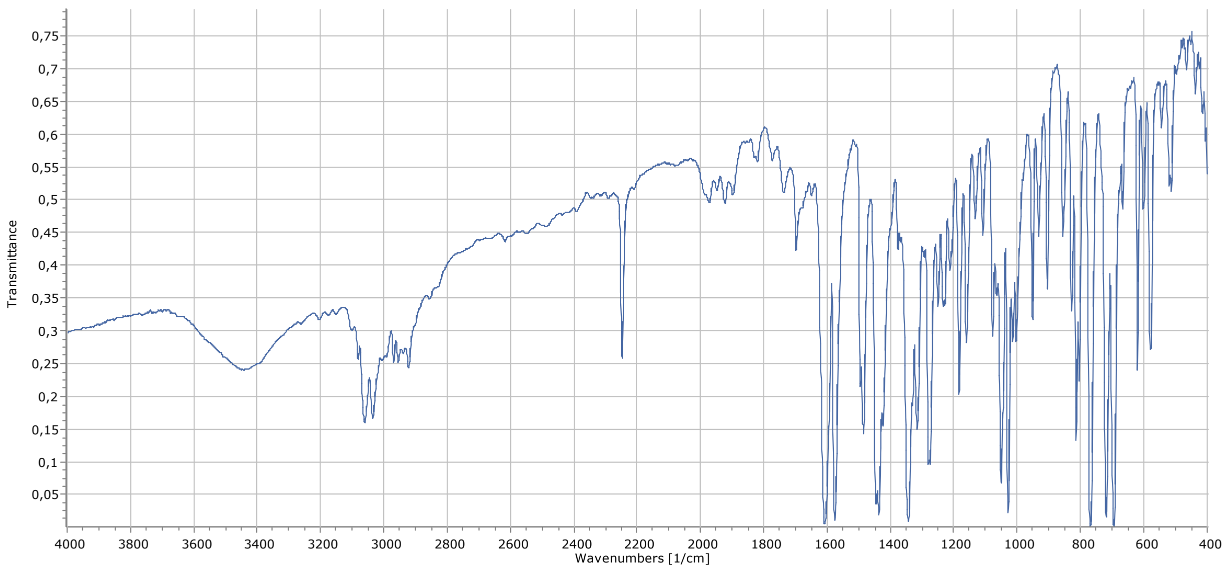

Figure S114: FT-IR spectrum of compound *trans*-**4h** (KBr).

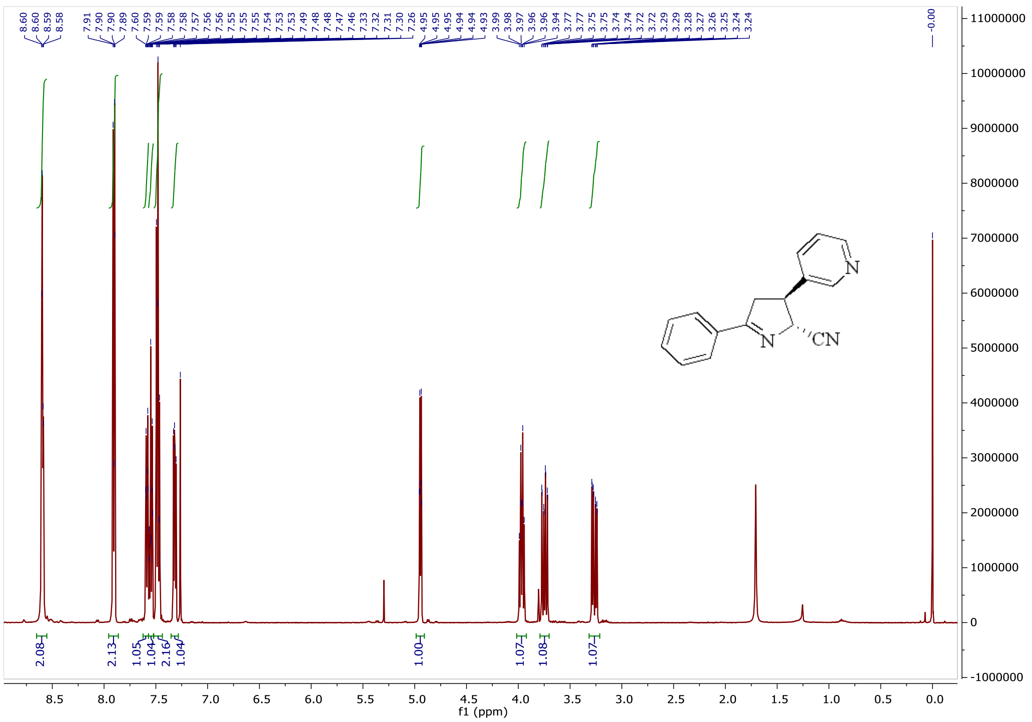

Figure S115: ^{1}H-NMR spectrum of compound *trans*-**4h** ($CDCl_3$).

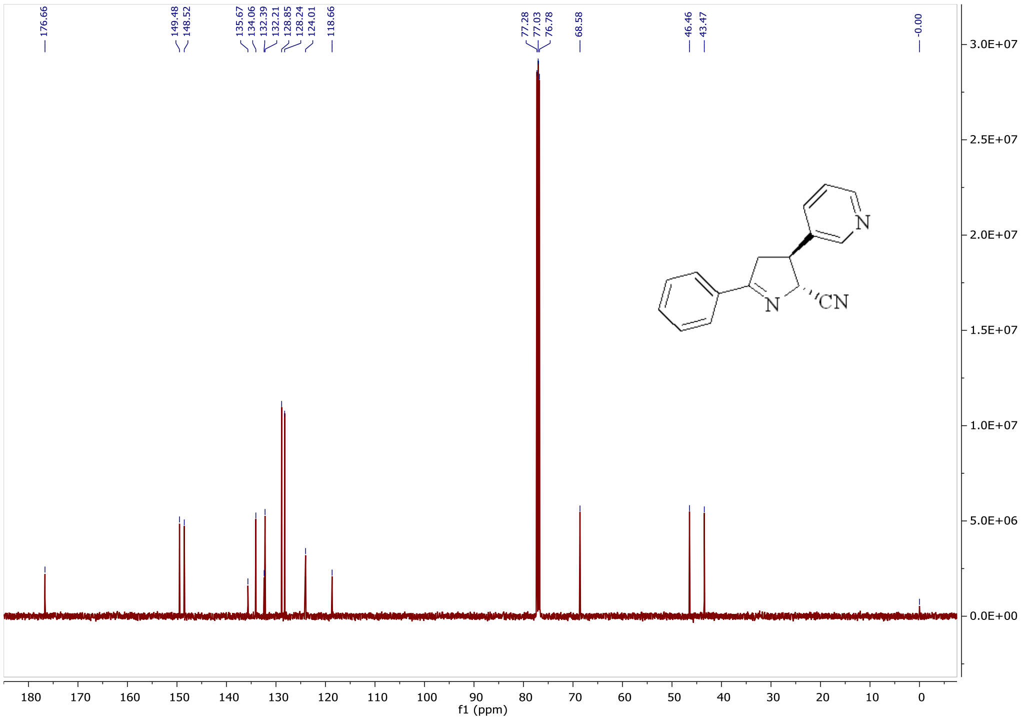

Figure S116: ^{13}C NMR spectrum of compound *trans*-**4h** ($CDCl_3$).

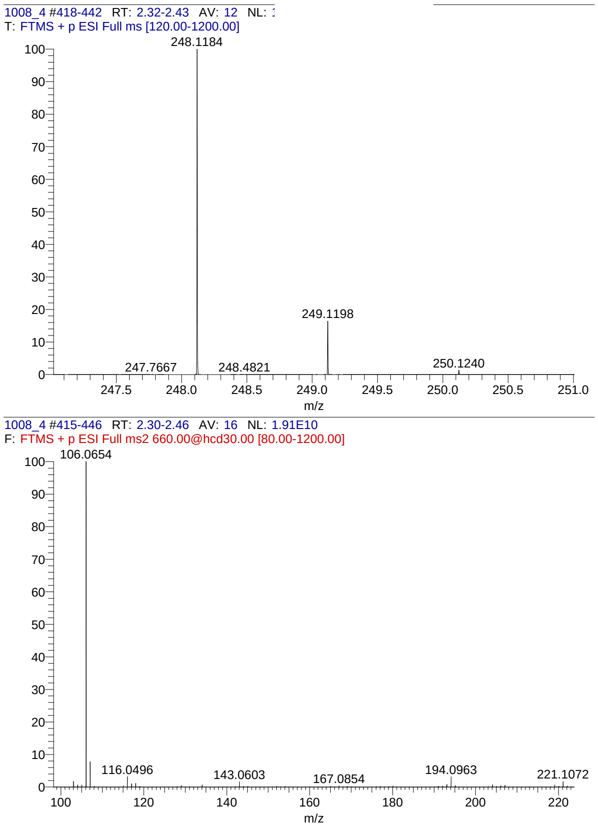

Figure S117: HRMS ESI and HRMS ESI-MS/MS spectrum of compound *trans*-**4h**.

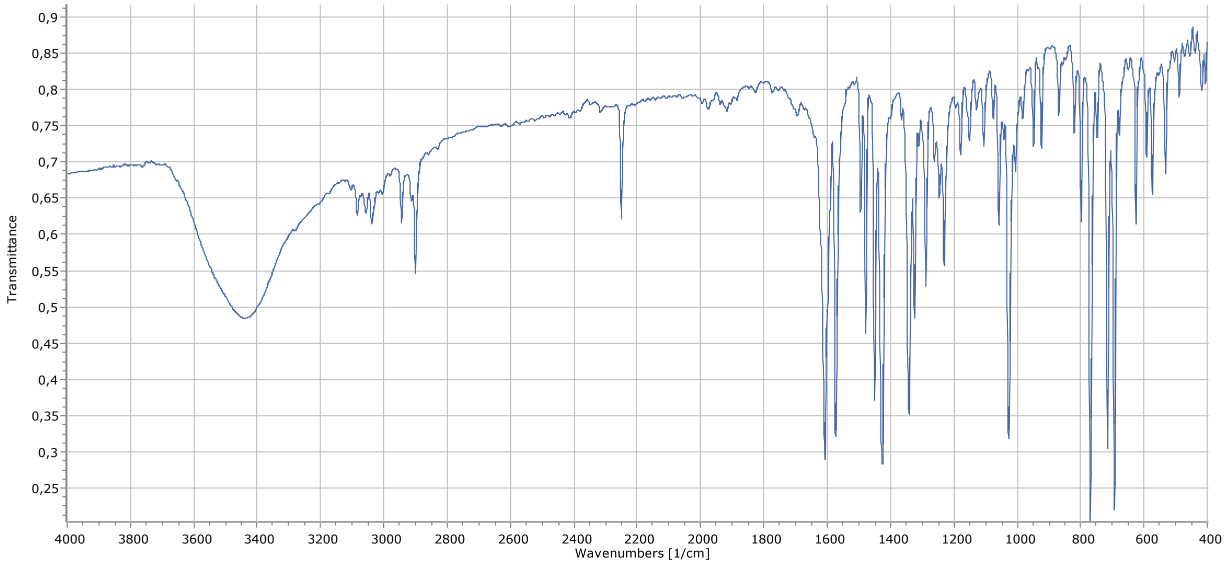

Figure S118: FT-IR spectrum of compound *cis*-**4h** (KBr).

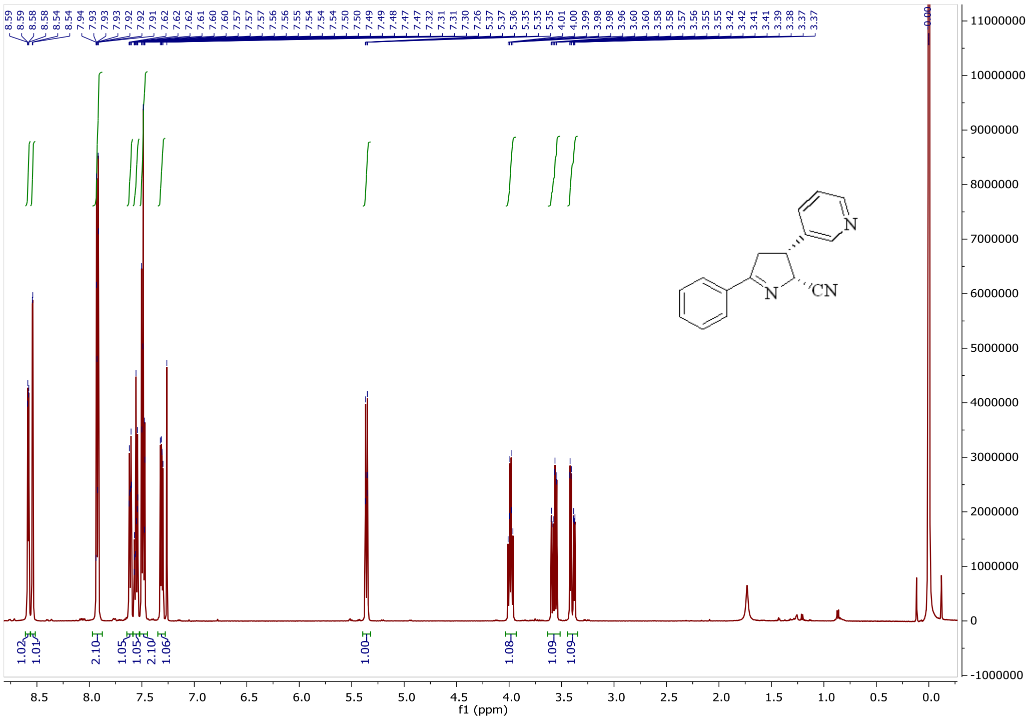

Figure S119: ^{1}H-NMR spectrum of compound *cis*-**4h** ($CDCl_3$).

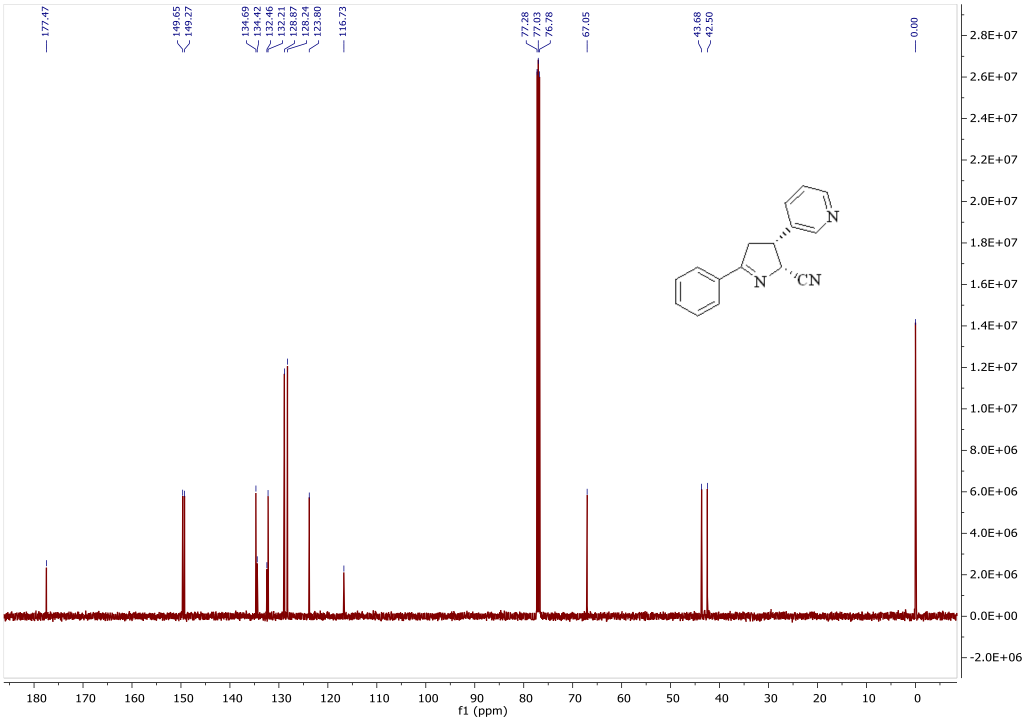

Figure S120: ^{13}C NMR spectrum of compound *cis*-**4h** ($CDCl_3$).

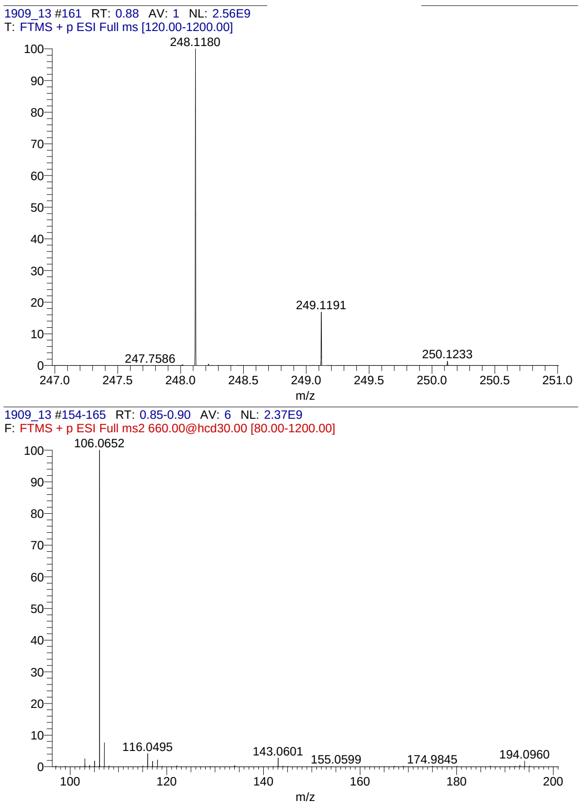

Figure S121: HRMS ESI and HRMS ESI-MS/MS spectrum of compound *cis*-**4h**.

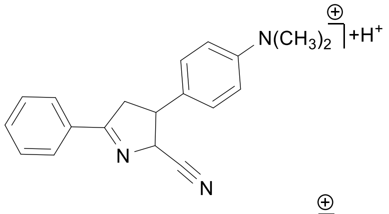

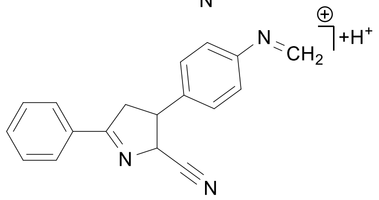

m/z = 274

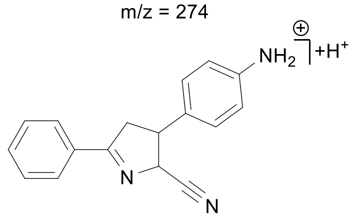

m/z = 235

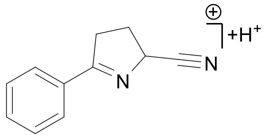

m/z = 171

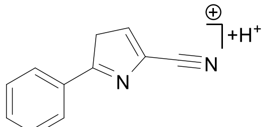

m/z = 169

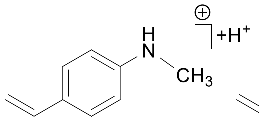

m/z = 134

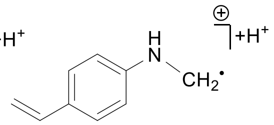

m/z = 133

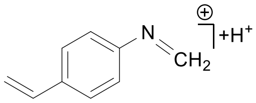

m/z = 132

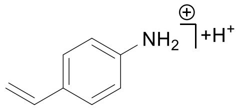

m/z = 120

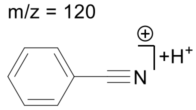

m/z = 104

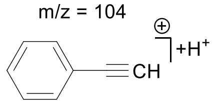

m/z = 103

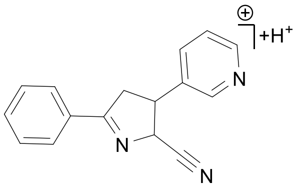

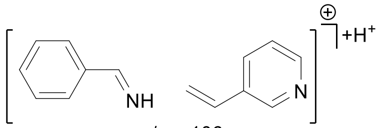

m/z = 106

Figure S122: Proposed structures of the most abundant fragment ions observed in a MS/MS spectra of *trans*-**4c**, *trans*-**4h** and *cis*-**4h**.

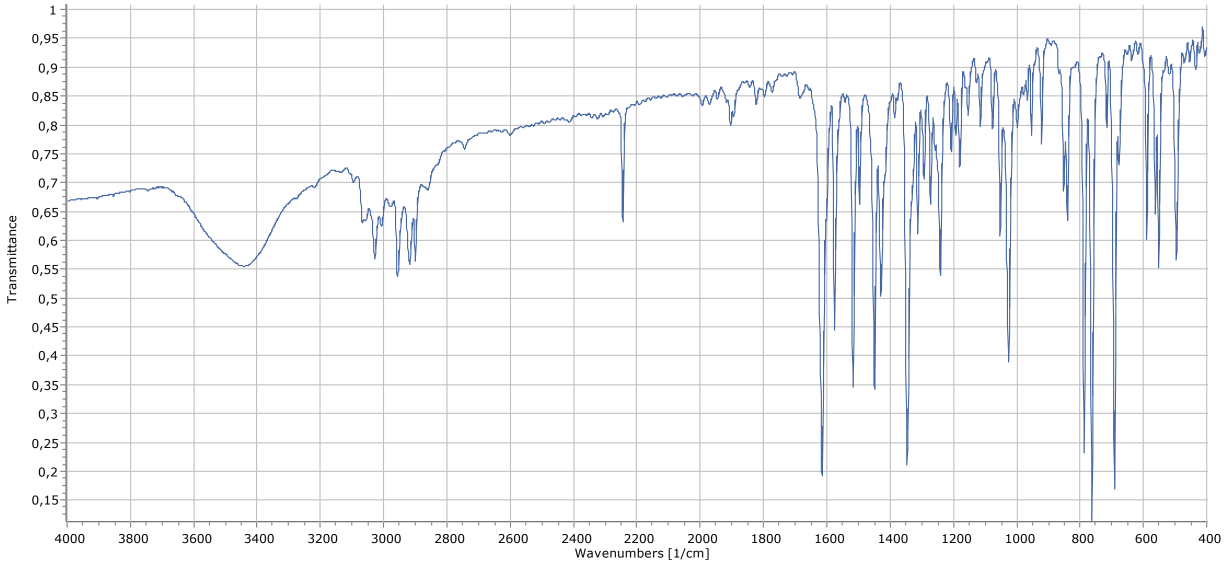

Figure S123: FT-IR spectrum of compound *cis*-**4i** (KBr).

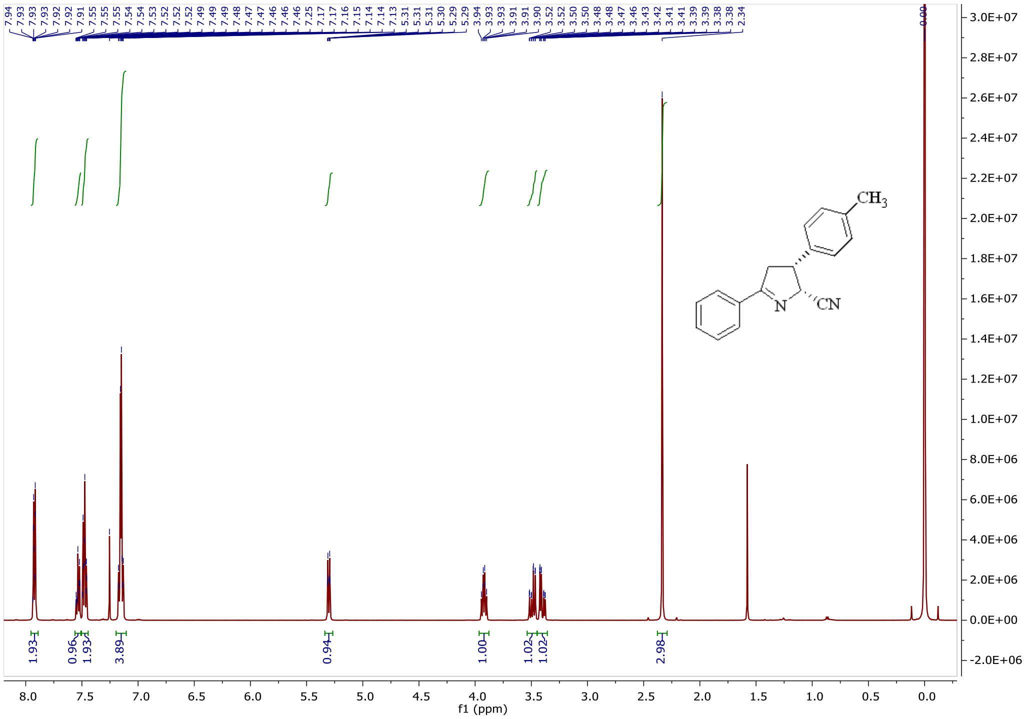

Figure S124: ^{1}H-NMR spectrum of compound *cis*-**4i** ($CDCl_3$).

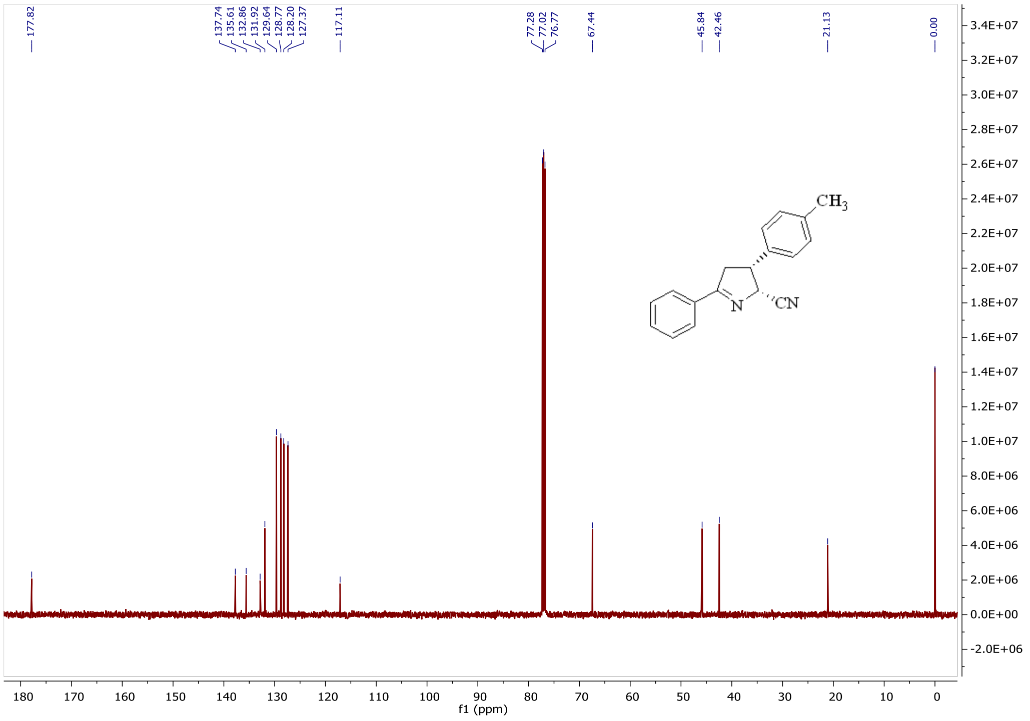

Figure S125: ^{13}C NMR spectrum of compound *cis*-**4i** ($CDCl_3$).

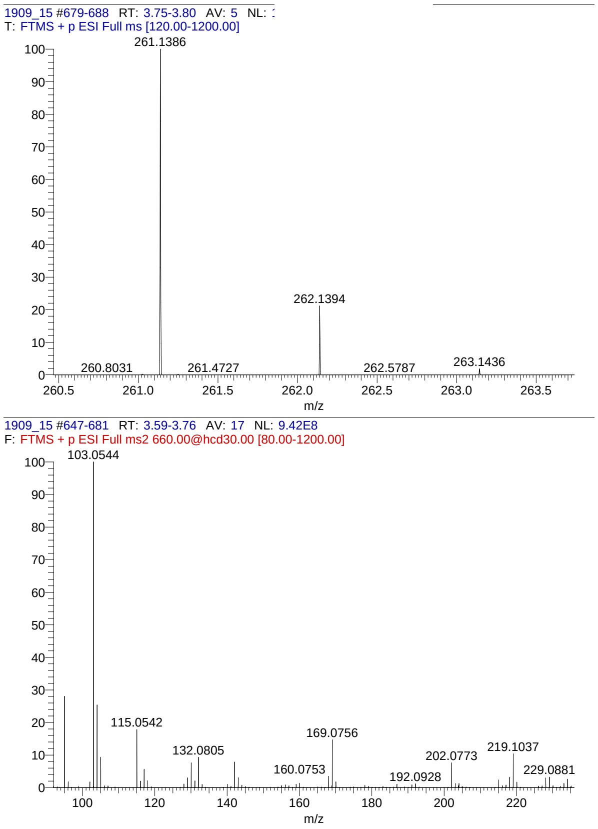

Figure S126: HRMS ESI and HRMS ESI-MS/MS spectrum of compound *cis*-**4i**.

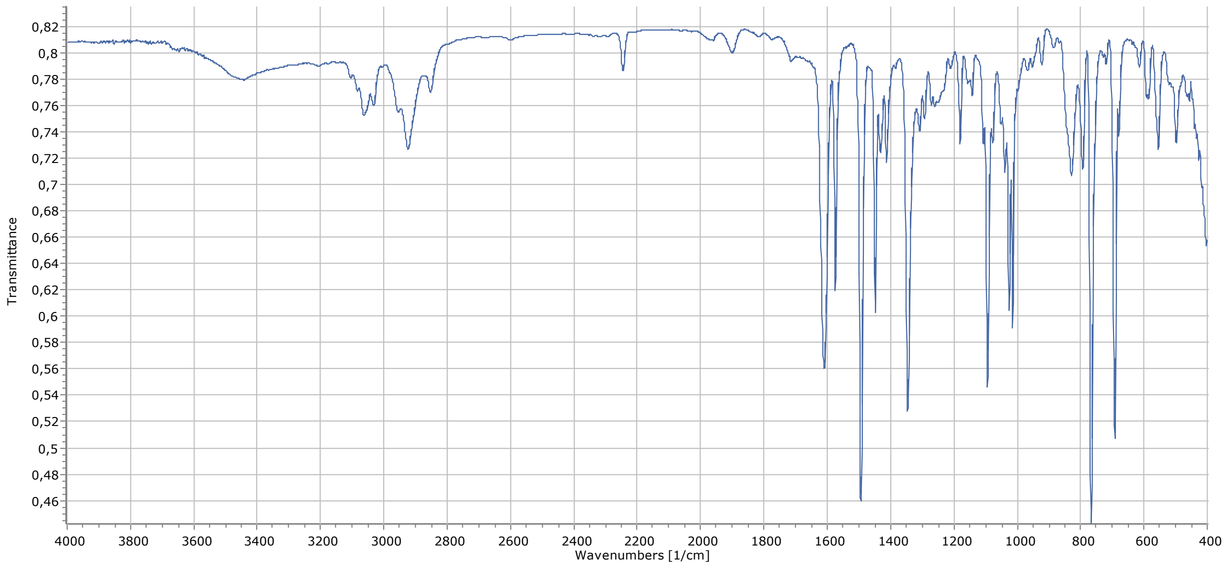

Figure S127: FT-IR spectrum of compound *cis*-**4j** (capilary layer).

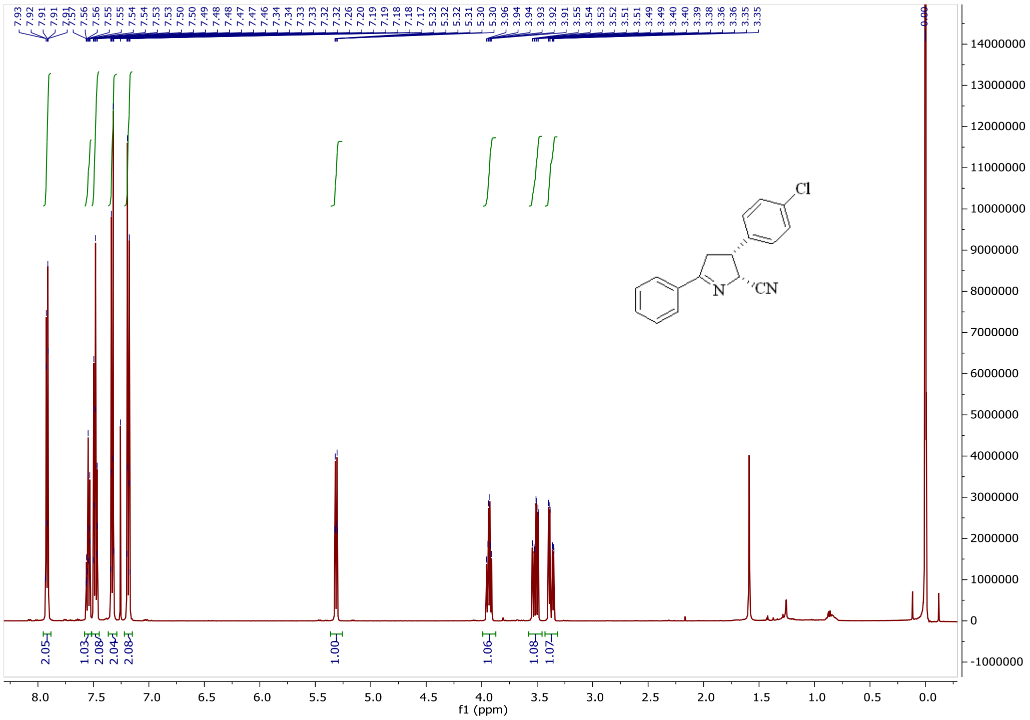

Figure S128: ^{1}H-NMR spectrum of compound *cis*-**4j** ($CDCl_3$).

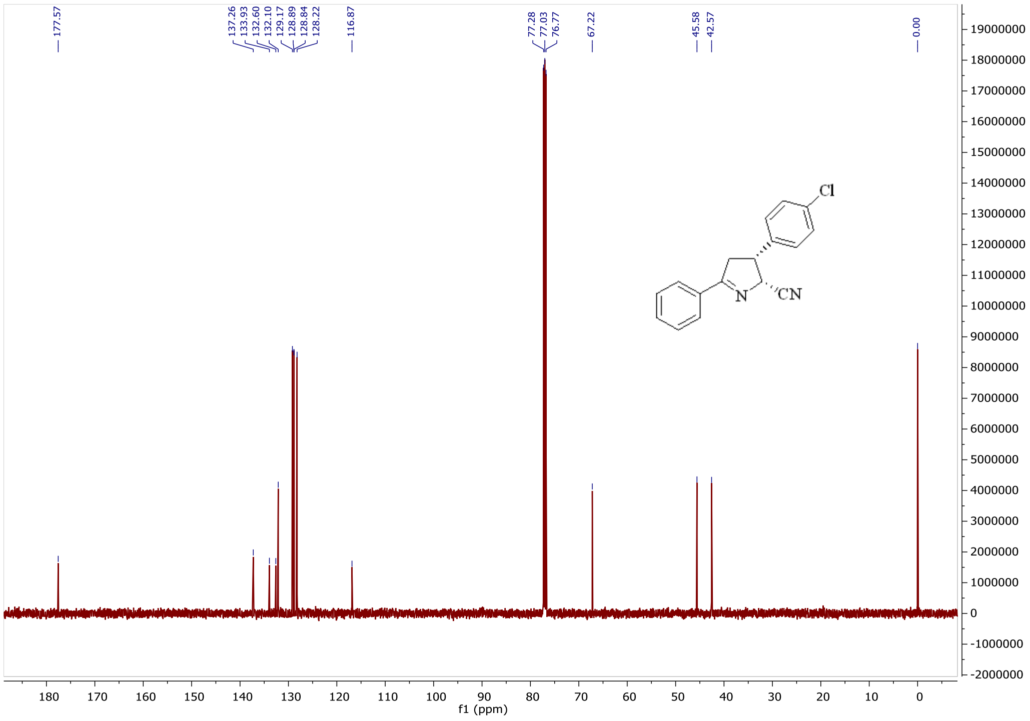

Figure S129: ^{13}C NMR spectrum of compound *cis*-**4j** ($CDCl_3$).

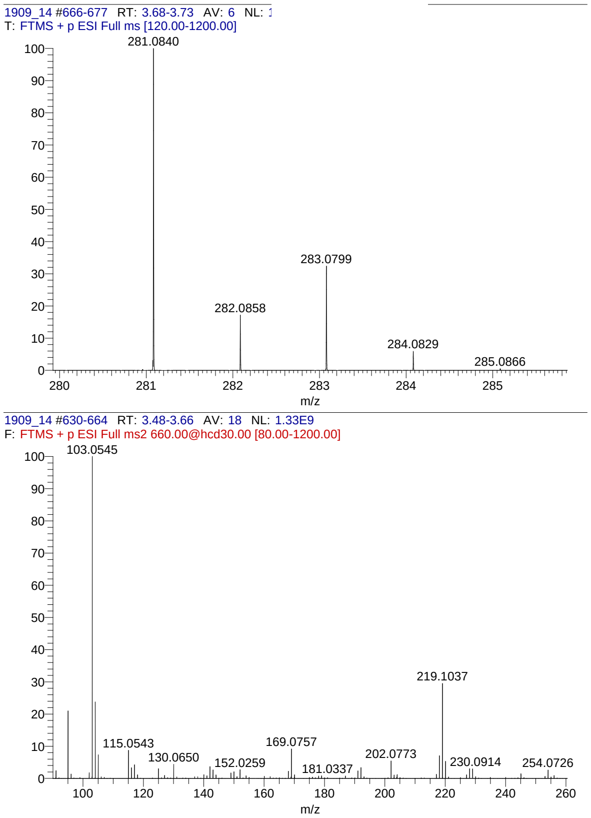

Figure S130: HRMS ESI and HRMS ESI-MS/MS spectrum of compound *cis*-**4j**.

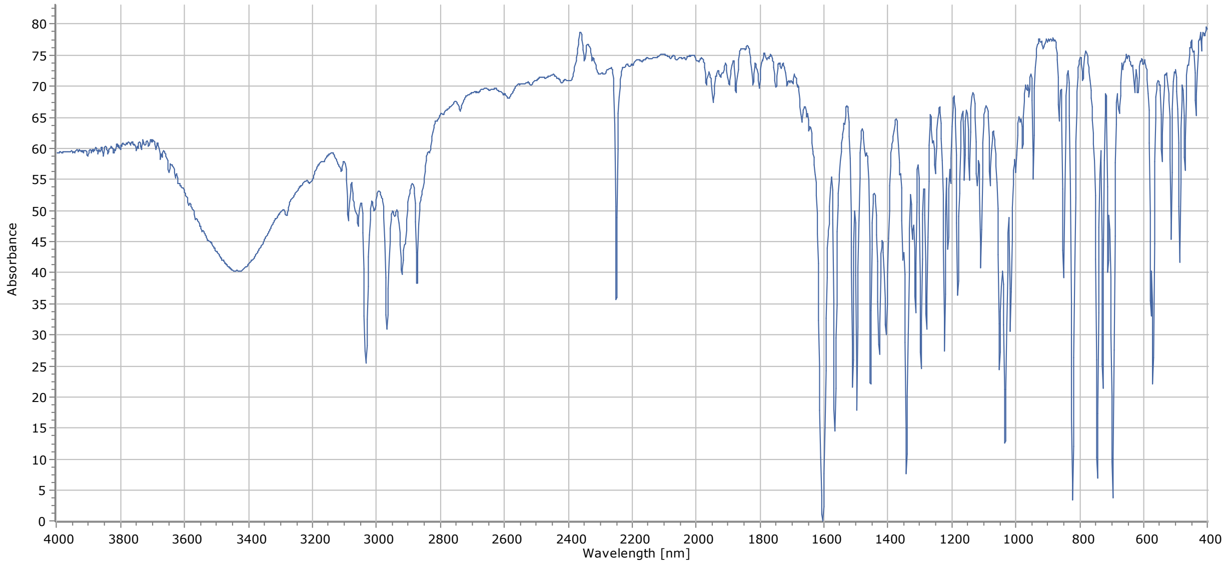

Figure S131: FT-IR spectrum of compound *cis*-**4k** (KBr).

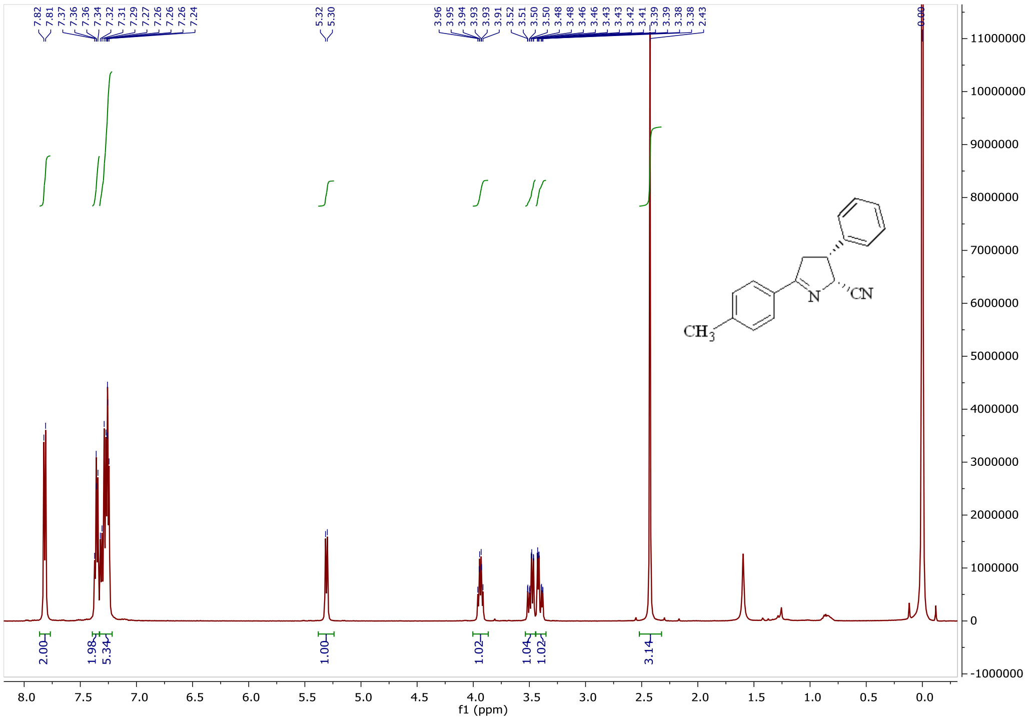

Figure S132: ^{1}H-NMR spectrum of compound *cis*-**4k** ($CDCl_3$).

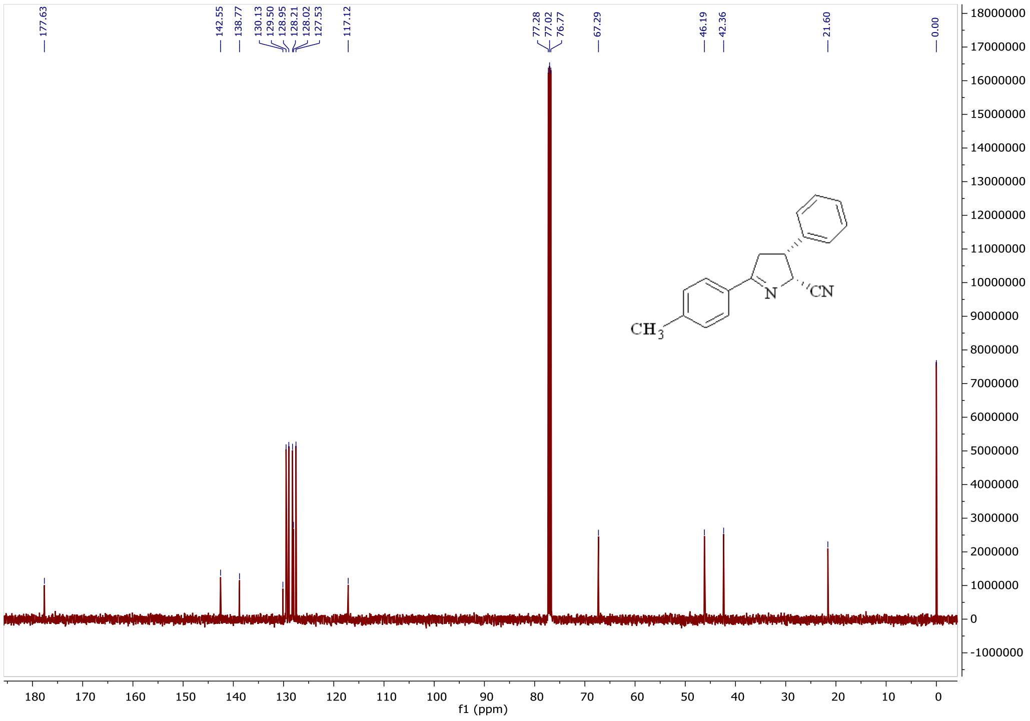

Figure S133: ^{13}C NMR spectrum of compound *cis*-**4k** ($CDCl_3$).

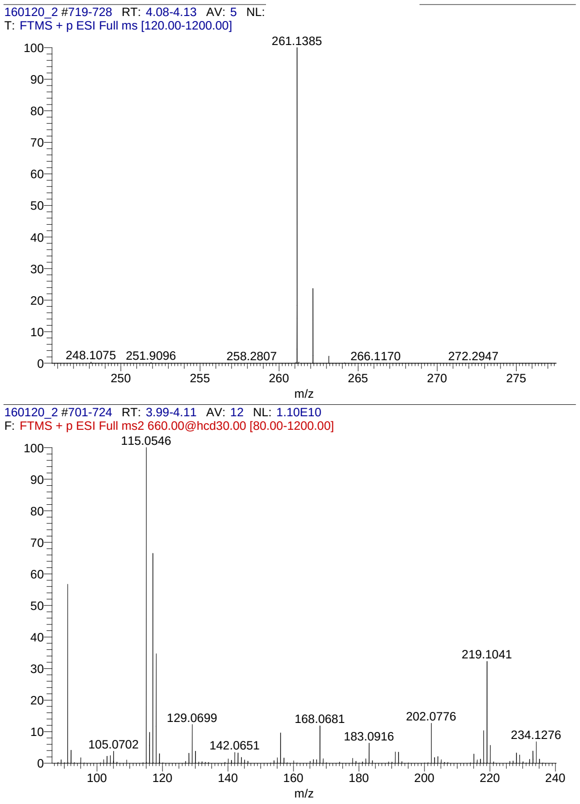

Figure S134: HRMS ESI and HRMS ESI-MS/MS spectrum of compound *cis*-**4k**.

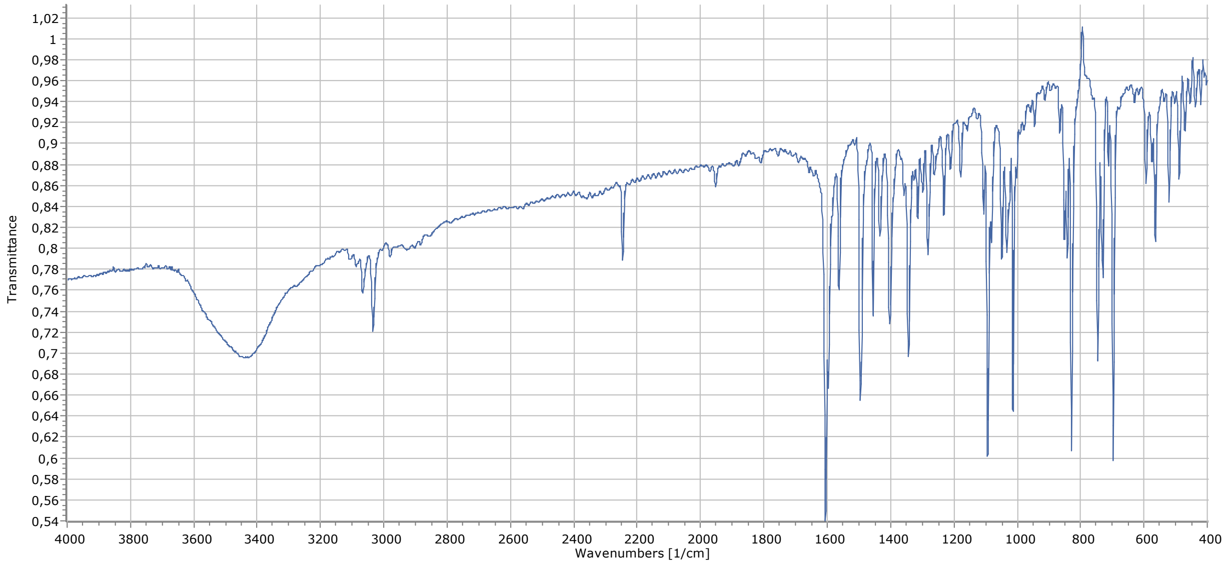

Figure S135: FT-IR spectrum of compound *cis*-**4l** (KBr).

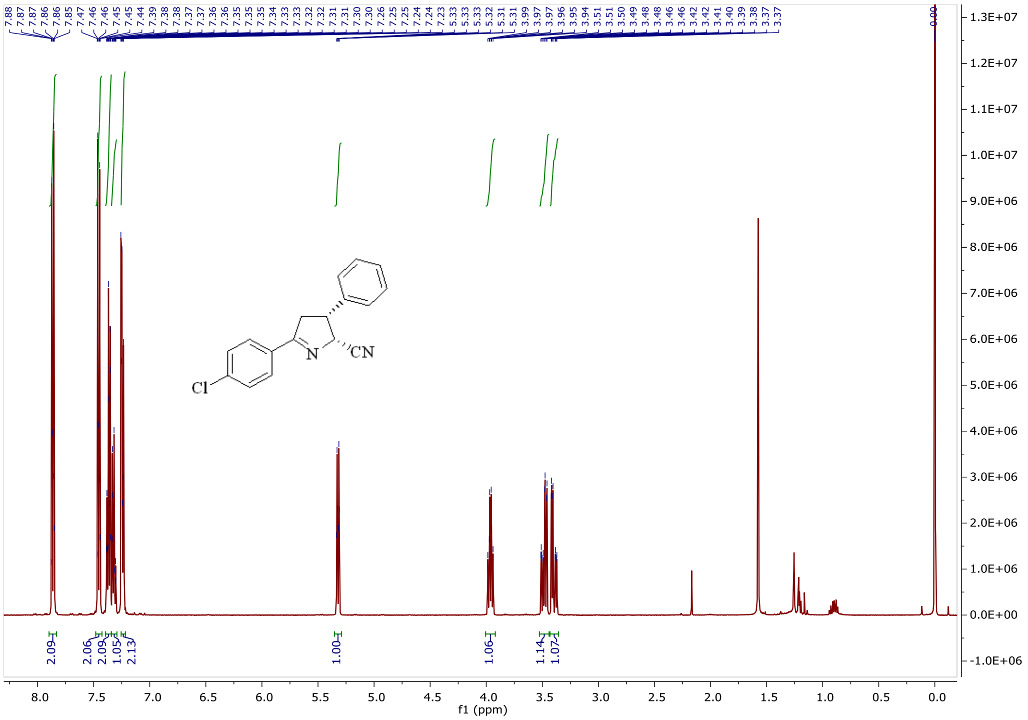

Figure S136: ^{1}H-NMR spectrum of compound *cis*-**4l** ($CDCl_3$).

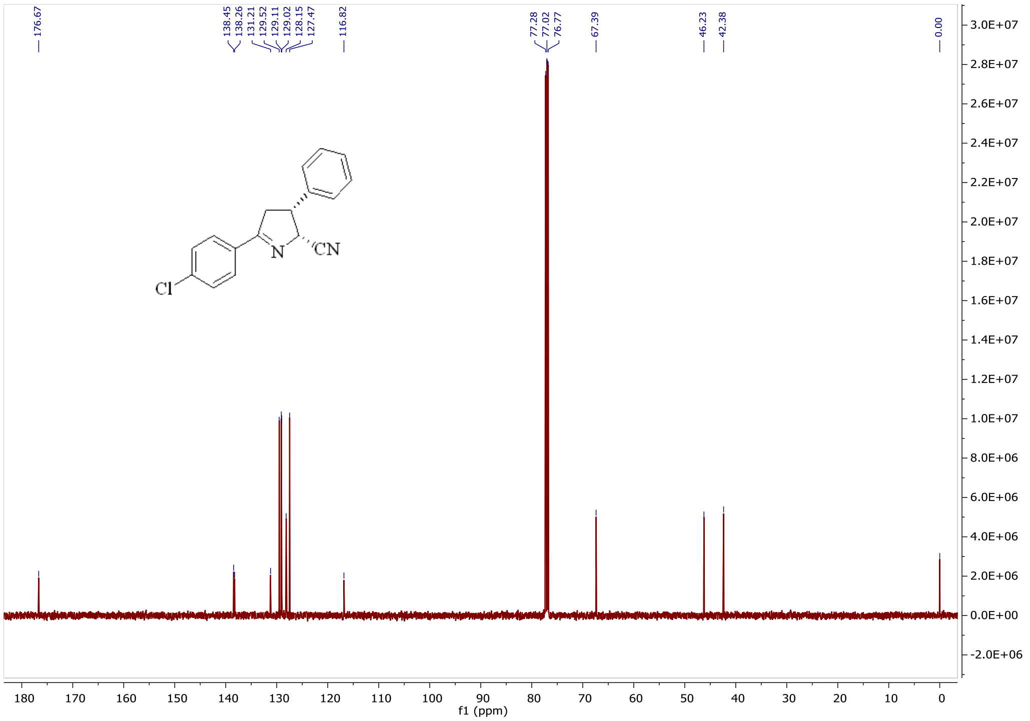

Figure S137: ^{13}C NMR spectrum of compound *cis*-**4l** (CDCl$_3$).

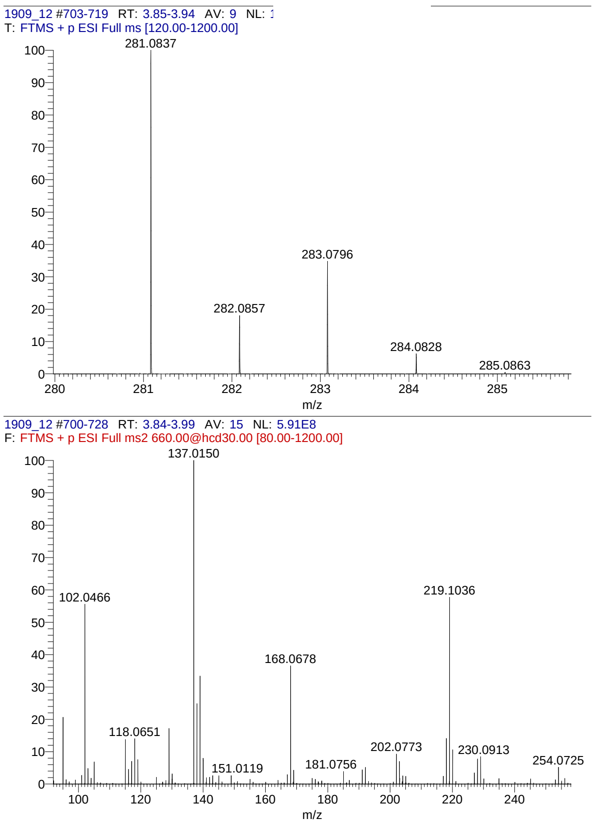

Figure S138: HRMS ESI and HRMS ESI-MS/MS spectrum of compound *cis*-**4l**.

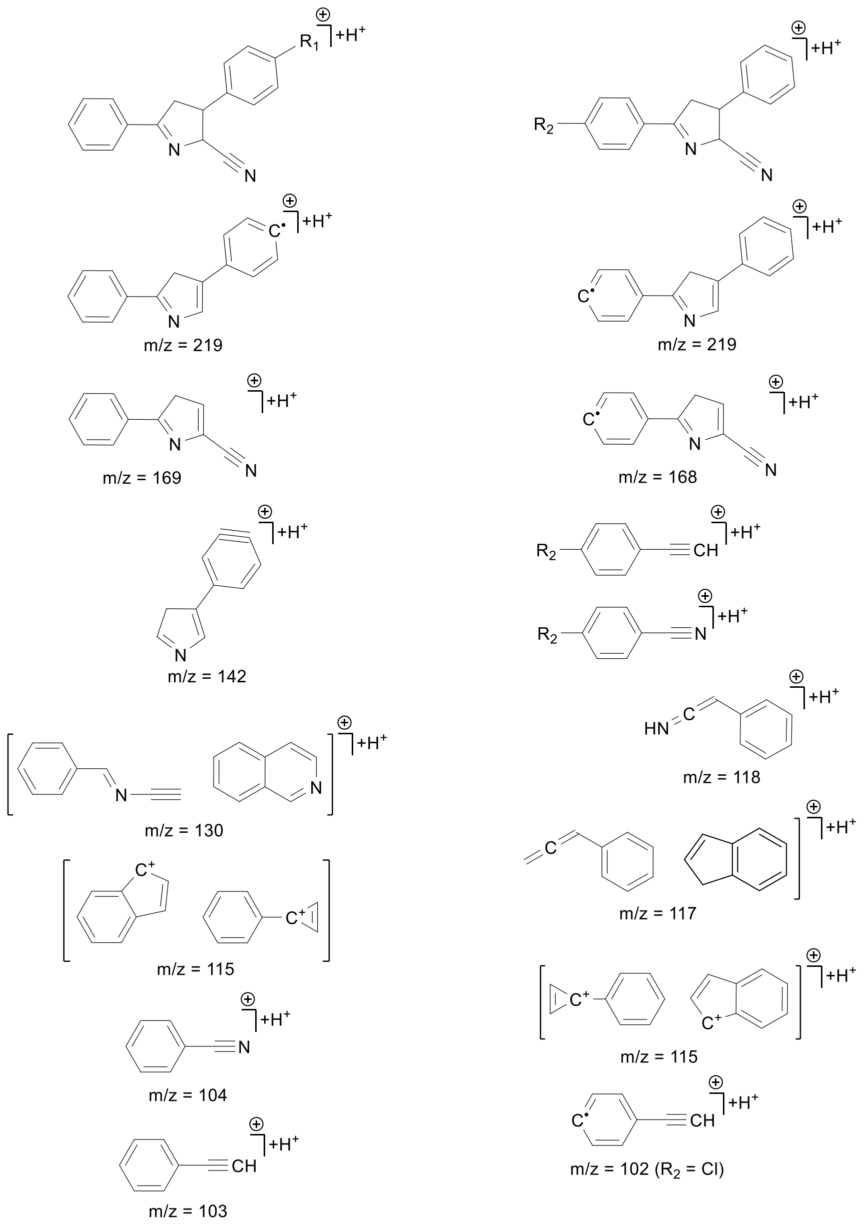

Figure S139: Proposed structures of the most abundant fragment ions observed in a MS/MS spectra of compounds *trans*-**4b**, *trans*-**4d-4g**, *cis*-**4a**, *cis*-**4b**, *cis*-**4d-4g** and *cis*-**4i-4l**.

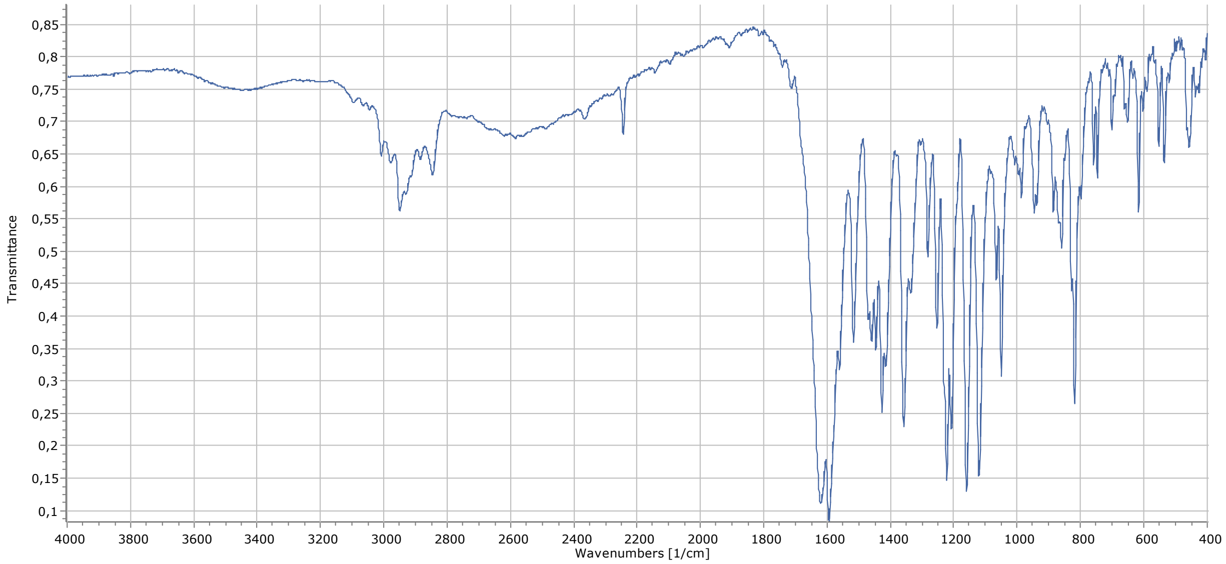

Figure S140: FT-IR spectrum of compound *trans*-**4m** (KBr).

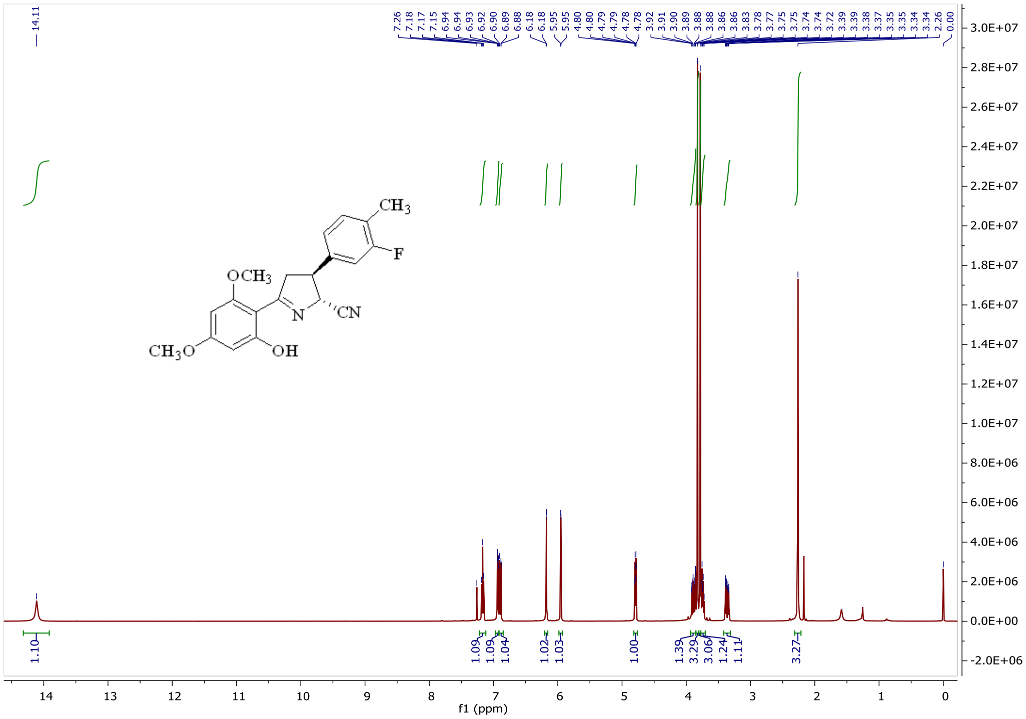

Figure S141: ^{1}H-NMR spectrum of compound *trans*-**4m** ($CDCl_3$).

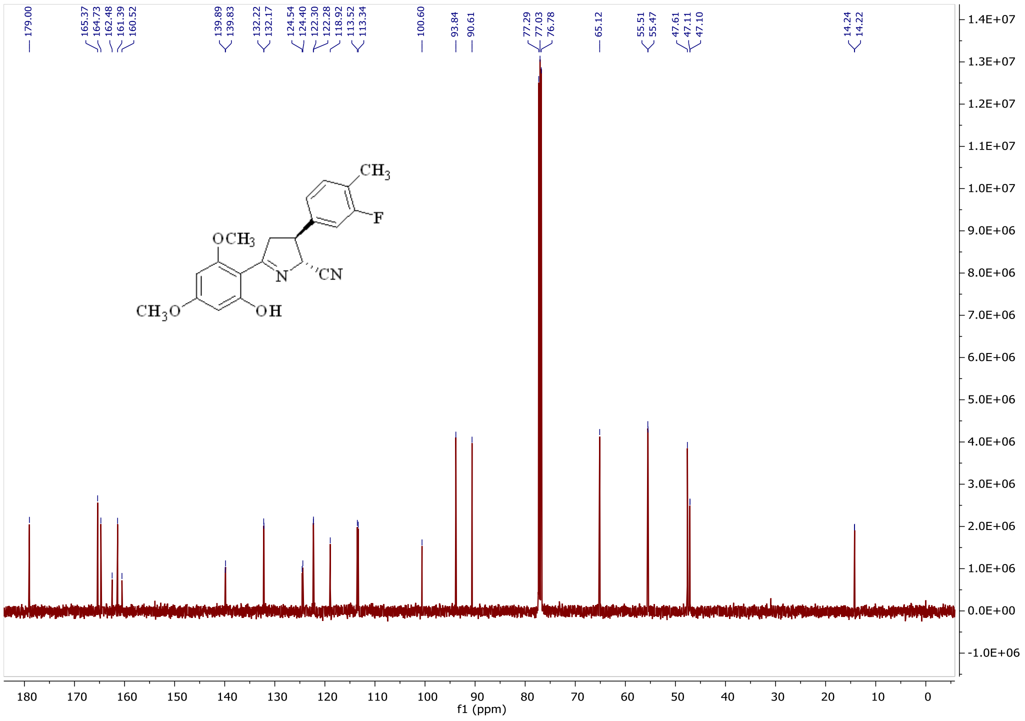

Figure S142: ^{13}C NMR spectrum of compound *trans*-**4m** ($CDCl_3$).

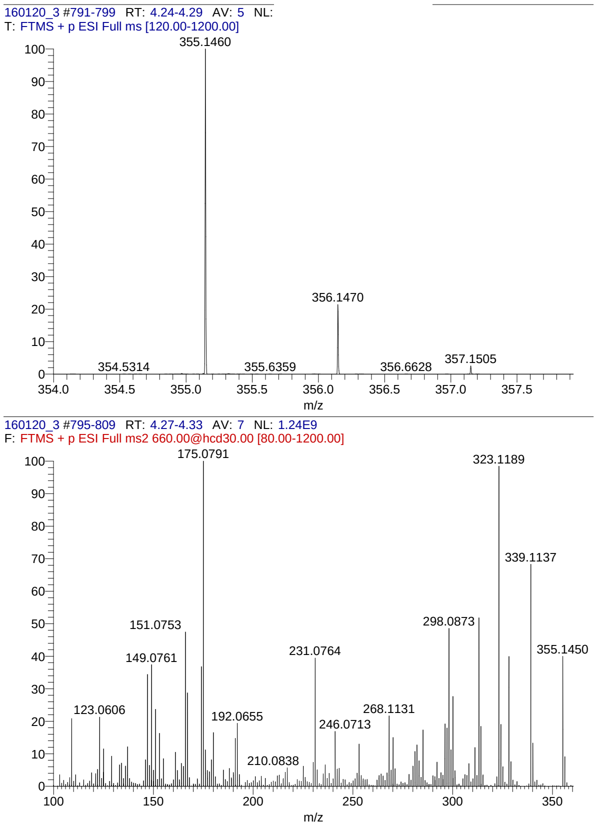

Figure S143: HRMS ESI and HRMS ESI-MS/MS spectrum of compound *trans*-**4m**.

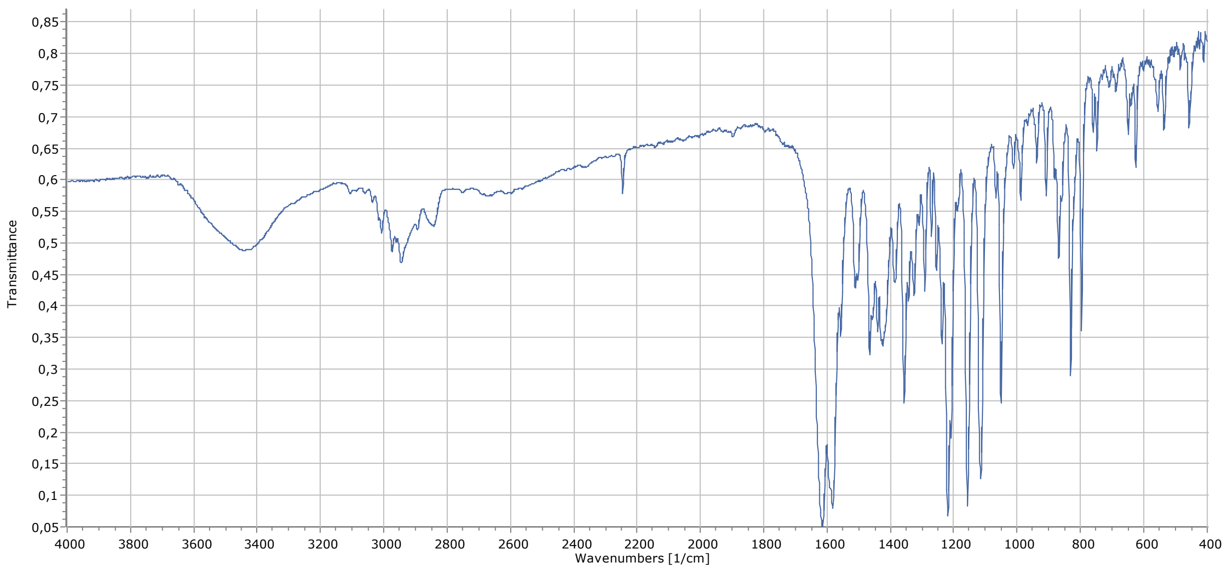

Figure S144: FT-IR spectrum of compound *cis*-**4m** (KBr).

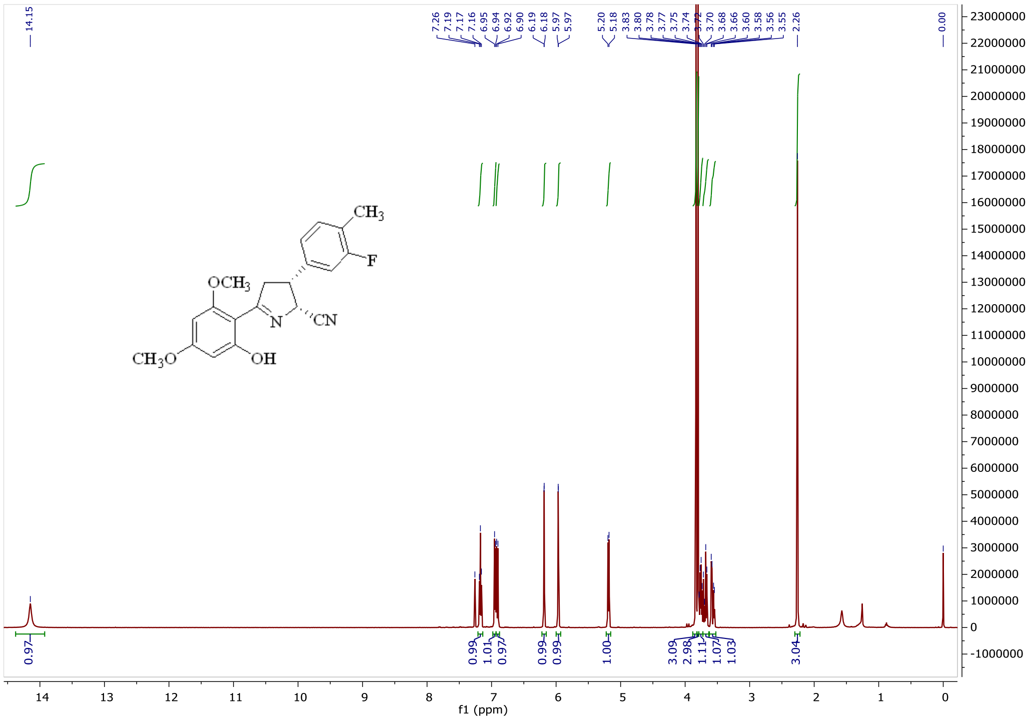

Figure S145: ^{1}H-NMR spectrum of compound *cis*-**4m** ($CDCl_3$).

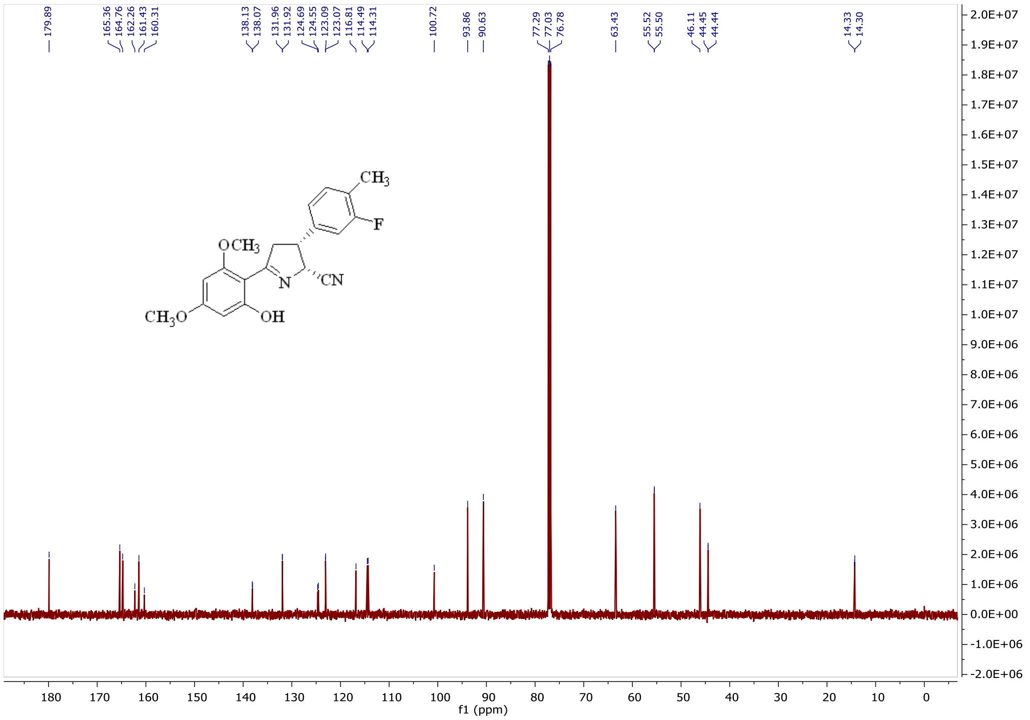

Figure S146: ^{13}C NMR spectrum of compound *cis*-**4m** ($CDCl_3$).

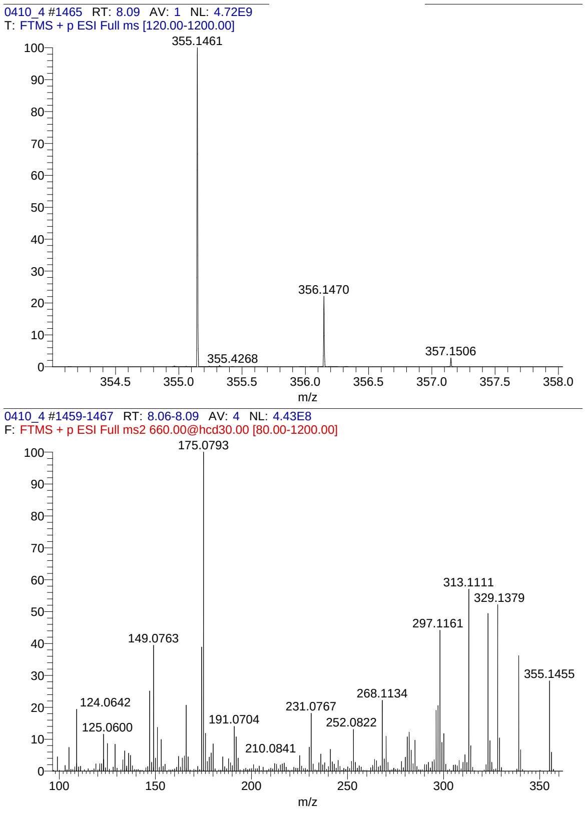

Figure S147: HRMS ESI and HRMS ESI-MS/MS spectrum of compound *cis*-**4m**.

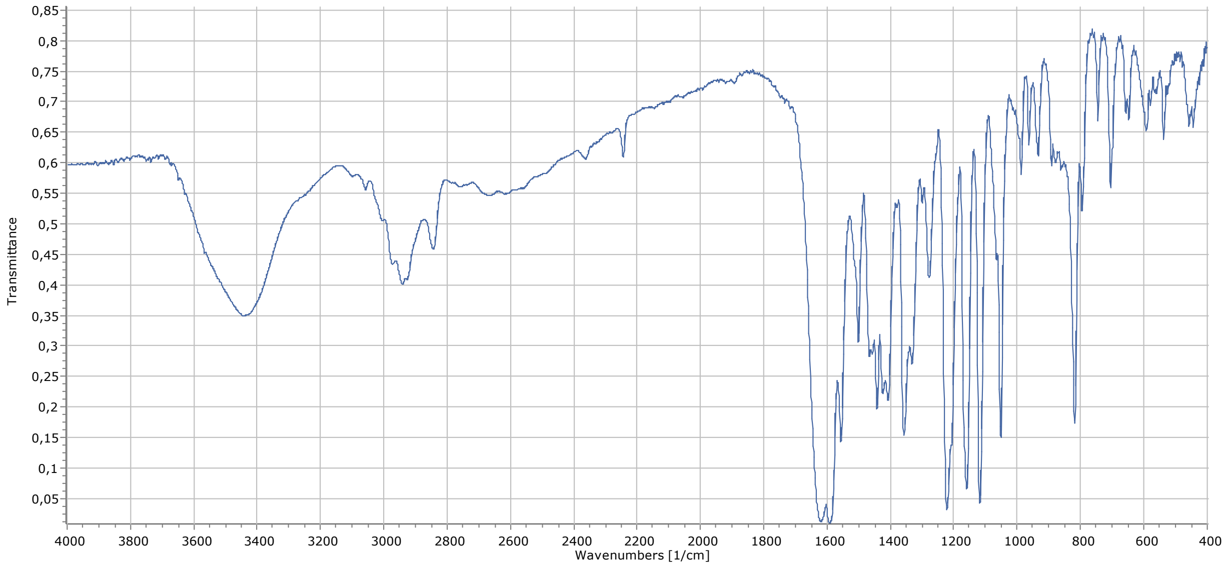

Figure S148: FT-IR spectrum of compound ***trans*-4n** (KBr).

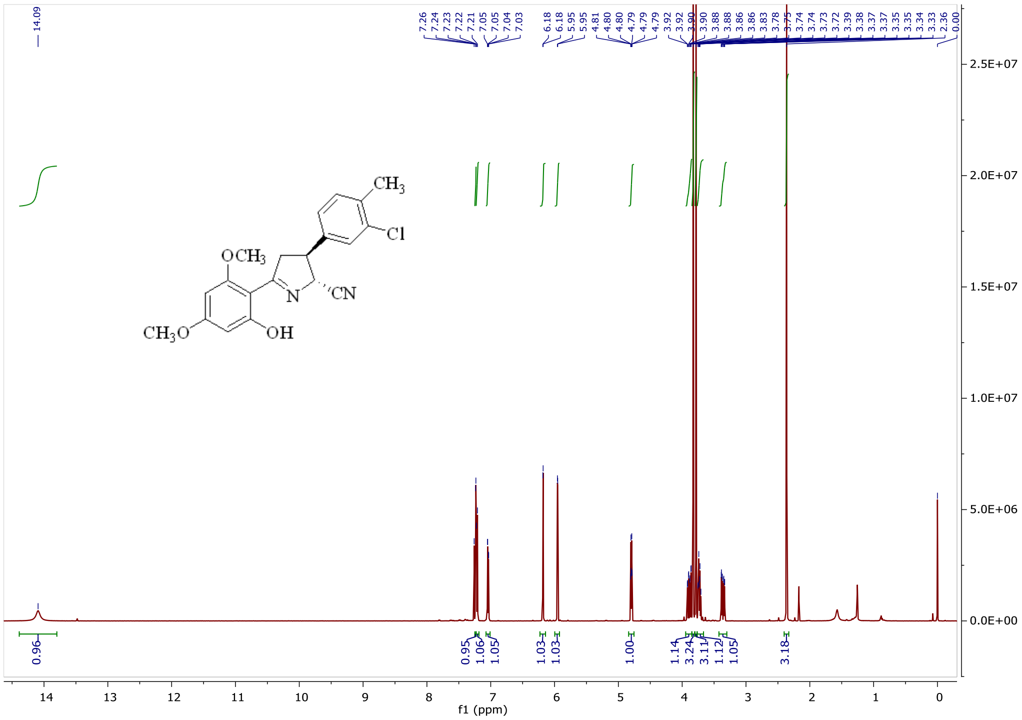

Figure S149: ^{1}H-NMR spectrum of compound *trans*-**4n** ($CDCl_3$).

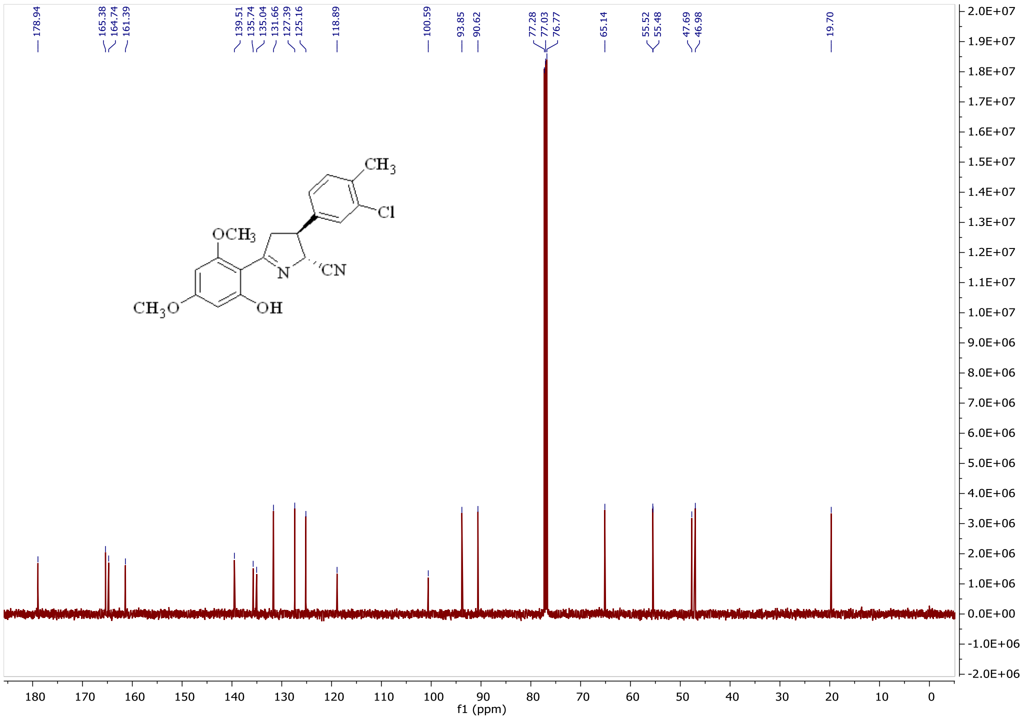

Figure S150: ^{13}C NMR spectrum of compound *trans*-**4n** ($CDCl_3$).

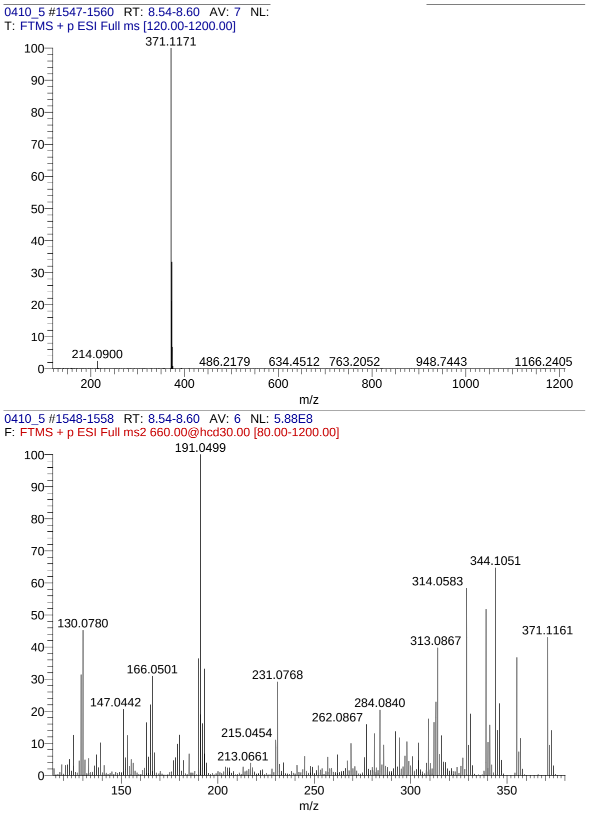

Figure S151: HRMS ESI and HRMS ESI-MS/MS spectrum of compound *trans*-**4n**.

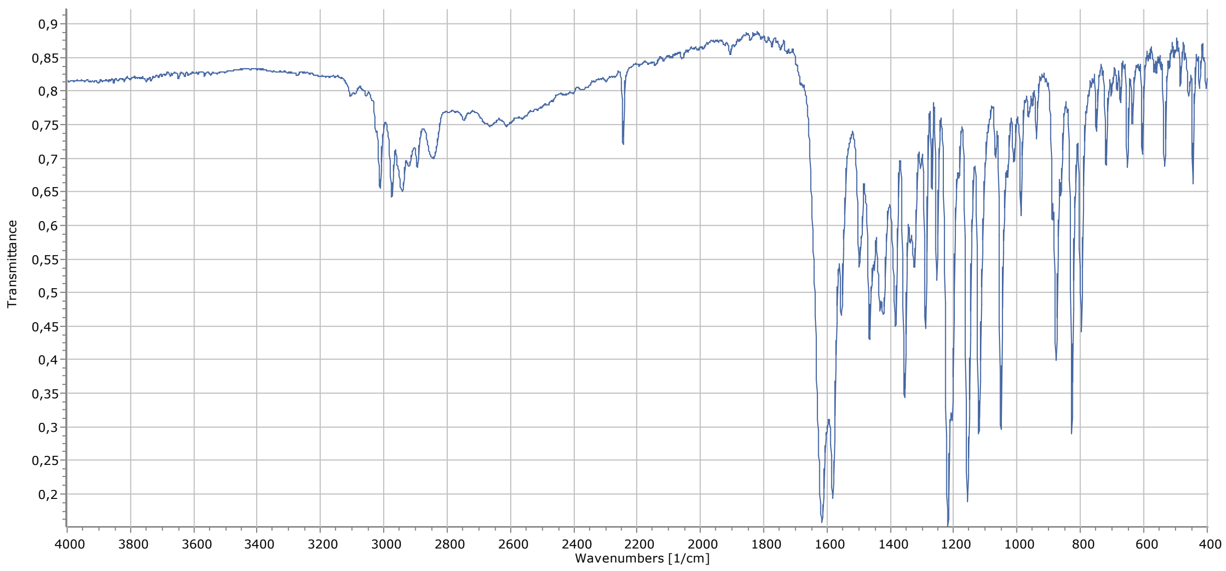

Figure S152: FT-IR spectrum of compound *cis*-**4n** (KBr).

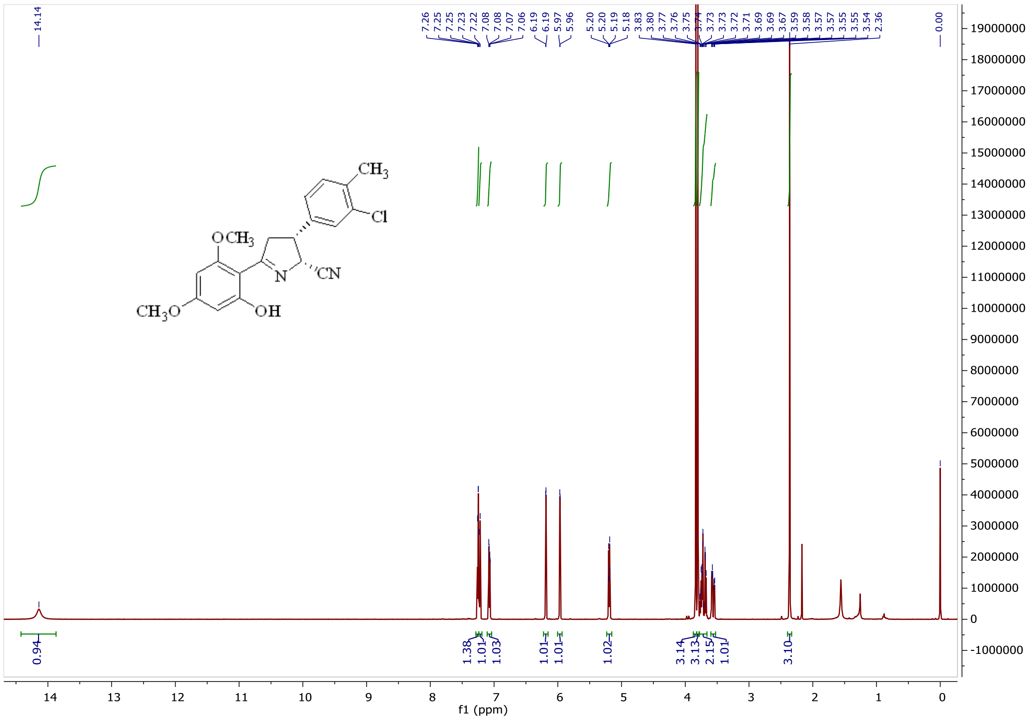

Figure S153: ¹H-NMR spectrum of compound *cis*-**4n** ($CDCl_3$).

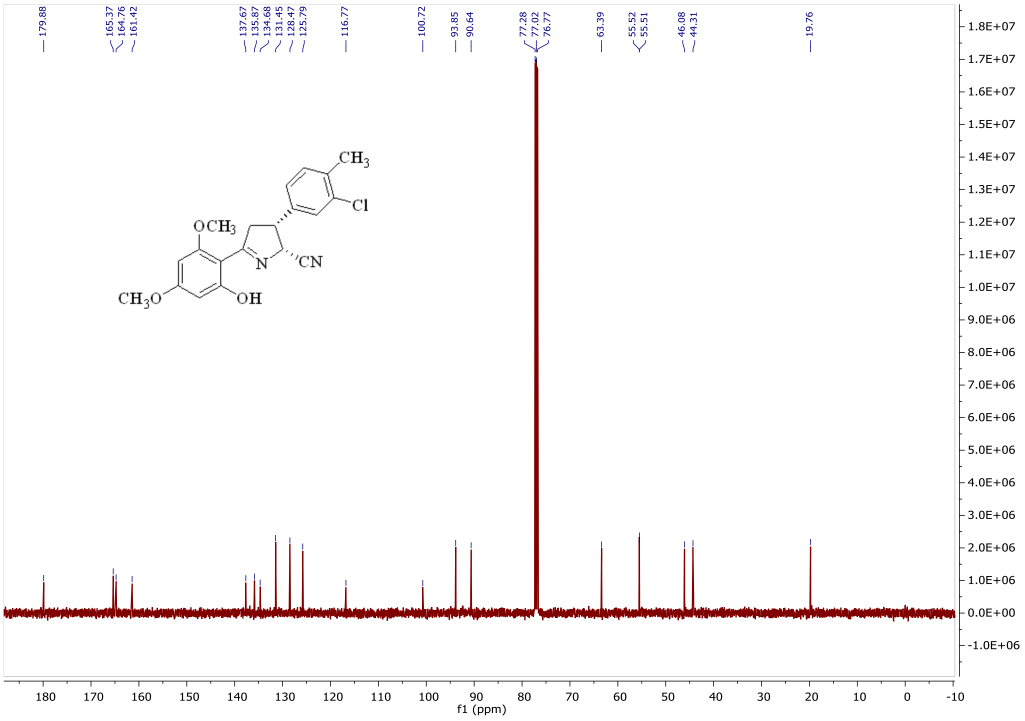

Figure S154: ^{13}C NMR spectrum of compound *cis*-**4n** ($CDCl_3$).

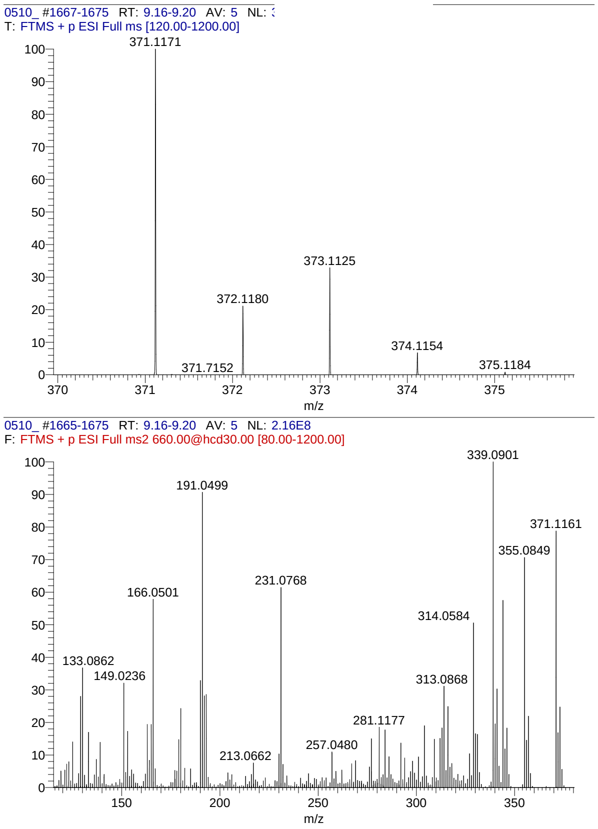

Figure S155: HRMS ESI and HRMS ESI-MS/MS spectrum of compound *cis*-**4n**.

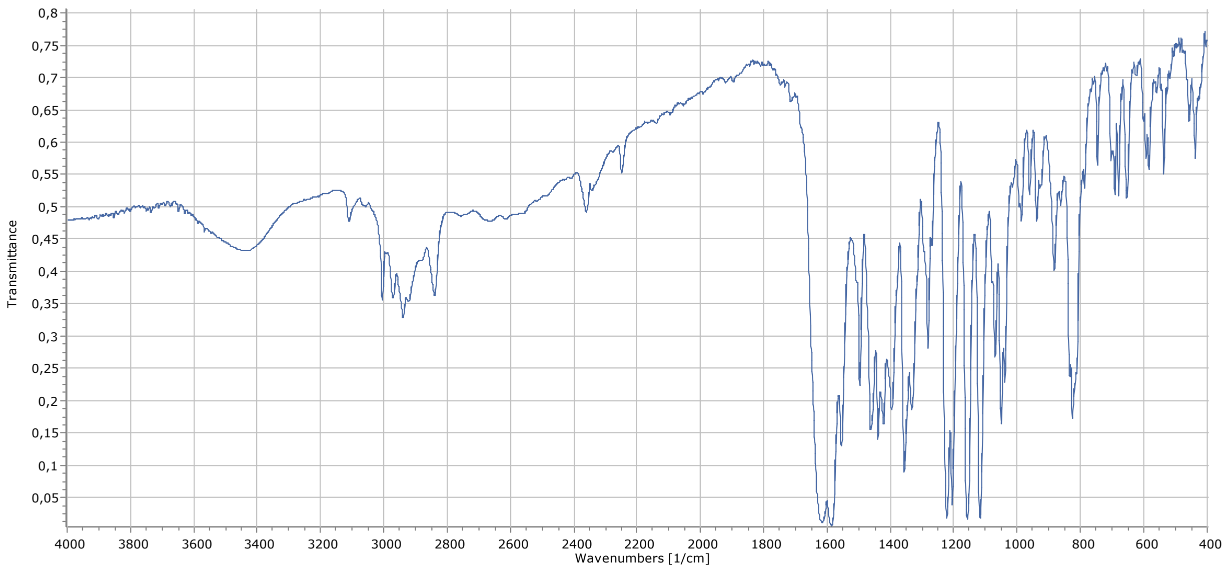

Figure S156: FT-IR spectrum of compound ***trans*-4o** (KBr).

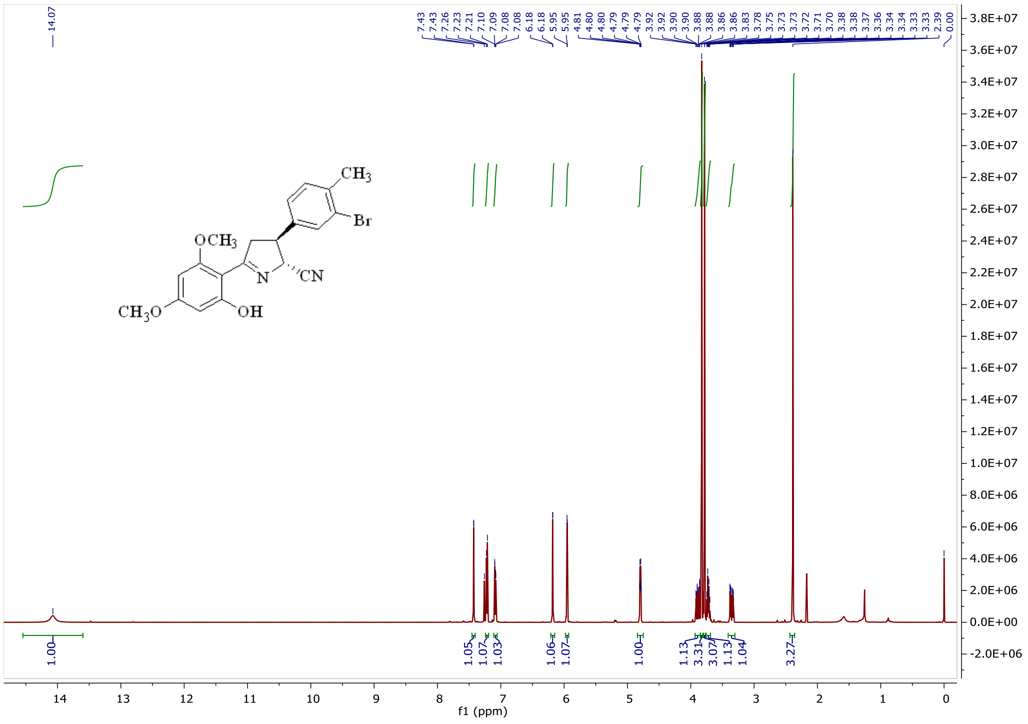

Figure S157: ^{1}H-NMR spectrum of compound *trans*-**4o** ($CDCl_3$).

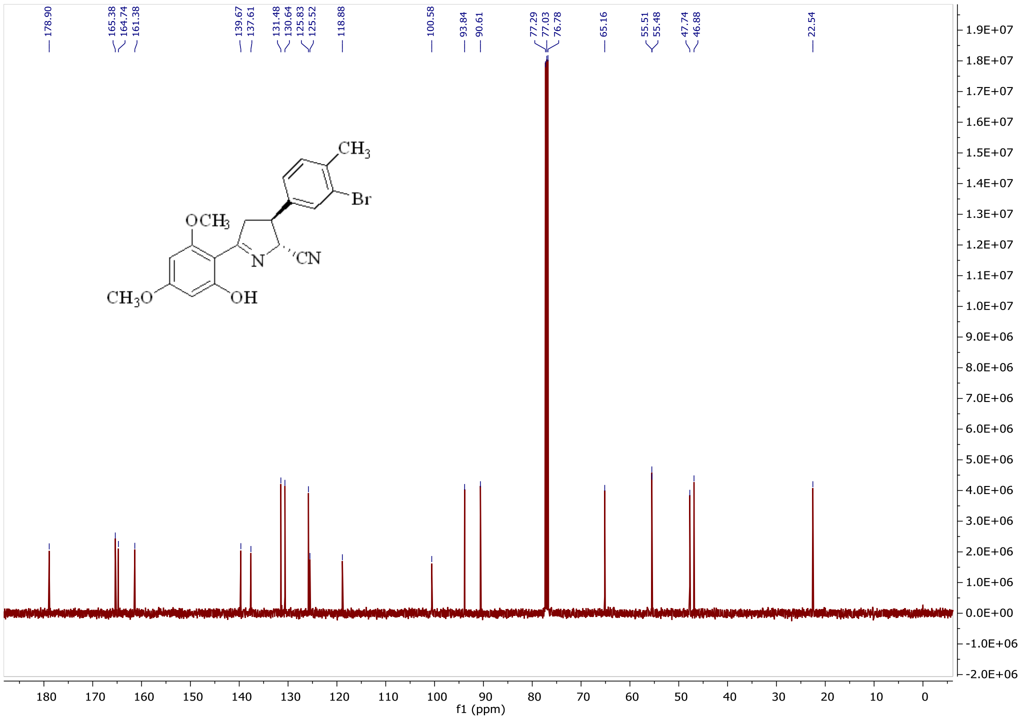

Figure S158: ^{13}C NMR spectrum of compound *trans*-**4o** ($CDCl_3$).

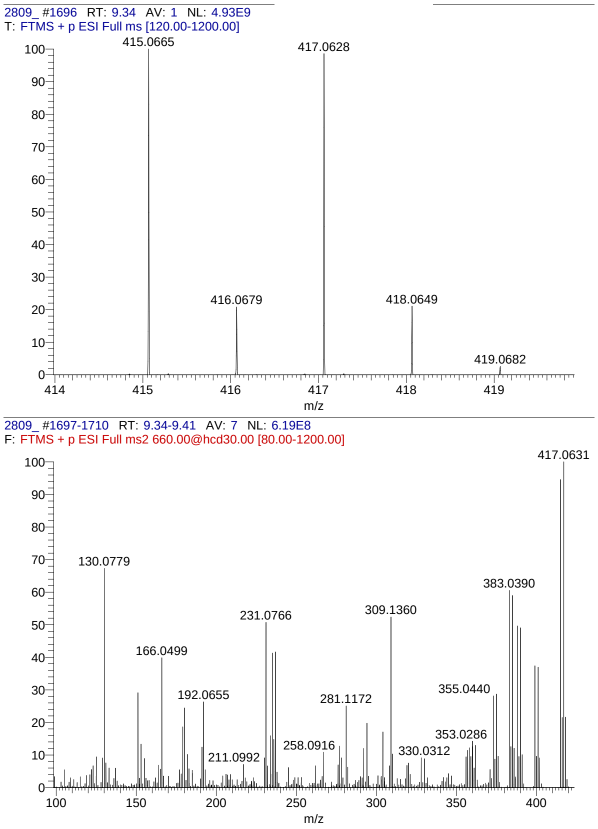

Figure S159: HRMS ESI and HRMS ESI-MS/MS spectrum of compound *trans*-**4o**.

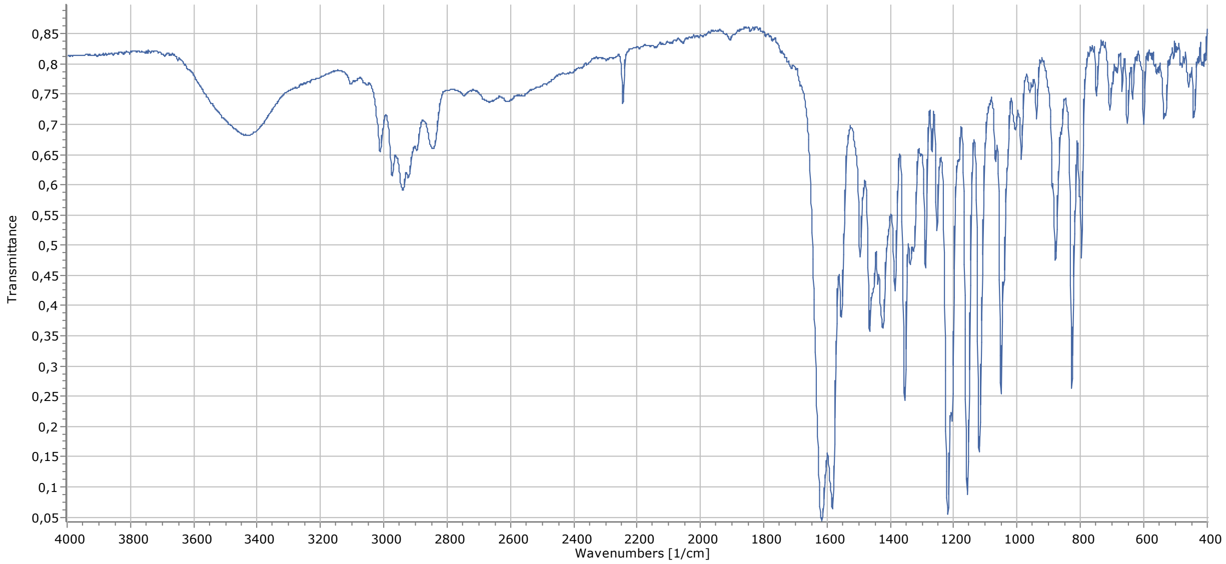

Figure S160: FT-IR spectrum of compound *cis*-**4o** (KBr).

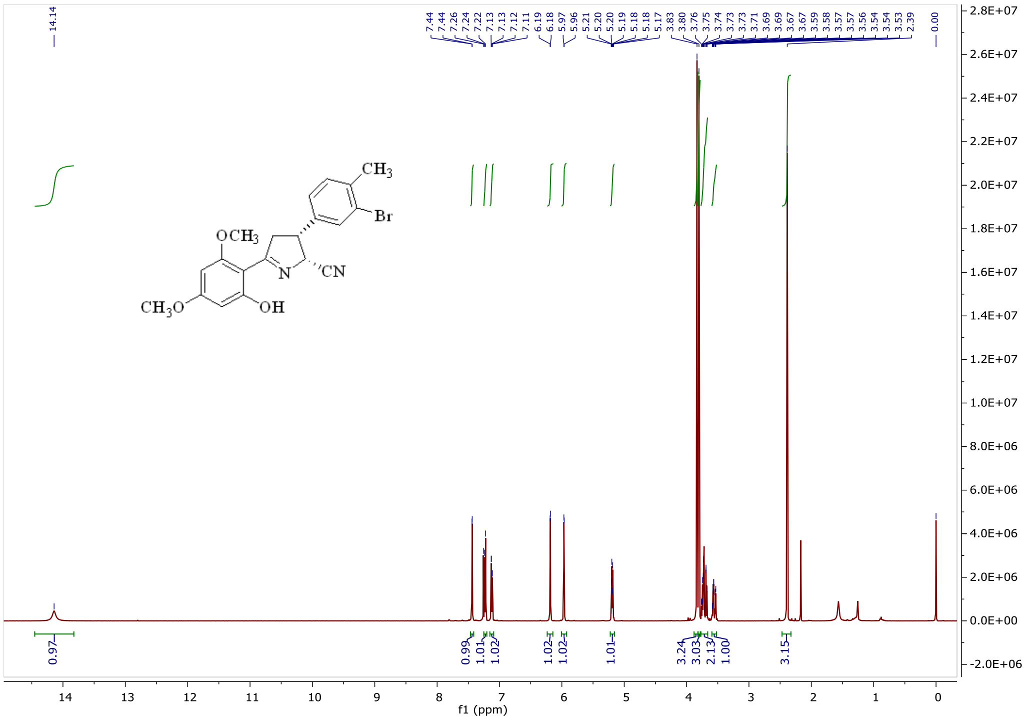

Figure S161: ^{1}H-NMR spectrum of compound *cis*-**4o** ($CDCl_3$).

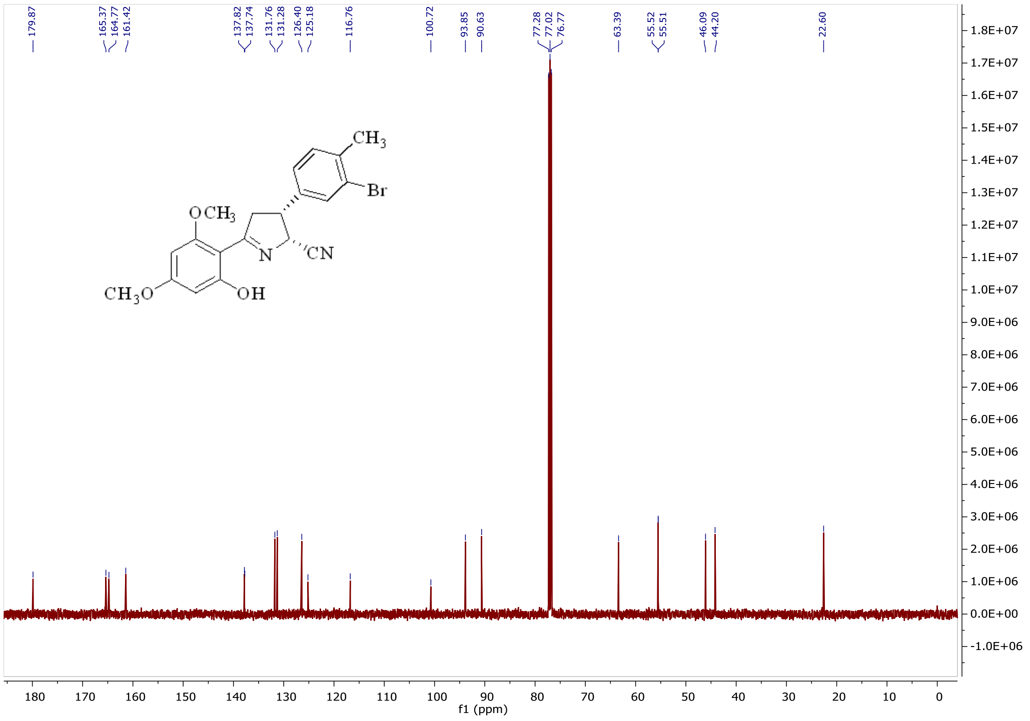

Figure S162: ^{13}C NMR spectrum of compound *cis*-**4o** ($CDCl_3$).

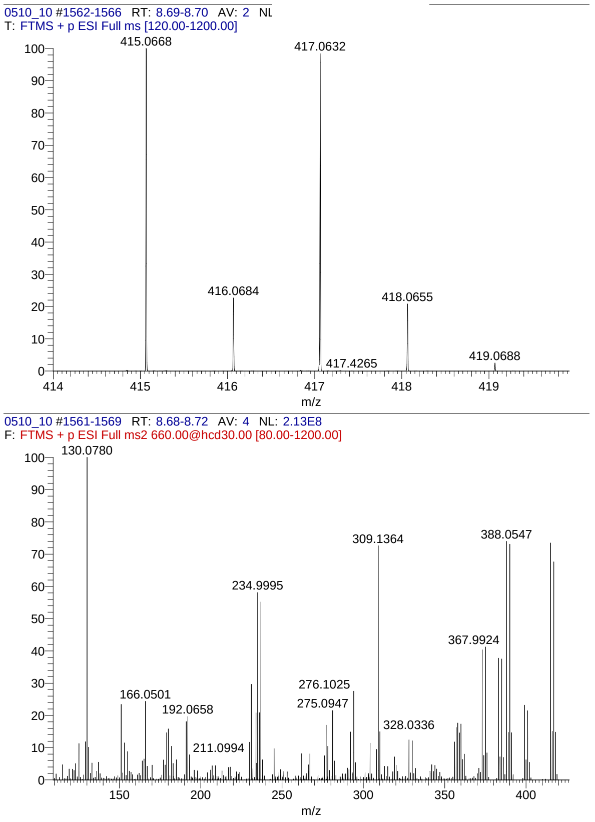

Figure S163: HRMS ESI and HRMS ESI-MS/MS spectrum of compound *cis*-**4o**.

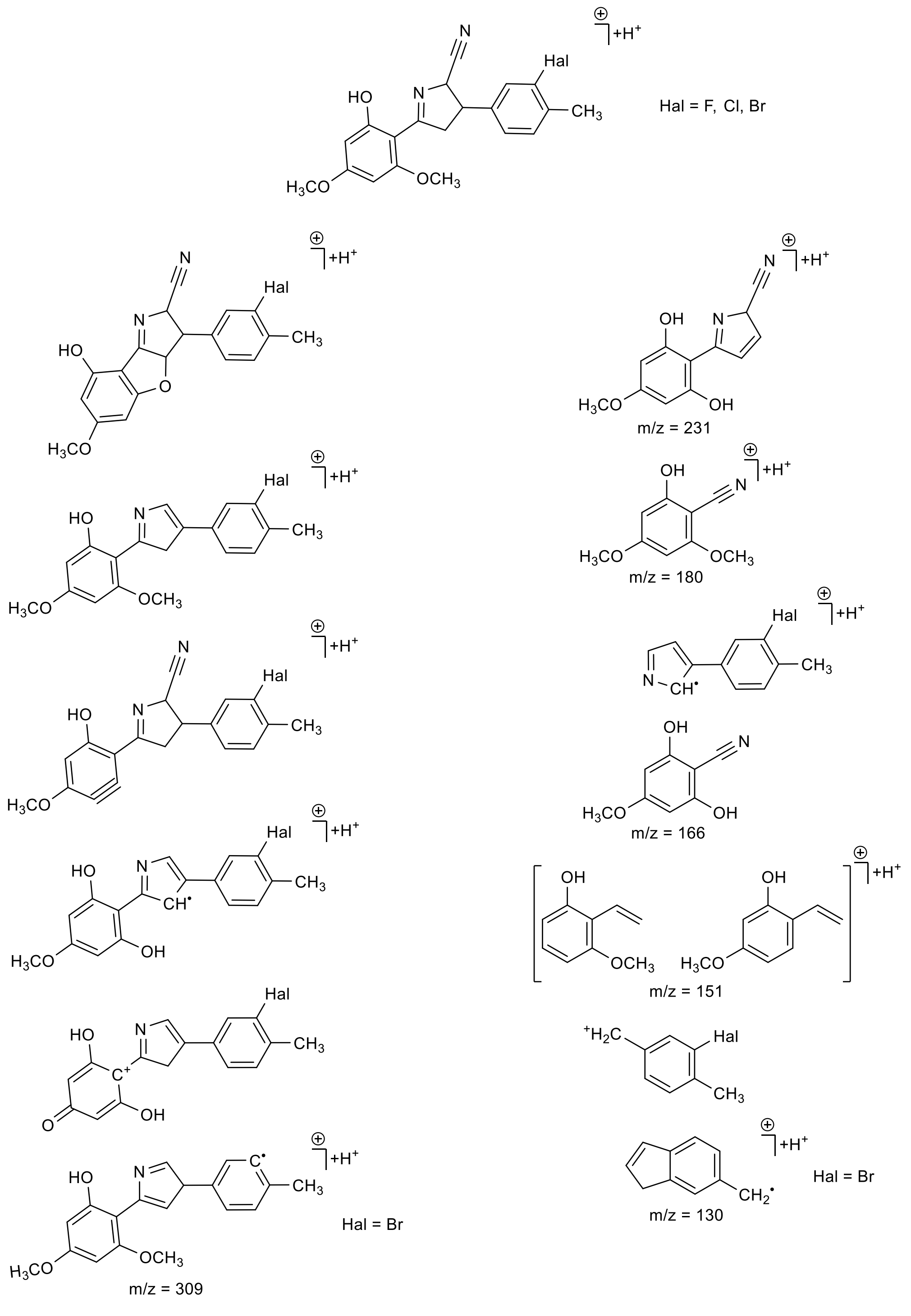

Figure S164: Proposed structures of the most abundant fragment ions observed in a MS/MS spectra of compounds *trans*-**4m–4o** and *cis*-**4m–4o**.

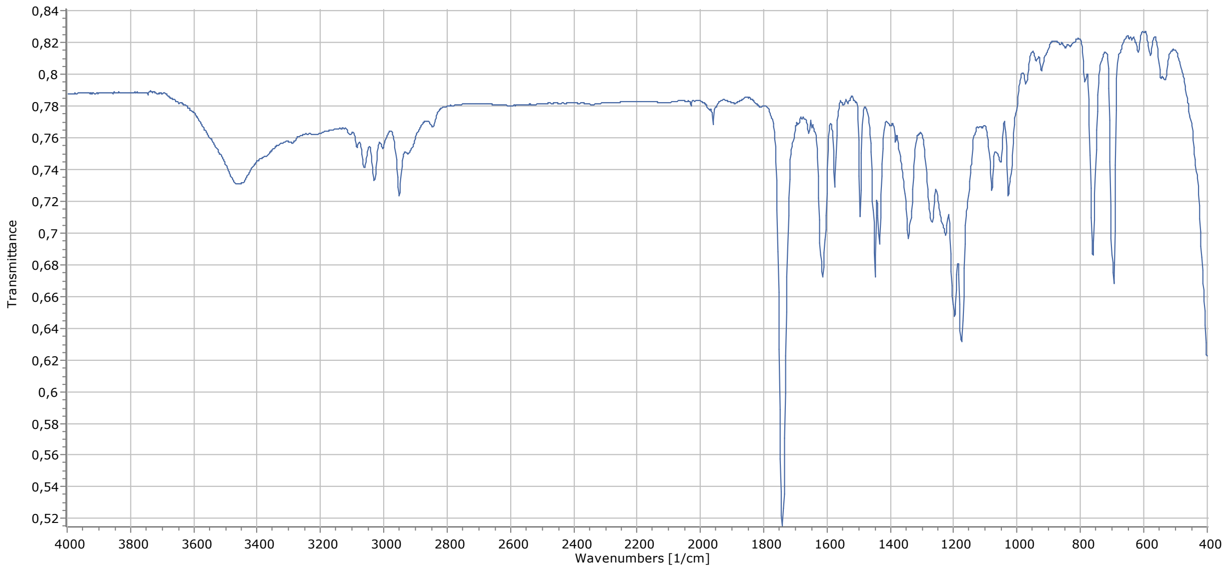

Figure S165: FT-IR spectrum of compound *trans*-**5a** (capillary layer).

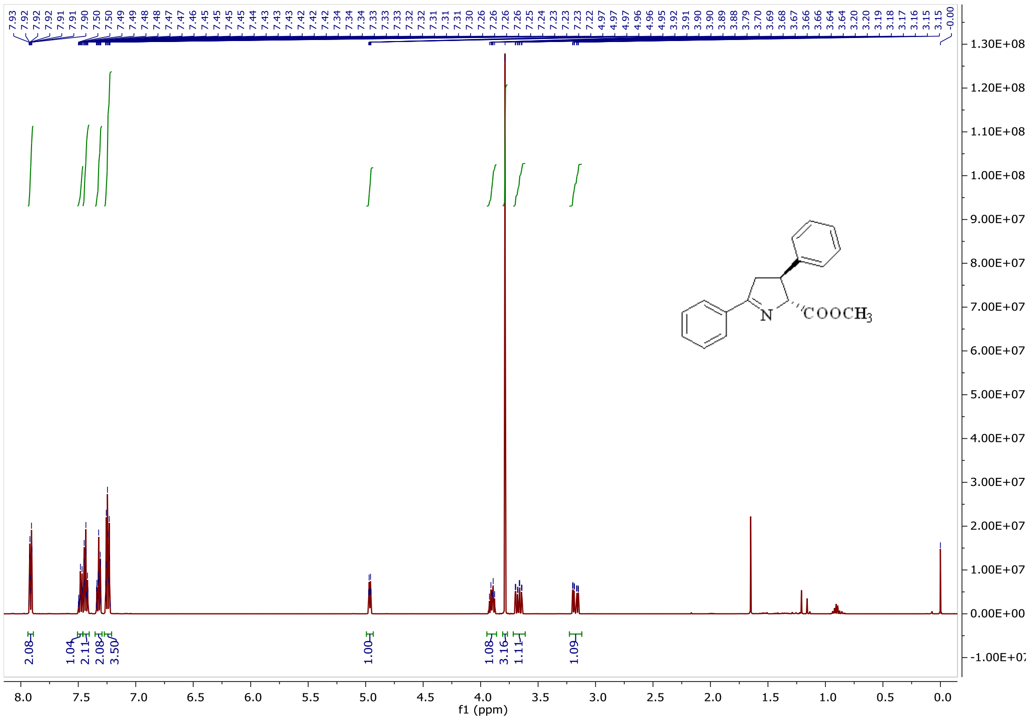

Figure S166: ^{1}H-NMR spectrum of compound *trans*-**5a** ($CDCl_3$).

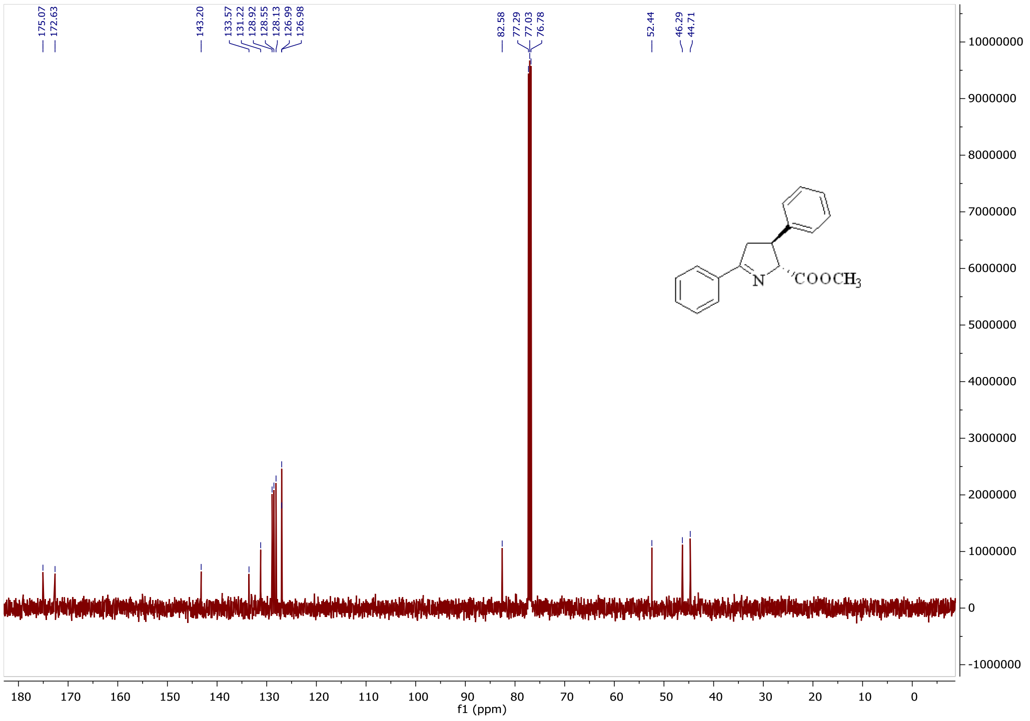

Figure S167: ^{13}C NMR spectrum of compound *trans*-**5a** ($CDCl_3$).

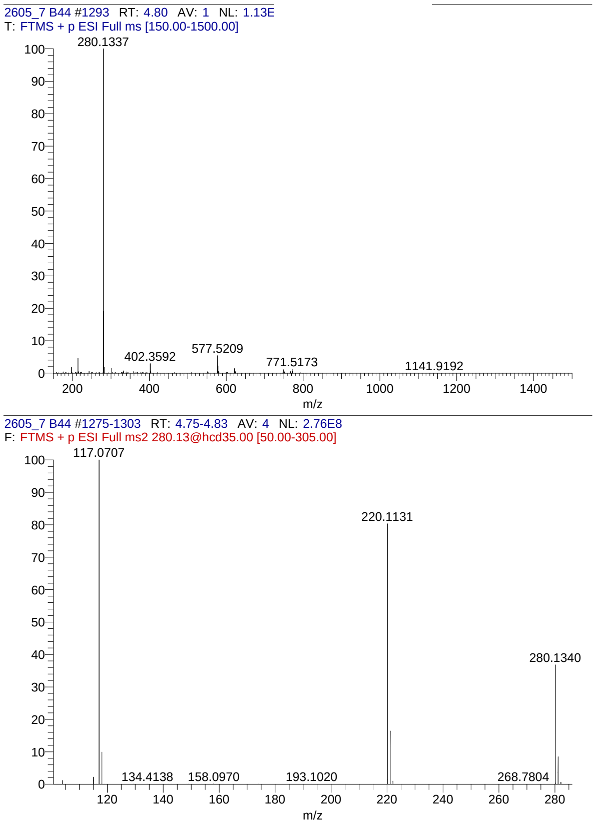

Figure S168: HRMS ESI and HRMS ESI-MS/MS spectrum of compound *trans*-**5a**.

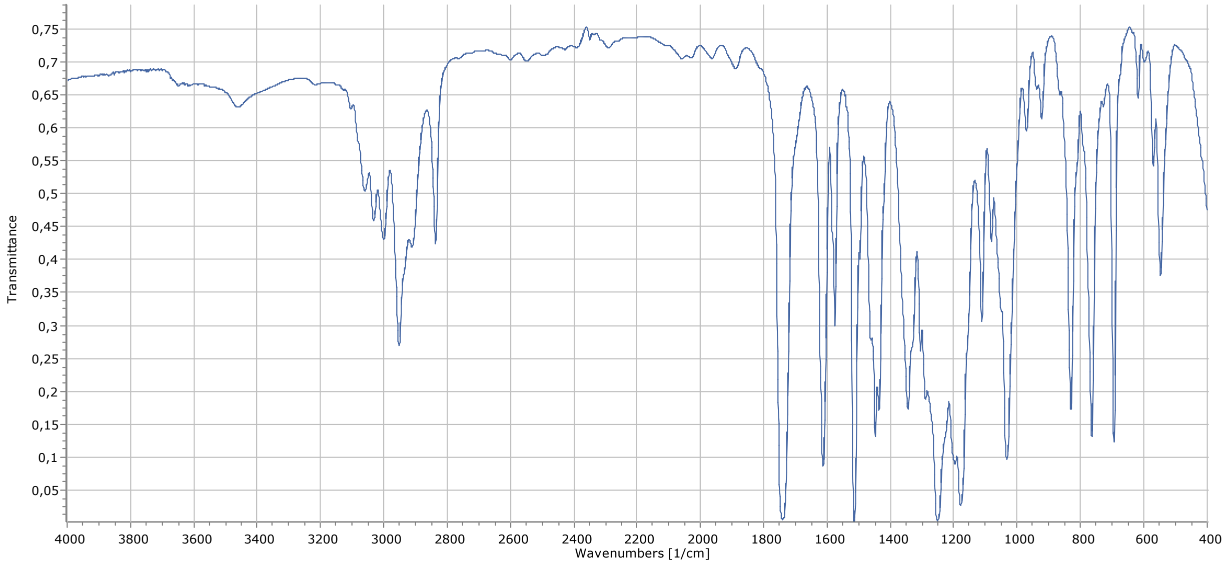

Figure S169: FT-IR spectrum of compound *trans*-**5b** (capillary layer).

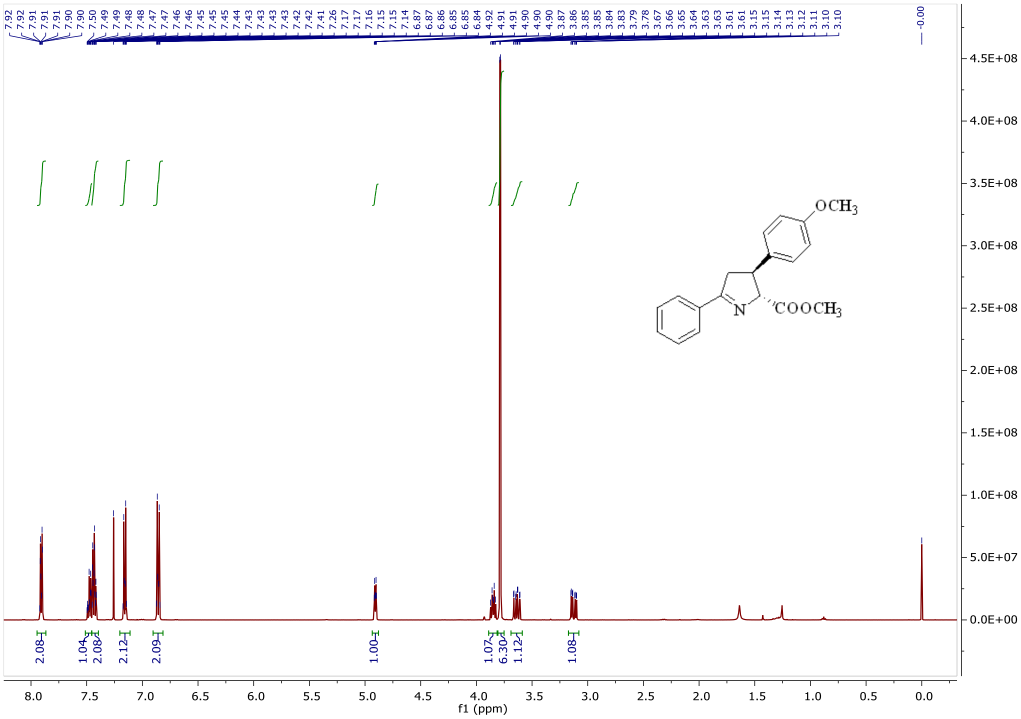

Figure S170: ^{1}H-NMR spectrum of compound *trans*-**5b** ($CDCl_3$).

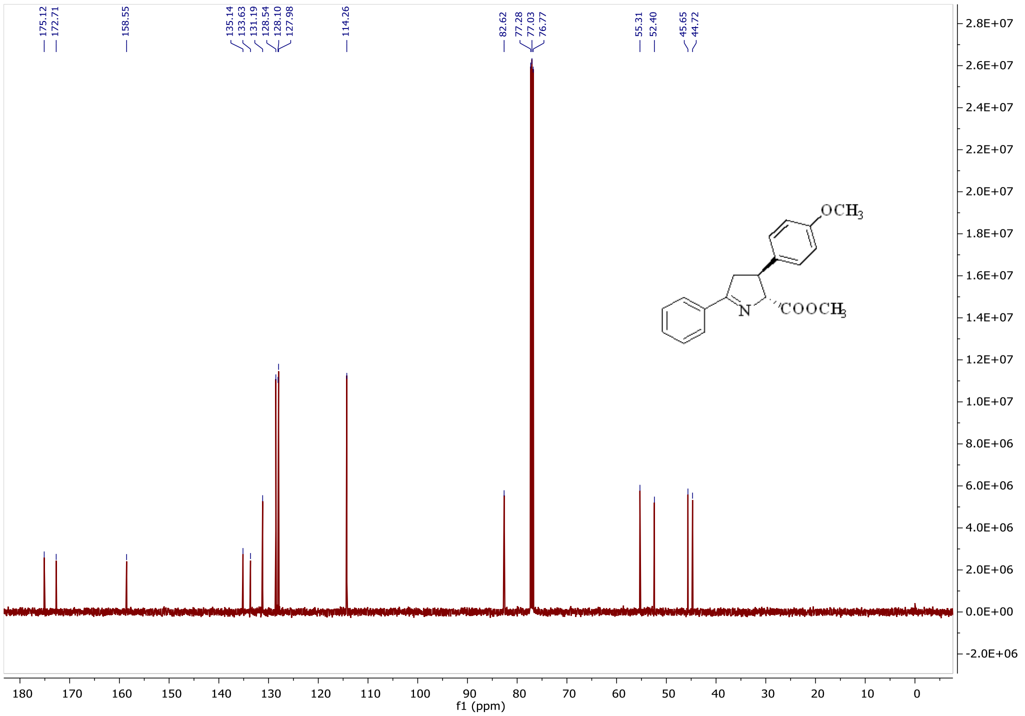

Figure S171: ^{13}C NMR spectrum of compound *trans*-**5b** ($CDCl_3$).

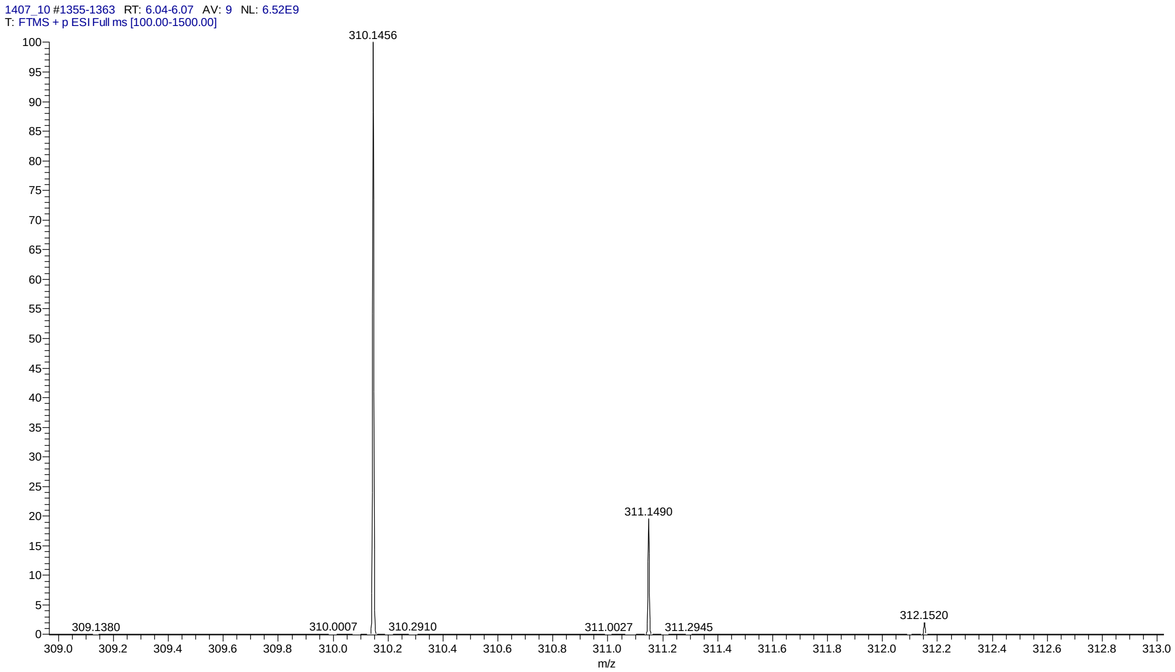

Figure S172: HRMS ESI spectrum of compound *trans*-**5b**.

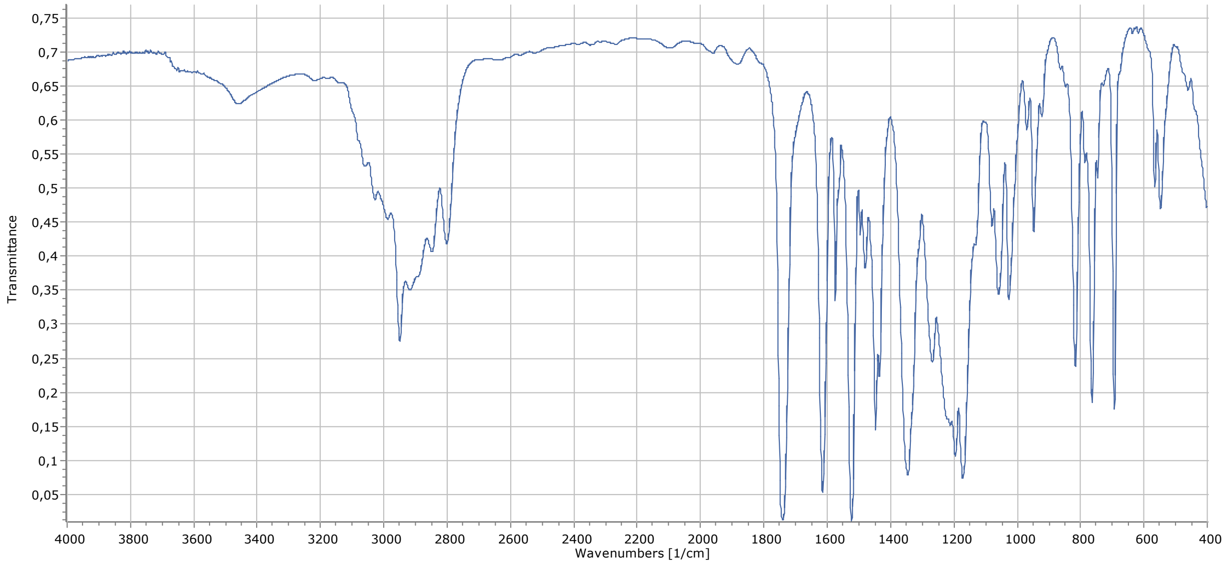

Figure S173: FT-IR spectrum of compound *trans*-**5c** (capillary layer).

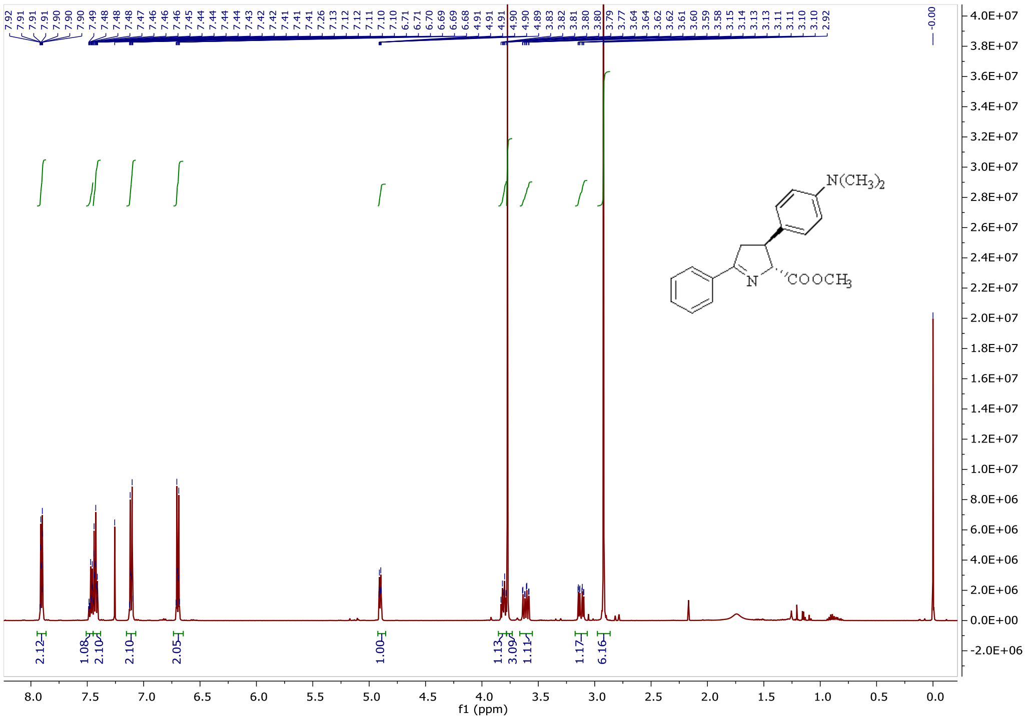

Figure S174: ^{1}H-NMR spectrum of compound *trans*-**5c** ($CDCl_3$).

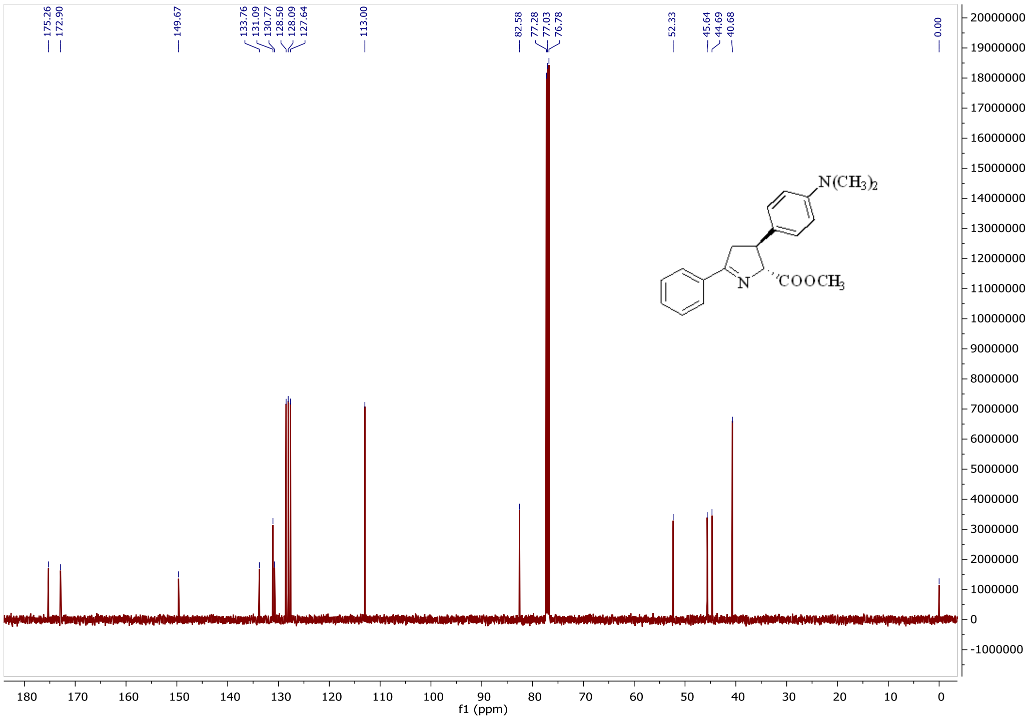

Figure S175: ^{13}C NMR spectrum of compound *trans*-**5c** ($CDCl_3$).

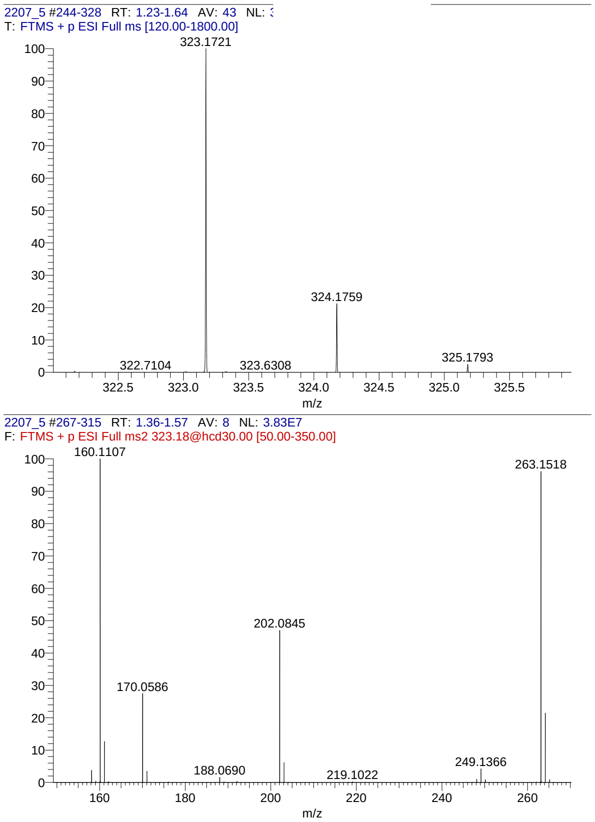

Figure S176: HRMS ESI and HRMS ESI-MS/MS spectrum of compound *trans*-**5c**.

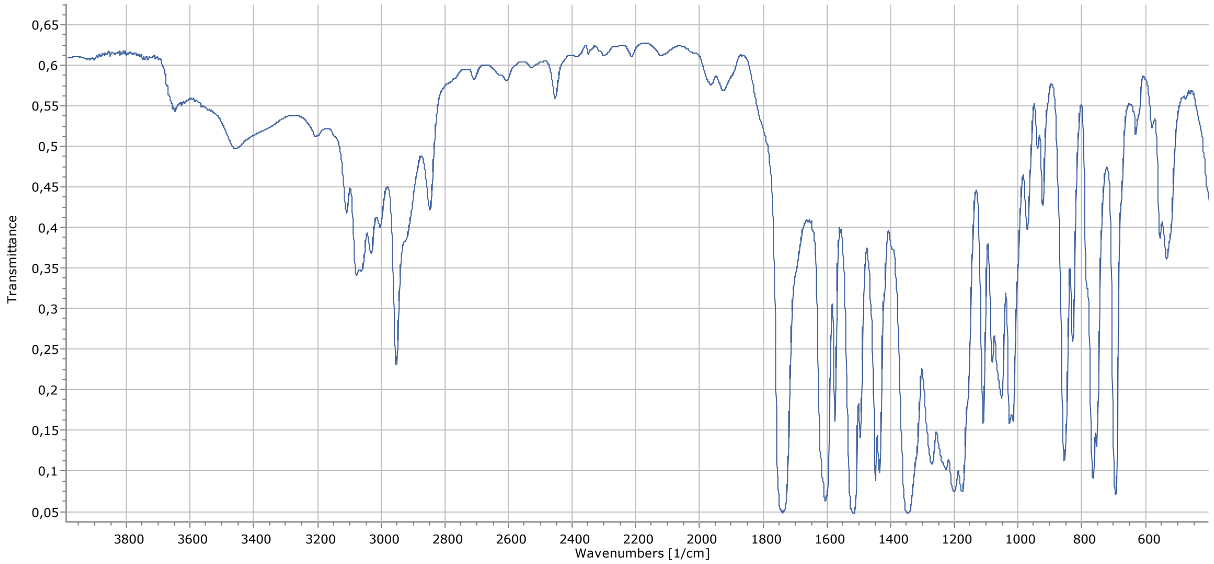

Figure S177: FT-IR spectrum of compound *trans*-**5d** (capillary layer).

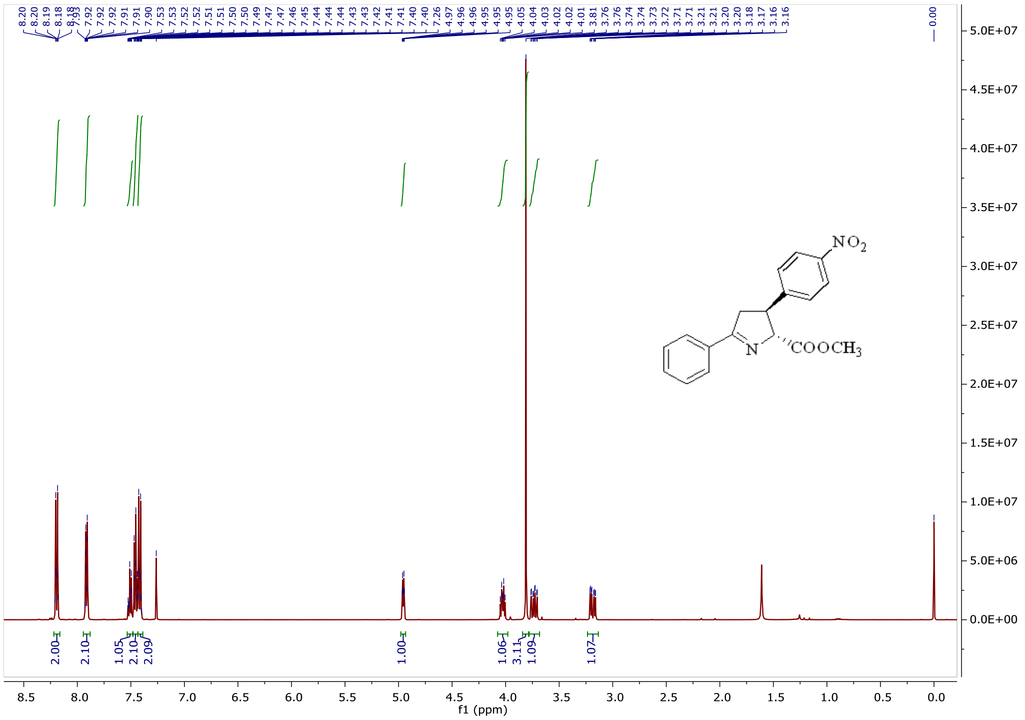

Figure S178: ^{1}H-NMR spectrum of compound *trans*-**5d** ($CDCl_3$).

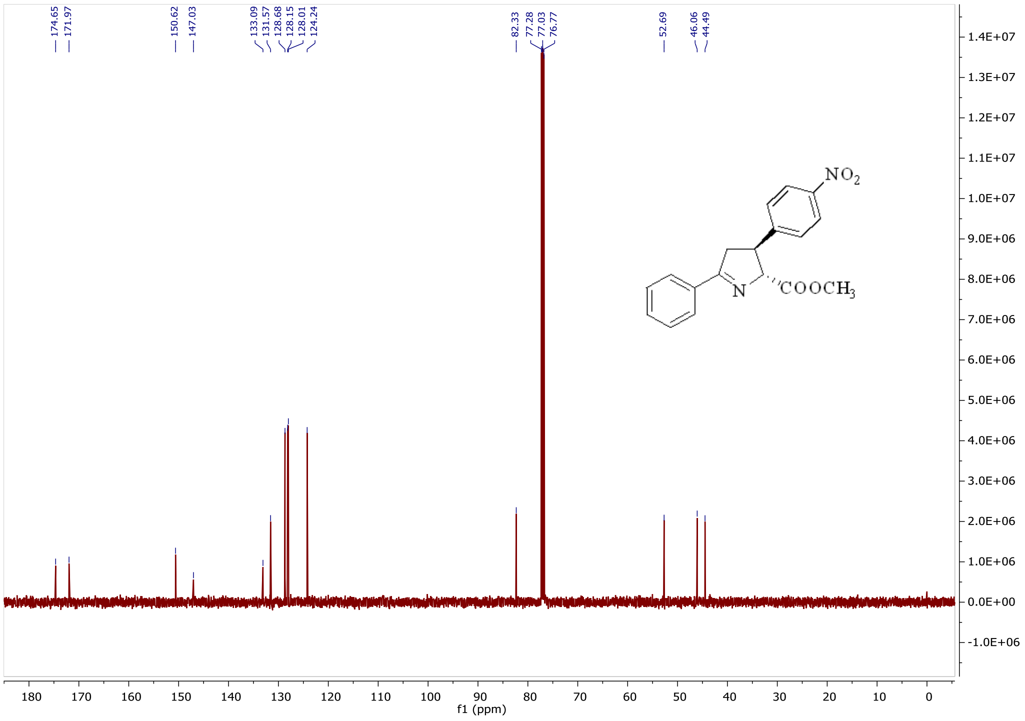

Figure S179: ^{13}C NMR spectrum of compound *trans*-**5d** ($CDCl_3$).

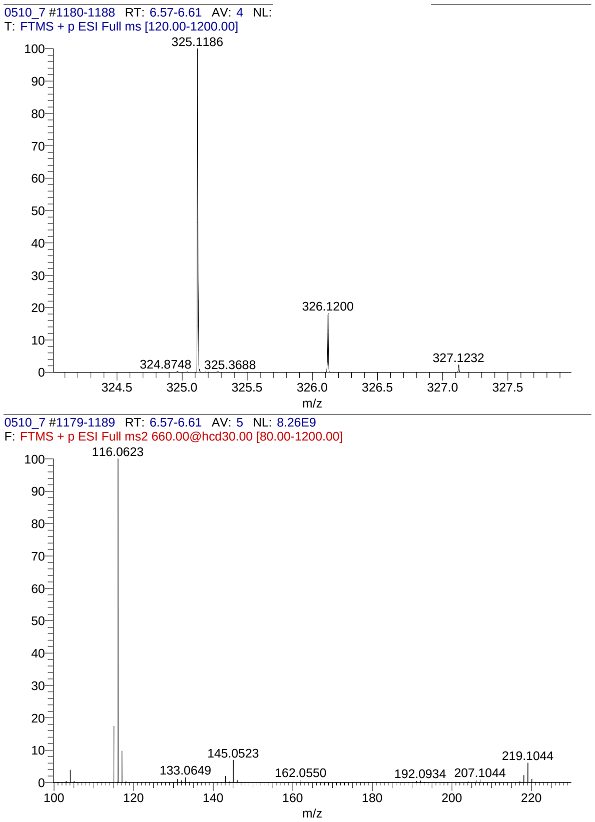

Figure S180: HRMS ESI and HRMS ESI-MS/MS spectrum of compound *trans*-**5d**.

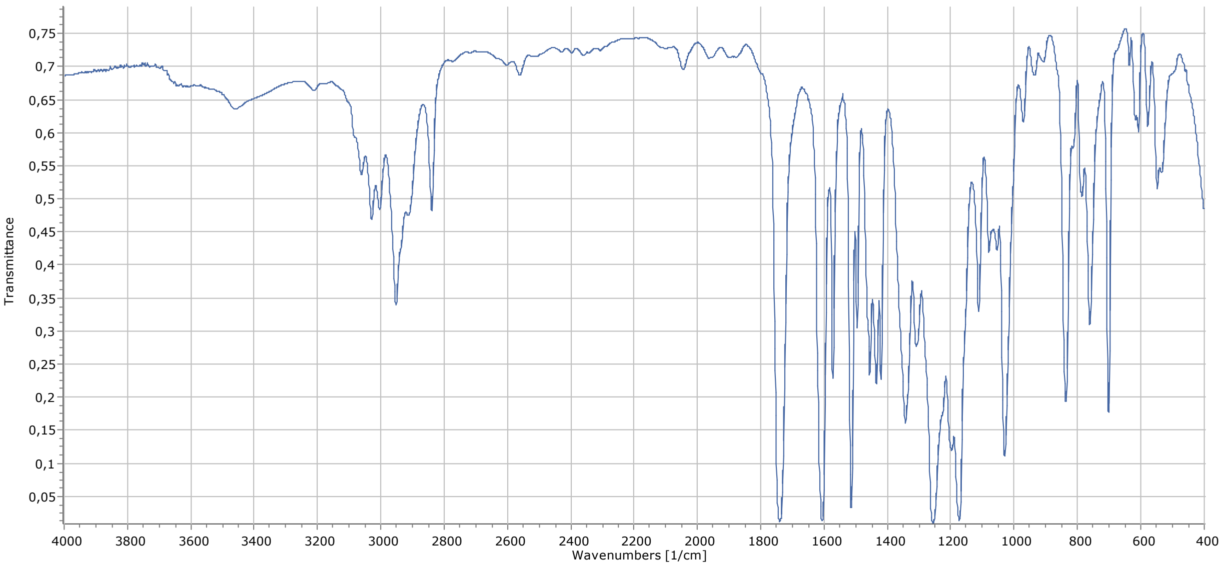

Figure S181: FT-IR spectrum of compound *trans*-**5e** (capillary layer).

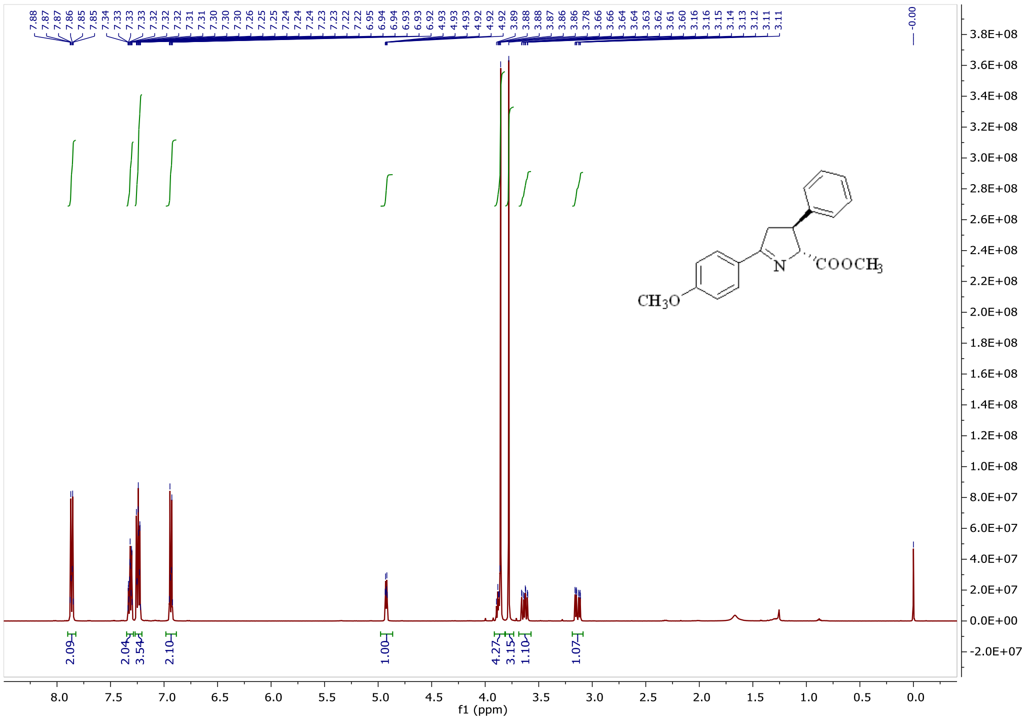

Figure S182: ^{1}H-NMR spectrum of compound *trans*-**5e** ($CDCl_3$).

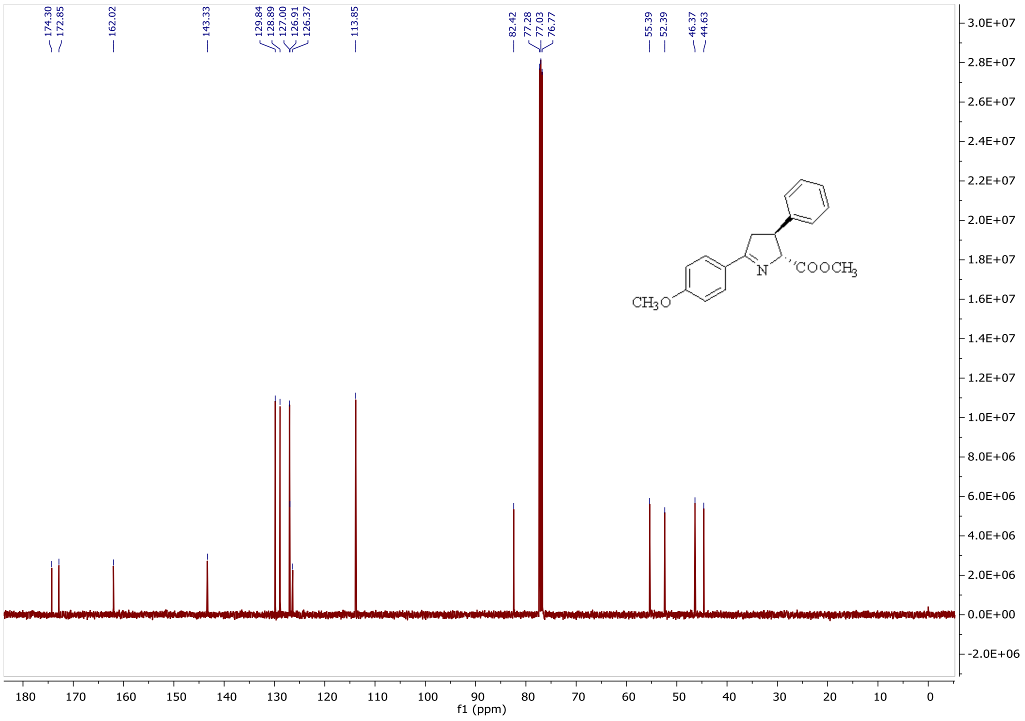

Figure S183: ^{13}C NMR spectrum of compound *trans*-**5e** ($CDCl_3$).

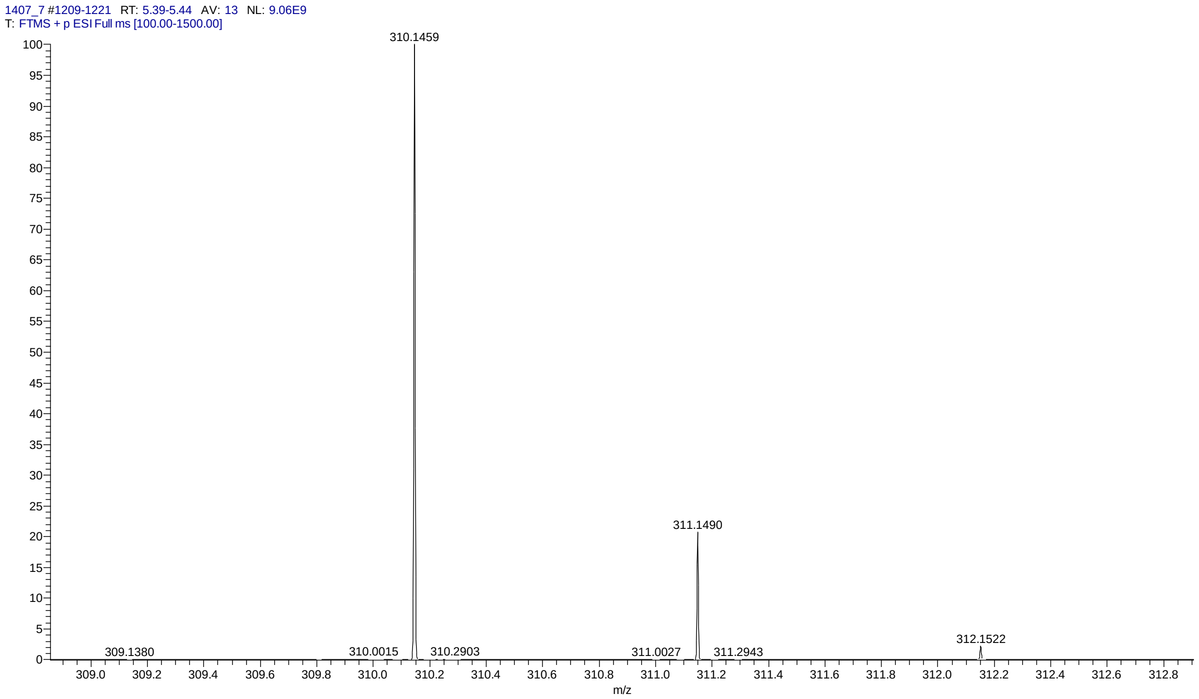

Figure S184: HRMS ESI spectrum of compound *trans*-**5e**.

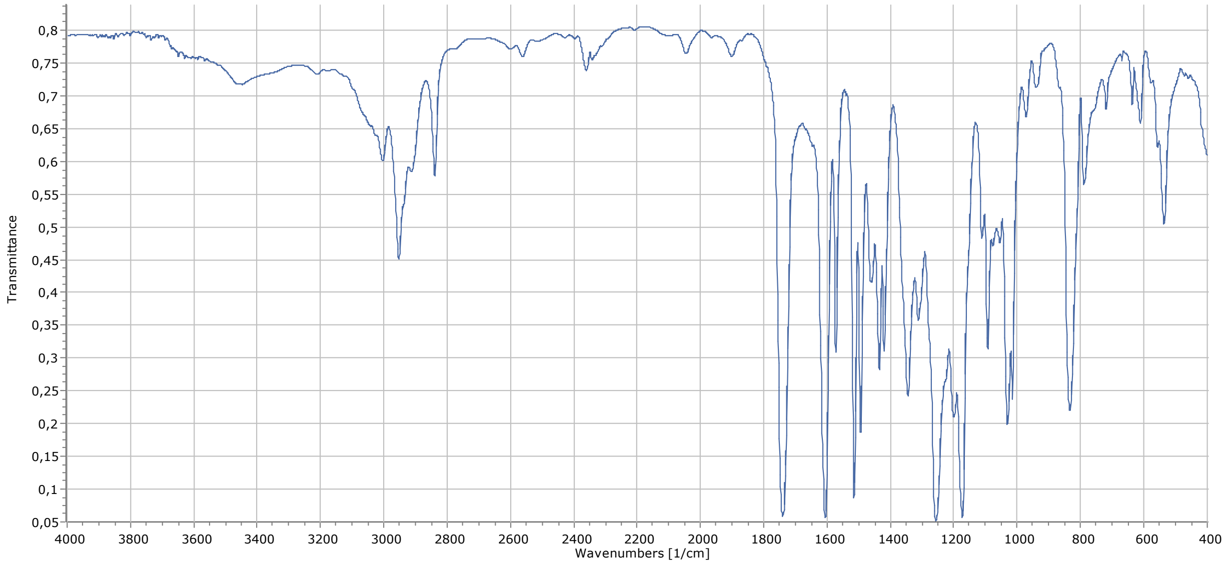

Figure S185: FT-IR spectrum of compound *trans*-**5f** (capillary layer).

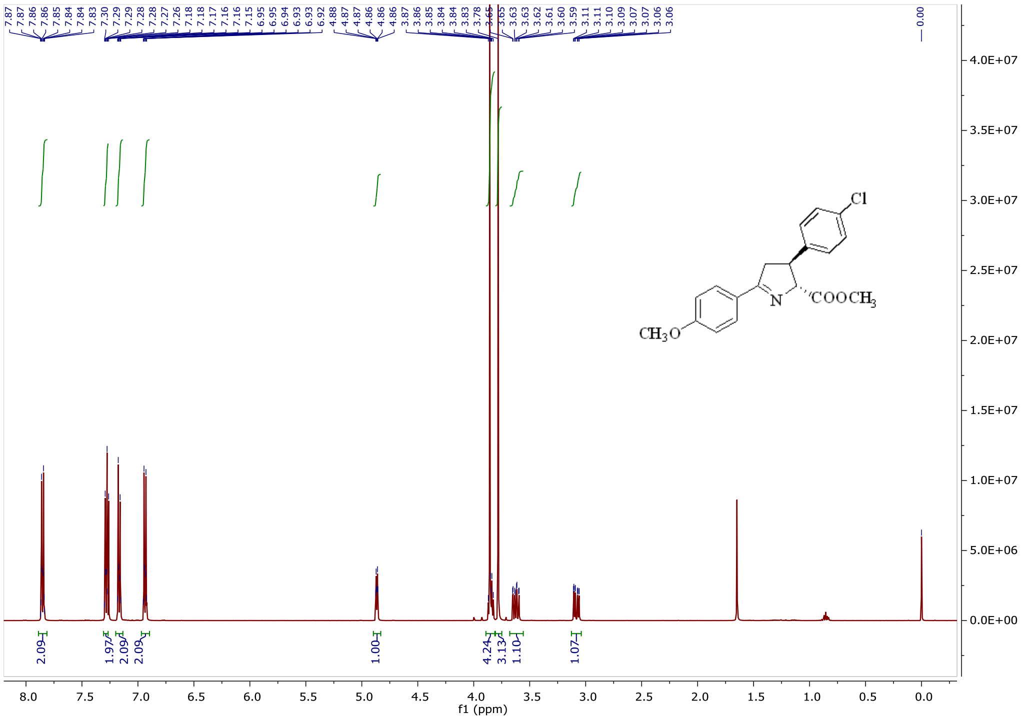

Figure S186: ^{1}H-NMR spectrum of compound *trans*-**5f** ($CDCl_3$).

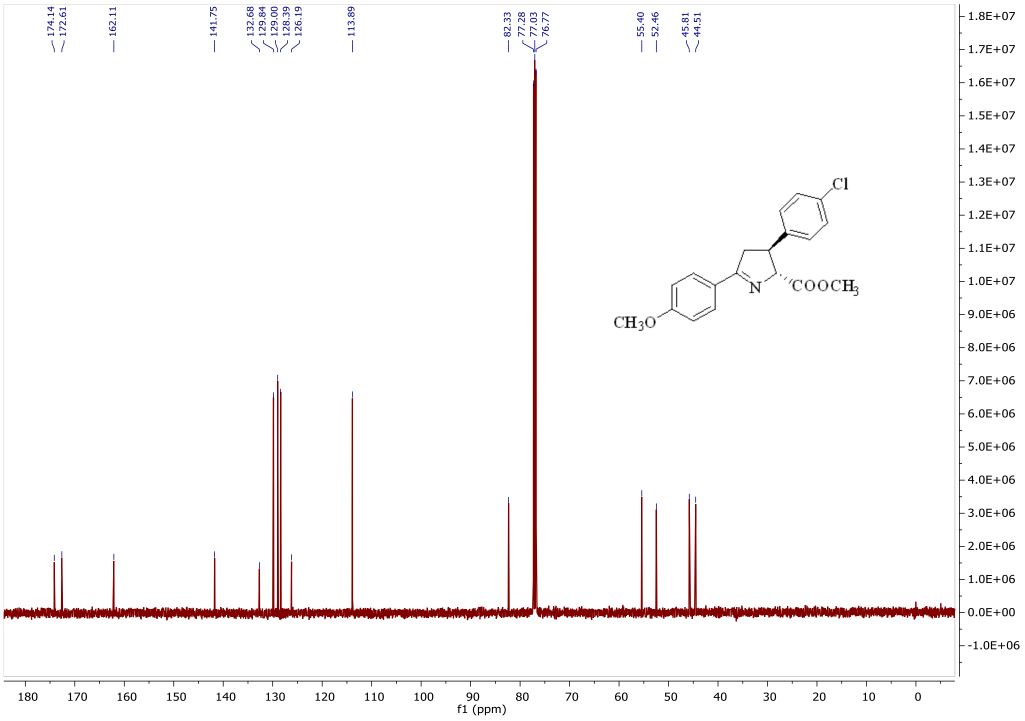

Figure S187: ^{13}C NMR spectrum of compound *trans*-**5f** ($CDCl_3$).

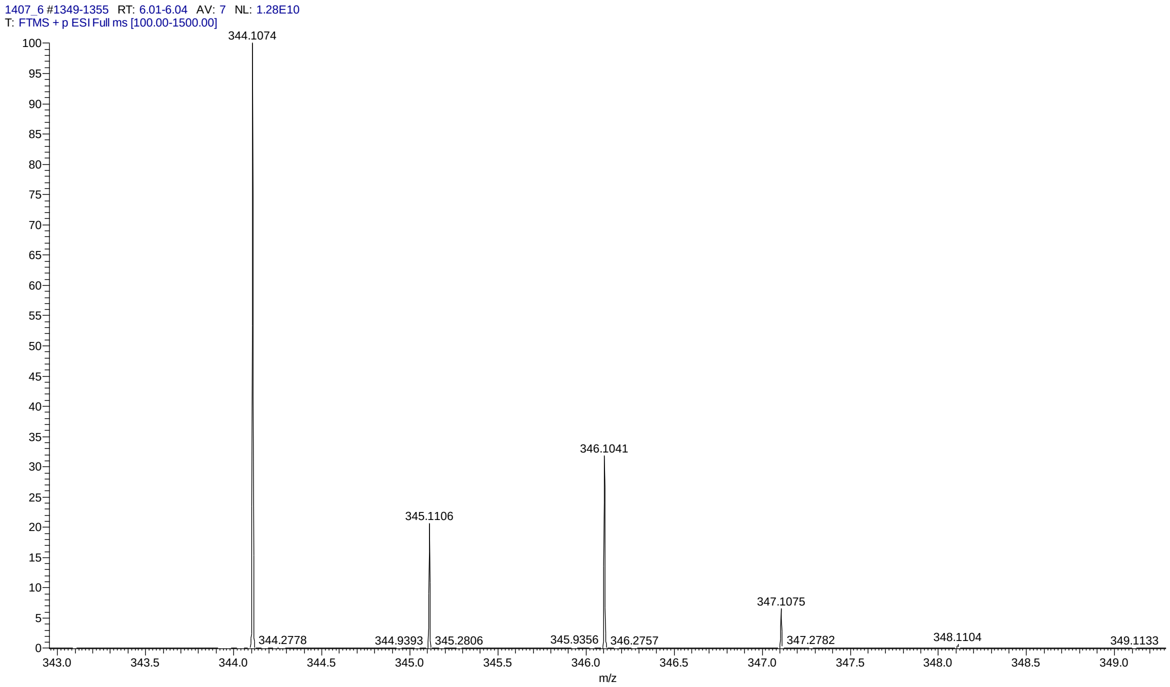

Figure S188: HRMS ESI spectrum of compound *trans*-**5f**.

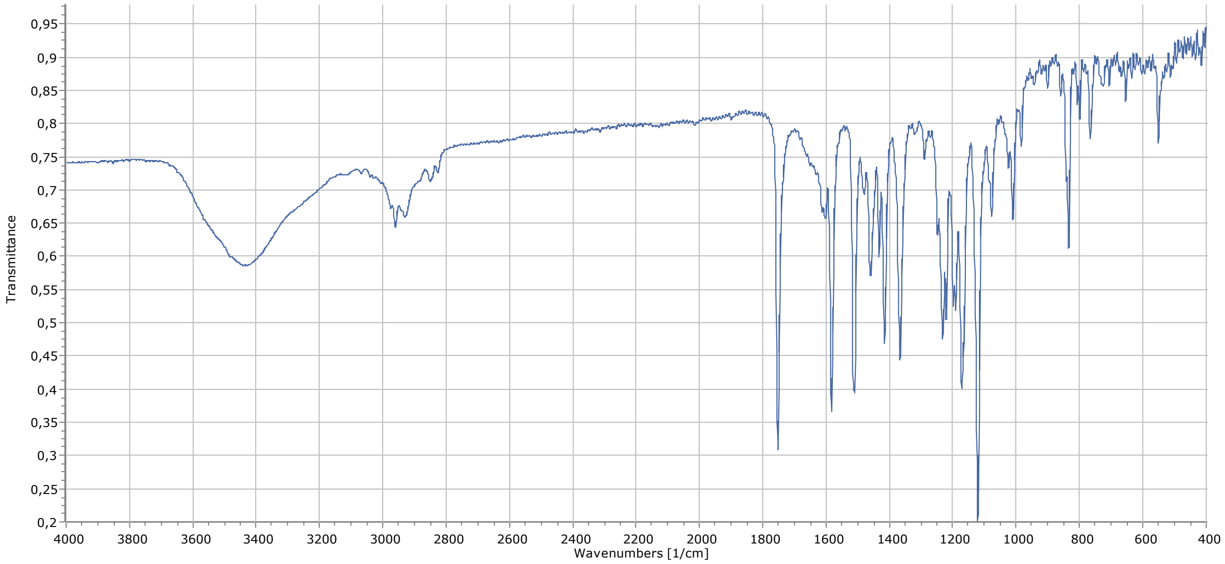

Figure S189: FT-IR spectrum of compound *trans*-**5g** (KBr).

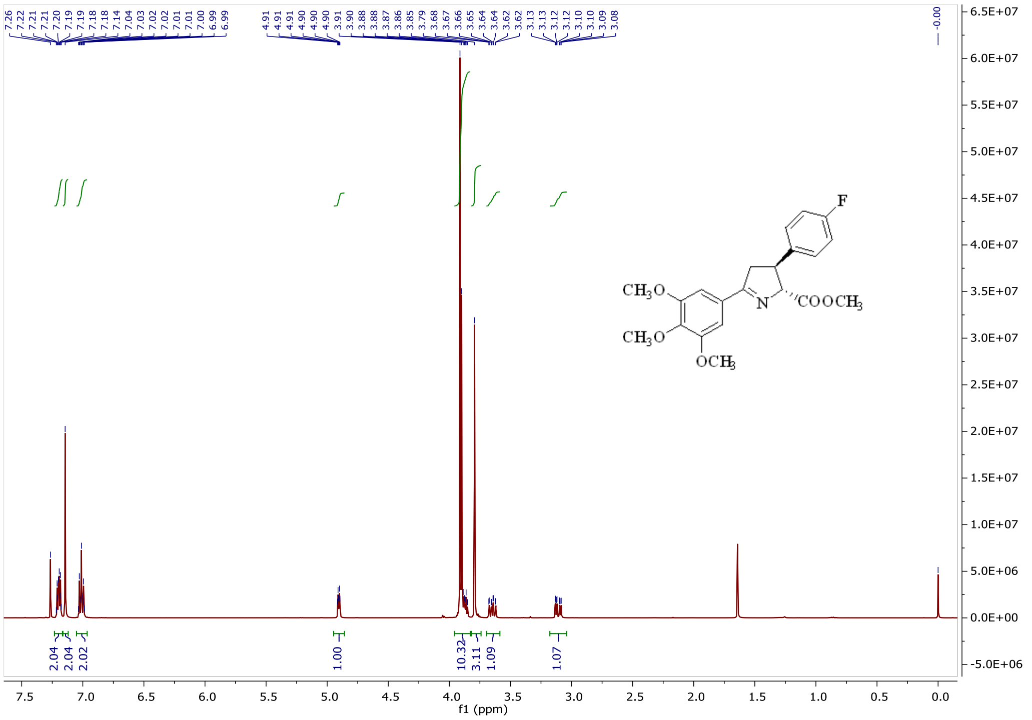

Figure S190: ^{1}H-NMR spectrum of compound *trans*-**5g** ($CDCl_3$).

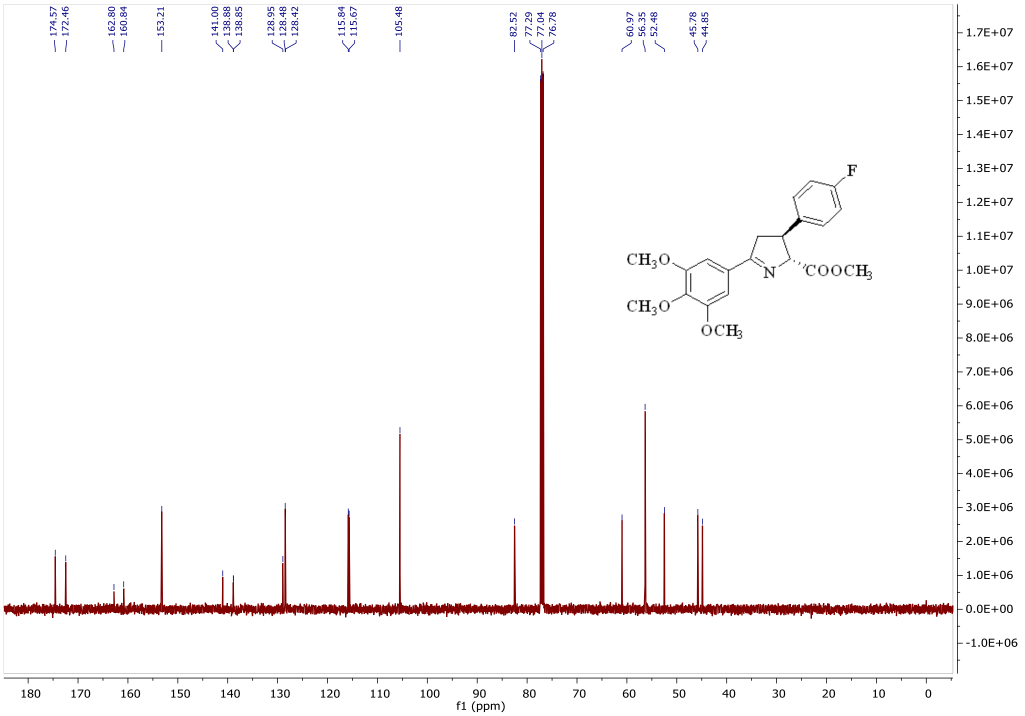

Figure S191: ^{13}C NMR spectrum of compound *trans*-**5g** ($CDCl_3$).

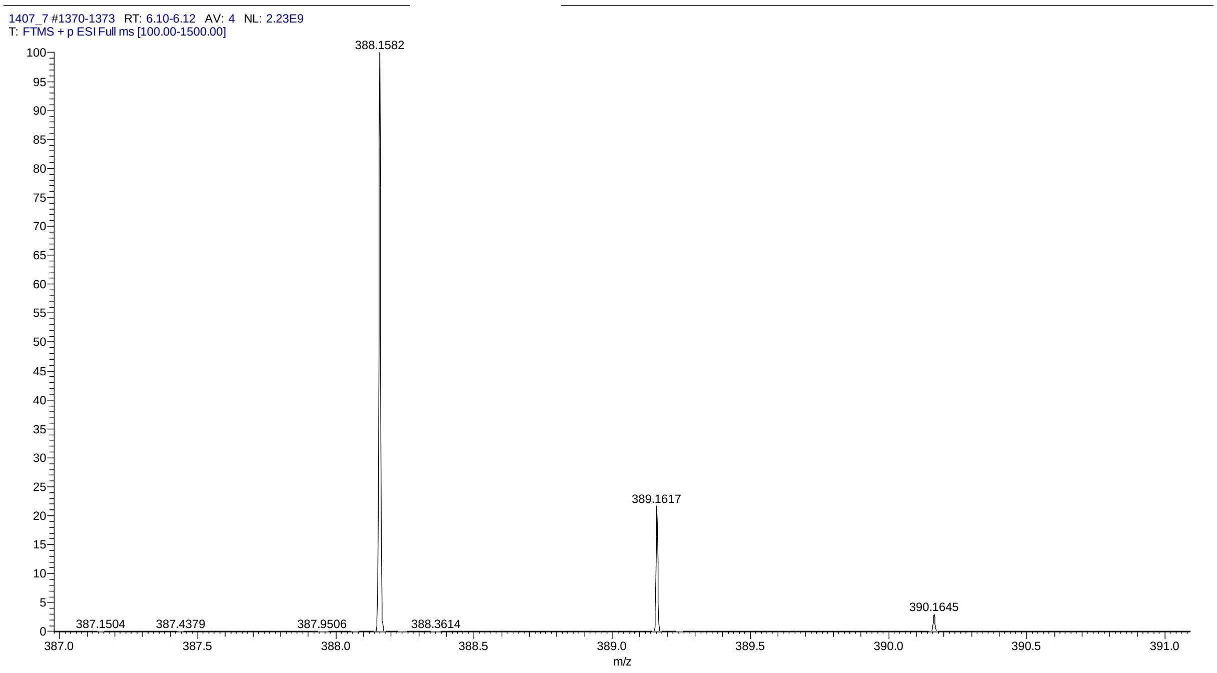

Figure S192: HRMS ESI spectrum of compound *trans*-**5g**.

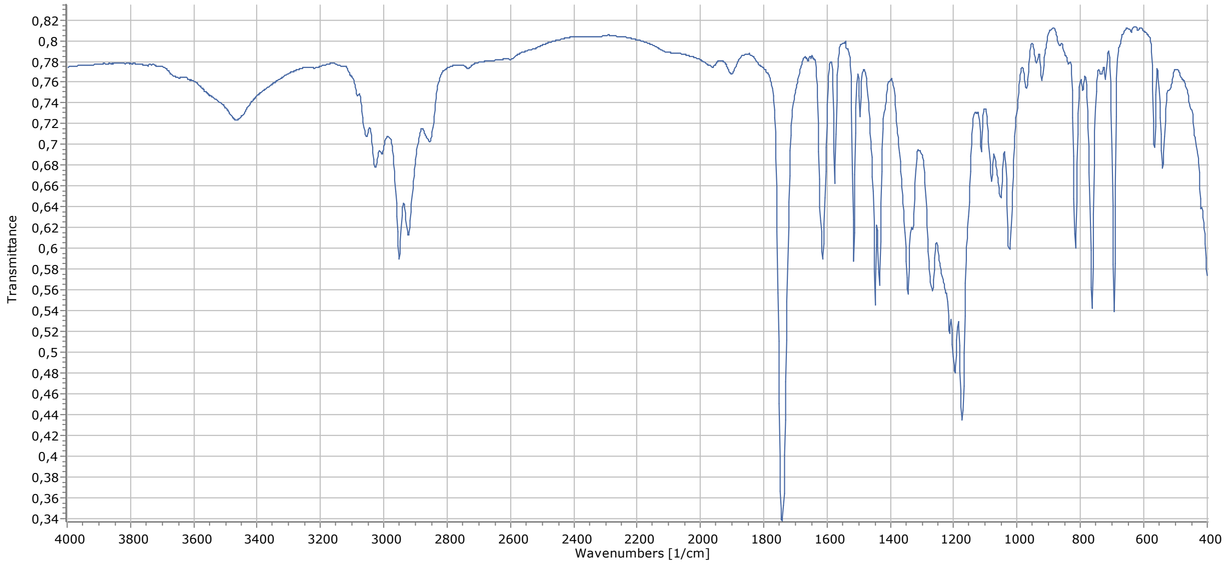

Figure S193: FT-IR spectrum of compound *trans*-**5i** (capillary layer).

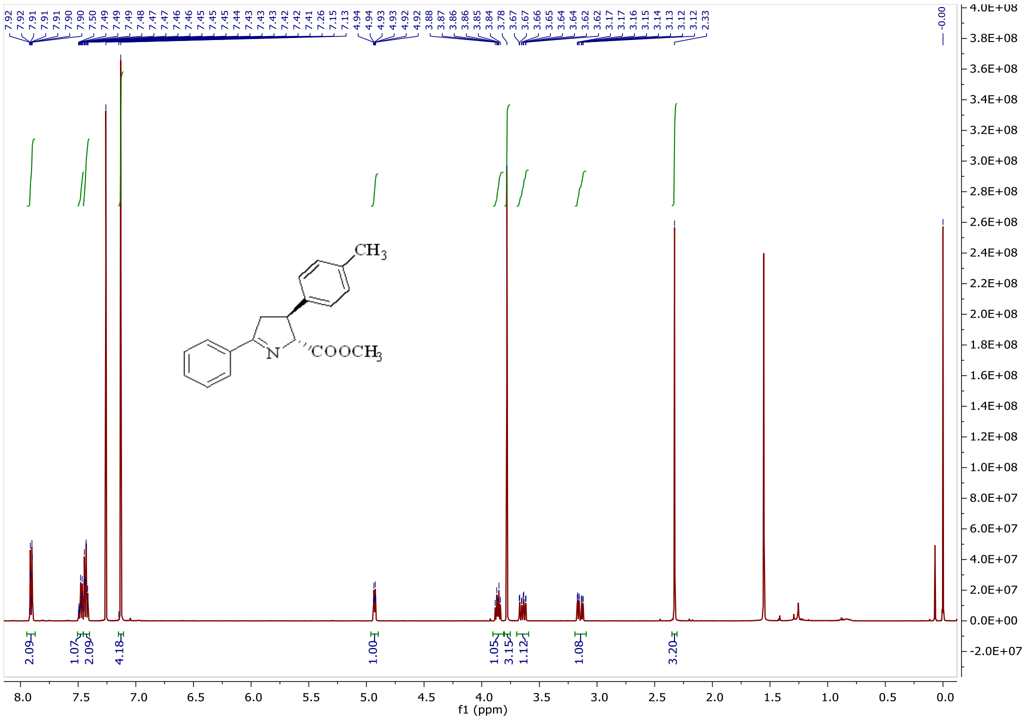

Figure S194: ^{1}H-NMR spectrum of compound *trans*-**5i** ($CDCl_3$).

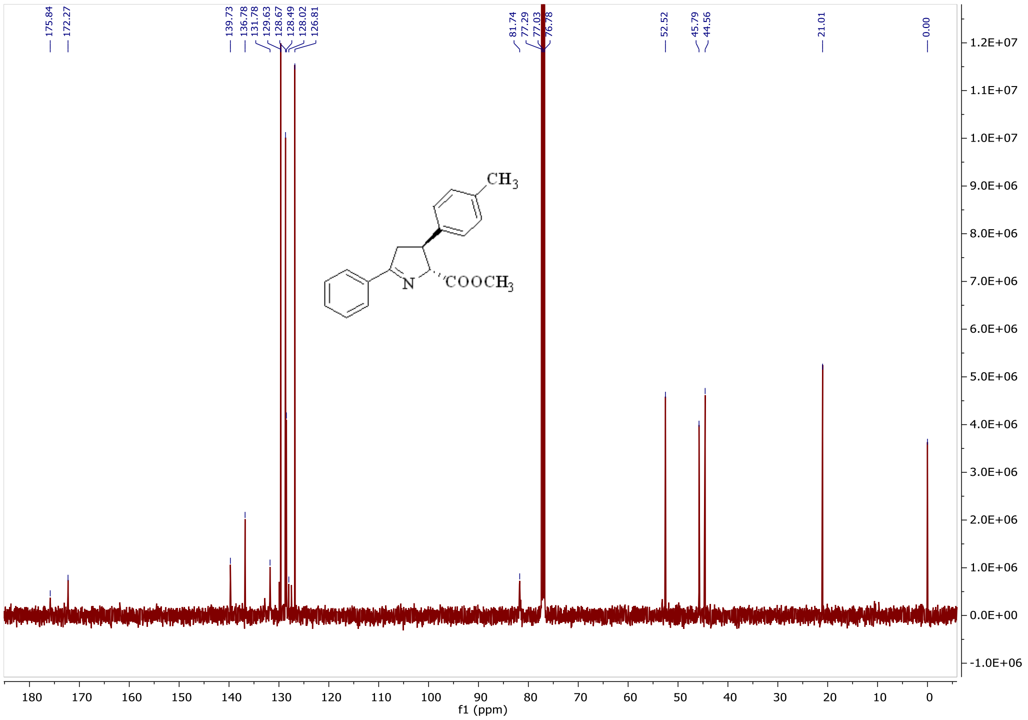

Figure S195: ^{13}C NMR spectrum of compound *trans*-**5i** ($CDCl_3$).

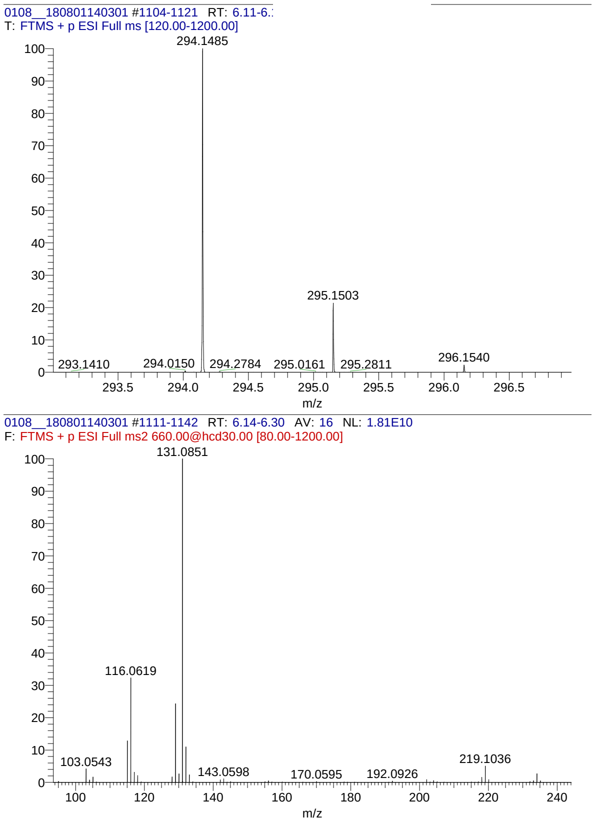

Figure S196: HRMS ESI and HRMS ESI-MS/MS spectrum of compound *trans*-**5i**.

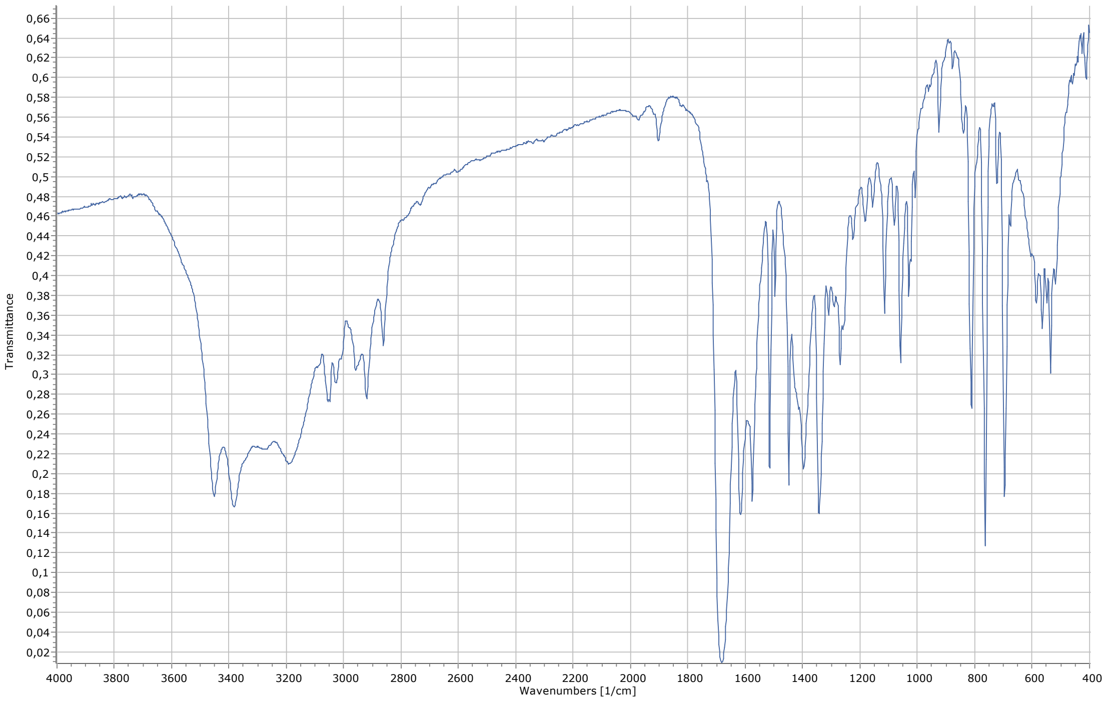

Figure S197: FT-IR spectrum of compound **6i** as a diastereoisomeric mixture (KBr).

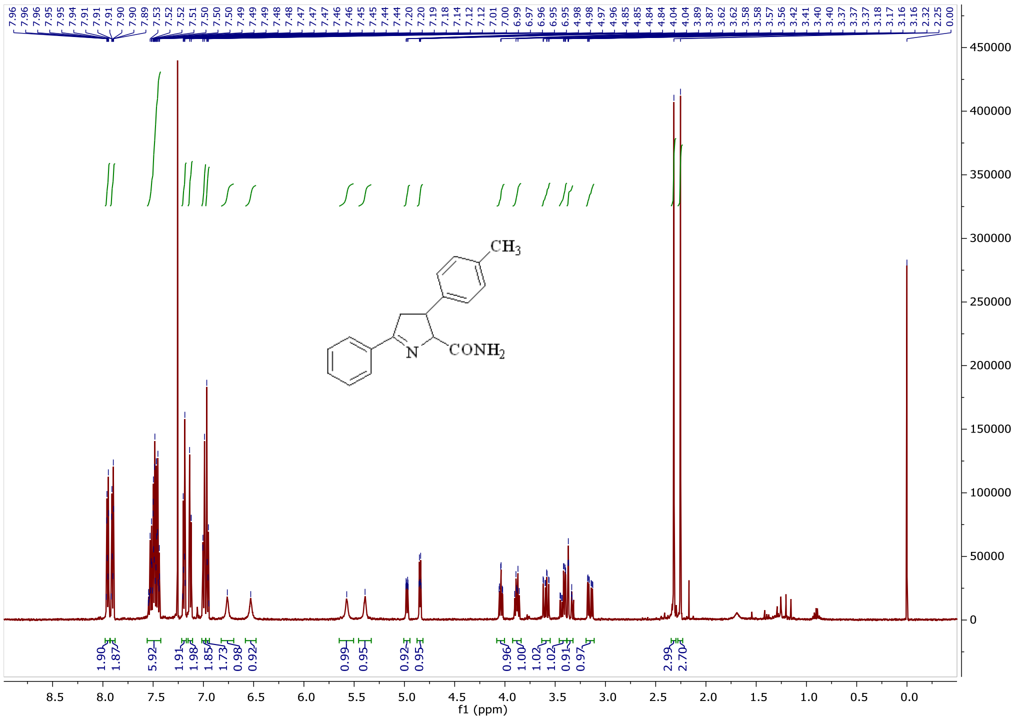

Figure S198: ^{1}H-NMR spectrum of compound **6i** as a diastereoisomeric mixture ($CDCl_3$).

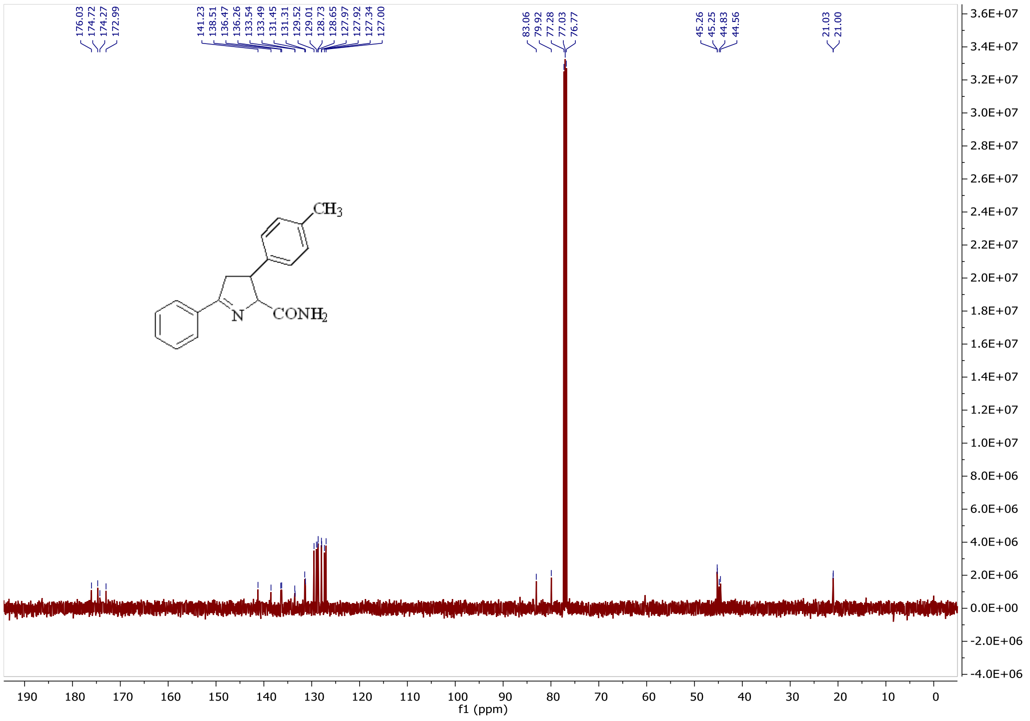

Figure S199: ^{13}C NMR spectrum of compound **6i** as a diastereoisomeric mixture ($CDCl_3$).

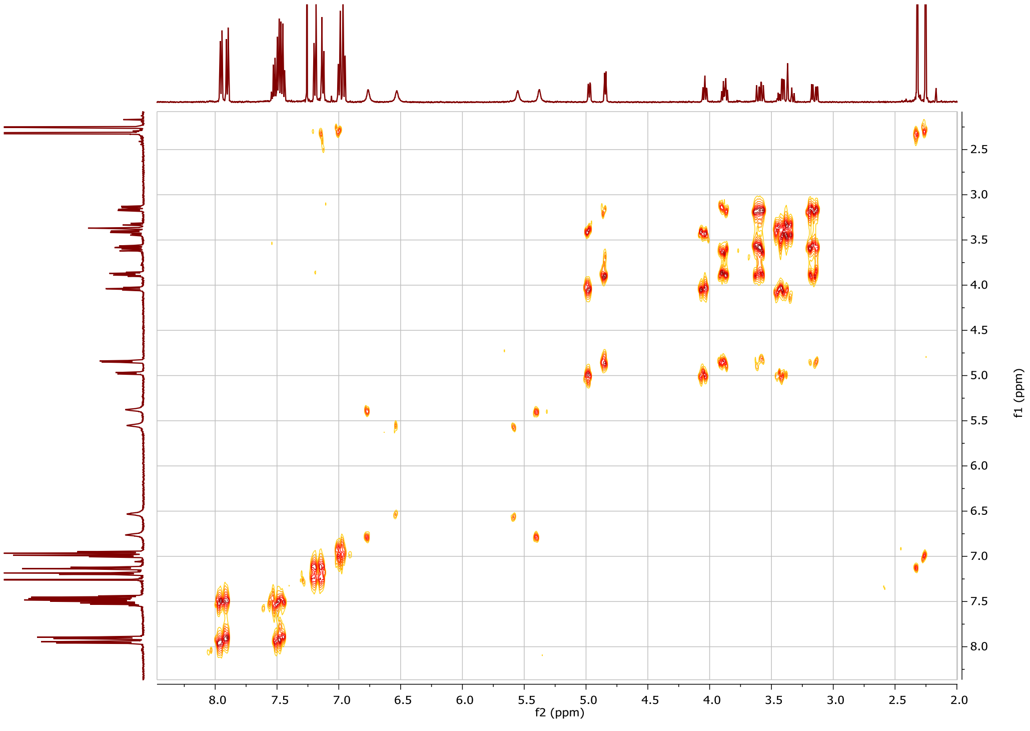

Figure S200: ^1H-^1H COSY spectrum of compound **6i** as a diastereoisomeric mixture ($CDCl_3$).

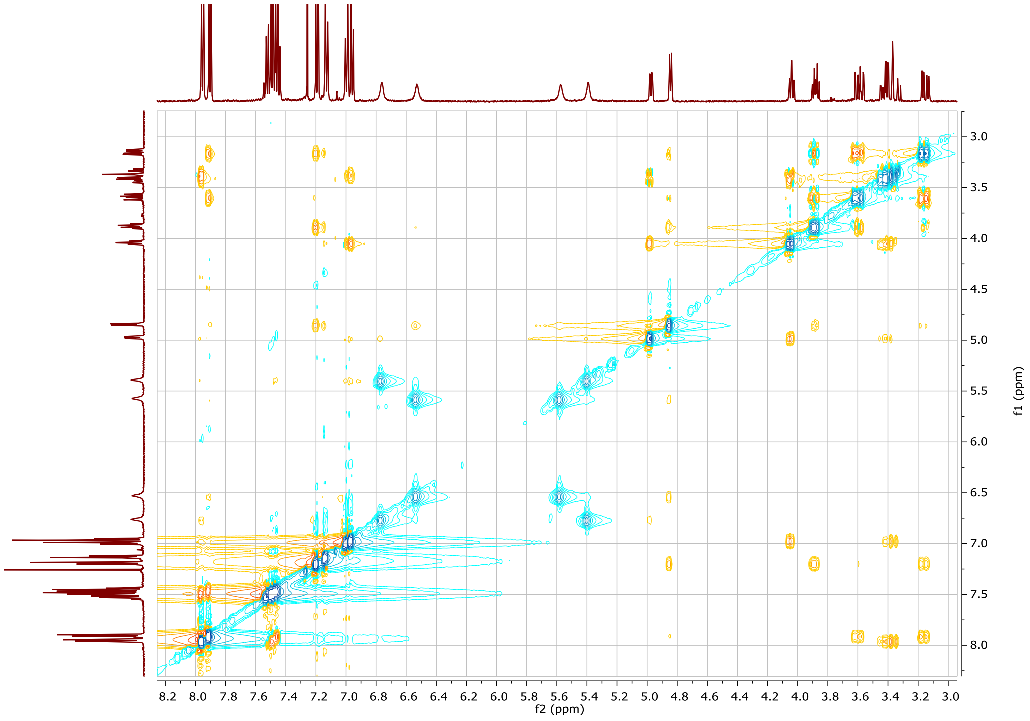

Figure S201: 2D-NOESY spectrum of compound **6i** as a diastereoisomeric mixture ($CDCl_3$).

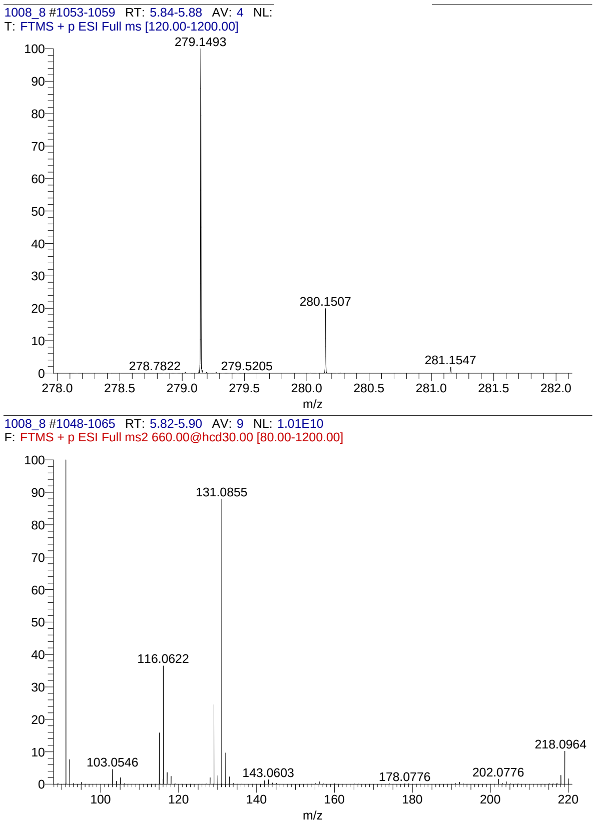

Figure S202: HRMS ESI and HRMS ESI-MS/MS spectrum of compound **6i** as a diastereoisomeric mixture.

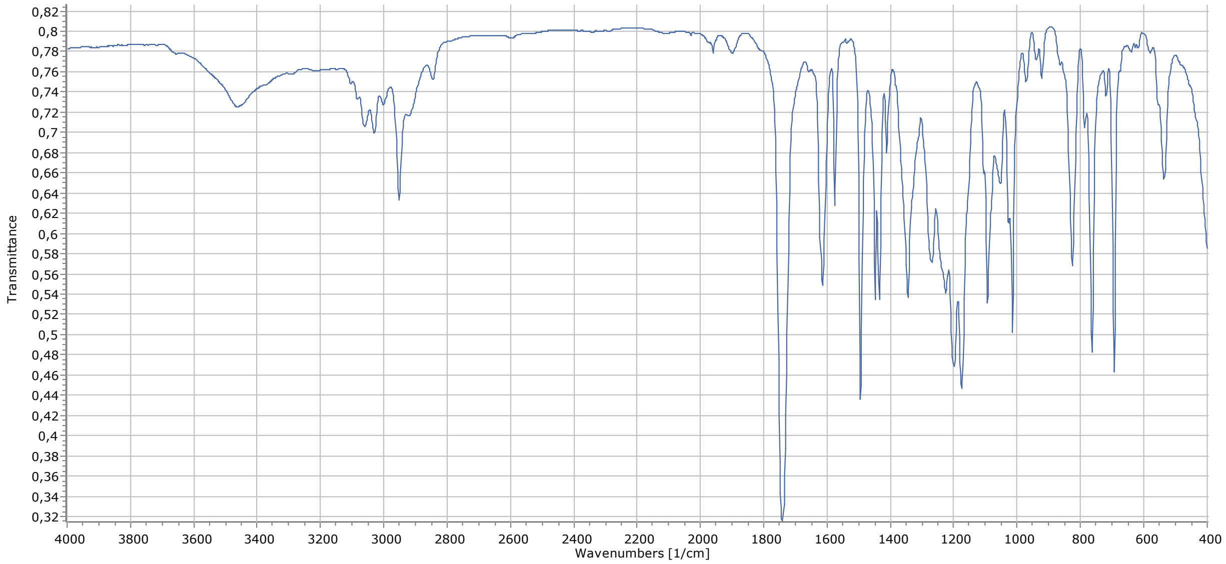

Figure S203: FT-IR spectrum of compound *trans*-**5j** (capillary layer).

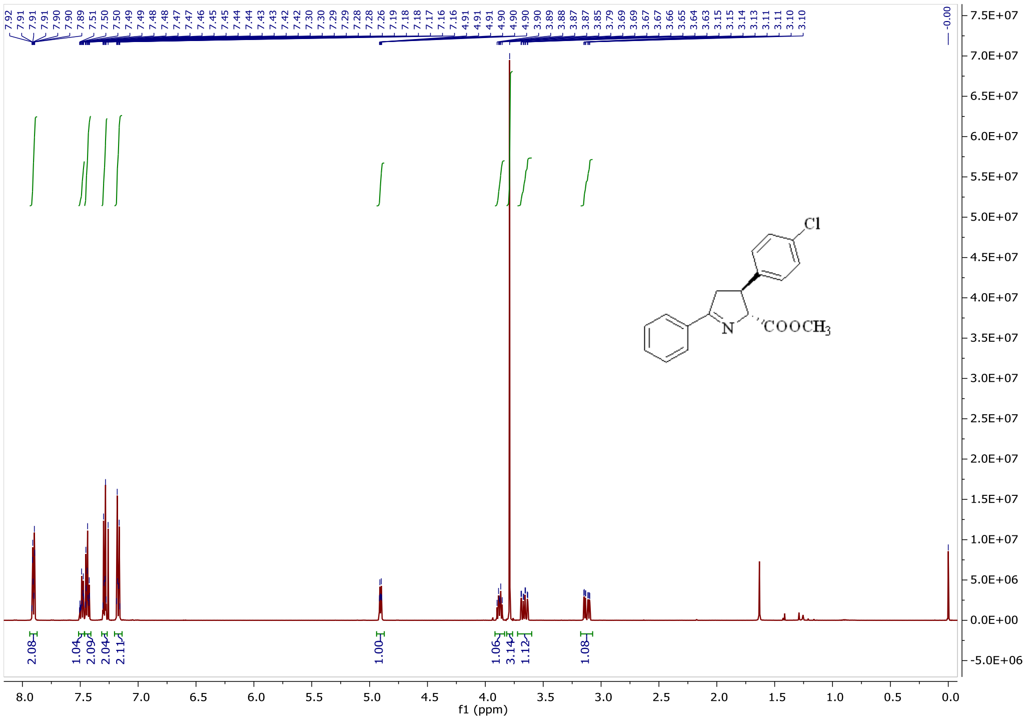

Figure S204: ^{1}H-NMR spectrum of compound *trans*-**5j** ($CDCl_3$).

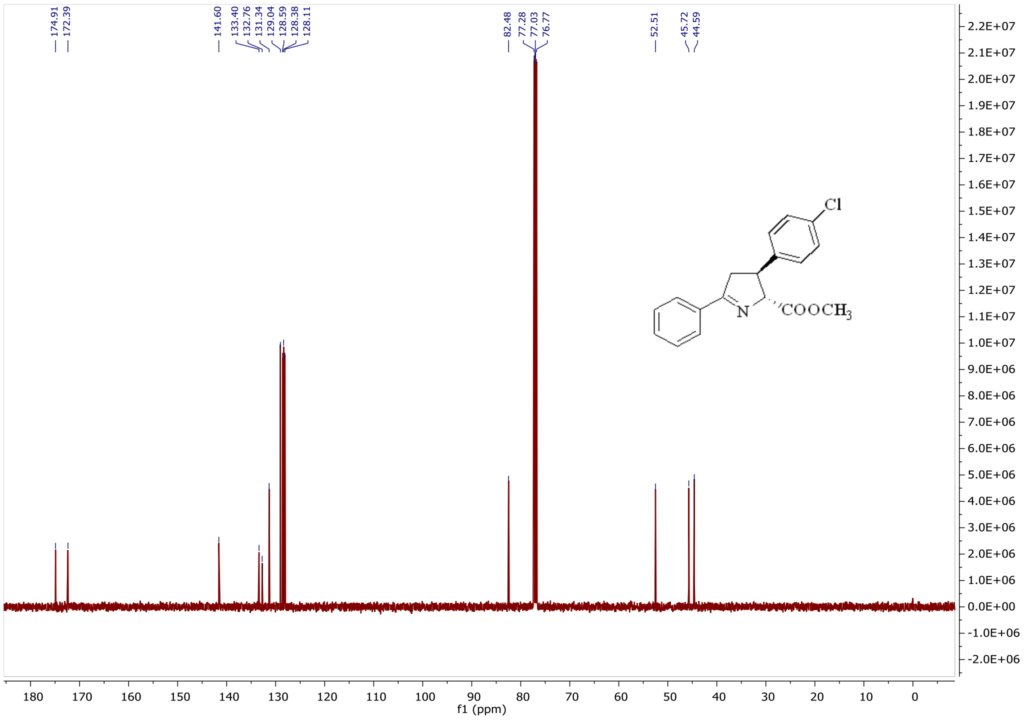

Figure S205: ^{13}C NMR spectrum of compound *trans*-**5j** ($CDCl_3$).

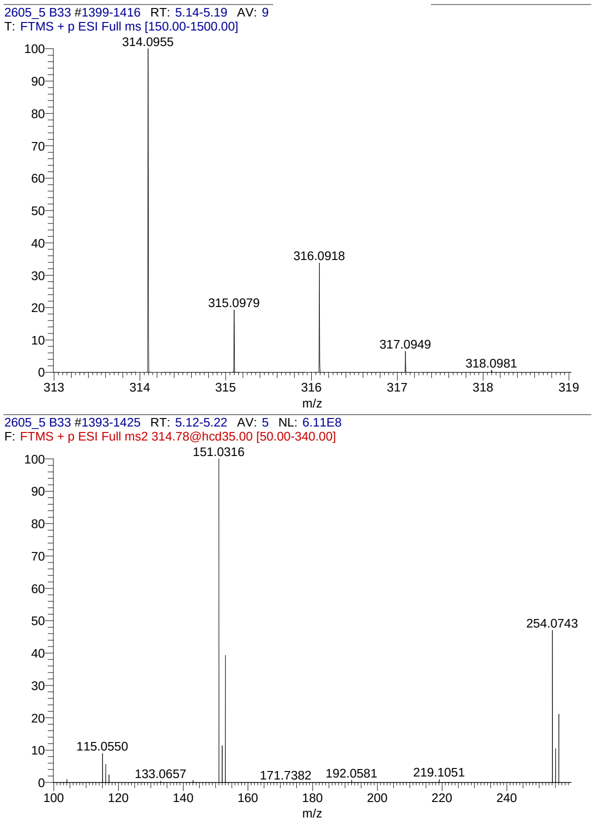

Figure S206: HRMS ESI and HRMS ESI-MS/MS spectrum of compound *trans*-**5j**.

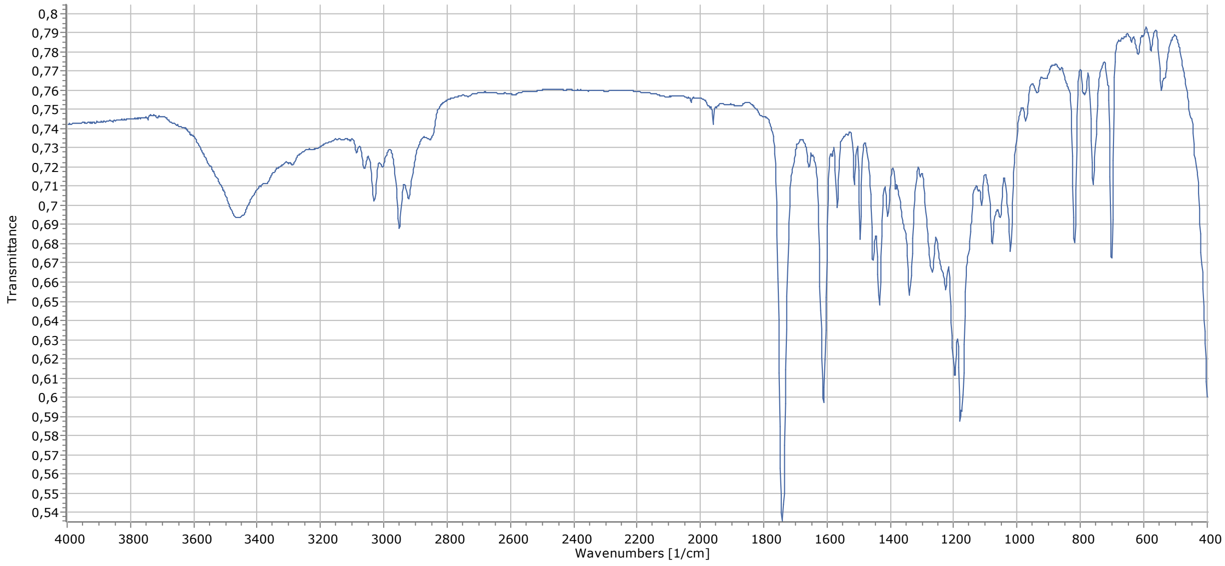

Figure S207: FT-IR spectrum of compound *trans*-**5k** (capillary layer).

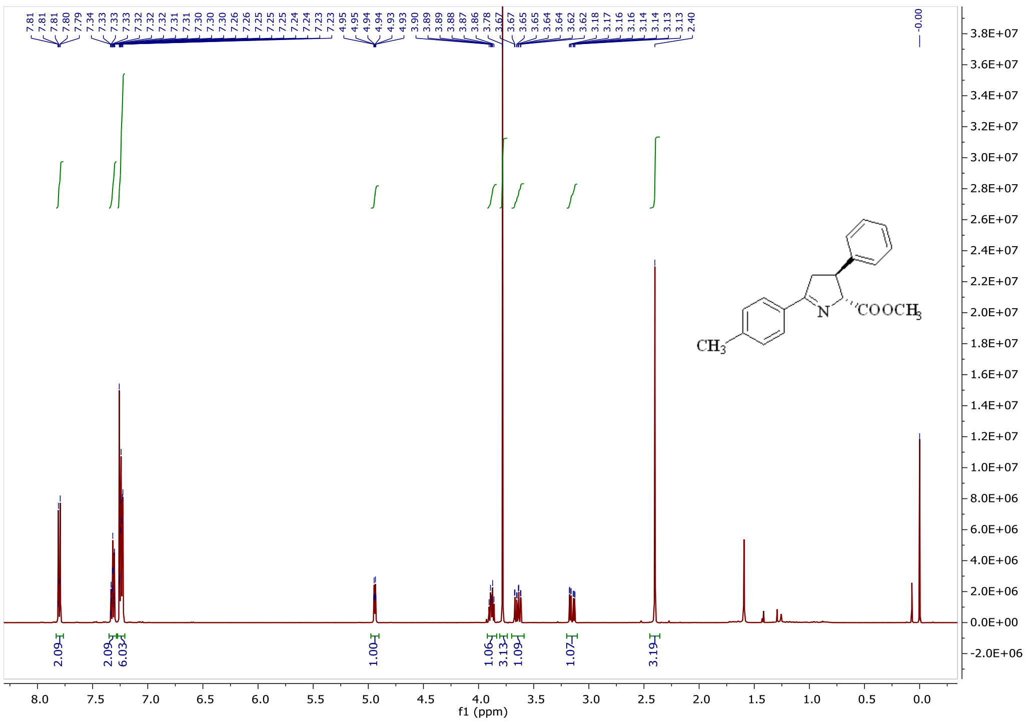

Figure S208: ^{1}H-NMR spectrum of compound *trans*-**5k** ($CDCl_3$).

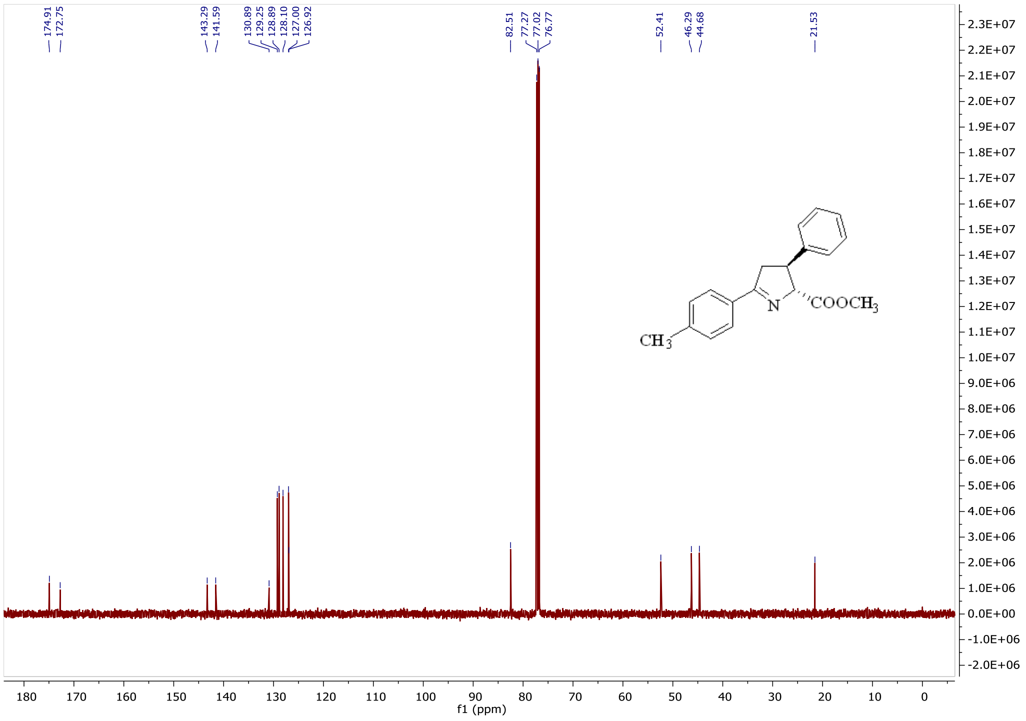

Figure S209: ^{13}C NMR spectrum of compound *trans*-**5k** ($CDCl_3$).

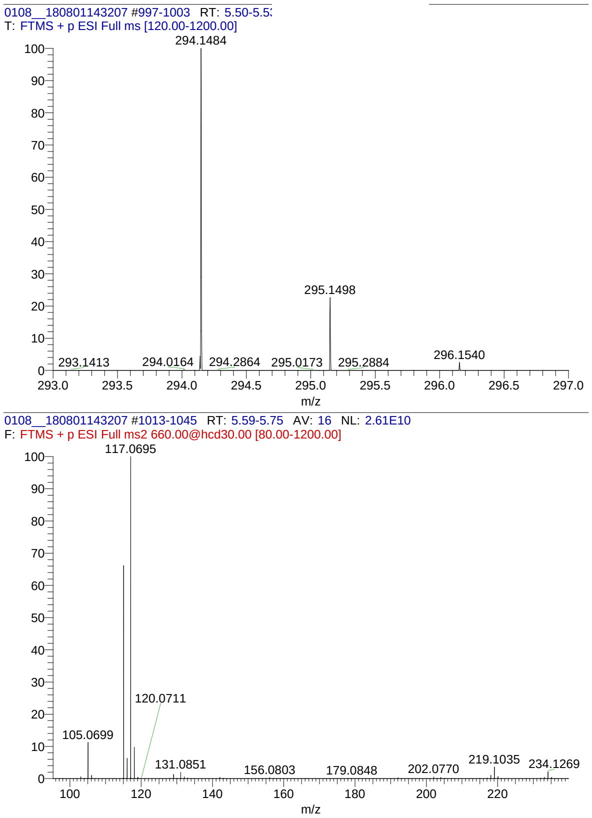

Figure S210: HRMS ESI and HRMS ESI-MS/MS spectrum of compound *trans*-**5k**.

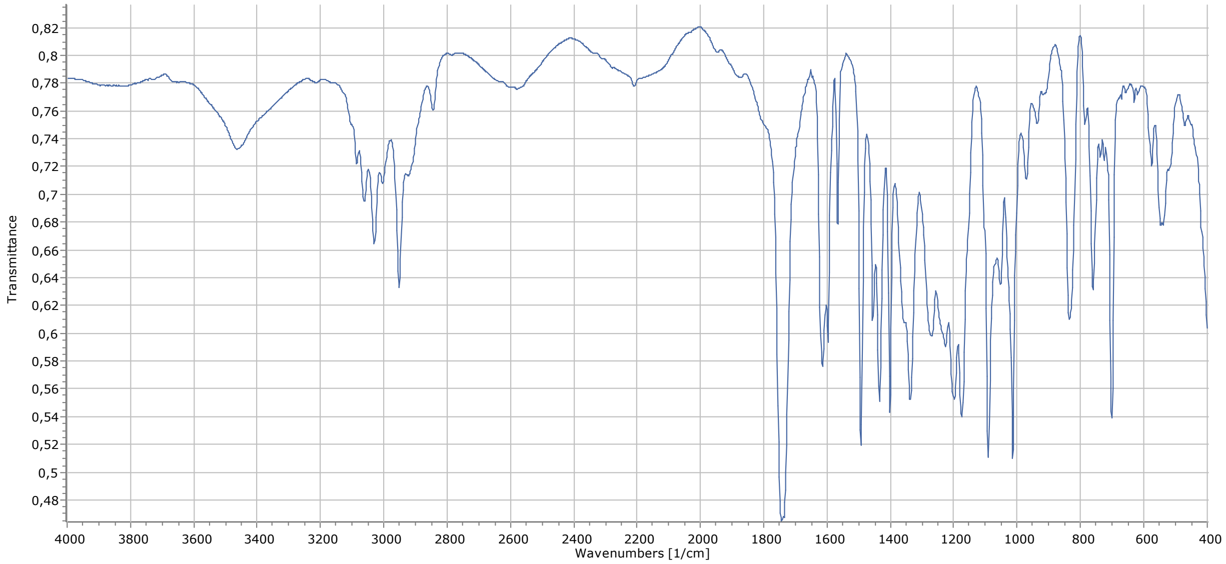

Figure S211: FT-IR spectrum of compound *trans*-**5l** (capillary layer).

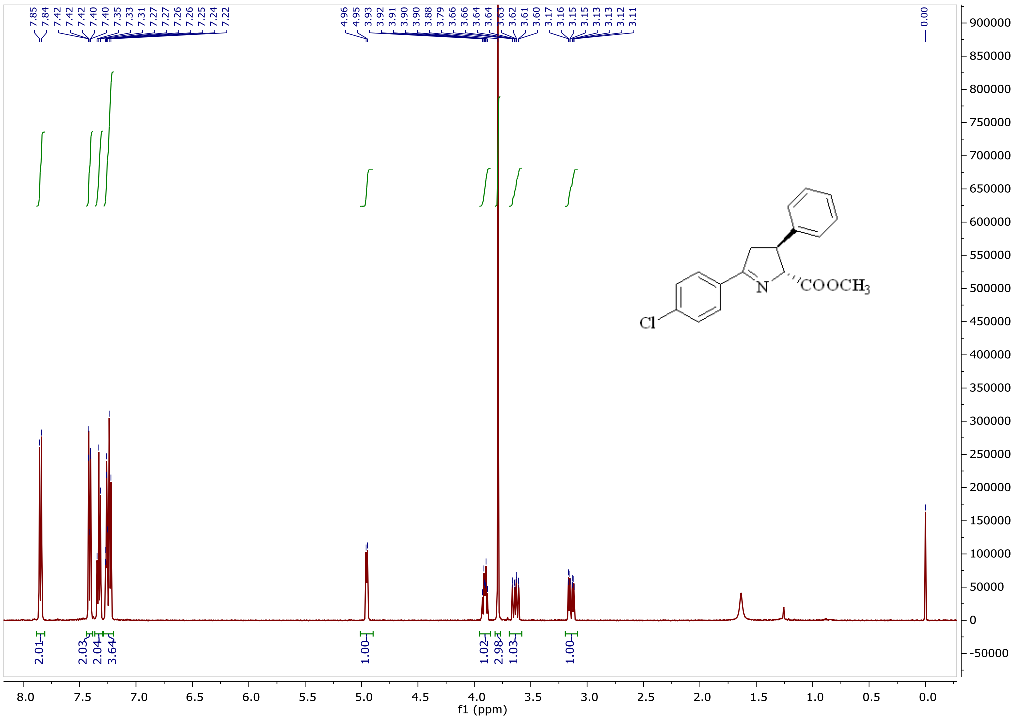

Figure S212: ^{1}H-NMR spectrum of compound *trans*-**5l** ($CDCl_3$).

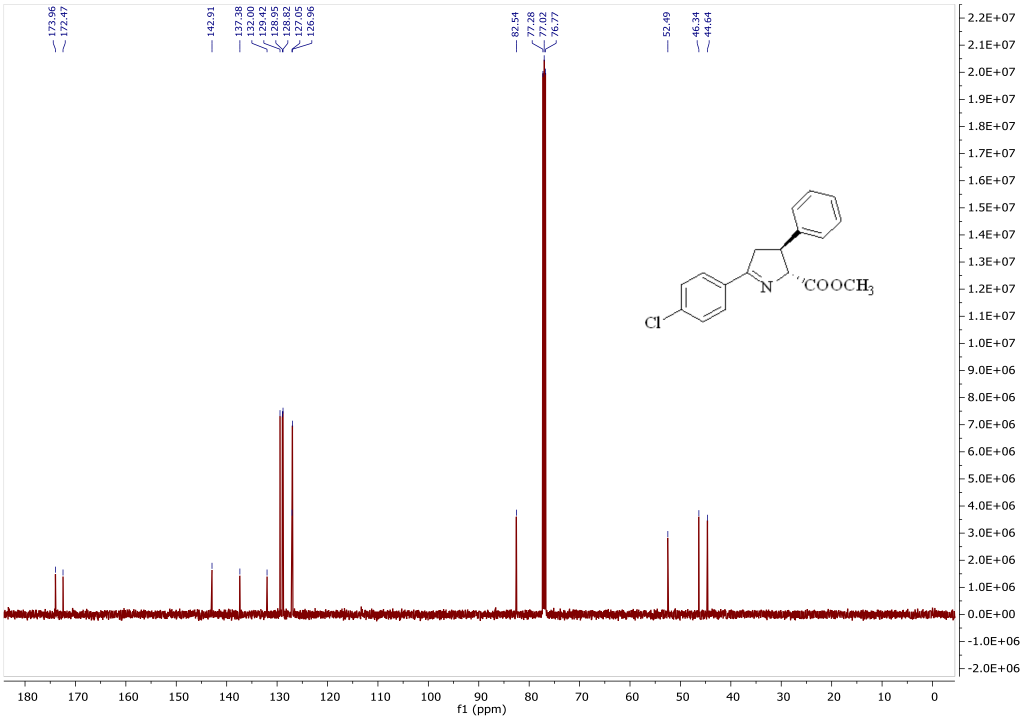

Figure S213: ^{13}C NMR spectrum of compound *trans*-**5l** ($CDCl_3$).

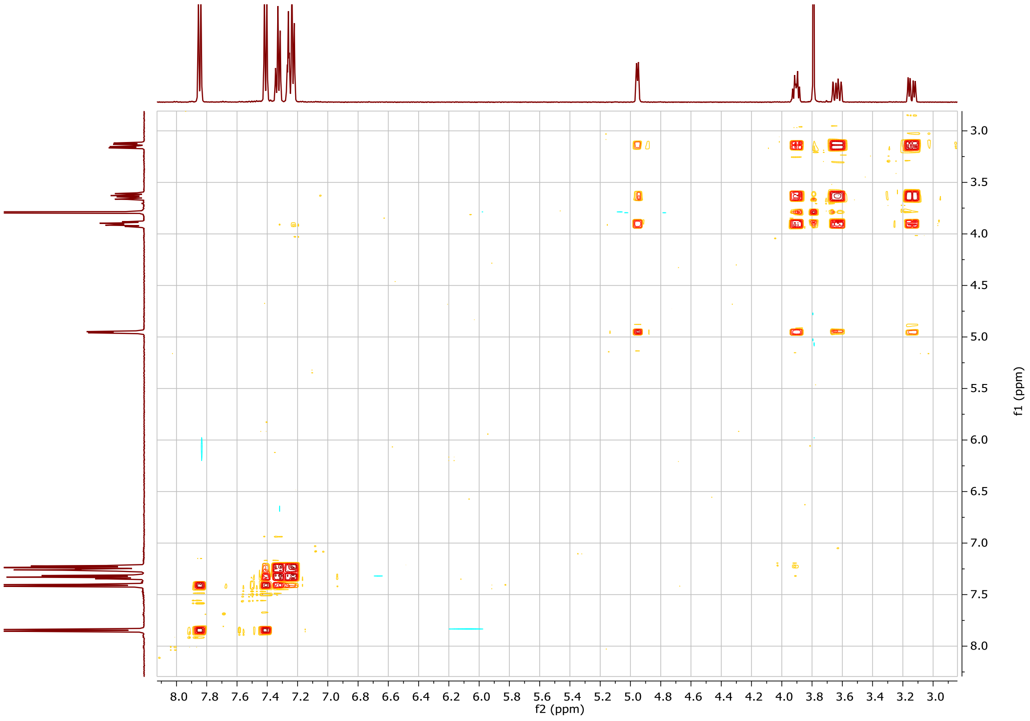

Figure S214: ^{1}H-^{1}H COSY spectrum of compound *trans*-**5l** ($CDCl_3$).

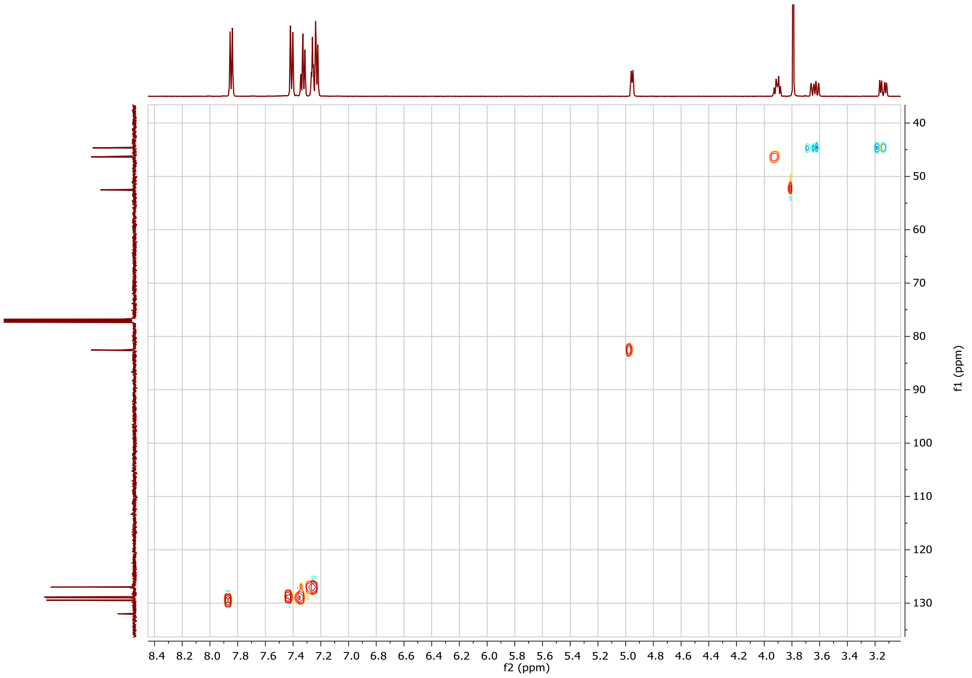

Figure S215: HSQC spectrum of compound *trans*-**5l** ($CDCl_3$).

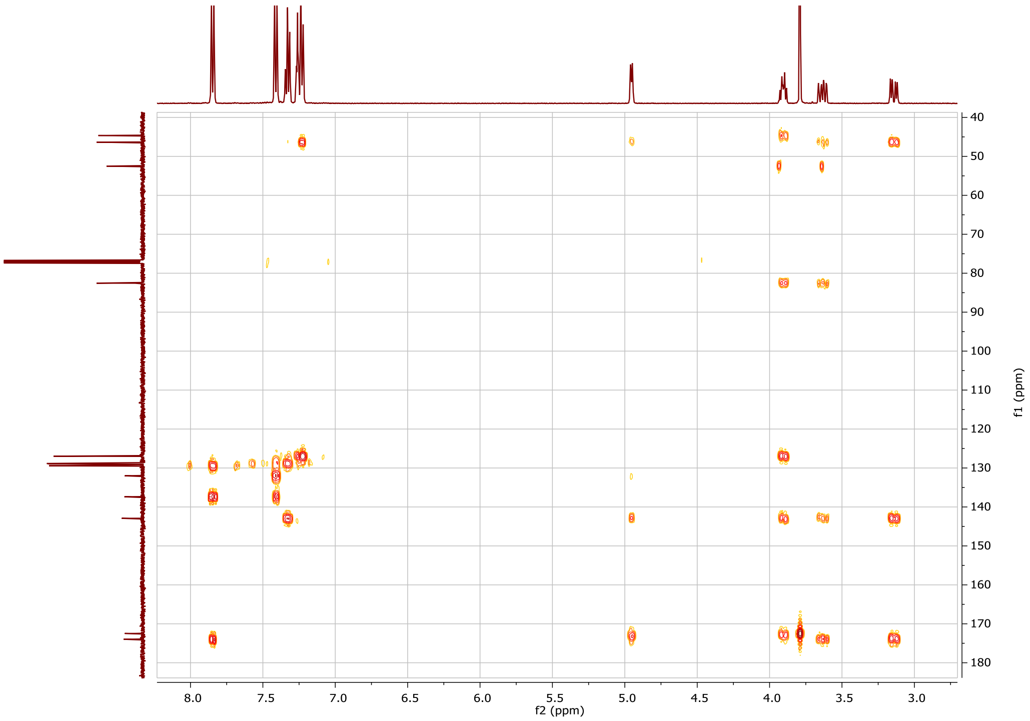

Figure S216: HMBC spectrum of compound *trans*-**5l** ($CDCl_3$).

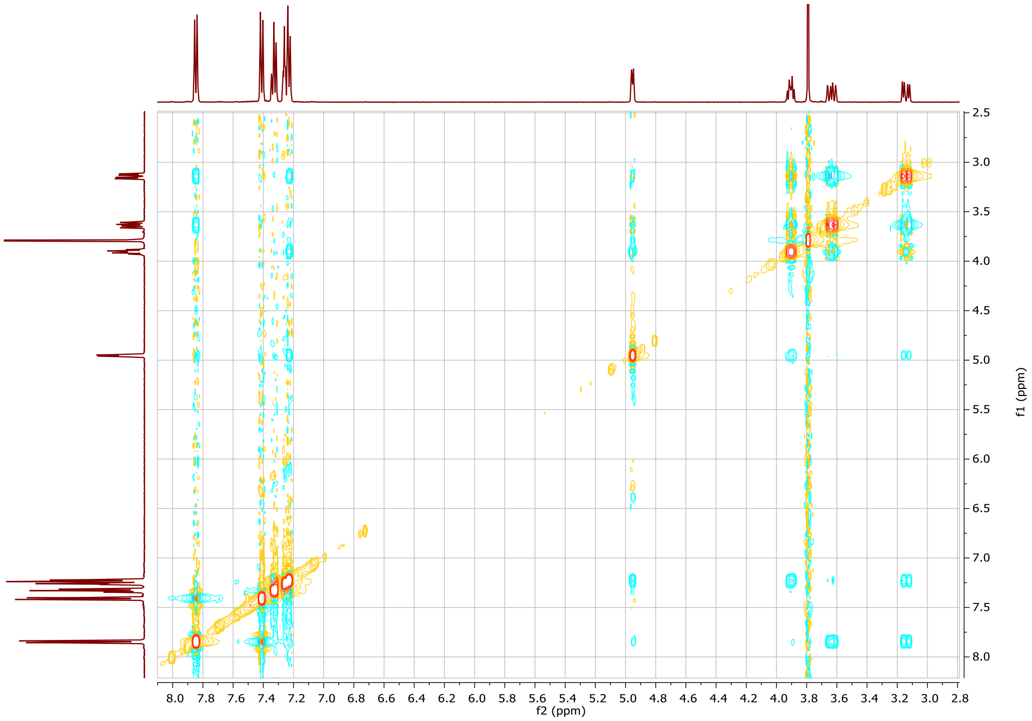

Figure S217: 2D-NOESY spectrum of compound ***trans*-5l** ($CDCl_3$).

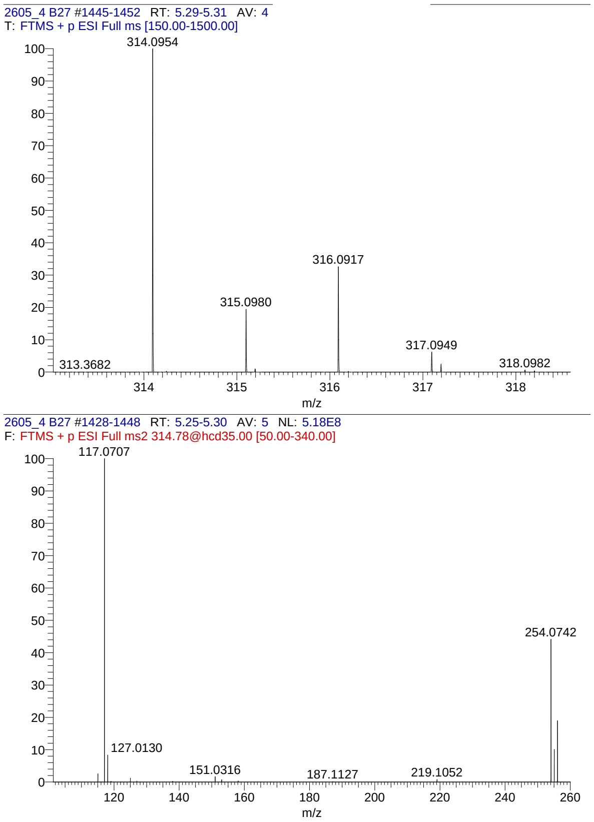

Figure S218: HRMS ESI and HRMS ESI-MS/MS spectrum of compound *trans*-**5l**.

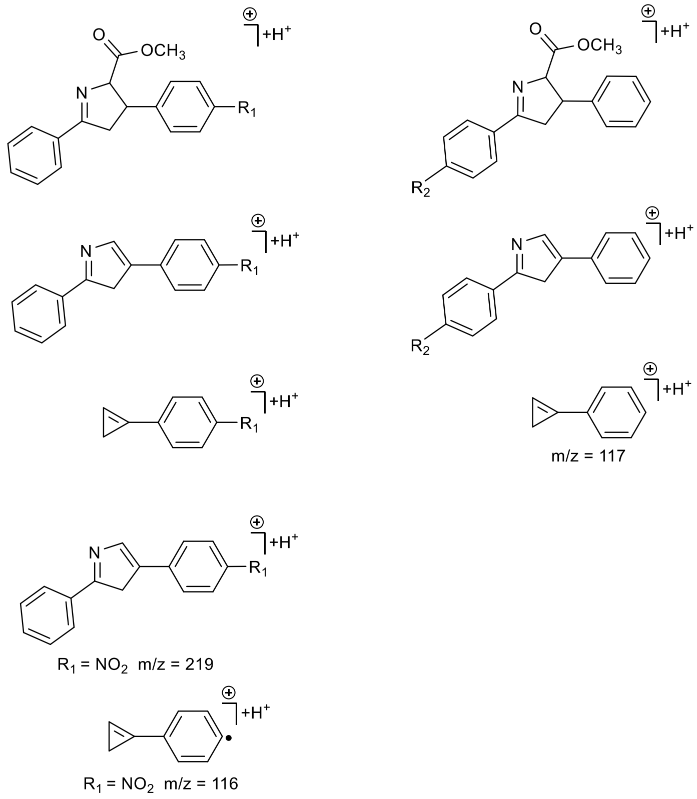

Figure S219: Proposed structures of the most abundant fragment ions observed in a MS/MS spectra of compounds *trans*-**5a-5g**, and *trans*-**5i-l**.

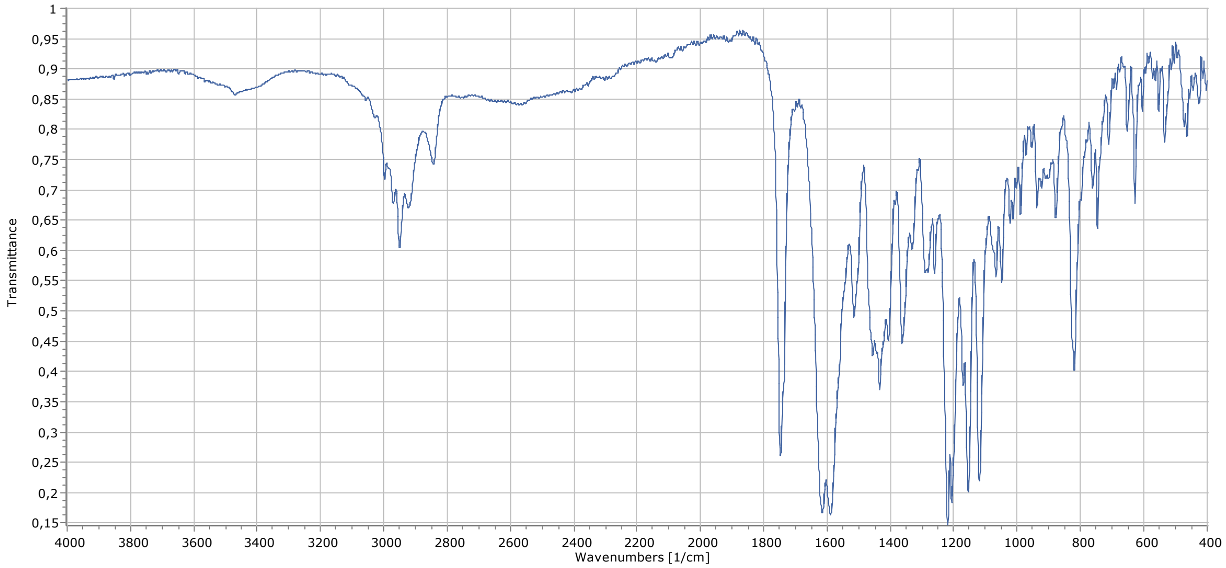

Figure S220: FT-IR spectrum of compound *trans*-**5m** (KBr).

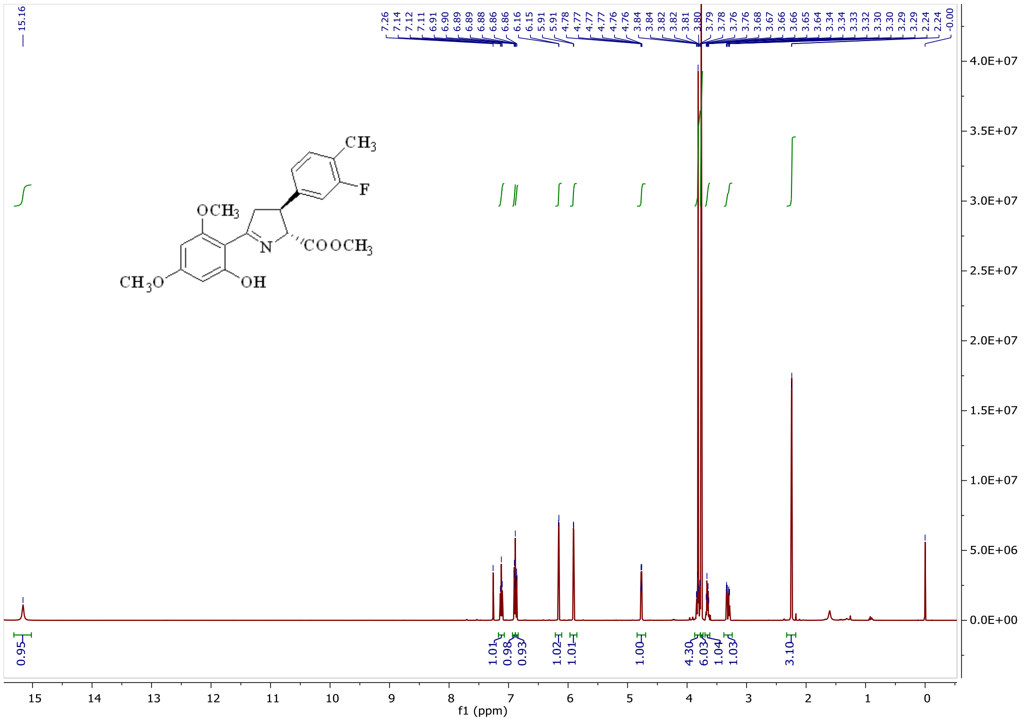

Figure S221: ^{1}H-NMR spectrum of compound *trans*-**5m** ($CDCl_3$).

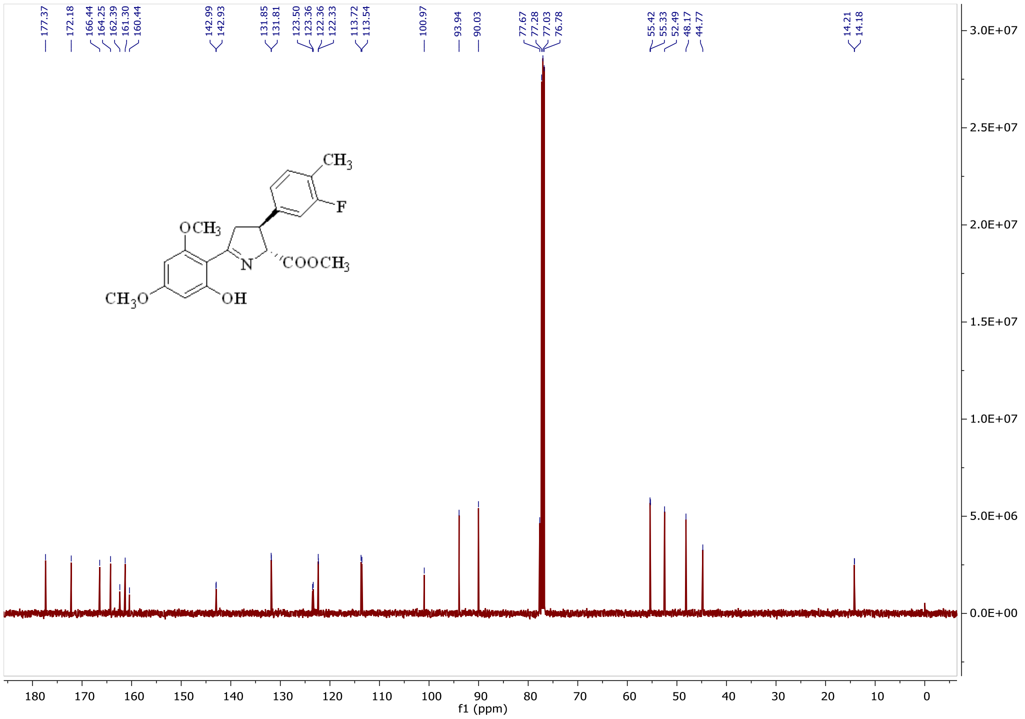

Figure S222: ^{13}C NMR spectrum of compound *trans*-**5m** ($CDCl_3$).

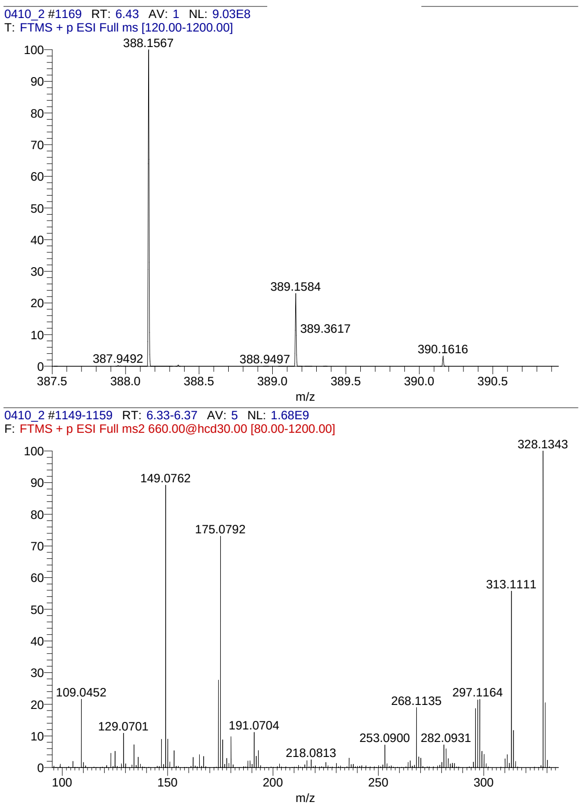

Figure S223: HRMS ESI and HRMS ESI-MS/MS spectrum of compound *trans*-**5m**.

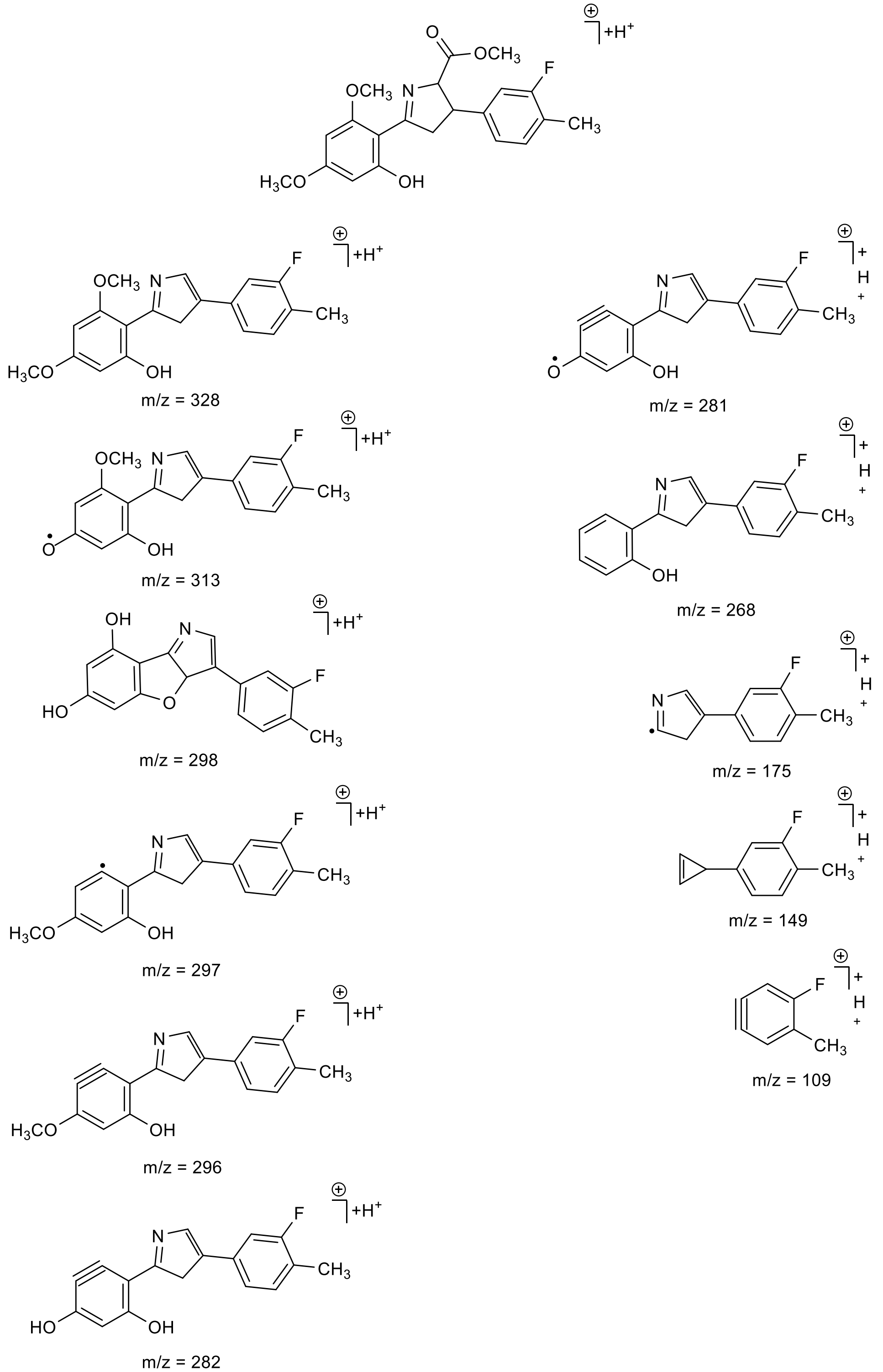

Figure S224: Proposed structures of the most abundant fragment ions observed in a MS/MS spectrum of compound *trans*-**5m**.

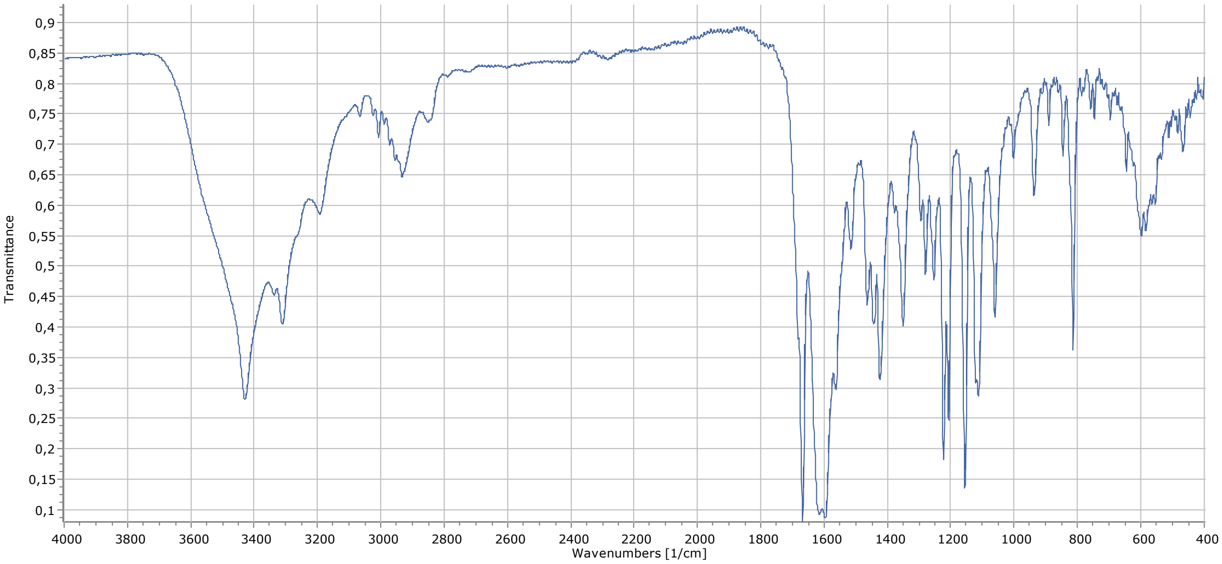

Figure S225: FT-IR spectrum of compound ***cis*-6m** (KBr).

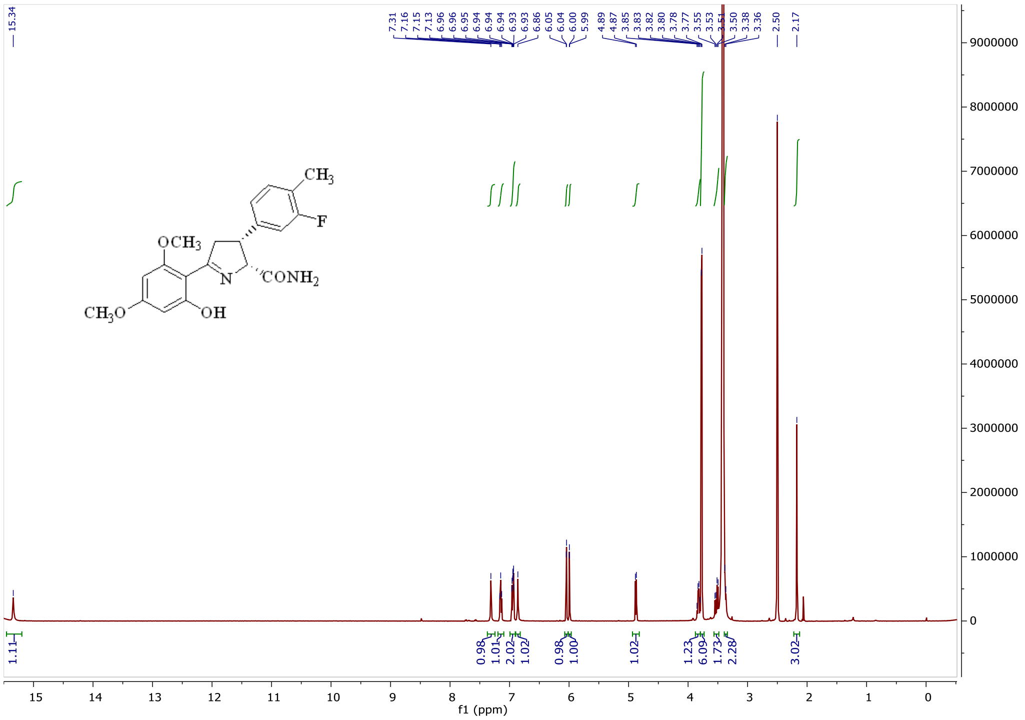

Figure S226: ^{1}H-NMR spectrum of compound *cis*-**6m** (DMSO-d_6).

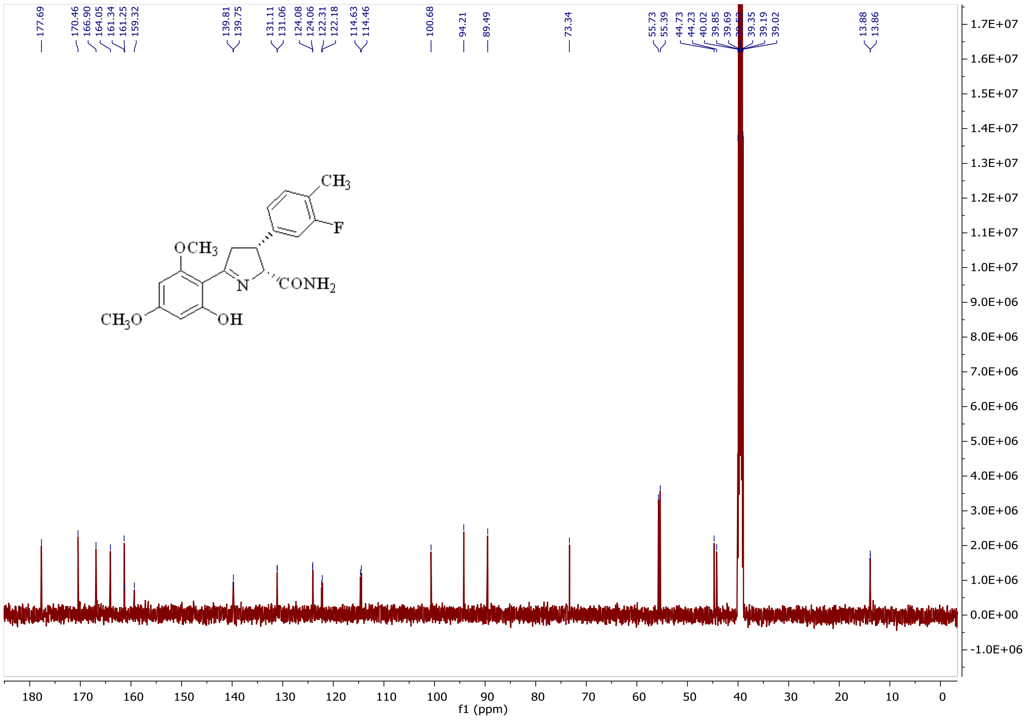

Figure S227: ^{13}C NMR spectrum of compound *cis*-**6m** (DMSO-d$_6$).

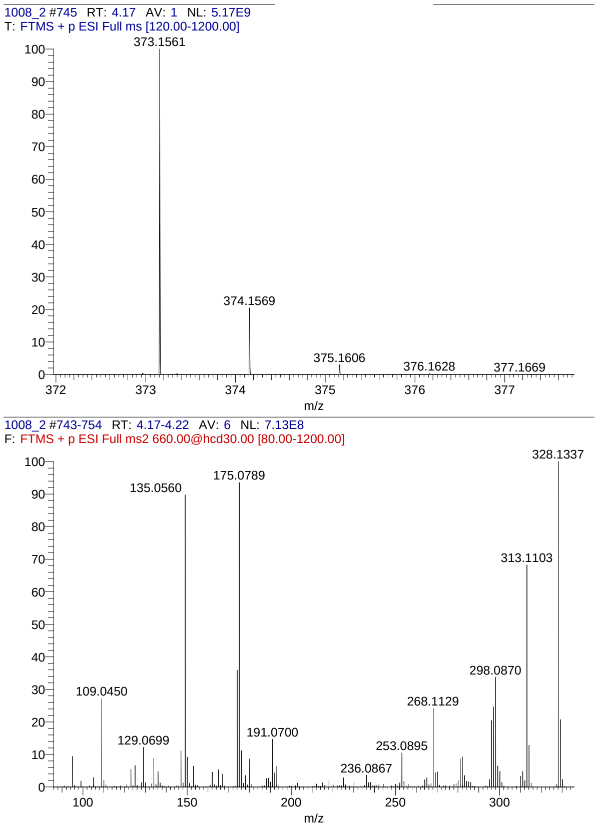

Figure S228: HRMS ESI and HRMS ESI-MS/MS spectrum of compound *cis*-**6m**.

UPLC analyses

The analyses were carried out on Thermo Scientific™ UltiMate™ 3000 Basic Manual system. The chromatograph was connected to a Dionex UltiMate 3000 diode array detector and fluorescence detector Dionex UltiMate 3000. The separation was performed using a Nucleodur C18 Pyramid column, 3.5 μm, EC 125/4 supplied by Macherey-Nagel - Germany. The samples were injected automatically (10.0 μL). The column was thermostatically controlled at 45°C and 0.300 mL/min flow rate was applied, using a linear gradient of 0.1% formic acid in water (solvent A) and 0.1% formic acid in acetonitrile (solvent B). The run time was 21 min, and the flow rate was 0.3 mL/min. The gradient elution program was as follows: 0 min, 40% B; 0–16 min, 40–95% B; 16-18 min 95% B; 18–19 min, 95–40% B; and 40%B for 2 min. Equilibration time was 2 min. The chromatogram was monitored at 215, 270, 280 and 370 nm. UV spectra of individual peaks were recorded in the range of 200–400 nm. Data acquisition was performed using Thermo Scientific™ Dionex™ Chromeleon™ CDS, version 7.x software.

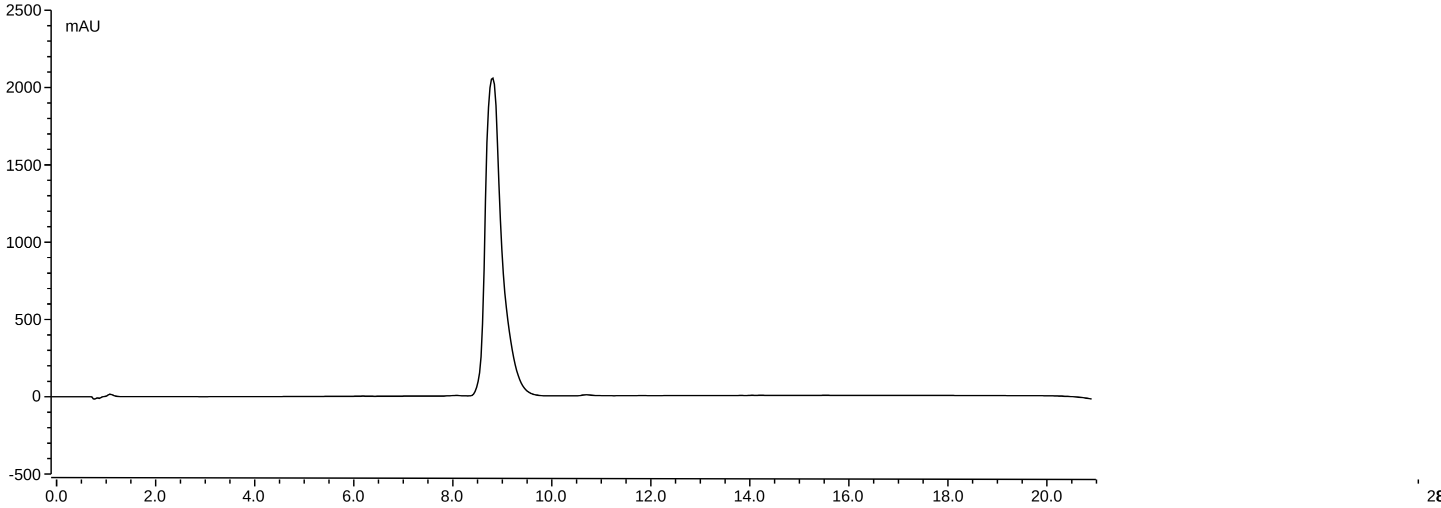

Figure S229: UPLC-DAD chromatogram detected at 270 nm of compound ***trans*-4k**.

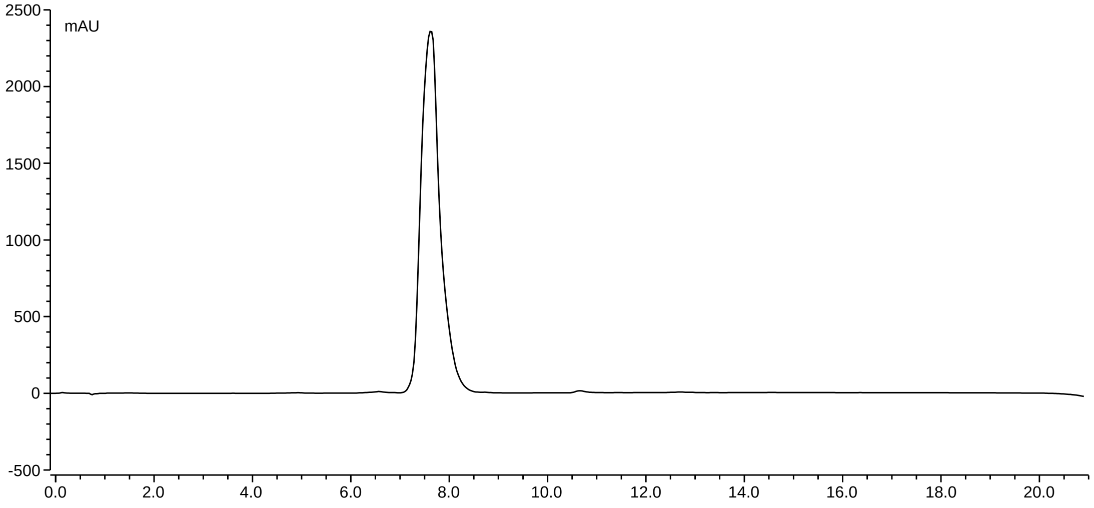

Figure S230: UPLC-DAD chromatogram detected at 270 nm of compound ***cis*-4k**.

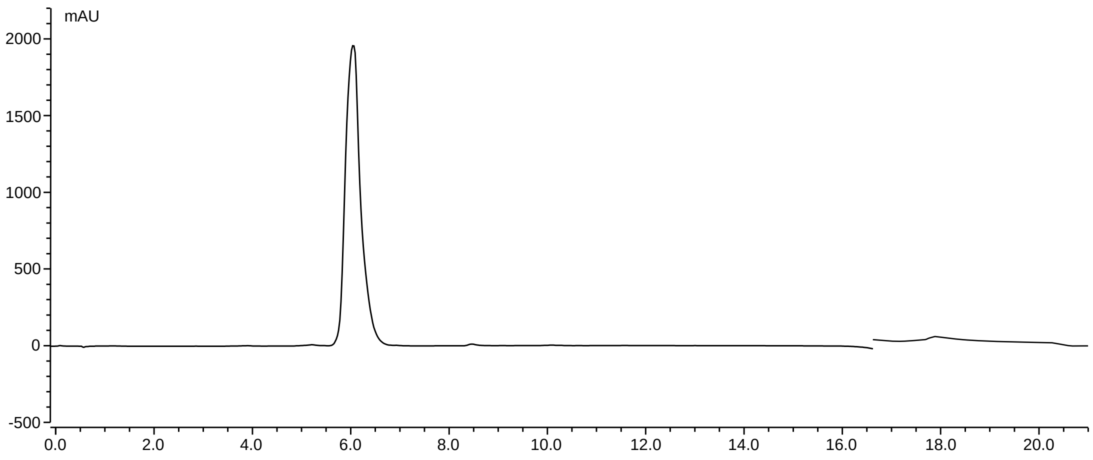

Figure S231: UPLC-DAD chromatogram detected at 270 nm of compound ***cis*-4e**.

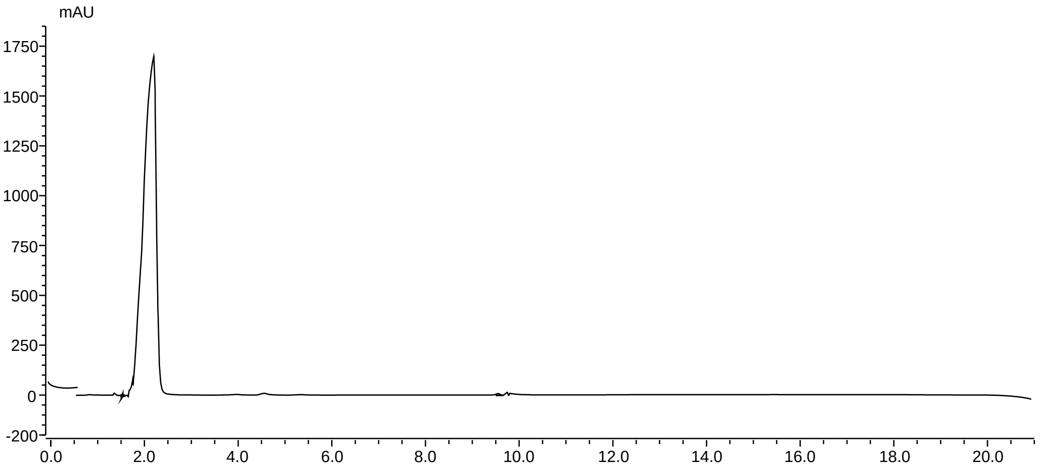

Figure S232: UPLC-DAD chromatogram detected at 270 nm of compound ***cis*-4h**.

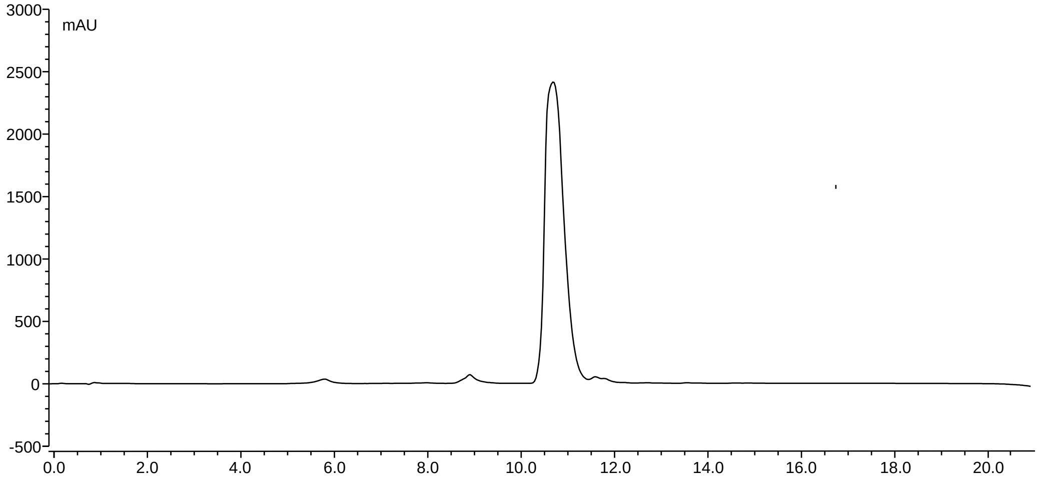

Figure S233: UPLC-DAD chromatogram detected at 270 nm of compound ***trans*-4m**.

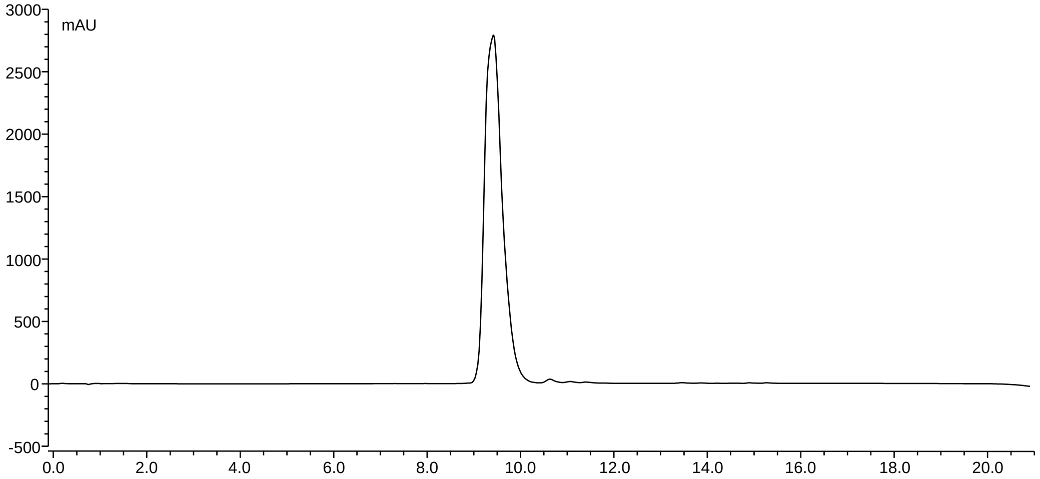

Figure S234: UPLC-DAD chromatogram detected at 270 nm of compound ***cis*-4m**.

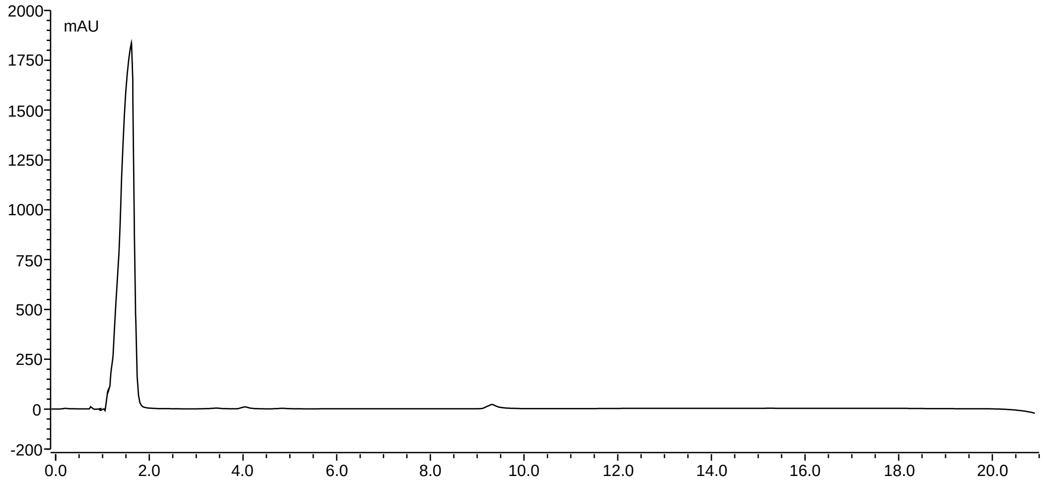

Figure S235: UPLC-DAD chromatogram detected at 270 nm of compound ***cis*-6m**.

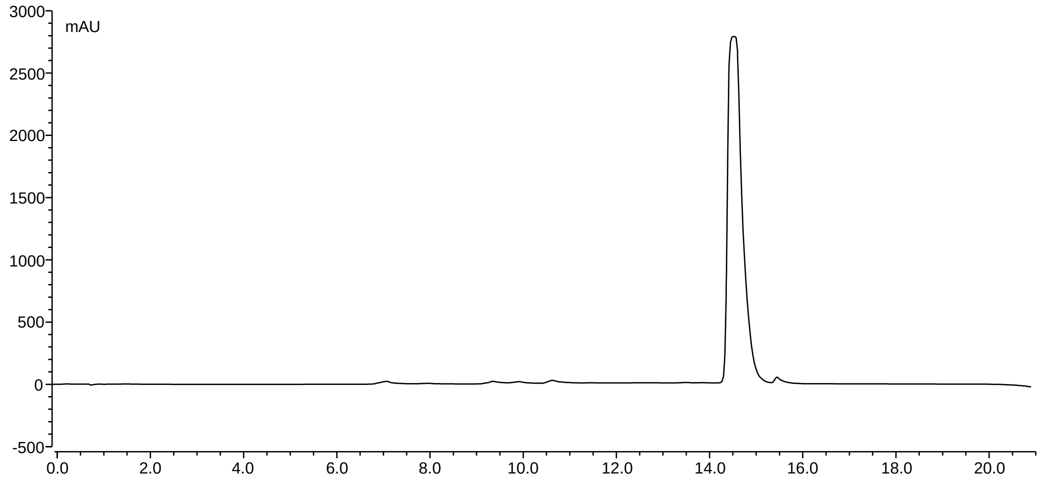

Figure S236: UPLC-DAD chromatogram detected at 270 nm of compound **3m**.
